# A Chiral Amine Transfer Approach to the Photocatalytic Asymmetric Synthesis of α-Trialkyl-α-tertiary Amines

**DOI:** 10.1021/acs.orglett.2c04308

**Published:** 2023-02-01

**Authors:** Georgia R. Harris, Aaron D. Trowbridge, Matthew J. Gaunt

**Affiliations:** Yusuf Hamied Department of Chemistry, University of Cambridge, Lensfield Road, Cambridge, CB2 1EW, U.K.

## Abstract

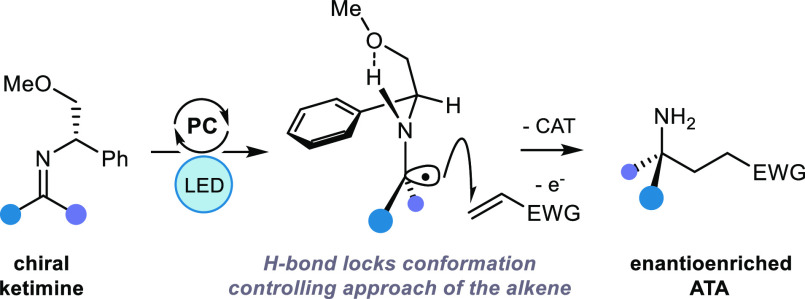

A long-standing challenge within radical chemistry is that of controlling the absolute stereochemistry of the products. Here, we report the stereocontrolled addition of α-amino radicals reductively generated from imines via visible-light-mediated photoredox-catalysis to alkenes, giving rise to enantioenriched α-trialkyl-α-tertiary amines. This process exploits a commercially available phenylglycinol derivative as a source of both nitrogen and chiral information. DFT studies support a stereochemical model whereby an intramolecular H-bond rigidifies the transition state of the enantiodetermining step.

Chiral alkylamines are important features in biologically active small molecules due to their high density of structural information, hydrogen-bonding capabilities, and tunable physical properties.^1^ In particular, alkylamines displaying a stereodefined fully substituted carbon center adjacent to the nitrogen atom, α-trialkyl-α-tertiary amines (α-trialkyl-ATAs), are an important variant of this class of functional molecule and feature in a wide range of pharmaceuticals, agrochemicals, and natural products (Figure 1A).^2^ However, the asymmetric construction of these molecules remains a significant synthetic challenge,^3^ principally due to the steric demands associated with forging fully substituted centers and poor stereodifferentiation between similar alkyl substituents.^4^ Consequently, existing strategies often rely on the relay of chiral information to the fully substituted carbon center via molecular rearrangements,^5^ or by remote functionalization,^6^ where the prochiral fully-substituted center is preformed and subsequently desymmetrized. A more direct approach via the 1,2-addition of organometallics to ketimines has been successful in some cases, with asymmetric induction achieved either through addition to a chiral auxiliary-derived sulfinimine or via catalyst-controlled delivery of the nucleophile to activated imine derivatives, but remains limited in terms of functional group tolerance.^7^

**Figure 1:**
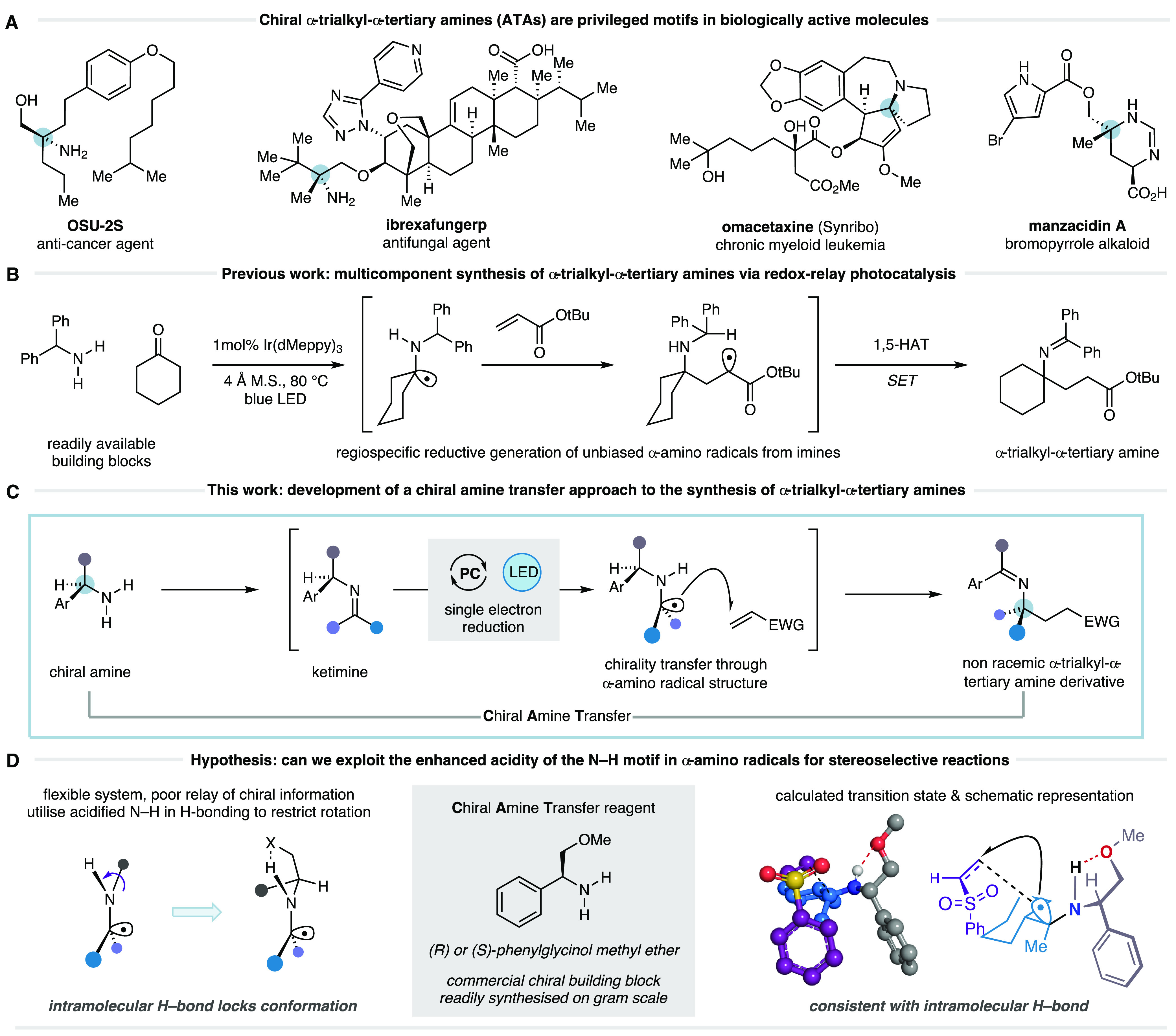
Exploiting α-amino radicals for the asymmetric synthesis of α-trialkyl ATAs.

Over the past decade, photoredox catalysis has become a powerful tool for the synthesis of complex amines through the intermediacy of nucleophilic α-amino radicals that can be generated via a range of activation modes.^8^ In particular, these methods have been utilized for the synthesis of α-trialkyl-ATAs.^9^ Controlling the stereochemistry in reactions with these open shell species, however, remains a significant goal in the field with few examples of transformations that address this problem. Notable cases of the successful enantiocontrol of α-amino radicals include work by Ooi, who demonstrated that chiral phosphoniums can control asymmetric radical–radical coupling reactions.^10^ Phipps has also developed an enantioselective Minisci-type reaction enabled by a chiral BINOL phosphoric acid that binds to both the α-amino radical and heteroarene,^11,12^ while Jiang has reported that chiral BINOL phosphoric acids can mediate enantioselective 1,2-additions of α-amino radicals to vinylpyridines.^13^

Our group recently reported a visible light-mediated catalytic synthesis of α-trialkyl-ATAs from primary benzylic amines, dialkyl ketones, and alkenes (Figure 1B).^14^ Single electron reduction of *in situ* formed imines gave α-amino radicals that could engage alkenes in a 1,2-addition to forge the desired α-trialkyl-ATA products. Crucial to the success of the reaction was the use of benzylic amines, which are thought to drive the redox neutral process by translocating the radicals formed after alkene addition to stabilized aminobenzyl radicals via 1,5-hydrogen atom transfer (HAT).

We envisioned that deployment of an enantiopure chiral benzylamine could mediate a chiral amine transfer process, whereby the stereochemical information and the nitrogen atom are relayed, through reaction of the α-amino radical, to the newly forming fully-substituted center (Figure 1C). The development of such a chiral amine transfer (CAT) reagent would enable the delivery of enantioenriched α-trialkyl-ATA products from simple starting materials. We began by investigating the effect of α-methylbenzylamine, an abundant chiral benzylic amine feedstock, in the photocatalytic hydroaminoalkylation reaction (Figure S1). Unfortunately, low enantiomeric ratios were obtained for the α-trialkyl ATAs derived from several nonsymmetrical ketones and α-methylbenzylamine. These results are, perhaps, unsurprising, given the low barrier of rotation around the C–N axis of the α-amino radical (calc. Δ*G*^‡^_rot_ = 9.8 kcal mol^–1^),^15^ the flexibility of the pendent chiral amine, and the early transition states often associated with radical transformations.^16^ Furthermore, while α-methylbenzylamine has been shown to govern stereochemical outcomes in polar reactions,^17^ diastereoselectivity is typically low in the reactions of α-amino radicals (containing an acyclic chiral amine motif) with alkenes.^18^

In order to overcome these obstacles, we proposed that rigidifying the conformation of the chiral amine fragment, relative to the reacting α-amino radical, would allow for a more efficient transfer of chirality. To this end, we recognized that the N–H bond in an α-amino radical is more acidic than the corresponding amine N–H motif. The ground state hydrogen bonding interaction between alkylamine N–H bonds and ether-type oxygens is considered weak (ca. 2–3 kcal mol^–1^), while the increased acidity of the N–H bond in the corresponding α-amino radical should lead to a stronger H-bonding interaction.^19^ Therefore, we questioned whether the N–H bond in an α-amino radical could be capable of forming an intramolecular hydrogen bond to a pendent Lewis basic atom within the stereodefined chiral amine architecture (Figure 1D). Based on this premise, we considered chiral amines based on the phenylglycinol scaffold, not only because they display a proximal oxygen atom that could fulfill our rigidifying hydrogen bond hypothesis, but also because they are readily accessible, low-cost chiral building blocks.

Concurrent with this, we initiated preliminary computational modeling to provide a theoretical basis for our experimentation. Using the addition of the α-amino radical derived from (*S*)-phenylglycinol methyl ether and cyclohexyl methyl ketone into phenyl vinyl sulfone as a model system, density functional theory (DFT) calculations were performed at wB97XD/6-311′++(d,p) level of theory (see SI for full details). Analysis of the transition states revealed that the lowest energy pathway was consistent with the presence of a hydrogen bond between the N–H of the α-amino radical and the oxygen atom of the ether linkage within the CAT reagent, at the point of radical addition.^20^

Having confirmed the viability of our proposed intramolecular hydrogen bond locked chiral amine transfer reagent, we tested our hypothesis experimentally. The condensation of acyclic ketones with sterically encumbered benzylamines is challenging, often resulting in low conversion to the corresponding imine.^14^ However, we found that stirring the CAT reagent **4a** (1 equiv) and ketone **1c** (2 equiv) at 80 °C in the presence of 20 mol % tris(2,2,2-trifluoroethyl)borate and 4 Å molecular sieves (MS) in CH_2_Cl_2_ for 24 h gave optimal assay yield of the imine (87% by ^1^H NMR, Table S2). Irradiation of the imine and phenyl vinyl sulfone (**2b**, 1.5 equiv) in the presence of 1 mol % Ir(dMeppy)_3_ and 4 Å MS in CH_2_Cl_2_ for 24 h at room temperature resulted in the formation of the α-trialkyl-ATA derived imine (**5**) in 70% yield with an enantiomeric ratio (e.r.) of 81:19. At this stage (*S*)-phenylglycine methyl ester was also tested as a CAT reagent to examine the effect of a carbonyl group as an alternative hydrogen bond acceptor. However, none of the desired product was observed in this case.

With these initial results in hand, we next assessed a range of phenylglycinol derivatives **4a**–**h**, which were prepared using standard literature procedures and displayed a variety of substituents on both the oxygen and the aromatic ring. Beyond the initial reaction (Table 1, entry 1), we found the corresponding *O*-Bn protected phenylglycinol **4b** to be equally effective compared to **4a** (entry 2). The corresponding *O*-PMB ether **4c** resulted in a decrease in yield (entry 3); however, a comparable enantiomeric ratio suggested that the group appended to the oxygen had little effect on the stereochemical outcome. Next, we turned our attention to the aryl group of the CAT reagent. While 4-MeO-substituted **4d** gave poor conversion to product (entry 4), albeit with good e.r., substitution at the meta-positions (**4e**) afforded the desired product in good e.r. and modest yield (entry 5). This can be rationalized by considering the increase in bulk of the aryl group that results from its functionalization along nonlinear exit vectors, which in turn increases the stereodifferentiation between the aryl group and hydrogen atom emanating from the chiral center of the CAT reagent. Moreover, electron-withdrawing substituents on the aromatic ring may serve to further increase H-bond strength through inductive stabilization of the nitrogen anion. No reaction was observed when using the corresponding chiral 1,2-diamine **4f** (entry 6), and α-phenylglycinol derivative **4g** afforded the desired α-trialkyl-ATA in diminished yield and lower e.r. (entry 7). Finally, (*R*)-α-ethylbenzylamine **4h**, where the OMe motif of **4a** is replaced with a methyl group, afforded the α-trialkyl-ATA product in low e.r., consistent with our model requiring an intramolecular hydrogen bond (entry 8). As a result of these observations, we selected (*R*)-phenylglycinol methyl ether **4a** as the optimal CAT reagent based upon its conversion to the desired chiral α-trialkyl-ATA and its availability (as well as being commercial, it can be prepared on a multigram scale from readily available *N*-Boc-(*R*)-phenylglycinol).

**Table 1:**
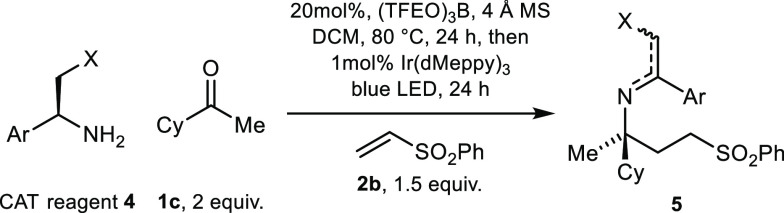
Exploration of CAT Reagents for the Synthesis of Nonracemic α-Trialkyl-α-tertiary Amines

entry	CAT reagent	e.r.^a^	yield (%)^b^
1	**4a**	81:19	70
2	**4b**	84:16	68
3	**4c**	82:18	46
**4**	**4d**	82:18	24
S	**4e**	10:90	52
6	**4f**	–	–
7	**4g**	73:27	47^c^
8	**4h**	32:68	>90^c^

a Enantiomeric ratio determined via derivatization to benzoylated amine and separation using chiral HPLC.

b Yields determined by ^1^H NMR relative to 1,1,2,2-tetrachloroethane internal standard.

c Yield determined via consumption of phenyl vinyl sulfone as determined by ^1^H NMR relative to 1,1,2,2-tetrachloroethane internal standard.
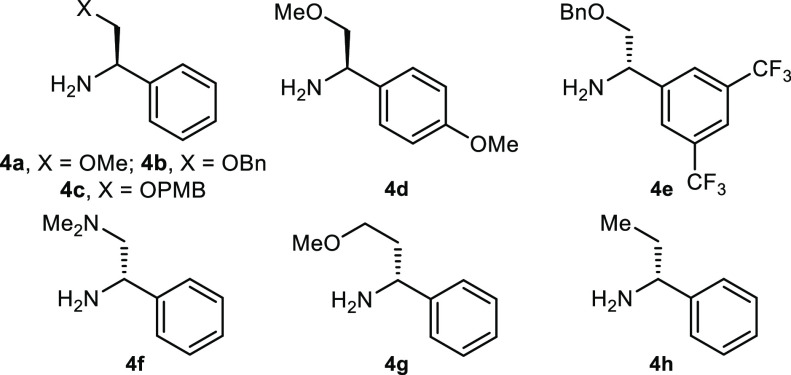

Having identified an optimal stereocontrolled multicomponent system, we next investigated the scope of this asymmetric hydroaminoalkylation reaction. We generally found that the redox-neutral photocatalytic cycle generated the α-trialkyl-ATA products (**5**) as enamines, from which the primary amine could be liberated (Figure 2). While steric challenges associated with the initial imine condensation restricted the ketone alkyl groups, methyl ketones possessing a range of structurally varied α-substituents were found to perform well (Figure 2A). Pleasingly, both cyclohexyl methyl ketone **1c** and isopropyl methyl ketone **1d** gave rise to good levels of stereoselectivity in the α-trialkyl-ATAs products **3c** and **3d**. These results offer a strong indication of the robustness of the transformation as stereodifferentiation between aliphatic substituents is typically regarded as more challenging than distinguishing an aryl ring from an alkyl substituent. Although increasing from 6- to 7-membered cyclic ketone **1e** saw selectivity maintained in ATA **3e**, a slight drop off was observed on reducing to a 4-membered ring **3b**. Saturated heterocycle-methyl ketones, displaying tetrahydrothiopyran and 4-(*N*-Boc)-piperidine motifs, generated the desired products (**3g** and **h**) with the expected selectivity. Ketal protected cyclohexanone **1i** delivered the corresponding ATA **3i** in good yield. Acetyl indane **1j** also formed the desired ATA product **3j** with good selectivity, and X-ray crystallography of 4-nitrbenzoyl protected **3j** confirmed the absolute stereochemistry of the major enantiomer of the product, which was in agreement with the stereochemical model.

**Figure 2:**
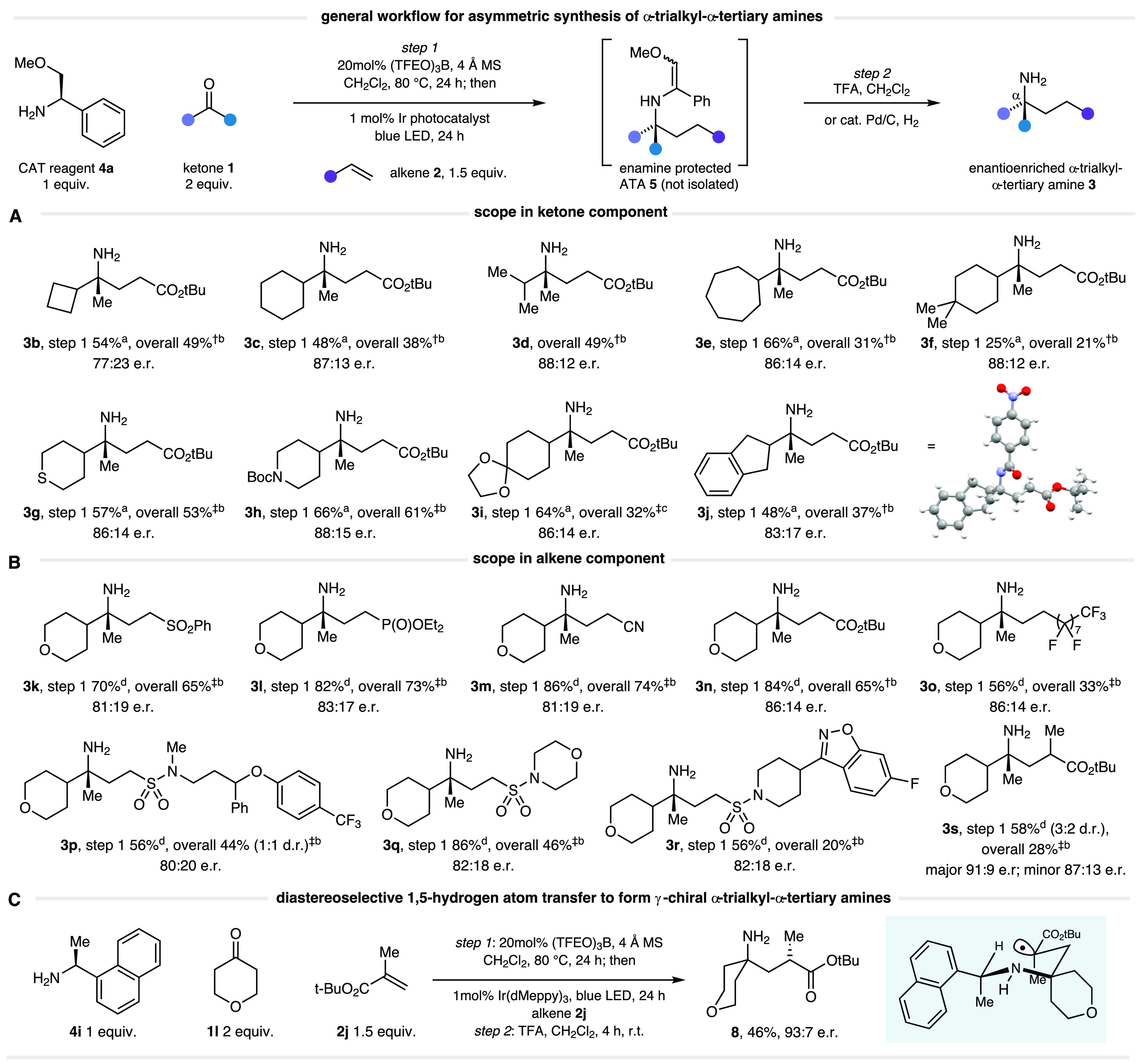
Scope for synthesis of enantioenriched ATAs through the combination of a simple CAT reagent derived from phenylglycinol, ketones, and olefins. Compound **3j**, ellipsoid contour 50% probability level. ^†^Reaction conducted using [Ir(4′-OMeppy)_3_] photocatalyst. ^‡^Reaction conducted using [Ir(dMeppy)_3_] photocatalyst. ^*a* 1^H NMR yield of **3** vs internal standard 1,1,2,2-tetrachloroethane following acid/base workup. ^*b*^Deprotection using TFA/CH_2_Cl_2_, 4 h, r.t. *^c^*Deprotection using cat. Pd/C, H_2_, EtOH, r.t. ^*d* 1^H NMR yield of **5** vs internal standard 1,1,2,2-tetrachloroethane immediately following irradiation.

A range of electron-deficient olefins were found to be excellent coupling partners (Figure 2B), allowing the incorporation of sulfone (**3k**), phosphonate (**3l**), nitrile (**3m**), and ester (**3n**) functionalities in good yields and with consistent levels of stereoselectivity. In addition, perfluoroalkene **2e** afforded the corresponding ATA **3o** with good conversion and selectivity. Vinyl sulfonamides (**2f**–**h**) were also found to be suitable acceptors, delivering a range of complex α-trialkyl-ATAs **3p**–**r**, highlighting the tolerance of the reaction toward medicinally relevant functionality. A 1,1-disubstituted acceptor, *tert*-butyl methacrylate **2i**, generated **3s** in moderate yield as a mixture of diastereomers, but with high selectivity (91:9 e.r.) in the major isomeric component. The low diastereomeric ratio seen in this example can be rationalized by considering the competing factors required in order to set the α vs γ position and the differing associated stereochemical models invoked *vide infra*.

We propose that the reaction proceeds via a redox-neutral photocatalytic cycle, support for which was reported in our previous work on a racemic version of this reaction (Figure S2).^14^ Under this pathway we consider the addition of the α-amino radical to the alkene to be enantiodetermining. However, when unsymmetrical 1,1-disubstituted acceptors are used there is potential for a chiral center to be formed at the γ-position, with 1,5-HAT becoming the enantiodetermining step. While limited diastereoselectivity was observed when amine **4a** was used as the CAT reagent, it led us to question whether further CAT reagent design could enable control of additional stereocenters.

To test this, we conducted a survey of different CAT reagents that were at our disposal (Table S4) and identified commercially available (+)-1-(1-naphthyl)ethylamine **4i** as an effective CAT reagent for controlling the γ-amino center (Figure 2C). Employing tetrahydropyran-4-one **1l** and *tert*-butyl methacrylate **2i** gave rise to γ-chiral α-trialkyl-ATA **8** in moderate yield and high stereoselectivity (93:7 e.r.). This can be rationalized by considering the minimization of 1,3-diaxial interactions between the naphthalene and *tert*-butyl ester in the proposed chairlike transition state of the 1,5-HAT process. Moreover, it highlights that a different set of stereochemical control elements are operational with subtle changes to the nature of the ketone, alkene, and CAT components, providing the potential for a more general approach to the stereoselective reaction of α-amino radicals.

In conclusion, we have developed a multicomponent photoredox-mediated platform for the asymmetric construction of complex α-trialkyl ATAs from simple feedstocks. While the stereoselectivity in this process is not yet optimal, the results presented break new ground in understanding how to control diastereoselective radical reactions toward the formation of enantioenriched products, a challenge to which a general solution remains elusive. We envisage that this distinct approach toward radical selectivity, paired with the practical simplicity of our platform, will inspire further investigation into novel modes of stereocontrol in photocatalytic radical reactions.

## Data Availability

The data underlying this study are available in the published article and its Supporting Information.

## Supporting Information Available

Experimental procedures, DFT calculation, and spectroscopic data for new compounds (PDF)
