# Supplementary material for: A Chiral Amine Transfer Approach to the Photocatalytic Asymmetric Synthesis of α-Trialkyl-α-tertiary Amines
Source: Org Lett. 2023 Feb 1;25(5):861–6. doi: 10.1021/acs.orglett.2c04308 (PMC9926512; doi:10.1021/acs.orglett.2c04308)

Supplementary information

A chiral amine transfer approach to the photocatalytic asymmetric synthesis of α-trialkyl-α-tertiary amines

Georgia R. Harris, Aaron D. Trowbridge, Matthew J. Gaunt

Correspondence to: mjg32@cam.ac.uk

Table of Contents

Supplementary Figures 1
General Experimental 2
Optimisation Studies 3
General Procedures 6
α-Tertiary Amine Products 7
  Ketone Scope 7
  Alkene Scope 13
  Product Derivatisation 20
  Setting the γ-amino centre 21
Starting Material Synthesis 21
  CAT Reagent Synthesis 21
  Ketone Synthesis 25
  Acceptor Synthesis 27
Computational studies 29
X-ray Crystallography Data 51
References 53
Chiral HPLC and SFC Data 54
NMR Spectra 76

Supplementary Figures

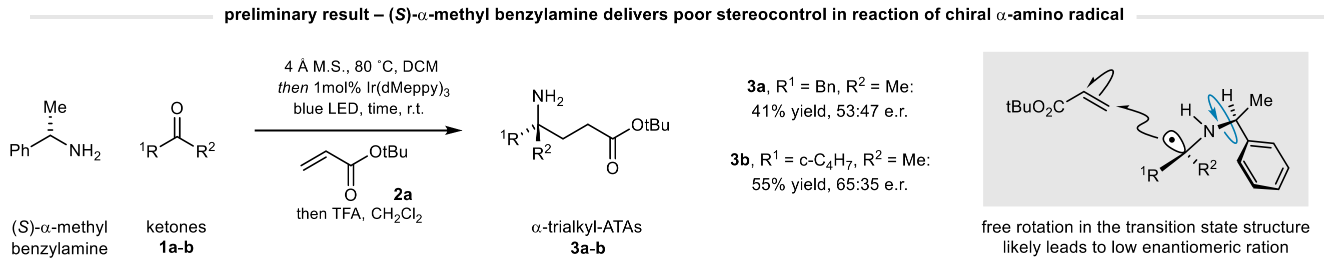

Figure SI1

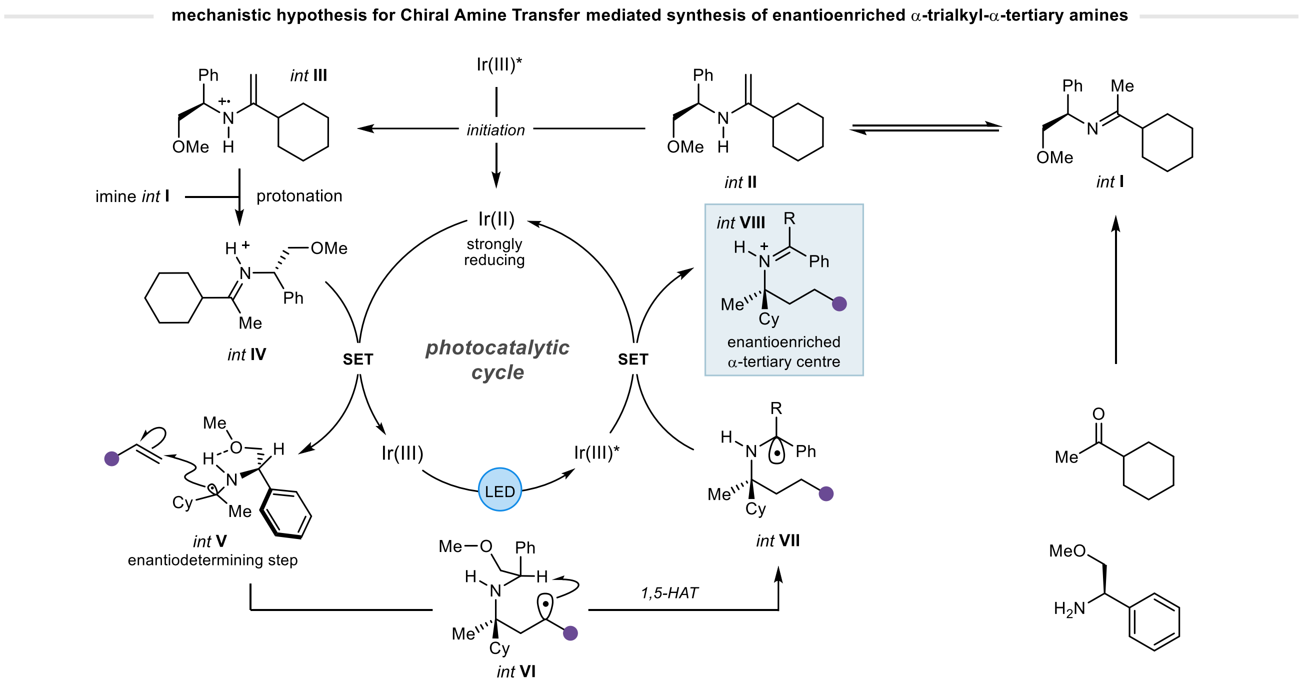

Figure SI2

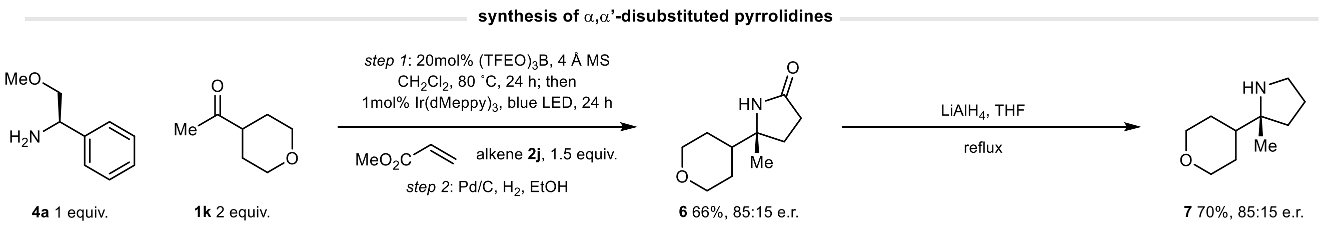

Figure SI3

General Experimental

Solvents and reagents: All reactions were run under an inert atmosphere (N_2) unless otherwise stated, with oven-dried glassware, using standard techniques. Anhydrous solvents were obtained from solvent stills. Dichloromethane used in the photocatalytic reactions was dried using 4Å MS (beads), degassed (freeze-pump-thaw × 3) and stored in a Schlenk flask under N_2. Powdered 4Å molecular sieves (MS) were activated prior to use by heating (250 °C) under high-vacuum (<1 mbar) and stored under N_2 in a round-bottomed flask. Acrylate and acrylonitrile acceptors were distilled prior to use and stored at 5 °C under N_2. Photocatalyst, Ir(dMeppy)$_3$ was prepared according to our previously reported procedure.[1] All other commercial reagents were used as supplied unless otherwise stated.

Reaction setup: Reactions that required heating were heated with an oil bath unless otherwise stated. Irradiation of the reaction mixture was achieved using a 40 W Kessil A160WE LED – Tuna blue aquarium light (max blue, max intensity). Clear hydrolytic glass microwave vials (5 mL) with PTFE/silicon septum lined crimp caps were used as the standard reaction vessel. Vials were placed 5 cm away from the light source, and cooled by an overhead fan.

Chromatography: Analytical thin-layer chromatography (TLC) was performed on Merck Kieselgel 60 F254 0.20 mm precoated, glass backed silica gel plates. Visualization of the developed chromatogram was performed by UV absorbance (λ_{max} = 254 nm), and/or by aqueous $KMnO_4$ or ninhydrin stain.

Flash column chromatography was performed using silica gel (Merck Geduran Si 60 [40-63 µm]) with the indicated solvent system, or using a Teledyne Combi*Flash* NextGen 100 equipped with RediSef Rf columns (40 – 60 µm irregular, normal phase).

Chiral HPLC analysis was performed on a Shimadzu XR high-performance liquid chromatography instrument fitted with either a CHIRALPAK® AD-H, AS, IA, IC or OD column, using a hexane/^{i}PrOH solvent system. Chiral SFC analysis was performed using a Waters ACQUITY UPC2 System with YMC CHIRAL ART SB column (4.6 x 250 mm, 3.0 µm), in a mixed solvent system of supercritical CO_2 and MeOH, and with a system backpressure of 138 bar.

Characterisation: NMR spectra were recorded at 400 MHz, 500 MHz, 600 MHz or 700 MHz on Bruker AM-400/500/600/700 instruments at 298 K unless otherwise specified. Samples were run in deuterated solvents. Chemical shifts (δ) for ^{1}H NMR spectra are reported in parts per million (ppm) relative to tetramethylsilane referenced to residual protic solvent ($CHCl_3$ = 7.26 ppm, C_6D_5H = 7.16 ppm, $CHDCl_2$ = 5.32 ppm, CHD_2OD = 3.31 ppm). Coupling constants (*J*) are reported in Hertz (Hz). Abbreviations for splitting patterns are as follows: s, singlet; d, doublet; t, triplet; q, quartet; m, multiplet. Chemical shifts for ^{13}C NMR spectra were recorded with complete proton decoupling and are reported in ppm from tetramethylsilane referenced to the solvent resonance ($CDCl_3$ = 77.16 ppm, C_6D_6 = 128.06 ppm, CD_2Cl_2 = 53.84 ppm, CD_3OD.= 49.00 ppm). Spectra were analysed using Mestranova or TopSpin software. ^{1}H NMR yields were determined by analysis of crude reaction mixtures with reference to 1,1,2,2-tetrachloroethane as an internal standard.

IR spectra were recorded using neat samples with a Thermo Fisher Scientific Nicolet Summit PRO FTIR Spectrometer. Absorption maxima (ν_{max}) are given in wavenumbers in units of cm^{-1} with characteristic signals assigned.

HRMS experiments were carried out using a Shimadzu LCMS-9030 Q-TOF mass spectrometer.

Melting points (mp) were recorded using a Gallenkamp melting point apparatus and are uncorrected.

Optical rotations were measured on a Perkin Elmer Model 343 polarimeter using a Na lamp (λ 589 nm, D-line).

X-Ray crystallography was performed on a Nonius Kappa CCD or a Bruker D8-Quest Photon-100 at the University of Cambridge Chemistry X-ray laboratory by Dr. Andrew Bond.

Compound names are those generated by PerkinElmer ChemDraw Professional v. 20.1.0.112, according to IUPAC nomenclature.

Optimisation Studies

Optimisation – α-tertiary amino centre

Table SI1: (S)-(-)-α-methylbenzylamine CAT reagent

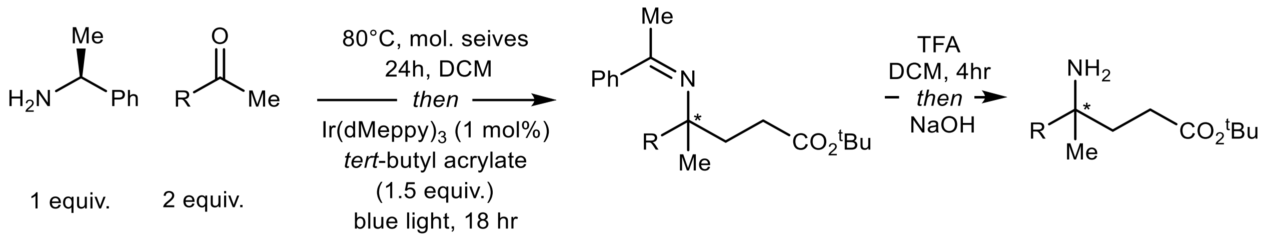

	Ketone	Yield %	e.r.
**1**	Benzyl acetone	41	53:17
**2**	Cyclobutylmethyl ketone	55	65:35

Table SI2: Imine formation

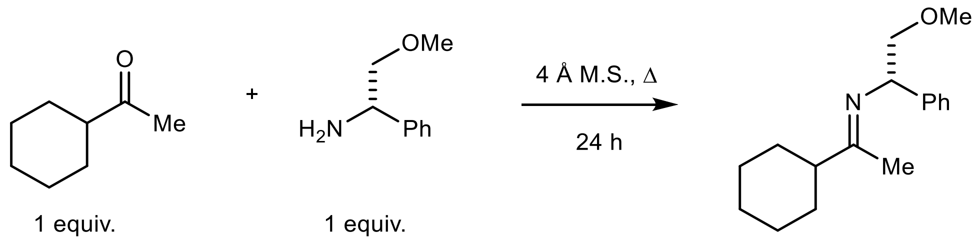

	Temperature, °C	Solvent	Additive, %		Concentration, M	NMR Yield %
**1**	80	DCM	Tris(2,2,2-trifluoroethyl) borate	20	1	87
**2**	80	DCM	$Mg(OTf)_2$<br>Tris(2,2,2-trifluoroethyl) borate	20	1	14
**3**	80	DCM	Tris(2,2,2-trifluoroethyl) borate	20	2	80
**4**	80	DCM	Tris(2,2,2-trifluoroethyl) borate	100	1	70
**5**	100	DCE	Tris(2,2,2-trifluoroethyl) borate	20	1	66
**6**	100	DCE	Tris(2,2,2-trifluoroethyl) borate	100	1	70

**7**	80	DCM	BF_3	20	1	78
**8**	80	DCM	Acetic acid	20	1	32
**9**	100	Toluene	Tris(2,2,2-trifluoroethyl) borate	20	1	64
**10**	100	Toluene	BF_3	20	1	60
**11**	100	Toluene	Acetic Acid	20	1	54

Table SI3: Solvent and temperature screen

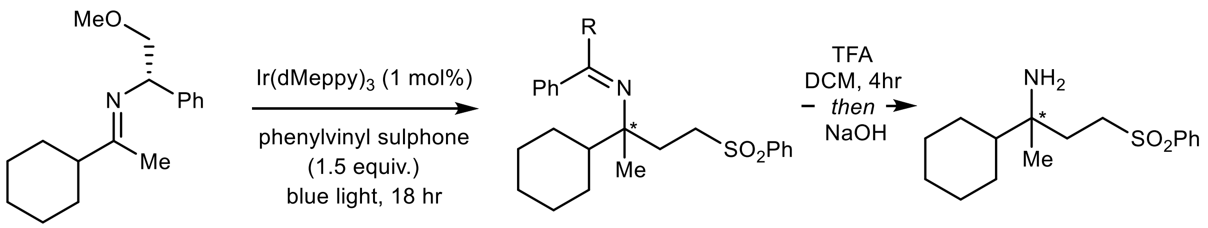

	Solvent: DCM (9:1) *Irradiation*	Temperature *Irradiation*	NMR Yield %	e.e.*
**1**	DCM	r.t.	70	79
**2**	Chloroform	r.t.	-	-
**3**	DCE	r.t.	84	68
**4**	Ether	r.t	-	-
**5**	Toluene	r.t	62	75
**6**	Benzene	r.t	8	-
**7**	Trifluorotoluene	r.t	-	-
**8**	DCM	5 °C	68	69
**9**	DCM	0 °C	40	70

**10**	DCM	-5 °C	58	76

*starting from pre-formed imine stock solution made *via* a dean stark condensation

**determined by Chiral HLPC following product derivatisation to the 4-nitro bezoylated amine

Table SI4: Optimisation – γ-amino centre

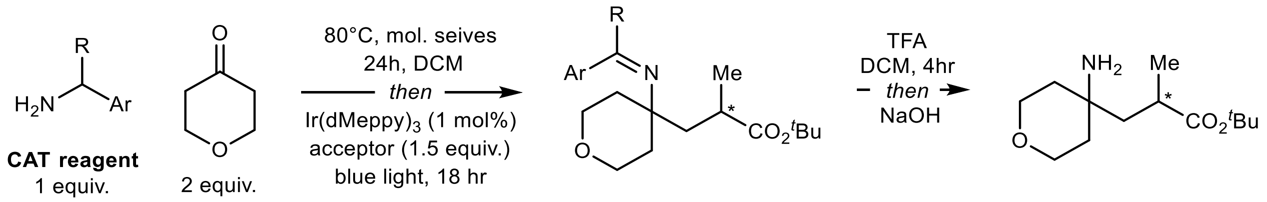

	CAT reagent	Yield %	e.e.
**1**	(S)-(-)-α-methylbenzylamine	64	39
**2**	(S)-(-)-1-(1-Naphthyl)ethylamine	40	86
**3**	(R)-(+)-1-(2-Naphthyl)ethylamine	43	-53
**4**	(R)-2-methoxy-1-phenylethan-1-amine	74	0

*products isolated as the corresponding lactam, following condensation of the primary amine onto the ester

General Procedures

General Procedure A *Setting the α-tertiary amino centre*

An oven-dried microwave vial was charged with a magnetic stirrer bar and activated 4 Å MS (200 mg, 100 mg/0.1 mmol amine). The vial was sealed and evacuate-refilled with N_2 (3 cycles). DCM (1 mL), (R)-2-methoxy-1-phenylethan-1-amine (30 µL, 0.20 mmol, 1.0 equiv), ketone (0.40 mmol, 2.0 equiv) and tris(2,2,2-trifluoroethyl) borate (8.6 µL, 0.04 mmol, 20 mol%) were added sequentially and the reaction mixture heated at 80 °C for 24 h. The reaction mixture was allowed to cool to room temperature and concentrated under vacuum to remove tris(2,2,2-trifluoroethyl) borate prior to irradiation. An iridium photocatalyst (1.0 mol%) and any other solid reagents were added at this point. The vial was then resealed and evacuate-refilled with N_2 (3 cycles). DCM (2 ml) and acceptor (0.30 mmol, 1.5 equiv) were added. The reaction mixture was irradiated for 24 hrs with vigorous stirring. Following irradiation, the reaction mixture was filtered through Celite® and concentrated under vacuum to afford the crude α-tertiary imine. A flask then was charged with the crude imine residue is dissolved in DCM (1 mL), TFA (15 µL, 0.2 mmol, 1 equiv) and a magnetic stirrer bar. The mixture was stirred at room temperature for 4 h. The crude mixture was diluted with PE (60:40) (10 ml), added to 1M HCl (10 ml) and the flask washed out with PE (60:40): Et_2O 1:1 (3ml). The aqueous phase was collected and the organic layer washed with water (10 ml). The combined aqueous layers were washed with PE (60:40): Et_2O 9:1 (10 ml) and then basified with 10% NaOH (2 ml). The product was then extracted into DCM (2 x 10 ml), dried over $NaSO_4$, filtered and concentrated under vacuum to afford the crude α-tertiary primary amine.

Racemic samples were made by the above procedure using benzylamine (22 µL, 0.2 mmol) as the amine.

General Procedure B *Benzoyl derivatisation*

A vial was charged with primary α-tertiary amine (0.1 mmol), 4-nitrobenzoyl chloride (28 mg, 0.15 mmol, 1.5 equiv.), 4-dimethylaminopyridine (2.4 mg, 0.02 mmol, 20 mol%), triethylamine (42 µL, 0.3 mmol, 3 equiv.), DCM (1.5 ml) and a magnetic stirrer bar. The mixture was stirred overnight and the crude reaction loaded directly on to a pipette column (silica gel, EtOAc in PE (60:40)) to obtain the benzoyl protected product derivatives.

General Procedure C *Setting the γ-amino centre*

An oven-dried microwave vial was charged with a magnetic stirrer bar and activated 4 Å MS (200 mg, 100 mg/0.1 mmol amine). The vial was sealed and evacuate-refilled with N_2 (3 cycles). DCM (1 mL), amine (0.20 mmol, 1.0 equiv) and 4-oxotetrahydropyran (37 µL, 0.40 mmol, 2.0 equiv) were added sequentially and the reaction mixture heated at 80 °C for 24 h. A second microwave vial was charged with $Ir(dMeppy)_3$ (1.5 mg, 1 mol%), sealed, wrapped with Parafilm™, and back-refilled with nitrogen (x3). Dry degassed CH_2Cl_2 (1 mL) was added. The photocatalyst solution and acceptor (0.3 mmol, 1.5 equiv) were added to the cooled reaction mixture, and the vial was irradiated with blue light for 18h. Following irradiation, the reaction mixture was filtered through Celite® and concentrated under vacuum to afford the crude α-tertiary imine. A flask was then charged with the crude imine, palladium on carbon (106 mg, 10% *w/w*, 5 mol% Pd) and a magnetic stirrer bar, and back-refilled with N_2 (3 cycles). EtOH (1ml) was added, and the flask back-refilled with H_2 (3 cycles). The mixture was stirred under a positive pressure of hydrogen for 16h. The flask was

back-refilled with N_2 (3 cycles) and the reaction mixture filtered through celite and concentrated *in vacuo* to afford the crude α-tertiary primary amine.

Racemic samples were made by the above procedure using benzylamine (22 µL, 0.2 mmol) as the amine.

α-Tertiary Amine Products

Ketone Scope

tert-butyl (R)-4-amino-4-cyclobutylpentanoate, 3b

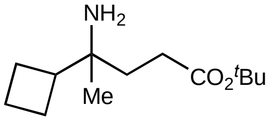

Prepared according to general procedure A using cyclobutyl methyl ketone (44 µL, 0.40 mmol, 2.0 equiv) as the ketone and *tert*-butyl acrylate (44 µL, 0.30 mmol, 1.5 equiv) as the acceptor, and Ir(4'-OMeppy)$_3$ (1.5 mg) as the photocatalyst. Purification by acid/base extraction afforded the title compound as pale yellow oil (54% yield by ^{1}H qNMR; >96% purity). Further purification by automated flash column chromatography (RediSep Rf Gold 4.0 g, 1–20% [20% {1.75 M NH_3 in MeOH} in DCM] DCM) gave the title compound as a pale yellow oil (22.2 mg, 49%, e.r. 77:23). **^{1}H NMR** (400 MHz, $CDCl_3$) δ: 2.33 – 2.18 (m, 3H), 1.83 – 1.76 (m, 5H), 1.68 – 1.62 (m, 1H), 1.57 – 1.48 (m, 2H), 1.43 (s, 9H), 0.90 (s, 3H). **^{13}C NMR** (101 MHz, $CDCl_3$) δ: 173.8, 80.2, 51.9, 46.2, 35.3, 30.6, 28.2, 23.8, 22.9, 22.6, 17.0. **IR** ν_{max}/cm^{-1}: 2972, 1725, 1456, 1391, 1366, 1304, 1255, 1150. **HRMS** m/z calculated for $C_{13}H_{25}NO_2$ $[M+H]^+$ 228.1958, found 228.1962 (Δ = 1.9 ppm). $[\alpha]_D^{20}$ = −2.2 (*c* = 0.5).

tert-butyl (R)-4-cyclobutyl-4-(4-nitrobenzamido)pentanoate

tert-butyl (*R*)-4-amino-4-cyclobutylpentanoate was derivatised using general procedure B for the purpose of enantiomeric ratio determination. This gave the title compound, whose enantiomers were more readily separable by chiral HPLC. **^{1}H NMR** (400 MHz, $CDCl_3$) δ: 8.25 (d, *J* = 8.6 Hz, 2H), 7.91 (d, *J* = 8.6 Hz, 2H), 6.62 (br. s, 1H), 2.96 (qt, *J* = 9.0 Hz, 1H), 2.36 (t, *J* = 7.5 Hz, 2H), 2.12 (dt, *J* = 7.1, 14.4 Hz, 1H), 2.00 – 1.79 (m, 6H), 1.75 – 1.65 (m, 1H), 1.43 (s, 3H), 1.42 (s, 9H). **HRMS** m/z calculated for $C_{20}H_{28}N_2O_5$ $[M+H]^+$ 377.2071, found 377.2069 (Δ = 0.7 ppm), 321.1450 $[M-C_4H_8]^+$. **Chiral HPLC** Chiralpak AD-H (Hexane/iPrOH = 85/15, 1.0 mL min^{-1}, 30 °C) t_R = 7.7 (major), 9.5 (minor) minutes.

tert-butyl (R)-4-amino-4-cyclohexylpentanoate, 3c

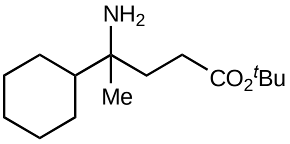

Prepared according to general procedure A using cyclohexyl methyl ketone (55 µL, 0.40 mmol, 2.0 equiv) as the ketone and *tert*-butyl acrylate (44 µL, 0.30 mmol, 1.5 equiv) as the acceptor, and Ir(4'-OMeppy)$_3$ (1.5 mg) as the photocatalyst. Purification by acid/base extraction afforded the title compound as pale yellow oil (48% yield by ^{1}H qNMR; >92%

purity). Further purification by automated flash column chromatography (RediSep Rf Gold 4.0 g, 1–20% [20% {1.75 M NH_3 in MeOH} in DCM] DCM) gave the title compound as a pale yellow oil (19.4 mg, 38%, e.r. 87:13). **^{1}H NMR** (400 MHz, $CDCl_3$) δ: 2.28 – 2.22 (m, 2H), 1.82 – 1.69 (m, 4H), 1.69 – 1.60 (m, 3 H), 1.43 (s, 9H), 1.28 – 0.96 (m, 8H), 0.95 (s, 3H). **^{13}C NMR** (101 MHz, $CDCl_3$) δ: 173.9, 80.2, 53.1, 47.9, 35.3, 30.4, 28.2, 27.4, 27.1, 27.0, 26.7, 25.1. **IR** ν_{max} /cm^{-1}: 2924, 2852, 1725, 1449, 1391, 1366, 1302, 1255, 1148. **HRMS** m/z calculated for $C_{15}H_{30}NO_2$ [M+H]$^+$ 256.2271, found 256.2273 (Δ = 0.9 ppm). $[\alpha]_D^{20}$ = +1.0 (*c* = 0.5).

***tert*-butyl (*R*)-4-cyclohexyl-4-(4-nitrobenzamido)pentanoate**

tert-butyl (*R*)-4-amino-4-cyclohexylpentanoate was derivatised using general procedure B for the purpose of enantiomeric ratio determination. This gave the title compound, whose enantiomers were more readily separable by chiral HPLC. **^{1}H NMR** (400 MHz, $CDCl_3$) δ: 8.25 (d, *J* = 9.0 Hz, 2H), 7.94 (d, *J* = 9.0 Hz, 2H), 6.83 (br. s, 1H), 2.39 (t, *J* = 6.8 Hz, 2H), 2.29 – 2.17 (m, 2H), 1.83 – 1.84 (m, 6H), 1.43 (s, 3H), 1.42 (s, 9H), 1.33 – 1.19 (m, 2H), 1.16 – 1.00 (m, 3H). **HRMS** m/z calculated for $C_{22}H_{32}N_2O_5$ [M+H]$^+$ 405.2384, found 405.2382 (Δ = 0.5 ppm), 349.1761 [M−C_4H_8]$^+$. **Chiral HPLC** Chiralpak AD-H (Hexane/iPrOH = 85/15, 1.0 mL min^{-1}, 30 °C) t_R = 6.6 (major), 9.6 (minor) minutes.

***tert*-butyl (*R*)-4-amino-4,5-dimethylhexanoate, 3d**

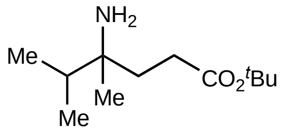

Prepared according to general procedure A using 3-methyl-2-butanone (43 µL, 0.40 mmol, 2.0 equiv) as the ketone and *tert*-butyl acrylate (44 µL, 0.30 mmol, 1.5 equiv) as the acceptor, and Ir(4'-OMeppy)$_3$ (1.5 mg) as the photocatalyst. Purification by automated flash column chromatography (RediSep Rf Gold 4.0 g, 1–20% [20% {1.75 M NH_3 in MeOH} in DCM] DCM) gave the title compound as a pale yellow oil (21.1 mg, 49%, e.r. 88:12). **^{1}H NMR** (400 MHz, $CDCl_3$) δ: 2.27 (app. q, *J* = 8.1 Hz, 2H), 1.64 (t, *J* = 8.1 Hz, 2H), 1.55 (sept, *J* = 6.6 Hz, 1H), 1.44 (s, 9H), 0.95 (s, 3H), 0.89 (d, *J* = 6.6 Hz, 3H), 0.88 (d, *J* = 6.6 Hz, 3H). **^{13}C NMR** (101 MHz, $CDCl_3$) δ: 173.9, 80.2, 53.3, 37.3, 35.2, 30.5, 28.2, 24.3, 17.5, 17.1. **IR** ν_{max} /cm^{-1}: 2966, 2876, 1727, 1457, 1391, 1367, 1306, 1256, 1151. **HRMS** m/z calculated for $C_{12}H_{25}NO_2$ [M+H]$^+$ 216.1958, found 216.1959 (Δ = 0.3 ppm). $[\alpha]_D^{20}$ = −3.0 (*c* = 0.1).

***tert*-butyl (*R*)-4,5-dimethyl-4-(4-nitrobenzamido)hexanoate**

tert-butyl (*R*)-4-amino-4,5-dimethylhexanoate was derivatised using general procedure B for the purpose of enantiomeric ratio determination. This gave the title compound, whose enantiomers were more readily separable by chiral HPLC. **^{1}H NMR** (400 MHz, $CDCl_3$) δ: 8.26 (d, *J* = 9.0 Hz, 2H), 7.94 (d, *J* = 9.0 Hz, 2H), 6.87 (br. s, 1H), 2.68 (sept, *J* = 7.0 Hz, 1H), 2.40 (t, *J* = 6.6 Hz, 2H), 2.21 (dt, *J* = 7.0, 14.5 Hz, 1H), 1.78 (dt, *J* = 7.0, 14.5 Hz, 1H), 1.43 (s, 12H), 0.96 (d, *J* = 6.6 Hz, 3H), 0.95 (d, *J* = 6.6 Hz, 3H). **HRMS** m/z calculated for $C_{19}H_{28}N_2O_5$ [M+H]$^+$ 365.2071, found 365.2072 (Δ = 0.4 ppm), 309.1450 [M−C_4H_8]$^+$. **Chiral HPLC** Chiralpak AD-H (Hexane/iPrOH = 85/15, 1.0 mL min^{-1}, 30 °C) t_R = 6.4 (major), 9.4 (minor) minutes.

tert-butyl (_R_)-4-amino-4-cycloheptylpentanoate, 3e

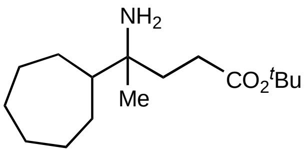

Prepared according to general procedure A using cycloheptyl methyl ketone (56 mg, 0.40 mmol, 2.0 equiv) as the ketone and *tert*-butyl acrylate (44 µL, 0.30 mmol, 1.5 equiv) as the acceptor, and Ir(4'-OMeppy)$_3$ (1.5 mg) as the photocatalyst. Purification by acid/base extraction afforded the title compound as pale yellow oil (66% yield by ^{1}H qNMR; 75% purity). Further purification by automated flash column chromatography (RediSep Rf Gold 4.0 g, 1–20% [20% {1.75 M NH_3 in MeOH} in DCM] DCM) gave the title compound as a pale yellow oil (16.7 mg, 31%, e.r. 86:14). **^{1}H NMR** (400 MHz, $CDCl_3$) δ: 2.25 (td, *J* = 3.6, 8.2 Hz, 2H), 1.80 – 1.62 (m, 6H), 1.55 – 1.37 (m, 16H), 1.32 – 1.17 (m, 4H), 0.95 (s, 3H). **^{13}C NMR** (101 MHz, $CDCl_3$) δ: 177.0 (minor), 173.9, 80.2, 62.8 (minor), 54.3, 49.3 (minor), 49.2, 35.2, 32.6 (minor), 30.5, 30.4 (minor), 29.6 (minor), 29.1 (minor), 28.9, 28.5, 28.4, 28.3, 28.1, 28.0, 27.9 (minor), 27.6 (minor), 27.5 (minor), 24.8, 23.6 (minor). *Multiple peaks appear as major and minor likely due to cycloheptyl ring flipping.* **IR** ν_{max} /cm^{-1}: 2919, 2854, 1726, 1694, 1456, 1391, 1366, 1302, 1255, 1150. **HRMS** m/z calculated for $C_{16}H_{31}NO_2$ $[M+H]^+$ 270.2428, found 270.2430 (Δ = 0.9 ppm). $[\alpha]_D^{20}$ = −1.0 (*c* = 0.1).

tert-butyl (_R_)-4-cycloheptyl-4-(4-nitrobenzamido)pentanoate

tert-butyl (*R*)-4-amino-4-cycloheptylpentanoate was derivatised using general procedure B for the purpose of enantiomeric ratio determination. This gave the title compound, whose enantiomers were more readily separable by chiral HPLC. **^{1}H NMR** (400 MHz, $CDCl_3$) δ: 8.25 (d, *J* = 8.7 Hz, 2H), 7.94 (d, *J* = 8.7 Hz, 2H), 7.01 (br. s, 1H), 2.51 (tt, *J* = 3.3, 10.1 Hz, 1H), 2.40 (t, *J* = 6.5 Hz, 2H), 2.19 (dt, *J* = 7.2, 14.5 Hz, 1H), 1.83 – 1.64 (m, 5H), 1.63 – 1.52 (m, 2H), 1.52 – 1.39 (m, 16H), 1.36 – 1.26 (m, 2H). **HRMS** m/z calculated for $C_{23}H_{34}N_2O_5$ $[M+H]^+$ 419.2541, found 419.2541 (Δ = 0.2 ppm), 366.1920 $[M-C_4H_8]^+$. **Chiral HPLC** Chiralpak AD-H (Hexane/iPrOH = 85/15, 1.0 mL min^{-1}, 30 °C) t_R = 6.3 (major), 9.8 (minor) minutes.

tert-butyl (_R_)-4-amino-4-(4,4-dimethylcyclohexyl)pentanoate, 3f

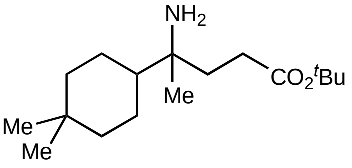

Prepared according to general procedure A using 4,4-dimethylcyclohexyl methyl ketone (62 mg, 0.40 mmol, 2.0 equiv) as the ketone and *tert*-butyl acrylate (44 µL, 0.30 mmol, 1.5 equiv) as the acceptor, and Ir(4'-OMeppy)$_3$ (1.5 mg) as the photocatalyst. Purification by acid/base extraction afforded the title compound as pale yellow oil (25% yield by ^{1}H qNMR; 80% purity). Further purification by automated flash column chromatography (RediSep Rf Gold 4.0 g, 1–20% [20% {1.75 M NH_3 in MeOH} in DCM] DCM) gave the title compound as a pale yellow oil (12.3 mg, 22%, e.r. 88:12). **^{1}H NMR** (400 MHz, $CDCl_3$) δ: 2.26 (td, *J* = 3.9, 8.0 Hz, 2H), 1.65 (dd, *J* = 7.3, 9.1 Hz, 2H), 1.60 – 1.49 (m, 2H), 1.47 – 1.40 (m,

11H), 1.28 – 1.10 (m, 7H), 0.98 (s, 3H), 0.88 (s, 3H), 0.86 (s, 3H). **^{13}C NMR** (101 MHz, $CDCl_3$) δ: 173.9, 80.2, 53.0, 47.9, 39.8, 35.5, 30.4, 30.0, 28.3, 25.2, 24.2, 23.0, 22.6. **IR** ν_{max}/cm^{-1}: 2942, 1728, 1456, 1366, 1301, 1256, 1150. **HRMS** m/z calculated for $C_{17}H_{33}NO_2$ [M+H]$^+$ 284.2584, found 284.2588 (Δ = 1.4 ppm). $[\alpha]_D^{20}$ = −1.3 (*c* = 0.3).

tert-butyl (R)-4-(4,4-dimethylcyclohexyl)-4-(4-nitrobenzamido)pentanoate

tert-butyl (*R*)-4-amino-4-(4,4-dimethylcyclohexyl)pentanoate was derivatised using general procedure B for the purpose of enantiomeric ratio determination. This gave the title compound, whose enantiomers were more readily separable by chiral HPLC. **^{1}H NMR** (400 MHz, $CDCl_3$) δ: 8.25 (d, *J* = 9.0 Hz, 2H), 7.94 (d, *J* = 9.0 Hz, 2H), 6.87 (br. s, 1H), 2.40 (t, *J* = 6.8 Hz, 2H), 2.27 – 2.14 (m, 2H), 1.81 (dt, *J* = 6.2, 14.5 Hz, 1H), 1.59 – 1.50 (m, 2H), 1.45 (s, 3H), 1.42 (s, 9H), 1.37 – 1.17 (m, 5H), 0.89 (s, 3H), 0.86 (s, 3H). **HRMS** m/z calculated for $C_{24}H_{36}N_2O_5$ [M+H]$^+$ 433.2697, found 433.2696 (Δ = 0.2 ppm), 377.2041 [M−C_4H_8]$^+$. **Chiral HPLC** Chiralpak AD-H (Hexane/iPrOH = 85/15, 1.0 mL min^{-1}, 30 °C) t_R = 8.2 (major), 10.9 (minor) minutes.

***tert*-butyl (*R*)-4-amino-4-(tetrahydro-2*H*-thiopyran-4-yl)pentanoate, 3g**

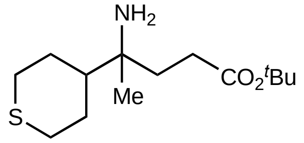

Prepared according to general procedure A using 1-(tetrahydro-2*H*-thiopyran-4-yl)ethan-1-one (58 mg, 0.40 mmol, 2.0 equiv) as the ketone and *tert*-butyl acrylate (44 µL, 0.30 mmol, 1.5 equiv) as the acceptor, and Ir(dMeppy)$_3$ (1.5 mg) as the photocatalyst. Purification by acid/base extraction afforded the title compound as pale yellow oil (57% yield by ^{1}H qNMR; 94% purity). Further purification by automated flash column chromatography (RediSep Rf Gold 4.0 g, 1–20% [20% {1.75 M NH_3 in MeOH} in DCM] DCM) gave the title compound as a pale yellow oil (28.8 mg, 53%, e.r. 84:16). **^{1}H NMR** (400 MHz, $CDCl_3$) δ: 2.69 – 2.63 (m, 4H), 2.24 (td, *J* = 2.6, 7.8 Hz, 2H), 2.15 (app. d, *J* = 13.3 Hz, 1H), 2.08 (app. d, *J* = 12.6 Hz, 1H), 1.65 (dd, *J* = 7.1, 8.9 Hz, 2H), 1.46 – 1.38 (m, 12H), 1.18 (app. d, *J* = 11.8 Hz 1H), 0.97 (s, 3H). **^{13}C NMR** (101 MHz, $CDCl_3$) δ: 173.5, 80.4, 53.5, 47.6, 34.9, 30.2, 29.64, 29.62, 28.9, 28.5, 28.2, 24.8. **IR** ν_{max}/cm^{-1}: 2934, 1723, 1456, 1428, 1366, 1299, 1255, 1147. **HRMS** m/z calculated for $C_{14}H_{27}NO_2S$ [M+H]$^+$ 274.1835, found 274.1841 (Δ = 2.1 ppm). $[\alpha]_D^{20}$ = +7.2 (*c* = 0.5).

tert-butyl (*R*)-4-(4-nitrobenzamido)-4-(tetrahydro-2H-thiopyran-4-yl)pentanoate

tert-butyl (*R*)-4-amino-4-(tetrahydro-2*H*-thiopyran-4-yl)pentanoate was derivatised using general procedure B for the purpose of enantiomeric ratio determination. This gave the title compound, whose enantiomers were more readily separable by chiral HPLC. **^{1}H NMR** (400 MHz, $CDCl_3$) δ: 8.25 (d, *J* = 8.8 Hz, 2H), 7.96 (d, *J* = 8.8 Hz, 2H), 7.20 (br. s, 1H), 2.79 – 2.60 (m, 4H), 2.51 – 2.40 (m, 3H), 2.19 (dt, *J* = 7.0, 14.8 Hz, 1H), 2.09 (app. t, *J* = 13.5, 2H), 1.70 (dt, *J* = 5.7, 14.8 Hz, 1H), 1.59 – 1.46 (m, 2H), 1.45 (s, 3H), 1.43 (s, 9H). **HRMS** m/z calculated for $C_{21}H_{30}N_2O_5S$ [M+H]$^+$ 423.1948, found 423.1950 (Δ = 0.4 ppm), 367.1281 [M−C_4H_8]$^+$. **Chiral HPLC** Chiralpak AD-H (Hexane/iPrOH = 85/15, 1.0 mL min^{-1}, 30 °C) t_R = 9.8 (major), 16.5 (minor) minutes.

tert-butyl (_R_)-4-(2-amino-5-(_tert_-butoxy)-5-oxopentan-2-yl)piperidine-1-carboxylate, 3h

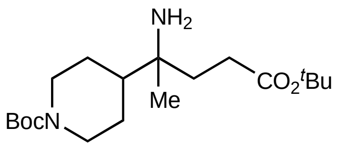

Prepared according to general procedure A using *tert*-butyl 4-acetylpiperidine-1-carboxylate (91 mg, 0.40 mmol, 2.0 equiv) as the ketone and *tert*-butyl acrylate (44 µL, 0.30 mmol, 1.5 equiv) as the acceptor, and Ir(dMeppy)$_3$ (1.5 mg) as the photocatalyst. Purification by acid/base extraction afforded the title compound as pale yellow oil (66% yield by ^{1}H qNMR; >96% purity). Further purification by automated flash column chromatography (RediSep Rf Gold 4.0 g, 1–20% [20% {1.75 M NH_3 in MeOH} in DCM] DCM) gave the title compound as a pale yellow oil (43.6 mg, 61%, e.r. 85:15). **^{1}H NMR** (400 MHz, $CDCl_3$) δ: 4.16 (br. s, 2H), 2.60 (br. t, *J* = 10.6 Hz, 2H), 2.25 (td, *J* = 5.0, 7.6 Hz, 2H), 1.71 – 1.57 (m, 4H), 1.43 (s, 9H), 1.42 (s, 9H), 1.30 – 1.02 (m, 5H), 0.95 (s, 3H). **^{13}C NMR** (101 MHz, $CDCl_3$) δ: 173.6, 154.5, 80.3, 79.4, 52.7, 46.5, 44.3 (br.), 35.2, 30.3, 28.6, 28.2, 26.6, 26.2, 24.8. **IR** ν_{max}/cm^{-1}: 2794, 1725, 1686, 1422, 1391, 1364, 1280, 1237, 1148, 1031. **HRMS** m/z calculated for $C_{19}H_{36}N_2O_4$ [M+H]$^+$ 357.2748, found 357.2748 (Δ = 0.1 ppm). $[\alpha]_D^{20}$ = +1.2 (*c* = 0.5).

tert-butyl (_R_)-4-(5-(_tert_-butoxy)-2-(4-nitrobenzamido)-5-oxopentan-2-yl)piperidine-1-carboxylate

tert-butyl (*R*)-4-(2-amino-5-(*tert*-butoxy)-5-oxopentan-2-yl)piperidine-1-carboxylate was derivatised using general procedure B for the purpose of enantiomeric ratio determination. This gave the title compound, whose enantiomers were more readily separable by chiral HPLC. **^{1}H NMR** (400 MHz, $CDCl_3$) δ: 8.25 (d, *J* = 8.8 Hz, 2H), 7.95 (d, *J* = 8.8 Hz, 2H), 7.12 (br. s, 1H), 4.21 – 4.16 (m, 2H), 2.66 (app. q, *J* = 12.2 Hz, 2H), 2.55 (tt, *J* = 3.0, 12.2 Hz, 1H), 2.42 (t, *J* = 6.6 Hz, 2H), 2.21 (dt, *J* = 6.9, 14.8 Hz, 1H), 1.72 – 1.60 (m, 3H), 1.44 (s, 12H), 1.42 (s, 9H), 1.33 – 1.23 (m, 2H). **HRMS** m/z calculated for $C_{26}H_{39}N_3O_7$ [M+H]$^+$ 506.2861, found 506.2861 (Δ = 0.1 ppm), 406.2335 [M−$C_5H_9O_2$]$^+$, 350.1712 [M−$C_9H_{17}O_2$]$^+$. **Chiral HPLC** Chiralpak AD-H (Hexane/iPrOH = 85/15, 1.0 mL min^{-1}, 30 °C) t_R = 7.6 (major), 10.3 (minor) minutes.

tert-butyl (_R_)-4-amino-4-(1,4-dioxaspiro[4.5]decan-8-yl)pentanoate, 3i

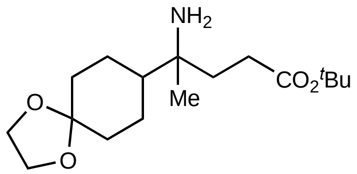

Prepared according to general procedure A using 1-(1,4-dioxaspiro[4.5]decan-8-yl)ethan-1-one (74 mg, 0.40 mmol, 2.0 equiv) as the ketone and *tert*-butyl acrylate (44 µL, 0.30 mmol, 1.5 equiv) as the acceptor, and Ir(dMeppy)$_3$ (1.5 mg) as the photocatalyst. Following irradiation, the reaction mixture was filtered through Celite® and concentrated under vacuum to afford the crude α-tertiary imine. A flask was then charged with the crude imine, palladium on carbon (106 mg, 10% *w/w*, 5 mol% Pd) and a magnetic stirrer bar, and back-refilled with N_2 (3 cycles). EtOH (1ml) was added, and the flask back-refilled with H_2 (3 cycles). The mixture was stirred under a positive pressure of hydrogen for 16h.

The flask was back-refilled with N_2 (3 cycles) and the reaction mixture filtered through celite and concentrated *in vacuo* to afford the crude α-tertiary primary amine as pale yellow oil (64% yield by ^{1}H qNMR; contains ca. 30% starting ketone). Further purification by automated flash column chromatography (RediSep Rf Gold 4.0 g, 1–20% [20% {1.75 M NH_3 in MeOH} in DCM] DCM) gave the title compound as a pale yellow oil (20.0 mg, 32%, e.r. 86:14). **^{1}H NMR** (400 MHz, $CDCl_3$) δ: 3.92 (s, 4H), 2.31 (dd, *J* = 6.7, 9.4 Hz, 2H), 1.84 – 1.71 (m, 6H), 1.52 (td, *J* = 4.0, 11.5 Hz), 1.46 – 1.37 (m, 13H), 1.08 (s, 3H). **^{13}C NMR** (101 MHz, $CDCl_3$) δ: 174.1 (minor), 173.7, 109.0 (minor), 108.8, 80.2, 80.0 (minor), 64.3, 55.8 (minor), 52.9, 46.8, 43.5, 35.6, 35.33 (minor), 35.32 (minor), 35.1, 31.1 (minor), 30.4, 30.0 (minor), 28.24 (minor), 28.21, 25.0, 24.8 (minor), 24.6, 24.2, 24.1 (minor), 21.3 (minor), 16.1 (minor). *Multiple peaks appear as major and minor likely due to conformational change of the cyclohexyl ring.* **IR** ν_{max}/cm^{-1}: 2942, 2873, 1723, 1447, 1366, 1300, 1248, 1145, 1099, 1036. **HRMS** m/z calculated for $C_{17}H_{31}NO_4$ [M+H]$^+$ 314.2326, found 314.2329 (Δ = 1.0 ppm). $[\alpha]_D^{20}$ = +0.4 (*c* = 0.5).

tert-butyl (*R*)-4-(4-nitrobenzamido)-4-(1,4-dioxaspiro[4.5]decan-8-yl)pentanoate

tert-butyl (*R*)-4-amino-4-(1,4-dioxaspiro[4.5]decan-8-yl)pentanoate was derivatised using general procedure B for the purpose of enantiomeric ratio determination. This gave the title compound, whose enantiomers were more readily separable by chiral HPLC. **^{1}H NMR** (400 MHz, $CDCl_3$) δ: 8.24 (d, *J* = 8.7 Hz, 2H), 7.95 (d, *J* = 8.7 Hz, 2H), 3.92 (app. t, *J* = 3.01 Hz, 4H), 2.40 (t, *J* = 7.0 Hz, 3H), 2.25 (dt, *J* = 7.0, 14.8 Hz, 1H), 1.83 – 1.67 (m, 6H), 1.64 – 1.49 (m, 3H), 1.45 (s, 3H), 1.41 (s, 9H). **HRMS** m/z calculated for $C_{24}H_{34}N_2O_7$ [M+H]$^+$ 463.2439, found 463.2438 (Δ = 0.2 ppm), 407.1778 [M−C_4H_8]$^+$. **Chiral HPLC** Chiralpak AD-H (Hexane/iPrOH = 85/15, 1.0 mL min^{-1}, 30 °C) t_R = 15.6 (major), 22.6 (minor) minutes.

***tert*-butyl (*R*)-4-amino-4-(2,3-dihydro-1*H*-inden-2-yl)pentanoate, 3j**

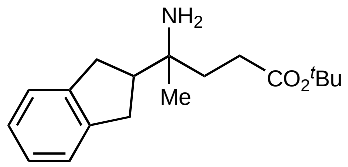

Prepared according to general procedure A using 1-(2,3-dihydro-1H-inden-2-yl)ethan-1-one (64 mg, 0.40 mmol, 2.0 equiv) as the ketone and *tert*-butyl acrylate (44 µL, 0.30 mmol, 1.5 equiv) as the acceptor, and Ir(dMeppy)$_3$ (1.5 mg) as the photocatalyst. Purification by acid/base extraction afforded the title compound as pale yellow oil (48% yield by ^{1}H qNMR; >93% purity). Further purification by automated flash column chromatography (RediSep Rf Gold 4.0 g, 1–20% [20% {1.75 M NH_3 in MeOH} in DCM] DCM) gave the title compound as a pale yellow oil (21.1 mg, 37%, e.r. 83:17). **^{1}H NMR** (400 MHz, $CDCl_3$) δ: 7.21 – 7.16 (m, 2H), 7.15 – 7.10 (m, 2H), 2.97 – 2.79 (m, 4H), 2.53 (qt, *J* = 9.4 Hz, 1H), 2.34 (qtd, *J* = 7.0, 10.2 Hz, 2H), 1.74 (qtd, *J* = 2.1, 8.1 Hz, 2H), 1.46 (s, 9H), 1.13 (br. s, 2H), 1.06 (s, 3H). **^{13}C NMR** (101 MHz, $CDCl_3$) δ: 173.7, 143.1, 143.0, 126.3, 124.6, 124.5, 80.3, 52.7, 50.6, 36.9, 34.1, 33.7, 30.7, 28.2, 25.2. **IR** ν_{max}/cm^{-1}: 2974, 2931, 1723, 1484, 1458, 1391, 1366, 1301, 1255, 1146. **HRMS** m/z calculated for $C_{18}H_{27}NO_2$ [M+H]$^+$ 290.2115, found 290.2122 (Δ = 2.4 ppm). $[\alpha]_D^{20}$ = −3.0 (*c* = 0.5).

***tert*-butyl (*R*)-4-(2,3-dihydro-1*H*-inden-2-yl)-4-(4-nitrobenzamido)pentanoate**

tert-butyl (*R*)-4-amino-4-(2,3-dihydro-1*H*-inden-2-yl)pentanoate was derivatised using general procedure B for the purpose of enantiomeric ratio determination. This gave the title compound, whose enantiomers were more readily separable by chiral HPLC. **^{1}H NMR** (400 MHz, $CDCl_3$) δ: 8.25 (d, *J* = 8.8 Hz, 2H), 7.88 (d, *J* = 8.8 Hz, 2H), 7.22 – 7.12 (m, 4H), 6.93 (br. s, 1H), 3.43 (qt, *J* = 8.5 Hz, 1H), 3.08 – 2.09 (m, 4H), 2.47 – 2.40 (m, 2H), 2.37 – 2.30 (m, 1H), 1.82 (dt, *J* = 6.3, 14.5 Hz, 1H), 1.55 (s, 3H), 1.43 (s, 9H). **HRMS** m/z calculated for $C_{25}H_{30}N_2O_5$ $[M+H]^+$ 439.2228, found 439.2226 (Δ = 0.4 ppm), 383.1605 $[M-C_4H_8]^+$. **Chiral HPLC** Chiralpak AD-H (Hexane/iPrOH = 85/15, 1.0 mL min^{-1}, 30 °C) t_R = 13.1 (major), 16.5 (minor) minutes.

Alkene Scope

4-(Phenylsulfonyl)-2-(tetrahydro-2H-pyran-4-yl)butan-2-amine, 3k

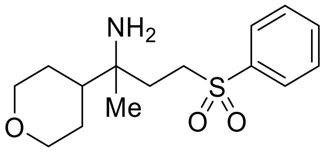

Prepared according to general procedure A using 1-tetrahydro-2H-pyran-4-ylethanone (50 µL, 0.40 mmol, 2.0 equiv) as the ketone, phenyl vinyl sulfone (50 mg, 0.30 mmol, 1.5 equiv) as the acceptor and Ir(dMeppy)$_3$ (1.5 mg) as the photocatalyst. Purification by automated flash column chromatography (RediSep Rf Gold 4.0 g, 1–20% [20% {1.75 M NH_3 in MeOH} in DCM] DCM) gave the title compound as crystalline white solid (38.6 mg, 65%, 0.13 mmol, e.r. 81:19). **^{1}H NMR** (500 MHz, $CDCl_3$) δ: 7.93 – 7.88 (m, 2H), 7.68 – 7.62 (m, 1H), 7.60 – 7.53 (m, 2H), 4.06 – 3.91 (m, 2H), 3.38 – 3.20 (m, 3H H_{11}), 3.20 – 3.09 (m, 1H), 1.75 (dd, *J* = 9.5, 7.3 Hz, 2H), 1.63 – 1.42 (m, 2H), 1.42 – 1.29 (m, 3H), 0.94 (s, 3H). **^{13}C NMR** (126 MHz, $CDCl_3$) δ: 139.3, 133.8, 129.4, 128.1, 68.3, 68.2, 53.0, 52.0, 45.7, 31.8, 27.3, 26.8, 24.0. **IR** ν_{max}/cm^{-1}: 2952, 2842, 1446, 1279, 1138, 1083, 1026. **HRMS** m/z (ASAP) calculated for $C_{15}H_{24}NO_3S$ $[M+H]^+$ 298.1477, found 298.1477 (Δ = 0.0 ppm). **mp** 125 °C. $[\alpha]_D^{20}$ = -0.60.

1.2 mmol Scale Reaction

A large oven-dried microwave vial was charged with a magnetic stirrer bar and activated 4 Å MS (1.2 g, 100 mg/0.1 mmol amine). The vial was sealed and evacuate-refilled with N_2 (3 cycles). DCM (6 mL), (R)-2-methoxy-1-phenylethan-1-amine (180 µL, 1.2 mmol, 1.0 equiv), 1-tetrahydro-2H-pyran-4-ylethanone (300 µL, 2.4 mmol, 2.0 equiv) and tris(2,2,2-trifluoroethyl) borate (52 µL, 0.24 mmol, 20 mol%) were added sequentially and the reaction mixture heated at 80 °C for 24 h. The reaction mixture was allowed to cool to room temperature and concentrated under vacuum to remove tris(2,2,2-trifluoroethyl) borate prior to irradiation. Ir(dMeppy)$_3$ (9 mg, 1.0 mol%) and phenyl vinyl sulfone (300 mg, 1.8 mmol, 1.5 equiv) were added. The vial was then resealed and evacuate-refilled with N_2 (3 cycles). DCM (12 ml) was added. The reaction mixture was irradiated for 24 hrs with vigorous stirring. Following irradiation, the reaction mixture was filtered through Celite® and concentrated under vacuum to afford the crude α-tertiary imine. A flask then was charged with the crude imine residue is dissolved in DCM (6 mL), TFA (90 µL, 1.2 mmol, 1 equiv) and a

magnetic stirrer bar. The mixture was stirred at room temperature for 4 h. The crude mixture was diluted with PE (60:40) (60 ml), added to 1M HCl (60 ml) and the flask washed out with PE (60:40): Et_2O 1:1 (18 ml). The aqueous phase was collected and the organic layer washed with water (60 ml). The combined aqueous layers were washed with PE (60:40): Et_2O 9:1 (60 ml) and then basified with 10% NaOH (12 ml). The product was then extracted into DCM (2 x 60 ml), dried over $NaSO_4$, filtered and concentrated under vacuum to afford the crude α-tertiary primary amine. Purification by automated flash column chromatography (RediSep Rf Silver 40.0 g, 1–20% [20% {1.75 M NH_3 in MeOH} in DCM] DCM) gave the title compound as crystalline white solid (178 mg, 50%, 0.60 mmol, e.r. 81:19).

4-nitro-*N*-(4-(phenylsulfonyl)-2-(tetrahydro-*2H*-pyran-4-yl)butan-2-yl)benzamide

4-(Phenylsulfonyl)-2-(tetrahydro-2H-pyran-4-yl)butan-2-amine was derivatised using general procedure B for the purpose of enantiomeric ratio determination. This gave the title compound, whose enantiomers were more readily separable by chiral HPLC. **^{1}H NMR** (700 MHz, $CDCl_3$) δ: 8.29 – 8.25 (m, 2H), 7.92 – 7.85 (m, 4H), 7.72 – 7.67 (m, 1H), 7.59 (t, J = 7.9 Hz, 2H), 4.06 – 3.95 (m, 2H), 3.42 – 3.32 (m, 2H), 3.28 – 3.13 (m, 2H), 2.60 – 2.46 (m, 2H), 2.04 – 1.99 (m, 1H), 1.56 – 1.39 (m, 4H), 1.34 (s, 3H). **HRMS** m/z (ESI) calculated for $C_{22}H_{27}N_2O_6S$ $[M+H]^+$ 447.1590, found 447.1590 (Δ = 0.0 ppm). **Chiral HPLC** Chiralpak AD-H (Hexane/iPrOH = 70/30, 1.0 mL min^{-1}, 30 °C) t_R = 10.7 (minor), 12.4 (major) minutes.

Diethyl (3-amino-3-(tetrahydro-*2H*-pyran-4-yl)butyl)phosphonate, 3l

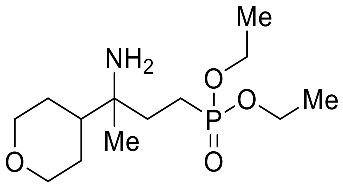

Prepared according to general procedure A using 1-tetrahydro-2H-pyran-4-ylethanone (50 µL, 0.40 mmol, 2.0 equiv) as the ketone, diethyl vinyl phosphonate (46 µL, 0.30 mmol, 1.5 equiv) as the acceptor and $Ir(dMeppy)_3$ (1.5 mg) as the photocatalyst. Purification by automated flash column chromatography (RediSep Rf Gold 4.0 g, 1–25% [20% {1.75 M NH_3 in MeOH} in DCM] DCM) gave the title compound as an off-white solid (43.0 mg, 73%, 0.15 mmol, e.r. 17:83). **^{1}H NMR** (700 MHz, $CDCl_3$) δ: 4.12 – 4.03 (m, 4H), 4.03 – 3.97 (m, 2H), 3.37 – 3.28 (m, 2H), 1.85 – 1.67 (m, 2H), 1.65 – 1.45 (m, 4H H_7 H_8), 1.45 – 1.37 (m, 3H), 1.29 (t, J = 7.1 Hz, 6H), 0.96 (s, 3H). **^{13}C NMR** (126 MHz, $CDCl_3$) δ: 68.45, 68.36, 61.7 (d, J = 6.6 Hz), 53.3 (d, J = 16.7 Hz), 44.7, 32.3 (d, J = 4.4 Hz), 27.4, 26.9, 24.0, 20.2 (d, J = 141.9 Hz), 16.6 (d, J = 6.0 Hz). **^{31}P NMR** (203 MHz, $CDCl_3$) δ: 34.05. **IR** ν_{max} /cm^{-1}: 2947, 2844, 1389, 1240, 1055, 1008, 954. **HRMS** m/z (ESI) calculated for $C_{13}H_{28}NO_4P$ $[M+H]^+$ 294.1829, found 294.1832 (Δ = 1.2 ppm). $[\alpha]_D^{20}$ = -1.00

Diethyl (3-(4-nitrobenzamido)-3-(tetrahydro-*2H*-pyran-4-yl)butyl)phosphonate

Diethyl (3-amino-3-(tetrahydro-2H-pyran-4-yl)butyl)phosphonate was derivatised using general procedure B for the purpose of enantiomeric ratio determination. This gave the title compound, whose enantiomers were more readily separable by chiral HPLC. **^{1}H NMR** (700 MHz, $CDCl_3$) δ: 8.27 (d, J = 8.7 Hz, 2H), 7.99 (d, J = 8.7 Hz, 2H), 4.16 – 4.05 (m, 4H), 4.04 – 4.00 (m, 2H), 3.46 – 3.34 (m, 2H), 1.94 – 1.70 (m, 2H), 1.61 – 1.43 (m, 7H), 1.40 (s, 3H), 1.36 – 1.29 (m, 6H)z.

HRMS m/z (ESI) calculated for $C_{20}H_{31}N_2O_7NaP$ $[M+H]^+$ 465.1761, found 465.1755 (Δ = -1.4 ppm). **Chiral HPLC** Chiralpak AD-H (Hexane/iPrOH = 93/07, 1.0 mL min^{-1}, 30 °C) t_R = 44.4 (minor), 47.8 (major) minutes.

4-Amino-4-(tetrahydro-2H-pyran-4-yl)pentanenitrile, 3m

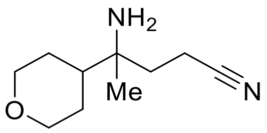

Prepared according to general procedure A using 1-tetrahydro-2H-pyran-4-ylethanone (50 µL, 0.40 mmol, 2.0 equiv) as the ketone, acrylonitrile (20 µL, 0.30 mmol, 1.5 equiv) as the acceptor and $Ir(dMeppy)_3$ (1.5 mg) as the photocatalyst. Purification by automated flash column chromatography (RediSep Rf Gold 4.0 g, 1–20% [20% {1.75 M NH_3 in MeOH} in DCM] DCM) gave the title compound as a pale yellow oil (27.0 mg, 74%, 0.15 mmol, e.r. 80:20). **^{1}H NMR** (700 MHz, $CDCl_3$) δ: 4.10 – 3.95 (m, 2H), 3.46 – 3.30 (m, 2H), 2.59 – 2.45 (m, 1H), 2.46 – 2.36 (m, 1H), 1.80 – 1.69 (m, 2H), 1.62 – 1.52 (m, 2H), 1.46 – 1.38 (m, 3H), 1.02 (s, 3H). **^{13}C NMR** (101 MHz, $CDCl_3$) δ: 120.7, 68.34, 68.26, 53.0, 45.9, 35.4, 27.5, 26.9, 23.7, 12.0. **IR** ν_{max} /cm^{-1}: 2946, 2846, 2244, 1667, 1445, 1243, 1123, 1088. **HRMS** m/z (ASAP) calculated for $C_{10}H_{19}N_2O$ $[M+H]^+$ 183.1497, found 183.1498 (Δ = 0.5 ppm). $[\alpha]_D^{20}$ = +0.20

***N*-(4-cyano-2-(tetrahydro-*2H*-pyran-4-yl)butan-2-yl)-4-nitrobenzamide**

4-Amino-4-(tetrahydro-2H-pyran-4-yl)pentanenitrile was derivatised using general procedure B for the purpose of enantiomeric ratio determination. This gave the title compound, whose enantiomers were more readily separable by chiral HPLC. **^{1}H NMR** (700 MHz, $CDCl_3$) δ: 8.30 (d, J = 8.8 Hz, 2H), 7.90 (d, J = 8.7 Hz, 2H), 4.09 – 4.01 (m, 2H), 3.45 – 3.37 (m, 2H), 2.82 – 2.73 (m, 1H), 2.60 – 2.52 (m, 1H), 1.53 – 1.44 (m, 7H), 1.33 (s, 3H). **HRMS** m/z (ESI) calculated for $C_{17}H_{22}N_3O_4$ $[M+H]^+$ 332.1610, found 226.1612 (Δ = 0.6 ppm). **Chiral HPLC** Chiralpak IA (Hexane/iPrOH = 90/10, 1.0 mL min^{-1}, 30 °C) t_R = 37.3 (major), 45.0 (minor) minutes.

***tert*-Butyl 4-amino-4-(tetrahydro-2H-pyran-4-yl)pentanoate, 3n**

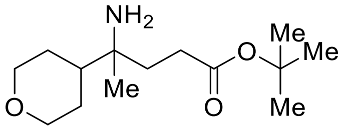

Prepared according to general procedure A using 1-tetrahydro-2H-pyran-4-ylethanone (50 µL, 0.40 mmol, 2.0 equiv) as the ketone, *tert*-butyl acrylate (44 µL, 0.30 mmol, 1.5 equiv) as the acceptor and $Ir(dMeppy)_3$ (1.5 mg) as the photocatalyst. Purification by automated flash column chromatography (RediSep Rf Gold 4.0 g, 1–20% [20% {1.75 M NH_3 in MeOH} in DCM] DCM) gave the title compound as an off-white solid (34.0 mg, 65%, 0.15 mmol, e.r. 85:15). **^{1}H NMR** (500 MHz, $CDCl_3$) δ: 4.08 – 3.95 (m, 2H), 3.43 – 3.27 (m, 2H), 2.37 – 2.18 (m, 2H), 1.70 (t, J = 8.1 Hz, 2H), 1.66 – 1.50 (m, 2H), 1.49 – 1.44 (m, 3H), 1.43 (s, 9H), 1.03 (s, 3H). **^{13}C NMR** (126 MHz, $CDCl_3$) δ: 173.3, 80.5, 68.5, 68.4, 57.1, 45.0, 34.4, 30.2, 28.2, 27.4, 27.1, 24.1. **IR** ν_{max} /cm^{-1}: 2950, 2849, 1727, 1456, 1367, 1246, 1154. **HRMS** m/z (ESI) calculated for $C_{14}H_{27}NO_3$ $[M+H]^+$ 258.2064, found 258.2053 (Δ = -4.1 ppm). $[\alpha]_D^{20}$ = -0.20

tert-butyl 4-(4-nitrobenzamido)-4-(tetrahydro-2H-pyran-4-yl)pentanoate

tert-Butyl 4-amino-4-(tetrahydro-2H-pyran-4-yl)pentanoate was derivatised using general procedure B for the purpose of enantiomeric ratio determination. This gave the title compound, whose enantiomers were more readily separable by chiral HPLC. **^{1}H NMR** (700 MHz, $CDCl_3$) δ: 8.28 (d, J = 8.8 Hz, 2H), 7.98 (d, J = 8.8 Hz, 2H), 4.07 – 3.97 (m, 2H), 3.52 – 3.37 (m, 2H), 2.51 – 2.41 (m, 2H), 1.78 – 1.70 (m, 2H), 1.59 – 1.50 (m, 5H), 1.48 (s, 3H), 1.46 (s, 9H).**HRMS** m/z (ESI) calculated for $C_{21}H_{31}N_2O_6$ $[M+H]^+$ 407.2182, found 407.2185 (Δ = 0.7 ppm). **Chiral HPLC** Chiralpak AD-H (Hexane/iPrOH = 85/15, 1.0 mL min^{-1}, 30 °C) t_R = 10.1 (major), 14.0 (minor) minutes.

5,5,6,6,7,7,8,8,9,9,10,10,11,11,12,12,12-heptadecafluoro-2-(tetrahydro-2H-pyran-4-yl)dodecan-2-amine, 3o

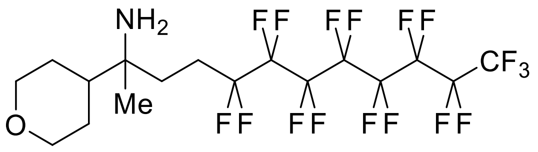

Prepared according to general procedure A using 1-tetrahydro-2H-pyran-4-ylethanone (50 µL, 0.40 mmol, 2.0 equiv) as the ketone, (perfluorooctyl)ethylene (80 µL, 0.30 mmol, 1.5 equiv) as the acceptor and $Ir(dMeppy)_3$ (1.5 mg) as the photocatalyst. Purification by automated flash column chromatography (RediSep Rf Gold 4.0 g, 1–20% [20% {1.75 M NH_3 in MeOH} in DCM] DCM) gave the title compound as a mixture of diastereomers as a pale yellow oil (38 mg, 33%, 0.07 mmol, e.r. 86:14). **^{1}H NMR** (400 MHz, $CDCl_3$) δ: 4.14 – 3.97 (m, 2H), 3.37 (tt, J = 11.4, 2.4 Hz, 2H), 2.35 – 2.01 (m, 2H), 1.63 – 1.52 (m, 4H), 1.51 – 1.38 (m, 3H), 1.01 (s, 3H), 0.92 (br s, 2H). **^{13}C NMR** (101 MHz, $CDCl_3$) δ: 68.5, 68.4, 52.4, 45.9, 29.9, 27.5, 27.0, 25.6 – 26.0 (m), 24.4. **^{19}F NMR** (376 MHz, $CDCl_3$) δ: -81.7 (t, J = 10.0 Hz), -115.2 – -115.5 (m), -122.6 (dt, J = 26.3, 13.4 Hz), -122.9 (tt, J = 17.7, 7.9 Hz), -123.5 – -123.8 (m), -124.1 (d, J = 15.3 Hz), -126.9 – -127.2 (m). **IR** ν_{max}/cm^{-1}: 2951, 2847, 1237, 1201, 1147, 1133, 1113, 1092. **HRMS** m/z m/z (ESI) calculated for $C_{17}H_{18}NOF_{17}$ $[M+H]^+$ 576.1190, found 576.1194 (Δ = 0.7 ppm).$[\alpha]_D^{20}$ = -2.2

N-(5,5,6,6,7,7,8,8,9,9,10,10,11,11,12,12,12-heptadecafluoro-2-(tetrahydro-2H-pyran-4-yl)dodecan-2-yl)-4-nitrobenzamide

5,5,6,6,7,7,8,8,9,9,10,10,11,11,12,12,12-heptadecafluoro-2-(tetrahydro-*2H*-pyran-4-yl)dodecan-2-amine was derivatised using general procedure B for the purpose of enantiomeric ratio determination. This gave the title compound, whose enantiomers were more readily separable by chiral HPLC. **^{1}H NMR** (400 MHz, $CDCl_3$) δ: 8.38 – 8.28 (m, 2H), 7.96 – 7.81 (m, 2H), 5.72 (s, 1H), 4.13 – 4.00 (m, 2H), 3.44 (t, *J* = 10.9, 2.6 Hz, 2H), 2.75 – 2.63 (m, 1H), 2.63 – 2.50 (m, 1H), 2.23 – 2.06 (m, 2H), 2.01 – 1.85 (m, 1H), 1.69 – 1.34 (m, 4H), 1.31 (s, 3H). **HRMS** m/z (ESI) calculated for $C_{24}H_{21}N_2O_4F_{17}$ $[M+H]^+$ 725.1303, found 725.1304 (Δ = 0.2 ppm). **Chiral SFC** YMC CHIRAL ART SB column (97% CO_2, 3% MeOH, 1.25 mL min^{-1}, 40 °C)**,** t_R = 14.36 (major), 16.06 (minor) minutes.

3-amino-N-methyl-N-(3-phenyl-3-(4-(trifluoromethyl)phenoxy)propyl)-3-(tetrahydro-2H-pyran-4-yl)butane-1-sulfonamide, 3p

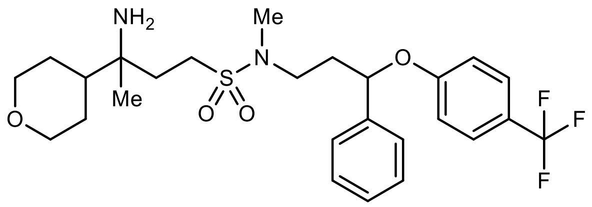

Prepared according to general procedure A using 1-tetrahydro-2H-pyran-4-ylethanone (50 µL, 0.40 mmol, 2.0 equiv) as the ketone, *N*-methyl-*N*-(3-phenyl-3-(4-(trifluoromethyl)phenoxy)propyl)ethenesulfonamide (120 mg, 0.30 mmol, 1.5 equiv) as the acceptor and Ir(dMeppy)$_3$ (1.5 mg) as the photocatalyst. Purification by automated flash column chromatography (RediSep Rf Gold 4.0 g, 0–5% [20% {1.75 M NH_3 in MeOH} in DCM] DCM) gave the title compound as a colourless oil (46.2 mg, 44%, 0.09 mmol, d.r. 1:1, 22:78, 20:80). **^{1}H NMR** (400 MHz, $CDCl_3$) δ: 7.42 (d, *J* = 8.7 Hz, 2H), 7.34 (d, *J* = 4.4 Hz, 4H H_9), 7.28 (dd, *J* = 4.8, 3.7 Hz, 1H), 6.90 (d, *J* = 8.6 Hz, 2H), 5.34 – 5.23 (m, 1H), 4.10 – 3.99 (m, 2H), 3.55 – 3.40 (m, 1H), 3.38 – 3.26 (m, 3H), 3.11 (m, 1H), 3.05 – 2.89 (m, 1H), 2.89 (s, 3H), 2.34 – 2.19 (m, 1H), 2.19 – 2.07 (m, 1H), 1.84 – 1.73 (m, 2H), 1.62 – 1.50 (m, 2H), 1.49 – 1.33 (m, 3H), 1.11 (br s, 2H), 0.96 (overlapping s, 3H).**^{13}C NMR** (101 MHz, $CDCl_3$) δ: 160.3 (d, *J* = 1.3 Hz), 140.5, 129.0, 128.2, 126.9 (q, *J* = 3.8 Hz), 125.9, 124.5 (d, *J* = 249.7 Hz), 123.0, 115.9 77.4, 68.3, 52.8, 47.1, 46.3, 45.1, 37.7, 35.2, 32.6, 27.5, 26.9, 24.1.**^{19}F NMR** (376 MHz, $CDCl_3$) δ -61.6. **IR** *v*max /cm^{-1}: 2948, 2650, 1325, 1250, 1152, 1111, 1068. **HRMS** m/z (ESI) calculated for $C_{26}H_{36}N_2O_4F_3S$ $[M+H]^+$ 529.2342, found 529.2345 (Δ = 0.5 ppm). $[\alpha]_D^{20}$ = -0.2.

N-(4-(N-methyl-N-(3-phenyl-3-(4-(trifluoromethyl)phenoxy)propyl)sulfamoyl)-2-(tetrahydro-2H-pyran-4-yl)butan-2-yl)-4-nitrobenzamide

3-amino-N-methyl-N-(3-phenyl-3-(4-(trifluoromethyl)phenoxy)propyl)-3-(tetrahydro-2H-pyran-4-yl)butane-1-sulfonamide was derivatised using general procedure B for the purpose of enantiomeric ratio determination. This gave the title compound, whose enantiomers were more readily separable by chiral HPLC. **^{1}H NMR** (400 MHz, $CDCl_3$) δ 8.25 (d, 2H), 7.86 (d, *J* = 8.8, 2.4 Hz, 2H), 7.42 (d, *J* = 8.5 Hz, 2H), 7.33 (d, *J* = 4.3 Hz, 4H), 7.28 (t, *J* = 4.2 Hz, 1H), 6.88 (dd, *J* = 8.8, 3.9 Hz, 2H), 6.11 – 5.98 (m, 1H), 5.27 (dt, *J* = 8.8, 3.6 Hz, 1H), 4.07 – 3.98 (m, 2H), 3.56 – 3.30 (m, 4H), 3.04 (t, *J* = 7.8 Hz, 2H), 2.93 – 2.86 (m, 3H), 2.66 (dq, *J* = 16.2, 8.3 Hz, 1H), 2.50 (s, 1H), 2.31 – 2.20 (m, 1H), 2.18 – 2.06 (m, 3H), 1.56– 1.46 (m, 3H), 1.33 – 1.31 (m, 3H). **HRMS** m/z (ASAP-) calculated for $C_{33}H_{38}N_3O_7F_3S$ $[M]^-$ 677.2383, found 677.2394 (Δ = 1.6 ppm). **Chiral HPLC** Chiralpak IA (Hexane/iPrOH = 85/15, 0.5 mL min^{-1}, 38 °C) t_R = 37.7 (minor), 43.7 (major) minutes, t_R = 39.8 (minor), 45.8 (major) minutes.

4-(morpholinosulfonyl)-2-(tetrahydro-2H-pyran-4-yl)butan-2-amine, 3q

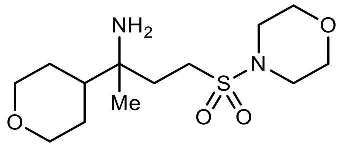

Prepared according to general procedure A using 1-tetrahydro-2H-pyran-4-ylethanone (50 µL, 0.40 mmol, 2.0 equiv) as the ketone, 4-(vinylsulfonyl)morpholine (53 mg, 0.30 mmol, 1.5 equiv) as the acceptor and Ir(dMeppy)$_3$ (1.5 mg) as the photocatalyst. Purification by automated flash column chromatography (RediSep Rf Gold 4.0 g, 1–10% [20% {1.75 M NH_3 in MeOH} in DCM] DCM) gave the title compound as a colourless oil(28.2 mg, 46%, 0.09 mmol, e.r. 82:18). **^{1}H NMR** (500 MHz, $CDCl_3$) δ: 4.09 – 4.00 (m, 2H), 3.78 – 3.72 (m, 4H), 3.39 – 3.31 (m, 2H), 3.31 – 3.24 (m, 4H), 3.17 – 3.09 (m, 1H), 3.01 – 2.92 (m, 1H), 1.82 (dd, *J* = 9.4, 7.3 Hz, 2H), 1.58 (t, *J* = 16.0, 12.1, 2.4 Hz, 2H), 1.48 – 1.33 (m, 3H), 0.99 (s, 3H).**^{13}C NMR** (126 MHz, $CDCl_3$) δ: 68.4, 68.3, 66.7, 52.8, 46.3, 46.0, 44.4, 32.4, 27.5, 26.9, 24.1. **IR** ν_{max}/cm^{-1}: 2920, 2851, 1452, 1323, 1260, 1151, 1112, 1091, 1075. **HRMS** m/z (ESI) calculated for $C_{13}H_{27}N_2O_4S$ [M+H]$^+$ 307.1686, found 307.1689 (Δ = 0.8 ppm). $[\alpha]_D^{20}$ = +1.6.

N-(4-(morpholinosulfonyl)-2-(tetrahydro-2H-pyran-4-yl)butan-2-yl)-4-nitrobenzamide

4-(morpholinosulfonyl)-2-(tetrahydro-2H-pyran-4-yl)butan-2-amine was derivatised using general procedure B for the purpose of enantiomeric ratio determination. This gave the title compound, whose enantiomers were more readily separable by chiral HPLC. **^{1}H NMR** (400 MHz, $CDCl_3$) δ 8.41 – 8.23 (m, 2H), 7.99 – 7.85 (m, 2H), 6.03 (s, 1H), 4.08 – 3.94 (m, 2H), 3.75 (t, J = 4.6 Hz, 4H), 3.40 (t, J = 11.4, 7.5, 2.1 Hz, 2H), 3.28 (q, J = 4.1 Hz, 4H), 3.08 – 2.95 (m, 2H), 2.72 (ddd, J = 15.3, 9.2, 6.4 Hz, 1H), 2.60 – 2.43 (m, 1H), 2.16 – 2.05 (m, 1H), 1.55 – 1.43 (m, 4H), 1.34 (s, 3H). **HRMS** m/z (ESI) calculated for $C_{20}H_{30}N_3O_7S$ [M+H]$^+$ 456.1799, found 456.1797 (Δ = -0.4 ppm). **Chiral HPLC** Chiralpak IA (Hexane/iPrOH = 70/30, 1.0 mL min^{-1}, 30 °C) t_R = 10.7 (major), 13.4 (minor) minutes.

4-((4-(6-fluorobenzo[d]isoxazol-3-yl)piperidin-1-yl)sulfonyl)-2-(tetrahydro-2H-pyran-4-yl)butan-2-amine, 3r

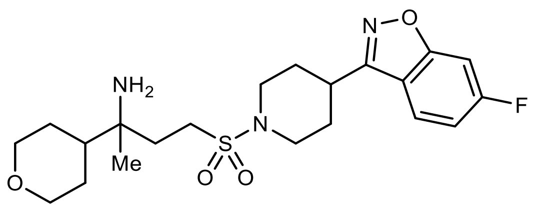

Prepared according to general procedure A using 1-tetrahydro-2H-pyran-4-ylethanone (50 µL, 0.40 mmol, 2.0 equiv) as the ketone, 6-fluoro-3-(1-(vinylsulfonyl)piperidin-4-yl)benzo[d]isoxazole (93 mg, 0.30 mmol, 1.5 equiv) as the acceptor and Ir(dMeppy)$_3$ (1.5 mg) as the photocatalyst. Purification by automated flash column chromatography (RediSep Rf Gold 4.0 g, 1–10% [20% {1.75 M NH_3 in MeOH} in DCM] DCM) gave the title compound as a colourless oil (17.5 mg, 20%, 0.04 mmol, e.r. 82:18). **^{1}H NMR** 400 MHz, $CDCl_3$) δ: 7.65 (dd, *J* = 8.7, 5.0 Hz, 1H), 7.26 (dd, *J* = 8.5, 2.1 Hz, 1H), 7.08 (td, *J* = 8.8, 2.1 Hz, 1H), 4.14 – 3.96 (m, 2H), 3.89 (dt, *J* = 12.6, 4.1 Hz, 2H), 3.36 (t, *J* = 11.3, 8.8, 2.5 Hz, 2H), 3.25 (dq, *J* = 9.8, 5.0, 4.5 Hz, 1H), 3.21 – 2.95 (m, 4H), 2.27 – 2.03 (m, 4H), 1.93 – 1.77 (m, 2H), 1.59 (t, *J* = 12.0, 9.3, 2.5 Hz, 2H), 1.42 (tt, *J* = 9.5, 5.5 Hz, 3H), 1.01 (s, 3H). **^{13}C NMR** (101 MHz, $CDCl_3$) δ: 164.3 (d, J = 251.2 Hz), 164.1 (d, J =

13.5 Hz), 160.1, 122.4 (d, J = 11.2 Hz), 117.1 (d, J = 1.6 Hz), 112.9 (d, J = 25.5 Hz), 97.8 (d, J = 26.8 Hz), 68.4, 68.3, 52.8, 46.3, 45.6 45.6, 45.4, 33.7, 32.7, 30.2, 27.5, 27.0, 24.2. **^{19}F NMR** (376 MHz, $CDCl_3$) δ: -109.9. **IR** *v*max /cm^{-1}: 2948, 2848, 1614, 1323, 1271, 1146, 1123, 1091. **HRMS** m/z (ESI) calculated for $C_{21}H_{31}N_3O_4FS$ $[M+H]^+$ 440.2014, found 440.2016 (Δ = 0.4 ppm). $[\alpha]_D^{20}$ = +2.0.

N-(4-((4-(6-fluorobenzo[d]isoxazol-3-yl)piperidin-1-yl)sulfonyl)-2-(tetrahydro-2H-pyran-4-yl)butan-2-yl)-4-nitrobenzamide

4-((4-(6-fluorobenzo[d]isoxazol-3-yl)piperidin-1-yl)sulfonyl)-2-(tetrahydro-2H-pyran-4-yl)butan-2-aminewas derivatised using general procedure B for the purpose of enantiomeric ratio determination. This gave the title compound, whose enantiomers were more readily separable by chiral HPLC. **^{1}H NMR** (500 MHz, $CDCl_3$) δ: 8.3 – 8.3 (m, 2H), 8.0 – 7.9 (m, 2H), 7.7 (dd, *J* = 8.7, 5.0 Hz, 1H), 7.3 – 7.2 (m, 1H), 7.1 (td, *J* = 8.8, 2.1 Hz, 1H), 6.1 (s, 1H), 4.1 – 4.0 (m, 2H), 3.9 – 3.7 (m, 2H), 3.5 – 3.4 (m, 2H), 3.3 – 3.2 (m, 1H), 3.2 – 3.1 (m, 1H), 3.1 – 3.0 (m, 3H), 2.8 – 2.7 (m, 1H), 2.6 – 2.5 (m, 1H), 2.2 – 2.1 (m, 4H), 1.6 (t, *J* = 7.0 Hz, 2H), 1.5 – 1.5 (m, 3H), 1.4 (s, 3H). **HRMS** m/z (ESI) calculated for $C_{28}H_{34}N_4O_7FS$ $[M+H]^+$ 589.2127, found 589.2116 (Δ = -1.9 ppm). **Chiral HPLC** Chiralpak AD-H (Hexane/iPrOH = 85/15, 1.0 mL min^{-1}, 30 °C) t_R = 50.9 (minor), 56.9 (major) minutes.

***tert*-Butyl 4-amino-2-methyl-4-(tetrahydro-2H-pyran-4-yl)pentanoate, 3s**

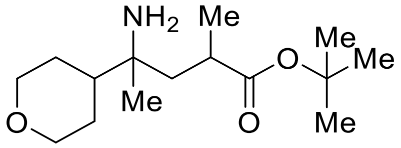

Prepared according to general procedure A using 1-tetrahydro-2H-pyran-4-ylethanone (50 µL, 0.40 mmol, 2.0 equiv) as the ketone, *tert*-butyl methacrylate (49 µL, 0.30 mmol, 1.5 equiv) as the acceptor and $Ir(dMeppy)_3$ (1.5 mg) as the photocatalyst. Purification by automated flash column chromatography (RediSep Rf Gold 4.0 g, 1–20% [20% {1.75 M NH_3 in MeOH} in DCM] DCM) gave the title compound as a mixture of diastereomers as a pale yellow oil (15.0 mg, 28%, 0.06 mmol, d.r. 4:5, major e.r. 91:9, minor e.r. 87:13). **^{1}H NMR** (500 MHz, $CDCl_3$) δ: 4.08 – 3.94 (m, 2H), 3.43 – 3.27 (m, 2H), 2.58 – 2.42 (m, 1H), 2.03 – 1.86 (m, 1H), 1.67 – 1.50 (m, 2H), 1.42 – 1.33 (m, 3H), 1.21 (dt, J = 14.4, 2.8 Hz, 1H), 1.16 – 1.09 (m, 3H), 0.97 – 0.94 (m, 3H). **^{13}C NMR** (126 MHz, $CDCl_3$) δ: 177.4, 177.2, 80.1, 80.0, 68.6, 68.55, 68.54, 53.3, 53.2, 46.7, 45.8, 43.8, 43.5, 36.5, 36.3, 28.2, 28.1, 27.7, 27.5, 27.2, 27.1, 24.8, 24.4, 20.6, 20.3. **IR** v_{max}/cm^{-1}: 2967, 2841, 1723, 1466, 1366, 1144. **HRMS** m/z (ASAP) calculated for $C_{15}H_{30}NO_3$ $[M+H]^+$ 272.2226, found 272.2223 (Δ = -1.1 ppm). $[\alpha]_D^{20}$ = -2.6.

***tert*-butyl 2-methyl-4-(4-nitrobenzamido)-4-(tetrahydro-*2H*-pyran-4-yl)pentanoate**

tert-Butyl 4-amino-2-methyl-4-(tetrahydro-2H-pyran-4-yl)pentanoate was derivatised using general procedure B for the purpose of enantiomeric ratio determination. This gave the title compound, whose enantiomers were more readily separable by chiral HPLC. **^{1}H NMR** δ 8.34 – 8.19 (m, 2H), 8.05 – 7.87 (m, 2H), 4.11 – 3.94 (m, 2H), 3.49 – 3.30 (m, 2H), 2.76 – 2.45 (m, 1H), 2.22 – 2.12 (m, 1H), 1.62 – 1.49 (m, 4H), 1.43 – 1.36 (m, 9H), 1.26 – 1.23 (m, 5H), 1.20 (dd, *J* = 9.8, 6.6 Hz, 3H). **HRMS** m/z (ESI) calculated for $C_{22}H_{33}N_2O_6$ $[M+H]^+$ 421.2333, found 421.2332 (Δ = -0.3 ppm). **Chiral HPLC**

Chiralpak IA (Hexane/iPrOH = 90/10, 1.0 mL min^{-1}, 30 °C) major diastereomer t_R = 8.4 (major), 9.7 (minor) minutes, minor diastereomer t_R = 10.4 (major), 19.3 (minor) minutes.

5-methyl-5-(tetrahydro-2H-pyran-4-yl)pyrrolidin-2-one, 6

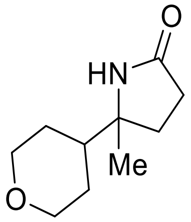

Prepared according to general procedure A using 1-tetrahydro-2H-pyran-4-ylethanone (50 µL, 0.40 mmol, 2.0 equiv) as the ketone, methylacrylate (27 µL, 0.30 mmol, 1.5 equiv) as the acceptor and Ir(dMeppy)$_3$ (1.5 mg) as the photocatalyst. The crude material was then refluxed in MeCN (5 mL) with trimethylamine (3 equiv.) in order to accelerate lactamisation. Purification by automated flash column chromatography (RediSep Rf Gold 4.0 g, 1–10% [20% {1.75 M NH_3 in MeOH} in DCM] DCM) gave the title compound as a white solid (9.7 mg, 27%, 0.06 mmol, e.r. 85:15). **^{1}H NMR** (500 MHz, $CDCl_3$) δ: 6.49 (s, 1H), 4.15 – 3.98 (m, 2H), 3.38 – 3.21 (m, 2H), 2.65 – 2.21 (m, 2H), 2.04 (ddd, *J* = 13.1, 9.8, 6.9 Hz, 1H), 1.78 (ddd, *J* = 13.1, 9.9, 6.0 Hz, 1H), 1.65 – 1.30 (m, 5H), 1.23 (s, 3H). **^{13}C NMR** (126 MHz, $CDCl_3$) δ: 177.4, 68.2, 68.0, 61.4, 45.5, 31.3, 30.5, 27.6, 27.5, 24.7. **IR** ν_{max}/cm^{-1}: 2940, 2849, 1689 (C=O), 1378, 1242, 1120, 1087, 1013. **HRMS** m/z (ESI) calculated for $C_{10}H_{18}NO_2$ [M+H]$^+$ 184.1332, found 184.1331 (Δ = -0.9 ppm). $[\alpha]_D^{20}$ = +6.4. **Chiral HPLC** Chiralpak AD-H (Hexane/iPrOH = 85/15, 1.0 mL min^{-1}, 30 °C) t_R = 8.3 (major), 9.8 (minor) minutes. **mp** 128 °C.

Product Derivatisation

2-methyl-2-(tetrahydro-2H-pyran-4-yl)pyrrolidine, 7

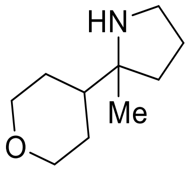

5-methyl-5-(tetrahydro-2H-pyran-4-yl)pyrrolidin-2-one (18 mg, 0.1 mmol) was dissolved in THF (2 ml) and cooled to 0°C under N_2. Lithium aluminium hydride in THF (0.125 mL, 2.4 M, 0.3 mmol) was added dropwise and the reaction mixture refluxed for 14 h. The mixture was cooled to 0°C, quenched by the dropwise addition of NaOH (10% aq.) and allowed to stir for a further 4 h. The mixture was diluted with water and the product extracted into EtOAc. The organic layer was separated, dried over $NaSO_4$ and concentrated under reduced pressure. Purification by automated flash column chromatography (RediSep Rf Gold 4.0 g, 0–10% [20% {1.75 M NH_3 in MeOH} in DCM] DCM) gave the title compound as a colourless oil (11.9 mg, 70%, 0.07 mmol, e.r. 85:15). **^{1}H NMR** (500 MHz, $CDCl_3$) δ: 4.08 – 3.93 (m, 2H), 3.45 – 3.30 (m, 2H), 3.14 – 3.05 (m, 1H), 3.05 – 2.97 (m, 1H), 1.93 – 1.79 (m, 2H), 1.72 – 1.42 (m, 7H), 1.09 (s, 3H). **^{13}C NMR** (126 MHz, $CDCl_3$) δ: 68.4, 65.1, 45.8, 45.6, 36.5, 28.8, 28.4, 25.1, 21.9. **IR** ν_{max}/cm^{-1}: 2923, 2850. **HRMS** m/z (ESI)

calculated for $C_{10}H_{20}NO$ $[M+H]^+$ 170.1539, found 170.1540 (Δ = 0.2 ppm). $[\alpha]_D^{20}$ = +5.5. **Chiral HPLC** Chiralpak IC (Hexane/iPrOH = 90/10, 1.0 mL min^{-1}, 30 °C) t_R = 14.5 (major), 20.7 (minor) minutes.

Setting the γ-amino centre

***tert*-butyl 3-(4-aminotetrahydro-2*H*-pyran-4-yl)-2-methylpropanoate, 8**

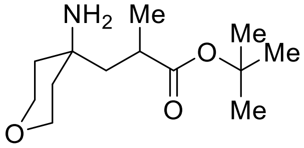

Prepared according to general procedure C using (S)-(-)-1-(1-Naphthyl)ethylamine (32 µL, 0.2 mmol) as the amine and *tert*-butyl methacrylate (48 µL, 0.30 mmol) as the acceptor. Purification by automated flash column chromatography (RediSep Rf 4.0 g, 0–10% [20% {1.75 M NH_3 in MeOH} in CH_2Cl_2] in CH_2Cl_2) gave the title compound as a pale yellow oil (19.7 mg, 0.08 mmol, 41%, 86% e.e.). **^{1}H NMR** (400 MHz, CDCl3) δ: 3.81 – 3.62 (m, 4H), 2.60 – 2.44 (m, 1H), 2.10 – 1.98 (m, 1H), 1.71 – 1.53 (m, 3H), 1.44 (s, 9H), 1.32 (dd, *J* = 14.4, 2.7 Hz, 2H), 1.16 (d, *J* = 7.1 Hz, 3H). **^{13}C NMR** (101 MHz, $CDCl_3$) δ: 177.1, 80.3, 64.0, 63.9, 49.2, 47.4, 38.5, 35.8, 28.1, 20.3. **IR** ν_{max}/cm^{-1}: 3172, 2965, 2858, 1721, 1675, 1453, 1382, 1273, 1156, 1102 **HRMS** m/z (ESI) calculated for $C_{13}H_{26}NO_3$ $[M+H]^+$ 244.1913, found 244.1922 (Δ = 3.7 ppm). $[\alpha]_D^{20}$ = -5.8. **Chiral HPLC** Chiralpak AS (Hexane/iPrOH = 95/05, 1.0 mL min^{-1}, 30 °C) t_R = 4.9 (major), 6.7 (minor) minutes.

Starting Material Synthesis

CAT Reagent Synthesis

(R)-2-methoxy-1-phenylethan-1-amine, 4a

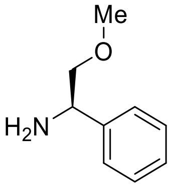

A flask was charged with Boc-D-phenylglycinol (10 g, 42 mmol), methyl *p*-toluenesulfonate (15.6 g, 84 mmol), tetrabutylammonium bromide (1.34 g, 4.1 mmol), PhMe (200 ml) and a magnetic stir bar. The solution was cooled to 0 °C and aqueous potassium hydroxide (8.5 ml, 20 M) was added. The mixture was stirred rapidly under N_2 for 4 hrs. The crude mixture was washed with water, aq HCl (3 M), sat. $NaHCO_3$, and brine, dried over $NaSO_4$ and concentrated under reduced pressure. The residue was dissolved in acetone (200 ml) and c. HCl was added (10 ml) and the mixture was refluxed overnight. After cooling to room temperature the reaction mixture was concentrated under reduced pressure, dissolved in water and washed with EtO_2. The aqueous layer was then basified with NaOH and the product extracted with DCM. The organic layer was washed with $NaSO_4$ and concentrated under reduced pressure. Purification *via* short pass distillation (120 °C, 1 mBar) provided the title compound as a colourless oil (4.93 g, 33 mmol, 79%). **^{1}H NMR** (400 MHz, CDCl3) δ: 7.41 (d, *J* = 8.2 Hz, 2H), 7.38 – 7.32 (m, 2H), 7.32 – 7.25 (m, 1H), 4.21 (dd, *J* = 8.9, 3.8 Hz, 1H),

3.57 – 3.48 (m, 1H), 3.43 – 3.36 (m, 4H), 1.79 (s, 2H). **^{13}C NMR** (101 MHz, $CDCl_3$) δ: 142.1, 128.6, 127.6, 127.0, 78.7, 59.1, 55.5. **IR** ν_{max}/cm^{-1}: 2923, 2886, 1452, 1111, 968. **HRMS** m/z (ESI) calculated for $C_9H_{14}NO$ [M+H]$^+$ 152.1070, found 152.1072 (Δ = 1.21 ppm). $[\alpha]_D^{20}$ = +45.6

***tert*-Butyl (R)-(2-hydroxy-1-(4-hydroxyphenyl)ethyl)carbamate**

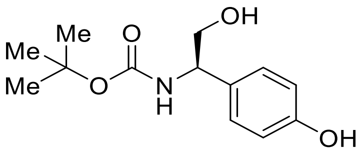

Prepared according to a procedure from Wipf.[2] A flask was charged with $NaBH_4$ and a magnetic stir bar before being evacuated and back-refilled with N_2. Then THF (70 mL) was added and the mixture cooled to 0 °C. A solution of iodine (10.7 g, 42 mmol) in THF (21 mL) was added dropwise over 1.5 hrs. Subsequently 4-hydroxy-D-phenylglycine (7 g, 42 mmol) was added in small portions over and hour and the resulting mixture was refluxed for 18 hrs. The reaction mixture was cooled on ice, carefully quenched by the slow addition of MeOH and diluted with THF (35 mL). Trimethylamine (6.2 mL) was added followed by the portion wise addition of boc anhydride (9.3 g, 40 mmol) in THF (28 mL). The mixture was allowed to warm to room temperature, stirred for 3 hrs, and concentrated under vacuum. EtOAc (56 mL) and water (42 mL) were added and the crude suspension was stirred rapidly, followed by gradual addition of (HCl (1.2 M):brine (1:1, 56 mL). The product was extracted into EtOAc (x 3) and the combined organic phases were washed with HCl (0.3 M), $NaHCO_3$, brine and water, dried over $NaSO_4$ and concentrated under vacuum. The crude solid was recrystallised from DCM: ethanol 10:1 heated under reflux. PE (60:40) was added to the cool solution until it appeared cloudy. The solution was allowed to sit overnight at 6 °C. The resultant crystals were collected via filtration and washed with cold PE (60:40) providing the title compound as a white crystalline solid (2.57 g, 10 mmol, 24%). **^{1}H NMR** (400 MHz, MeOD) δ: 7.13 (d, J = 8.1 Hz, 2H), 6.80 – 6.69 (m, 2H), 4.56 (s, 1H), 3.64 (dd, J = 11.4, 5.4 Hz, 1H), 3.59 (dd, J = 11.3, 7.7 Hz, 1H), 1.42 (s, 9H). **^{13}C NMR** (176 MHz, DMSO) δ: 156.5, 155.7, 128.3, 115.2, 78.1, 65.4, 56.6, 28.7. Quaternary carbon C_4 could not be resolved. **IR** ν_{max}/cm^{-1}: 3391, 3233, 2972, 2904, 1641, 1556, 1518, 1312, 1160, 1055, 1040. **HRMS** m/z (ESI) calculated for $C_{13}H_{19}NO_4Na$ [M+Na]$^+$ 276.1212, found 276.1215 (Δ = 1.1 ppm). **mp** 175 °C. $[\alpha]_D^{20}$ = -13.5.

***tert*-Butyl (R)-(2-methoxy-1-(4-methoxyphenyl)ethyl)carbamate**

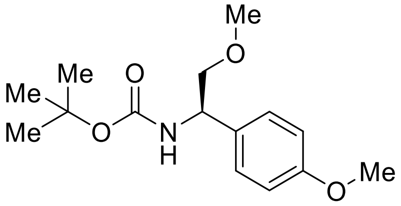

A flask was charged with *tert*-Butyl (R)-(2-hydroxy-1-(4-hydroxyphenyl)ethyl)carbamate (2.53 g, 10 mmol), methyl iodide (2.5 mL, 40 mmol), tetrabutylammonium bromide (0.32 g, 1.0 mmol), DCM (40 ml) and a magnetic stir bar. The solution was cooled to 0 °C and aqueous potassium hydroxide (4.0 ml, 20 M) was added. The mixture was stirred rapidly under N_2 for 16 hrs. The crude mixture was washed with water, aq HCl (3 M), sat. $NaHCO_3$, and brine, dried

over $NaSO_4$ and concentrated under reduced pressure. Purification *via* flash column chromatography (silica gel, EtOAc in PE (60:40), 0-20%) provided the title compound as a white crystalline solid (1.58 g, 5.62 mmol, 56%). **^{1}H NMR** (700 MHz, $CDCl_3$) δ: 7.23 (d, J = 8.2 Hz, 2H), 6.90 – 6.83 (m, 2H), 5.19 (s, 1H), 4.77 (s, 1H), 3.79 (s, 3H), 3.65 – 3.46 (m, 2H), 3.34 (s, 3H), 1.41 (s, 9H). **^{13}C NMR** (101 MHz, $CDCl_3$) δ: 158.9, 155.5, 132.7, 127.8, 114.0, 79.6, 75.7, 59.1, 55.4, 53.7, 28.5. **IR** ν_{max}/cm^{-1}: 2977, 2847, 1680, 1526, 1513, 1239, 1168, 1099· **HRMS** m/z (ESI) calculated for $C_{15}H_{23}NO_4Na$ [M+Na]$^+$ 304.1525, found 304.1520 (Δ = -1.6 ppm). **mp** 70 °C. $[\alpha]_D^{20}$ = -49.5.

(R)-2-((4-methoxybenzyl)oxy)-1-phenylethan-1-amine, 4c

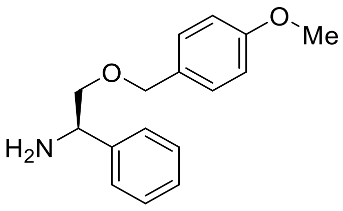

A flask was charged with (R)-2-phenylglycinol (1.25 g, 9.1 mmol), sodium iodide (140 mg, 0.9 mmol), and a magnetic stir bar, and back re-filled with N_2. DMF (12.5 ml) and 1-chloromethyl-4-methoxybenzene (1.36 ml, 10 mmol) were added and the reaction mixture was cooled to 0 °C. Sodium hydride (60% dispersion in mineral oil, 0.75g, 19 mmol) was added portion wise. The reaction mixture was allowed to heat to room temperature before refluxing at 80 °C for 6 hrs. The flask was cooled, the reaction quenched with water diluted with ether and washed with water and brine (x5). c. HCl (1 ml, 10 mmol) was added to the organic layer and the resulting precipitate was collected via filtration. The precipitate was then taken up in water, which was basified with aq. NaOH (10%) and the product extracted into DCM. Purification *via* flash column chromatography (silica gel, {5 M NH_3 in MeOH} in DCM, 0-2%) provided the title compound as a yellow oil (0.5 g, 1.95 mmol, 21%). **^{1}H NMR** (400 MHz, $CDCl_3$) δ: 7.41 – 7.21 (m, 7H), 6.87 (d, J = 8.5 Hz, 2H), 4.49 (s, 2H), 4.22 (dd, J = 8.9, 3.7 Hz, 1H), 3.80 (s, 3H), 3.59 (dd, J = 9.2, 3.7 Hz, 1H), 3.43 (t, J = 9.1 Hz, 1H), 1.79 (br s). **^{13}C NMR** (101 MHz, $CDCl_3$) δ: 159.2, 142.4, 130.2, 129.4, 128.4, 127.4, 126.9, 113.8, 76.3, 73.0, 55.6, 55.3. **IR** ν_{max}/cm^{-1}: 2854, 1611, 1511, 1244, 1172, 1061, 1030. **HRMS** m/z (ESI) calculated for $C_{16}H_{20}NO_2$ [M+H]$^+$ 258.1494, found 258.1489 (Δ = -1.9 ppm). $[\alpha]_D^{20}$ = -26.4

(R)-2-methoxy-1-(4-methoxyphenyl)ethan-1-amine, 4d

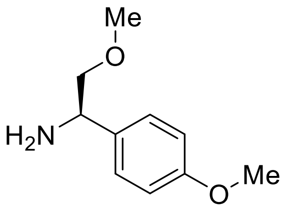

tert-Butyl (R)-(2-methoxy-1-(4-methoxyphenyl)ethyl)carbamate (0.6 g, 2.15 mmol) was dissolved in acetone (10 ml) and c. HCl was added (0,5 ml) and the mixture was refluxed overnight. After cooling to room temperature the reaction mixture was concentrated under reduced pressure, dissolved in water and washed with EtO_2. The aqueous layer was then basified with NaOH and the product extracted with DCM. The organic layer dried over $NaSO_4$ and concentrated under reduced pressure, providing the title compound as a yellow oil (0.34 g, 1.90 mmol, 88%). **^{1}H**

NMR (400 MHz, $CDCl_3$) δ: δ 7.30 (d, J = 8.2 Hz, 2H), 6.87 (d, J = 8.3 Hz, 2H), 4.15 (dd, J = 8.9, 3.9 Hz, 1H), 3.79 (d, J = 1.1 Hz, 3H), 3.47 (dd, J = 9.3, 3.9 Hz, 1H), 3.38 (d, J = 1.1 Hz, 3H), 3.34 (t, J = 9.0 Hz, 1H), 1.75 (s, 2H). **^{13}C NMR** (101 MHz, $CDCl_3$) δ: 158.9, 134.6, 127.8, 113.8, 79.1, 58.9, 55.3, 54.8. **IR** ν_{max} /cm^{-1}: 2900, 1511, 1301, 1177, 1107, 1090, 1033. **HRMS** m/z (ESI) calculated for $C_{10}H_{15}NO_2$ $[M+H]^+$ 182.1176, found 182.1177 (Δ = 0.76 ppm). $[\alpha]_D^{20}$ = -38.2

(S)-2-(benzyloxy)-1-(3,5-bis(trifluoromethyl)phenyl)ethan-1-amine, 4e

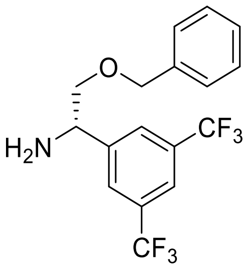

2-Benzylacetaldhyde (4 ml, 29 mmol), *(R)-tert*-butanesulfinamide (3.92 g, 33 mmol) and copper sulphate (11.2 g, 70 mmol) were added to DCM and stirred for 14 h at r.t. The crude material was filtered through celilte, concentrated under reduced pressure and purified *via* flash column chromatography to provide the Elman imine (4.73 g, 18.6 mmol, 64%). To prepare the Grignard reagent a solution of 1-bromo-3,5-bistrifluorobenzene (4.25 ml, 26 mmol) and 1,2-dibromoethane (115 µL) in Et_2O:THF (4:1, 70mL) was added dropwise to iodine activated magnesium turnings (1.28 g, 53 mmol) in Et_2O:THF (4:1, 10mL) in order to maintain a self-reflux. After addition was complete the mixture was heated under reflux for 14 h.

Trimethyl aluminium (8.6 mL) was added dropwise to a solution of the imine (3.81 g, 15 mmol) in toluene (30 mL) at -78 °C. The mixture was stirred for 1 h, followed by dropwise addition of the Grignard reagent. The reaction temperature was maintained at -78 °C for 48 h before being allowed to warm to r.t. and stirred for a further 24 h. The reaction mixture was cooled back to -78 °C and quenched by the dropwise addition of sat. $NaSO_4$, before being heated back to r.t. and filtered through celite and silica. The resulting diastereomers were separated *via* flash column chromatography (silica gel, EtOAc in 60:40 PE, 0-30%). The sulfinamide of the major diastereomer was dissolved in MeOH (25 mL), HCl (6N in IPA, 7.16 mL, 1 equiv.) was added and the mixture stirred for 1 h before being concentrated under reduced pressure. The amine HCl salt was purified *via* flash column chromatography (silica gel, EtOAc in 60:40 PE, 0-50%, then, MeOH in DCM, 0-10%), and then free based with aq. NaOH to yield the title compound as a yellow oil (1.28 g, 3.5 mmol, 23 %, 96 % e.e.).**^{1}H NMR** (500 MHz, $CDCl_3$) δ: 7.96 – 7.82 (m, 2H), 7.81 – 7.74 (m, 1H), 7.38 – 7.27 (m, 5H), 4.55 (s, 2H), 4.37 (dd, J = 7.9, 4.2 Hz, 1H), 3.61 (dd, J = 9.2, 4.2 Hz, 1H), 3.47 (dd, J = 9.2, 7.9 Hz, 1H), 1.77 (br s, 2H). **^{13}C NMR** (126 MHz, $CDCl_3$) δ: 145.6, 137.8, 131.7 (q, *J* = 33.2 Hz), 128.6, 128.0, 127.9, 127.5 (dd, *J* = 3.6, 1.3 Hz), 123.5 (q, *J* = 272.6 Hz), 121.5 (p, *J* = 3.8 Hz), 75.7, 73.6, 55.2.**^{19}F NMR** (376 MHz, $CDCl_3$) δ: -61.6. **IR** ν_{max} /cm^{-1}: 2859, 1277, 1169, 1128. **HRMS** m/z (ESI) calculated for $C_{17}H_{16}NOF_6$ $[M+H]^+$ 364.1131, found 364.1129 (Δ = -0.5 ppm). $[\alpha]_D^{20}$ = -5.4. **Chiral HPLC** Chiralpak OD (Hexane/iPrOH = 90/10, 1.0 mL min^{-1}, 30 °C) t_R = 6.9 (major), 9.5 (minor) minutes.

Ketone Synthesis

1-(4,4-dimethylcyclohexyl)ethan-1-one, 1f

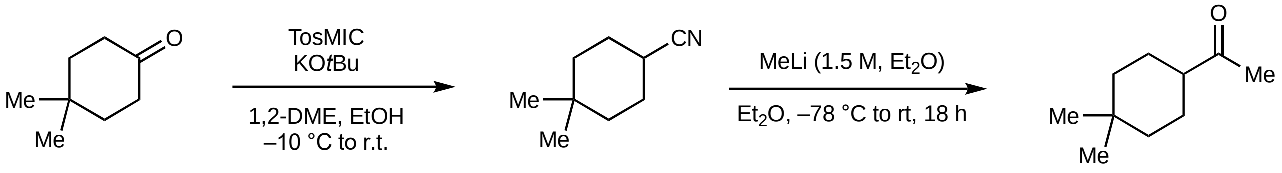

To a stirred solution of 4,4-dimethylcyclohexanone (1.26 g, 10 mmol) and TosMIC (2.54 g, 13 mmol) in anhydrous 1,2-dimethoxyethane (50 mL) and anhydrous EtOH (1 mL) at –10 °C (salt-ice bath) under N_2 was added KO^tBu (2.68 g, 24 mmol) portion-wise over 30 mins. The reaction mixture was stirred at 0 °C for 1 hour and warmed to room temperature overnight. The solvent was removed *in vacuo* and the mixture diluted with Et_2O (50 mL), washed with water (3 x 50 mL), brine (50 mL), and dried (Na_2SO_4). The solvent was removed *in vacuo* and the crude material purified by column chromatography (silica gel, EtOAc in PE, 10-50%) to afford 4,4-dimethylcyclohexane-1-carbonitrile as a colourless oil (1.43 g, 100%). Data was in accordance with the literature. **^{1}H NMR** (400 MHz, $CDCl_3$) δ: 2.54 (sept, *J* = 4.3 Hz, 1H), 1.87 – 1.70 (m, 4H), 1.51 (dd, *J* = 3.8, 7.3 Hz, 1H), 1.48 (dd, *J* = 3.8, 7.3 Hz 1H), 1.25 (dd, *J* = 4.2, 9.2 Hz, 1H), 1.22 (dd, *J* = 4.2, 9.2 Hz), 0.94 (s, 3H), 0.91 (s, 3H). **^{13}C NMR** (101 MHz, $CDCl_3$) δ: 122.7, 53.6, 36.8, 29.6, 27.8, 25.5. **HRMS** m/z (ESI) calculated for $C_9H_{15}N$ $[M+H]^+$ 138.1277, found 138.1277 (Δ = -0.3 ppm).

To a stirred solution of 4,4-dimethylcyclohexane-1-carbonitrile (0.96 g, 7 mmol) in anhydrous Et_2O (25 mL) at –78 °C was added methyl lithium (1.5 M in Et_2O) (10 mL) dropwise under N_2. The resulting solution was stirred at –78 °C for 1 hour, warmed to room temperature overnight, and subsequently cooled to at –78 °C. The reaction mixture was quenched with H_2O (10 mL) and warmed to room temperature. The organic layer was separated and aqueous extracted with Et_2O (3 x 10 mL). The organics were combined, washed with brine, dried (Na_2SO_4), and removed *in vacuo*. The crude material was purified by flash column chromatography (silica gel, EtOAc in PE, 0-15%) to afford the title compound as a colourless oil (0.36 g, 33%). **IR** ν_{max} /cm^{-1}: 2941, 2864, 2845, 1706, 1457, 1386, 1364, 1351, 1265, 1234, 1197, 1169, 1150. **^{1}H NMR** (400 MHz, $CDCl_3$) δ: 2.23 (tt, *J* = 3.8, 11.6 Hz, 1H), 2.13 (s, 3H), 1.74 – 1.66 (m, 2H), 1.53 (td, *J* = 3.5, 12.6 Hz, 2H), 1.46 (app. t, *J* = 12.8 Hz, 2H), 1.19 (td, *J* = 3.5, 12.8 Hz, 2H), 0.91 (s, 3H), 0.88 (s, 3H). **^{13}C NMR** (101 MHz, $CDCl_3$) δ: 212.6, 51.5, 38.5, 32.5, 29.9, 28.2, 24.6, 24.5. **HRMS** m/z (ESI) calculated for $C_{10}H_{18}O$ $[M+H]^+$ 155.1430, found 155.1433 (Δ = 1.7 ppm).

1-(1,4-dioxaspiro[4.5]decan-8-yl)ethan-1-one, 1i

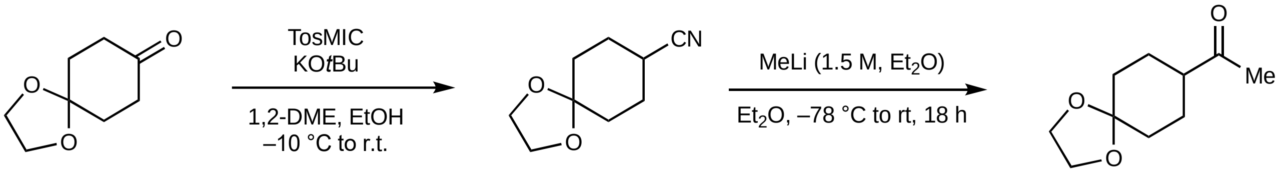

To a stirred solution of 1,4-cyclohexanedione monoethylene acetal (1.56 g, 10 mmol) and TosMIC (2.54 g, 13 mmol) in anhydrous 1,2-dimethoxyethane (50 mL) and anhydrous EtOH (1 mL) at –10 °C (salt-ice bath) under N_2 was added KO^tBu (2.68 g, 24 mmol) portion-wise over 30 mins. The reaction mixture was stirred at 0 °C for 1 hour and warmed

to room temperature overnight. The solvent was removed *in vacuo* and the mixture diluted with Et_2O (50 mL), washed with water (3 x 50 mL), brine (50 mL), and dried (Na_2SO_4). The solvent was removed *in vacuo* and the crude material purified by column chromatography (silica gel, EtOAc in PE, 10-100%) to afford 1,4-dioxaspiro[4.5]decane-8-carbonitrile as a colourless oil (1.18 g, 70%). Data was in accordance with the literature. **^{1}H NMR** (400 MHz, $CDCl_3$) δ: 3.95 (dd, *J* = 3.0, 4.2 Hz, 4H), 2.66 (app. sept, *J* = 4.2 Hz, 1H), 2.02 – 1.80 (m, 6H), 1.66 – 1.58 (m, 2H). **^{13}C NMR** (101 MHz, $CDCl_3$) δ: 121.9, 107.2, 64.6, 32.8, 27.1, 26.8. **HRMS** m/z (ESI) calculated for $C_9H_{13}NO_2$ $[M+H]^+$ 168.1019, found 168.1018 (Δ = -0.5 ppm).

To a stirred solution of 1,4-dioxaspiro[4.5]decane-8-carbonitrile (1.18 g, 7 mmol) in anhydrous Et_2O (25 mL) at –78 °C was added methyl lithium (1.5 M in Et_2O) (10 mL) dropwise under N_2. The resulting solution was stirred at –78 °C for 1 hour, warmed to room temperature overnight, and subsequently cooled to at –78 °C. The reaction mixture was quenched with H_2O (10 mL) and warmed to room temperature. The organic layer was separated and aqueous extracted with Et_2O (3 x 10 mL). The organics were combined, washed with brine, dried (Na_2SO_4), and removed *in vacuo*. The crude material was purified by flash column chromatography (silica gel, EtOAc in PE, 0-50%) to afford the title compound as a colourless oil (0.539 g, 42%). **IR** ν_{max} /cm^{-1}: 2944, 2882, 1704, 1447, 1362, 1305, 1245, 1169, 1136, 1096, 1033. **^{1}H NMR** (400 MHz, $CDCl_3$) δ: 3.94 (app. t, *J* = 2.2 Hz, 4H), 2.34 (tt, *J* = 3.7, 11.2 Hz, 1H), 2.14 (s, 3H), 1.91 – 1.86 (m, 2H), 1.82 – 1.76 (m, 2H), 1.70 (app. qd, *J* = 3.7, 12.6 Hz, 2H), 1.55 (td, *J* = 4.5, 12.6 Hz, 2H). **^{13}C NMR** (101 MHz, $CDCl_3$) δ: 211.3, 108.2, 64.41, 64.38, 50.0, 34.0, 27.9, 25.9. **HRMS** m/z (ESI) calculated for $C_{10}H_{16}O_3$ $[M+H]^+$ 185.1172, found 185.1173 (Δ = 0.2 ppm).

1-(2,3-dihydro-1*H*-inden-2-yl)ethan-1-one, 1j

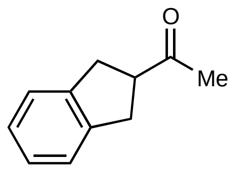

To a stirred solution of tetra-*N*-butylammonium bromide (0.16 g, 0.5 mmol) in 48% w/w aqueous NaOH (5 mL) was added α,α'-dibromo-*o*-xylene (2.64 g, 10 mmol) in toluene (6 mL), followed by acetylacetone (1.02 mL, 10 mmol) in toluene (6 mL). The resulting mixture was heated at 80 °C for 6 hours, cooled to room temperature, and diluted with water (20 mL). The organic layer was removed and the aqueous extracted with toluene (3 x 10 mL), the organics were combined, dried over Na_2SO_4, and the solvent removed *in vacuo*. The crude reaction mixture was purified by flash column chromatography (silica gel, 0 to 10% EtOAc in P.E.) to afford the product as a colourless oil (0.52 g, 33%). **IR** ν_{max} /cm^{-1}: 2940, 1707, 1484, 1459, 1436, 1358, 1252, 1199, 1163. **^{1}H NMR** (400 MHz, $CDCl_3$) δ: 7.23 – 7.19 (m, 2H), 7.18 – 7.14 (m, 2H), 3.45 (qt, *J* = 9.0 Hz, 1 H), 3.19 (qd, *J* = 5.1, 13.8 Hz, 4H), 2.25 (s, 3H). **^{13}C NMR** (101 MHz, $CDCl_3$) δ: 209.4, 141.6, 126.8, 124.5, 52.0, 35.1, 28.6. **IR** ν_{max} /cm^{-1}: 2940, 1707, 1484, 1459, 1436, 1358, 1252, 1199, 1163, 1025. **HRMS** m/z (ESI) calculated for $C_{11}H_{12}O$ $[M+H]^+$ 161.0961, found 161.0960 (Δ = -0.7 ppm).

Acceptor Synthesis

N-methyl-N-(3-phenyl-3-(4-(trifluoromethyl)phenoxy)propyl)ethenesulfonamide, 2f

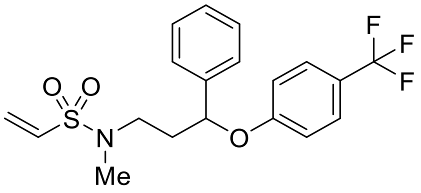

Prepared according to a procedure from Grela.[3] 2-Chloroethanesulphonyl chloride (0.84 mL, 8.0 mmol) was added to a solution of fluoxetine hydrochloride (3.05 g, 8.8 mmol) and trimethylamine (4.7 mL, 33 mmol) in DCM (30 mL, 0 °C). The reaction was allowed to warm to room temperature and was stirred for 5 h. The reaction mixture was washed with water (30 mL, x3, 0 °C), dried over $NaSO_4$, filtered and concentrated under reduced pressure. Purification *via* flash column chromatography (silica gel, EtOAc in PE (60:40), 0-25%) provided the title compound as a colourless oil (1.35 g, 3.4 mmol, 43%). **^{1}H NMR** (500 MHz, $CDCl_3$) δ: 7.48 – 7.41 (m, 2H), 7.39 – 7.31 (m, 4H), 7.31 – 7.27 (m, 1H), 6.96 – 6.89 (m, 2H), 6.41 (dd, *J* = 16.6, 10.0 Hz, 1H), 6.21 (d, *J* = 16.6 Hz, 1H), 5.97 (d, *J* = 9.9 Hz, 1H), 5.30 (dd, *J* = 8.9, 4.0 Hz, 1H), 3.41 – 3.33 (m, 1H), 3.29 – 3.19 (m, 1H), 2.80 (s, 3H), 2.33 – 2.21 (m, 1H), 2.19 – 2.07 (m, 1H). **^{13}C NMR** (126 MHz, $CDCl_3$) δ: 160.3 (d, *J* = 1.3 Hz), 140.5, 132.7, 129.0, 128.2, 128.0, 126.9 (q, *J* = 3.8 Hz), 125.9, 124.5 (q, *J* = 271.2 Hz), 123.1 (q, *J* = 32.7 Hz), 115.9, 77.4, 47.1, 37.7, 35.3. **^{19}F NMR** (376 MHz, $CDCl_3$) δ: -61.6. **IR** ν_{max}/cm^{-1}: 3062, 3032, 1614, 1517, 1326, 1250, 1152, 1110, 1068. **HRMS** m/z (ESI) calculated for $C_{19}H_{22}NO_3F_3S$ [M+H]$^+$ 401.1267, found 401.1256 (Δ = -2.6 ppm).

4-(vinylsulfonyl)morpholine, 2g

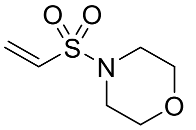

Prepared according to a procedure from Grela.[3] 2-Chloroethanesulphonyl chloride (0.84 mL, 8.0 mmol) was added to a solution of morpholine (0.77 mL, 8.8 mmol) and trimethylamine (3.4 mL, 24 mmol) in DCM (30 mL, 0 °C). The reaction was allowed to warm to room temperature and was stirred for 2 h. The reaction mixture was washed with water (30 mL, 0 °C), HCl (1 M, 30 mL, 0 °C) and water (30 mL, 0 °C), dried over $NaSO_4$, filtered and concentrated under reduced pressure. Purification *via* flash column chromatography (silica gel, EtOAc in PE (60:40), 10-40%) provided the title compound as a white crystalline solid (870 mg, 5.8 mmol, 72%). Data was in accordance with the literature. **^{1}H NMR** (500 MHz, $CDCl_3$) δ: 6.43 (dd, J = 16.6, 9.9 Hz, 1H), 6.27 (d, J = 16.6 Hz, 1H), 6.10 (d, J = 9.9 Hz, 1H), 3.82 – 3.69 (m, 4H), 3.20 – 3.07 (m, 4H). **^{13}C NMR** (126 MHz, $CDCl_3$) δ: 131.9, 129.7, 66.4, 45.8. **IR** ν_{max} /cm^{-1}: 2970, 2898, 2858, 1343, 1327, 1260, 1154, 1112, 1074. **HRMS** m/z (ESI) calculated for $C_6H_{12}NO_3S$ [M+H]$^+$ 178.0532, found 178.0531 (Δ = -1.1 ppm). **mp** 38 °C.

6-fluoro-3-(1-(vinylsulfonyl)piperidin-4-yl)benzo[d]isoxazole, 2h

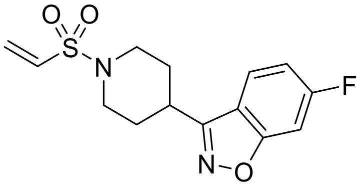

Prepared according to a procedure from Grela.[3] 2-Chloroethanesulphonyl chloride (0.49 mL, 4.6 mmol) was added to a solution of 6-fluoro-3-(4-piperidyl)-1,2-benzoisoxazole hydrochloride (1.31 g, 5.1 mmol) and trimethylamine (2.7 mL, 19 mmol) in DCM (20 mL, 0 °C). The reaction was allowed to warm to room temperature and was stirred for 5 h. The reaction mixture was washed with water (30 mL, x3, 0 °C), dried over $NaSO_4$, filtered and concentrated under reduced pressure. Purification *via* flash column chromatography (silica gel, EtOAc in PE (60:40), 10-40%) provided the title compound as a white crystalline solid (400 mg, 1.3 mmol, 28%).**^{1}H NMR** (400 MHz, $CDCl_3$) δ: 7.64 (dd, *J* = 8.7, 5.0 Hz, 1H), 7.28 – 7.25 (m, 1H), 7.09 (td, *J* = 8.8, 2.2 Hz, 1H), 6.48 (dd, *J* = 16.6, 9.9 Hz, 1H), 6.28 (d, *J* = 16.6 Hz, 1H), 6.07 (d, *J* = 9.9 Hz, 1H), 3.83 (dt, *J* = 12.3, 4.0 Hz, 2H), 3.22 (tt, *J* = 9.8, 5.0 Hz, 1H), 2.99 – 2.83 (m, 2H), 2.26 – 2.05 (m, 4H).**^{13}C NMR** (101 MHz, $CDCl_3$) δ: 164.4 (d, *J* = 251.5 Hz), 164.1 (d, *J* = 13.6 Hz), 160.1, 132.9, 128.8, 122.3 (d, *J* = 11.3 Hz), 117.1, 112.9 (d, *J* = 25.5 Hz), 97.8 (d, *J* = 26.6 Hz), 45.5, 33.6, 29.9. **^{19}F NMR** (376 MHz, $CDCl_3$) δ: -109.9. **IR** ν_{max} /cm^{-1}: 3080, 2928, 2851, 1613, 1345, 1331, 1272, 1249, 1154, 1122. **HRMS** m/z (ESI) calculated for $C_{14}H_{16}N_2O_3FS$ $[M+H]^+$ 311.0860, found 311.0861 (Δ = 0.4 ppm). **mp** 110 °C.

Computational studies

DFT calculations

All calculations were performed using the Gaussian 09 D.01.[4] Geometries were optimised at the wB97XD/6-31'+g(d,p) level of theory in DCM (IEFPCM).[5-8] Vibrational analysis confirmed that all reported structures were either true minima (no imaginary frequencies), or true first-order saddle points (for transition states, one imaginary frequency) on the potential energy surface. Transition states where confirmed by relaxation towards both the reactant and the products. Single-point energies of the optimised structures were calculated at the wB97XD/6-311++g(d,p) level of theory in DCM (IEFPCM).[10] wB97XD is a long-range corrected (LC) hybrid functional incorporating additional empirical dispersion corrections which has shown to be effective in predicting noncovalent interactions.[5] These basis sets and functional have been benchmarked for radical 1,2-additions and asymmetric radical reactions.[11,12] Gibbs free energies were obtained by adding the thermal correction term from geometry optimisation to the single point energy. To account for the change in the standard state (1 atm → 1 M) an entropic correction term of 1.89 kcal/mol (−RTlnQ) was added to all considered species.[13]

Gibbs Free Energy (6-311++g(d,p)) *= Single point energy (6-311++g(d,p)) + Thermal correction to Gibbs Free Energy (6-31'+g(d,p)) +* $x(-RT\ln Q)$

$T = 298$ K, x = *number of species*

Computed intermediates and transition states for the enantiodetermining step

1,2-addition of α-amino radical to phenylvinyl sulfone

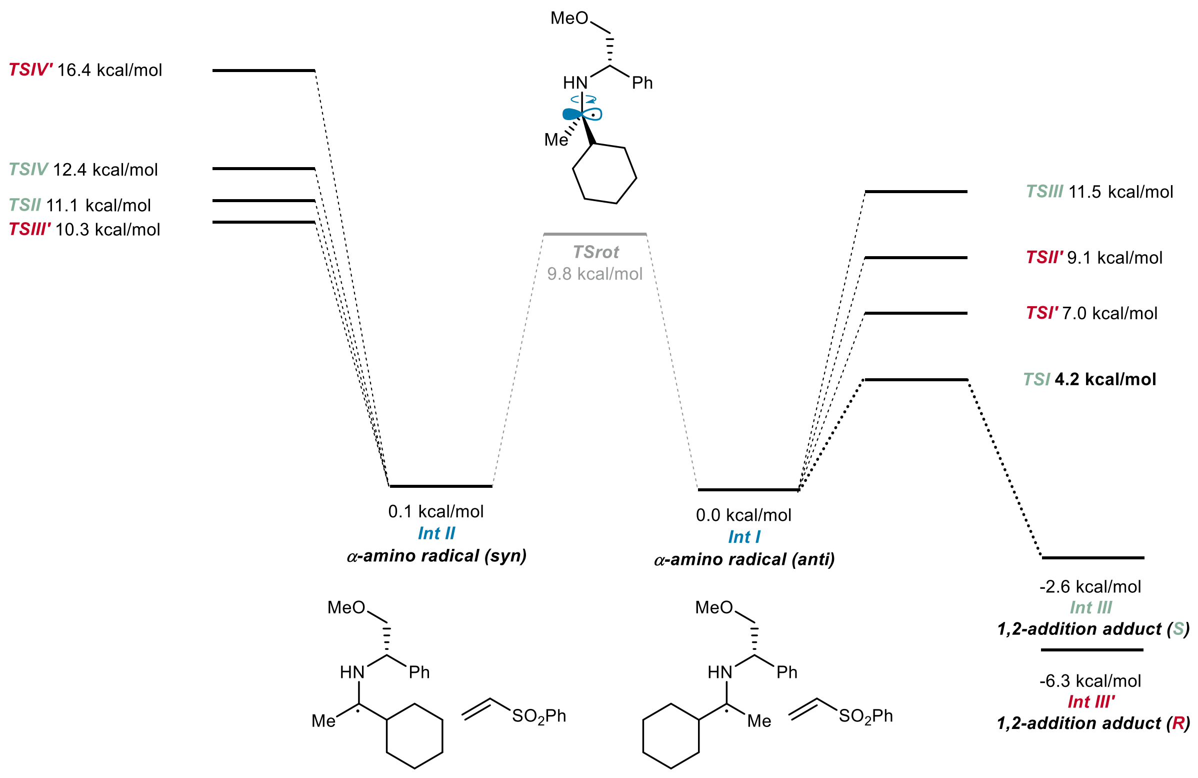

TS_I-TS_{IV} lead to the formation of (*S*) ATA geometry

$TS_{I'}$-$TS_{IV'}$ lead to the formation of (*R*) ATA geometry

α-amino radical barrier to rotation (syn/anti) = 9.8 kcal mol^{-1}

Syn α-amino radical

Geometry optimisation:
Sum of electronic and thermal Free Energies = -792.853487 Hartrees
Thermal correction to Gibbs Free Energy = 0.354255 Hartrees

Single Point Energy:
Energy = -793.360214 Hartrees

C	0.955826	0.640516	-1.041816
C	0.107824	0.864567	-2.255819
H	0.235687	0.048654	-2.978620
H	-0.955417	0.923175	-2.004208
H	0.369018	1.803845	-2.779283
C	0.650256	1.364372	0.245023
C	0.843547	2.893515	0.116664
C	-0.750795	1.056367	0.796837
H	1.357857	1.019060	1.011770
C	0.599736	3.606599	1.450055
H	0.141739	3.280436	-0.638030
H	1.855090	3.107810	-0.254059
C	-0.992262	1.765388	2.132900
H	-1.515861	1.385456	0.078011
H	-0.867321	-0.028906	0.911781
C	-0.787626	3.278390	2.010020
H	0.716163	4.691027	1.328030
H	1.365559	3.285583	2.172689
H	-2.003974	1.548350	2.498054
H	-0.292936	1.366226	2.883432
H	-0.929417	3.762675	2.984357
H	-1.553765	3.690977	1.336078
N	2.279114	0.284297	-1.346493
H	2.605981	0.582168	-2.259028
C	3.344099	0.285017	-0.364764
H	3.467285	1.278574	0.101415
C	3.128693	-0.729638	0.748394
C	3.665723	-0.487936	2.016158
C	2.434512	-1.920507	0.522675
C	3.516097	-1.419587	3.042436
H	4.195837	0.444511	2.204320
C	2.280098	-2.853415	1.548101
H	2.002214	-2.101827	-0.457828
C	2.820644	-2.606825	2.810556
H	3.936286	-1.215133	4.023921
H	1.732737	-3.774052	1.361986
H	2.694699	-3.332194	3.610188
C	4.654525	-0.045039	-1.077129
H	5.478889	-0.045167	-0.347195
H	4.584867	-1.052409	-1.522013
O	4.883226	0.917716	-2.083159
C	6.033931	0.636767	-2.851502

H	5.940028	-0.330864	-3.366854
H	6.130983	1.431818	-3.594205
H	6.936423	0.617701	-2.222391

Anti α-amino radical

Geometry optimisation:
Sum of electronic and thermal Free Energies = -792.853719 Hartrees
Thermal correction to Gibbs Free Energy = 0.355363 Hartrees

Single Point Energy:
Energy = -793.361423 Hartrees

C	-1.505601	0.056812	-0.120089
C	-0.720930	0.119989	-1.396663
H	-0.907190	1.051811	-1.960412
H	0.360233	0.069551	-1.223629
H	-0.987885	-0.717674	-2.048272
C	-2.962221	-0.338544	-0.165382
C	-3.808135	0.656060	-0.992375
C	-3.194720	-1.771688	-0.671507
H	-3.345356	-0.302186	0.869247
C	-5.294111	0.287186	-0.985109
H	-3.440136	0.654642	-2.029805
H	-3.659603	1.6713	-0.603036
C	-4.681871	-2.141033	-0.658097
H	-2.818433	-1.863969	-1.701050
H	-2.618909	-2.477224	-0.059020
C	-5.512712	-1.147267	-1.475294
H	-5.862201	0.993339	-1.604284
H	-5.684293	0.380311	0.039814
H	-4.819242	-3.159232	-1.043113
H	-5.045084	-2.144428	0.380879
H	-6.577263	-1.408719	-1.425666
H	-5.217467	-1.216124	-2.533258
N	-1.214036	1.006548	0.877009
H	-1.678553	0.785987	1.750821
C	0.151436	1.442517	1.124993
H	0.849618	0.588554	1.094700
C	0.629257	2.493183	0.133669
C	1.960829	2.509926	-0.287357
C	-0.240565	3.482088	-0.332542
C	2.417704	3.494715	-1.163627
H	2.6443	1.737584	0.061929
C	0.211484	4.468052	-1.207954
H	-1.281232	3.460530	-0.018356
C	1.542587	4.476893	-1.627439
H	3.454708	3.489020	-1.489496
H	-0.477414	5.229041	-1.566003
H	1.894105	5.241599	-2.315306

C	0.219741	2.042562	2.528565
H	1.246388	2.384292	2.731686
H	-0.450609	2.917608	2.585162
O	-0.175720	1.066109	3.466951
C	-0.244234	1.574859	4.782235
H	-0.985732	2.384631	4.855315
H	-0.545063	0.752103	5.434787
H	0.732550	1.958781	5.112240

TS_{rot}

Geometry optimisation:
Sum of electronic and thermal Free Energies = -792.839031 Hartrees
Thermal correction to Gibbs Free Energy = 0.354007 Hartrees

Single Point Energy:
Energy = -793.345421 Hartrees

C	-0.859259	-0.507818	0.228031
C	-0.518115	-1.939302	0.520101
H	-0.507360	-2.537565	-0.409474
H	0.481231	-2.035368	0.959463
H	-1.235902	-2.405099	1.201906
C	-2.281501	-0.017928	0.198909
C	-2.928876	-0.250616	-1.187689
C	-3.190517	-0.592663	1.295149
H	-2.230517	1.073662	0.342368
C	-4.335196	0.351732	-1.259471
H	-2.982287	-1.334381	-1.375453
H	-2.292061	0.183434	-1.968662
C	-4.589946	0.028469	1.235184
H	-3.287498	-1.680825	1.165791
H	-2.738016	-0.427811	2.281767
C	-5.232434	-0.182714	-0.139199
H	-4.785530	0.146819	-2.238944
H	-4.257806	1.445548	-1.168423
H	-5.225535	-0.398721	2.020874
H	-4.515705	1.107256	1.438517
H	-6.216809	0.300203	-0.177850
H	-5.400416	-1.259226	-0.294918
N	0.059618	0.301281	-0.510171
H	0.244250	-0.076779	-1.437940
C	1.305828	0.677333	0.155598
H	1.036132	0.976036	1.176515
C	2.378910	-0.404486	0.248829
C	3.022637	-0.655325	1.463522
C	2.766201	-1.138335	-0.877233
C	4.022536	-1.623460	1.558270
H	2.731352	-0.093199	2.349123
C	3.765161	-2.106505	-0.789340
H	2.280298	-0.965341	-1.835909

C	4.396189	-2.353755	0.430553
H	4.506182	-1.809124	2.513902
H	4.050756	-2.668893	-1.674745
H	5.172270	-3.111270	0.500559
C	1.914466	1.903759	-0.526903
H	2.9128	2.094949	-0.099535
H	2.049672	1.699897	-1.604877
O	1.085285	3.025287	-0.339388
C	1.604823	4.183060	-0.952774
H	1.717792	4.043568	-2.039060
H	0.897333	4.995213	-0.769019
H	2.584466	4.455994	-0.530270

Phenyl vinyl sulfone

Geometry optimisation:
Sum of electronic and thermal Free Energies = -857.963316 Hartrees
Thermal correction to Gibbs Free Energy = 0.108333 Hartrees

Single Point Energy:
Energy = -858.193912 Hartrees

C	-1.227152	-0.459475	0.574028
H	-0.672898	-1.392843	0.537939
H	-1.885724	-0.280867	1.420480
C	-1.104785	0.433903	-0.401182
H	-0.470096	0.328726	-1.277303
S	-2.040084	1.943508	-0.369079
C	-0.798275	3.205878	-0.154361
C	-0.271865	3.839246	-1.278485
C	-0.359086	3.507681	1.134904
C	0.719118	4.803154	-1.102104
H	-0.640271	3.590114	-2.269032
C	0.631523	4.472238	1.296313
H	-0.791866	3.004078	1.994356
C	1.169054	5.116312	0.180378
H	1.136691	5.309491	-1.967224
H	0.981536	4.722572	2.293380
H	1.941330	5.869019	0.312110
O	-2.902016	1.936399	0.814731
O	-2.642438	2.116806	-1.693687

TS_I

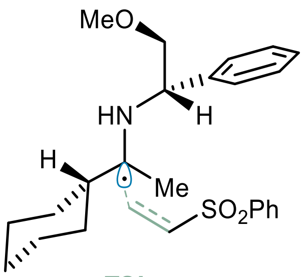

Geometry optimisation:
Sum of electronic and thermal Free Energies = -1650.807000 Hartrees
Thermal correction to Gibbs Free Energy = 0.488896 Hartrees

Single Point Energy:
Energy = -1651.571416 Hartrees

C	-2.745384	-0.603551	-1.646570
C	-4.159949	-1.833946	-0.153601
H	-4.225368	-0.948714	0.473858
H	-4.841788	-1.887704	-0.996273
C	-3.624854	-2.986938	0.334459
H	-3.631240	-3.941038	-0.184479
S	-2.805407	-3.034341	1.872571
O	-3.077018	-4.345806	2.481004
O	-3.124463	-1.818753	2.634331
C	-1.0504	-2.975512	1.525603
C	-0.316183	-1.862094	1.921773
C	-0.458795	-4.025621	0.822688
C	1.037559	-1.790830	1.595148
H	-0.805615	-1.063711	2.470576
C	0.891743	-3.943529	0.498393
H	-1.044076	-4.892702	0.528638
C	1.637678	-2.825180	0.879752
H	1.615596	-0.919658	1.889531
H	1.363562	-4.752555	-0.051792
H	2.690794	-2.763124	0.620101
C	-1.432007	-1.308864	-1.495077
H	-0.732310	-1.010405	-2.291016
H	-0.949809	-1.096102	-0.539636
H	-1.571891	-2.392127	-1.559355
C	-3.496122	-0.767387	-2.955545
C	-2.964935	0.201680	-4.037178
C	-3.477162	-2.203032	-3.499870
H	-4.550007	-0.491024	-2.772256
C	-3.763372	0.082810	-5.338148
H	-1.906434	-0.028126	-4.232248
H	-3.002249	1.230759	-3.661967
C	-4.293776	-2.323302	-4.789666
H	-2.438254	-2.491594	-3.714135
H	-3.849144	-2.908426	-2.747062

C	-3.782045	-1.355975	-5.860287
H	-3.346444	0.759298	-6.094424
H	-4.796833	0.413141	-5.154493
H	-4.254141	-3.355122	-5.159287
H	-5.350434	-2.104413	-4.574645
H	-4.399314	-1.424860	-6.764399
H	-2.761090	-1.647804	-6.148965
N	-2.872248	0.663625	-1.103600
H	-3.797954	1.056785	-1.226468
C	-2.274113	1.048665	0.163811
H	-2.311432	0.203549	0.871501
C	-0.828691	1.510950	0.039208
C	-0.042934	1.608031	1.192183
C	-0.271329	1.869430	-1.189097
C	1.276367	2.049644	1.120561
H	-0.465144	1.332204	2.157060
C	1.051687	2.305809	-1.265763
H	-0.875176	1.790376	-2.089286
C	1.830112	2.396674	-0.112326
H	1.873172	2.115842	2.026741
H	1.475746	2.572305	-2.230530
H	2.861321	2.734387	-0.173430
C	-3.113546	2.175583	0.763874
H	-2.687536	2.474003	1.732901
H	-3.087817	3.053703	0.094824
O	-4.435913	1.714299	0.919080
C	-5.298522	2.700603	1.446853
H	-5.355199	3.574760	0.781014
H	-6.289172	2.249937	1.536770
H	-4.960832	3.031714	2.439886

TS_{II}

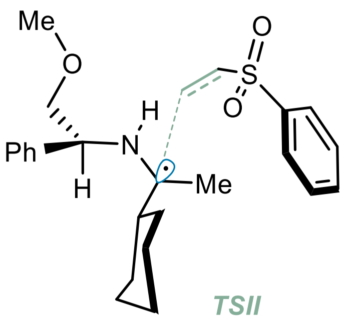

Geometry optimisation:
Sum of electronic and thermal Free Energies = -1650.796856 Hartrees
Thermal correction to Gibbs Free Energy = 0.490577 Hartrees

Single Point Energy:
Energy = -1651.562751 Hartrees

C	-1.722964	0.735739	-1.153464
C	-0.609037	1.710322	-2.788109

H	-1.435130	2.413055	-2.813023
H	0.248308	2.002506	-2.184724
C	-0.412534	0.973735	-3.940135
H	-1.188102	0.815602	-4.684434
S	1.066291	0.165576	-4.343551
O	1.251762	0.278858	-5.800345
O	2.148942	0.626959	-3.461628
C	0.825230	-1.580202	-4.008901
C	-0.140125	-2.277735	-4.737799
C	1.583492	-2.209782	-3.026282
C	-0.357533	-3.623734	-4.461442
H	-0.7143	-1.776057	-5.512030
C	1.361939	-3.560997	-2.760278
H	2.331548	-1.646394	-2.477291
C	0.390678	-4.264371	-3.470510
H	-1.108086	-4.174477	-5.021105
H	1.948824	-4.060654	-1.994713
H	0.218040	-5.315776	-3.257964
C	-2.200558	-0.497154	-1.881421
H	-2.882698	-0.229616	-2.696356
H	-1.369764	-1.055240	-2.317660
H	-2.740736	-1.158197	-1.189251
C	-0.678645	0.546585	-0.061077
C	-1.302072	-0.039502	1.226691
C	0.506238	-0.332382	-0.477351
H	-0.271148	1.534032	0.190161
C	-0.265652	-0.124290	2.351411
H	-1.689893	-1.045749	1.010220
H	-2.156667	0.557368	1.559779
C	1.554982	-0.417624	0.634164
H	0.153348	-1.347713	-0.706172
H	0.971947	0.059598	-1.384580
C	0.952184	-0.951826	1.935001
H	-0.728114	-0.549305	3.250784
H	0.059279	0.894592	2.611002
H	2.385385	-1.056295	0.307650
H	1.977406	0.582952	0.810064
H	1.703515	-0.953070	2.734038
H	0.643678	-1.997467	1.785383
N	-2.745673	1.632999	-0.920275
H	-3.596827	1.470164	-1.442266
C	-2.928376	2.649551	0.110920
H	-3.260145	2.170909	1.044465
C	-1.707286	3.490341	0.435410
C	-1.235690	3.541374	1.749309
C	-1.052280	4.239935	-0.546052
C	-0.113626	4.302004	2.075053
H	-1.737170	2.961728	2.521943
C	0.071346	4.998778	-0.226285
H	-1.410315	4.222806	-1.571831
C	0.547923	5.027245	1.084880

H	0.248098	4.320562	3.099717
H	0.577048	5.565793	-1.003309
H	1.429044	5.612892	1.332921
C	-4.072563	3.559481	-0.337953
H	-4.285545	4.288531	0.458577
H	-3.782593	4.113390	-1.246391
O	-5.201841	2.757149	-0.601553
C	-6.296343	3.498393	-1.101095
H	-6.037502	4.000316	-2.045074
H	-7.109813	2.792064	-1.280007
H	-6.626461	4.254904	-0.374510

TS_{III}

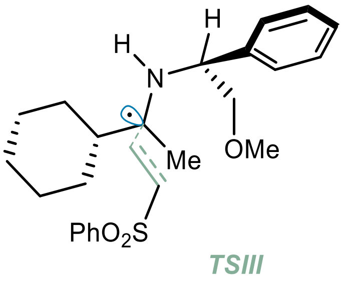

Geometry optimisation:
Sum of electronic and thermal Free Energies = -1650.796193 Hartrees
Thermal correction to Gibbs Free Energy = 0.490597 Hartrees

Single Point Energy:
Energy = -1651.562392 Hartrees

C	0.940576	2.607989	0.218182
C	1.308124	1.613251	2.207218
H	0.242933	1.402901	2.220933
H	1.961735	0.851113	1.788991
C	1.805622	2.439276	3.184550
H	1.201484	3.115476	3.780841
S	3.498446	2.516154	3.561230
O	3.634121	2.573734	5.026820
O	4.217587	1.459701	2.833599
C	4.104207	4.085074	2.941706
C	3.568747	5.265528	3.457065
C	5.068814	4.103583	1.938508
C	4.001529	6.484910	2.945707
H	2.812441	5.236789	4.236557
C	5.502692	5.331256	1.439636
H	5.464343	3.169348	1.551332
C	4.967139	6.518602	1.937882
H	3.579898	7.408426	3.331950
H	6.256641	5.357721	0.658088
H	5.302532	7.472586	1.540245
C	1.988403	3.680759	0.292345
H	1.922940	4.384345	-0.547969

H	1.919082	4.259805	1.215275
H	2.977207	3.215007	0.257471
C	1.266692	1.439018	-0.705603
C	0.307465	0.241344	-0.617325
C	1.347306	1.927192	-2.170426
H	2.268764	1.083088	-0.424103
C	0.747110	-0.892359	-1.549841
H	-0.707611	0.543194	-0.919294
H	0.237706	-0.126517	0.412359
C	1.764261	0.799481	-3.119014
H	0.361615	2.318952	-2.461041
H	2.059974	2.756687	-2.251804
C	0.837731	-0.412376	-3.000325
H	0.044667	-1.730427	-1.468237
H	1.728824	-1.267541	-1.225254
H	1.777690	1.171021	-4.151239
H	2.793429	0.492538	-2.880346
H	1.187326	-1.224012	-3.649876
H	-0.167938	-0.134814	-3.350316
N	-0.390674	2.969136	0.260297
H	-1.022362	2.271518	-0.102321
C	-0.983154	4.305628	0.235281
H	-2.050906	4.130693	0.420443
C	-0.502760	5.207407	1.363406
C	-0.730232	4.794732	2.680327
C	0.124224	6.436281	1.146442
C	-0.336967	5.585164	3.756750
H	-1.214021	3.836243	2.858272
C	0.518793	7.232801	2.222392
H	0.328863	6.783147	0.137194
C	0.289960	6.811148	3.529829
H	-0.519482	5.244821	4.772966
H	1.012818	8.182333	2.034254
H	0.602064	7.429893	4.367196
C	-0.913856	4.930085	-1.160099
H	-1.411009	5.913407	-1.158094
H	0.128496	5.074113	-1.485710
O	-1.577013	4.053293	-2.042718
C	-1.451566	4.444035	-3.392527
H	-0.395964	4.450983	-3.705567
H	-1.999230	3.715232	-3.994430
H	-1.878270	5.444107	-3.561071

TS$_{IV}$

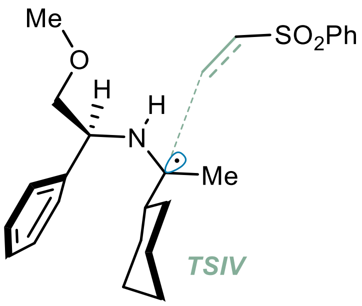

Geometry optimisation:
Sum of electronic and thermal Free Energies = -1650.794473 Hartrees
Thermal correction to Gibbs Free Energy = 0.491157 Hartrees

Single Point Energy:
Energy = -1651.561391 Hartrees

C	1.213023	3.183059	-0.889682
C	1.439205	3.776371	-3.101901
H	1.309466	4.804390	-2.778470
H	2.457246	3.408575	-3.193705
C	0.448741	3.206491	-3.855476
H	-0.5611	3.598625	-3.912454
S	0.709914	1.775240	-4.802450
O	0.023039	1.956039	-6.092438
O	2.139642	1.433893	-4.815537
C	-0.139460	0.433368	-3.966125
C	-1.503773	0.555131	-3.696821
C	0.573222	-0.705044	-3.599776
C	-2.158058	-0.479393	-3.034393
H	-2.050166	1.445319	-3.997259
C	-0.094172	-1.739706	-2.944551
H	1.635295	-0.770811	-3.815174
C	-1.4538	-1.625744	-2.658964
H	-3.218653	-0.392597	-2.816715
H	0.451616	-2.633102	-2.654912
H	-1.968414	-2.432368	-2.144165
C	1.647595	1.749981	-1.064892
H	0.989145	1.225242	-1.762485
H	2.669907	1.677529	-1.444547
H	1.595122	1.216364	-0.105590
C	2.137811	4.093183	-0.094140
C	2.357549	3.527301	1.330330
C	3.504834	4.354550	-0.752579
H	1.653199	5.070716	0.017219
C	3.213355	4.461949	2.186617
H	2.868314	2.557170	1.249415
H	1.392665	3.343870	1.813261
C	4.375877	5.273884	0.111241
H	4.030240	3.401673	-0.909993
H	3.376302	4.815906	-1.735980

C	4.566962	4.724970	1.525783
H	3.348667	4.026230	3.184383
H	2.681123	5.413963	2.326196
H	5.346348	5.422713	-0.377565
H	3.898369	6.263713	0.172146
H	5.161344	5.422790	2.128348
H	5.134421	3.783489	1.475634
N	-0.149604	3.284415	-0.671680
H	-0.682536	2.625913	-1.223749
C	-0.941164	4.452909	-0.274954
H	-1.977766	4.113932	-0.390154
C	-0.772712	4.787360	1.204932
C	-1.205459	3.843921	2.143877
C	-0.210149	5.978484	1.666199
C	-1.061270	4.073213	3.508491
H	-1.644463	2.911519	1.794635
C	-0.063506	6.214232	3.034463
H	0.138791	6.733607	0.966080
C	-0.483316	5.262072	3.959340
H	-1.398582	3.325747	4.221701
H	0.388289	7.142591	3.373942
H	-0.363277	5.443170	5.024046
C	-0.827214	5.668052	-1.196908
H	-1.410564	6.498324	-0.767886
H	0.214907	6.013260	-1.298636
O	-1.349163	5.315652	-2.455599
C	-1.232547	6.3559	-3.403252
H	-0.176906	6.608096	-3.587487
H	-1.680888	5.999139	-4.333313
H	-1.761144	7.260999	-3.069731

TS$_{I'}$

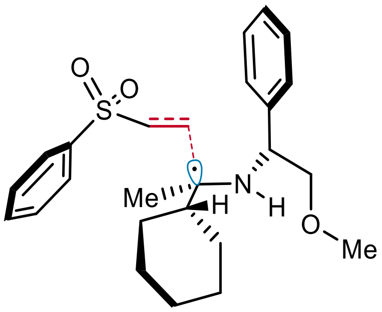

TSI'

Geometry optimisation:
Sum of electronic and thermal Free Energies = -1650.802973 Hartrees
Thermal correction to Gibbs Free Energy = 0.486757 Hartrees

Single Point Energy:
Energy = -1651.565315 Hartrees

C	-1.958387	0.502103	-1.512132
C	-0.461028	1.672280	-2.868882
H	-1.352541	2.150989	-3.263305
H	-0.050151	2.076501	-1.948539
C	0.360125	1.015330	-3.742349
H	0.079724	0.725825	-4.751235
S	1.955109	0.486681	-3.293912
O	2.854304	0.721928	-4.435344
O	2.318054	1.047390	-1.985378
C	1.834769	-1.290291	-3.097293
C	1.602247	-2.078099	-4.225670
C	1.908136	-1.849819	-1.824902
C	1.418162	-3.448314	-4.067957
H	1.558107	-1.625969	-5.212671
C	1.731485	-3.225337	-1.678604
H	2.099226	-1.213309	-0.965997
C	1.478458	-4.020653	-2.795520
H	1.227948	-4.069684	-4.938335
H	1.789779	-3.673451	-0.690860
H	1.331576	-5.090424	-2.675849
C	-0.994511	-0.080401	-0.518398
H	-0.365359	0.685963	-0.055858
H	-1.526110	-0.615369	0.284888
H	-0.331053	-0.790559	-1.012722
C	-2.678129	-0.4330	-2.475802
C	-1.795378	-1.540659	-3.065112
C	-3.917980	-1.077794	-1.811215
H	-3.042892	0.184439	-3.316085
C	-2.565410	-2.397834	-4.071805
H	-1.435681	-2.193996	-2.256670
H	-0.910773	-1.110315	-3.536779
C	-4.695965	-1.950729	-2.800030
H	-3.581445	-1.693417	-0.963185
H	-4.573149	-0.303622	-1.397533
C	-3.806729	-3.024232	-3.433052
H	-1.903391	-3.177218	-4.469527
H	-2.869947	-1.773556	-4.925562
H	-5.552428	-2.413335	-2.293971
H	-5.107322	-1.309336	-3.594171
H	-4.373443	-3.599232	-4.175837
H	-3.489563	-3.735058	-2.655270
N	-2.758743	1.529615	-1.063367
H	-3.479752	1.848836	-1.696121
C	-2.503995	2.377916	0.088129
H	-2.172043	1.736430	0.912633
C	-1.440009	3.442858	-0.147243
C	-0.295690	3.479948	0.650898
C	-1.585388	4.390988	-1.165747
C	0.696793	4.435944	0.430230
H	-0.170340	2.745801	1.444805
C	-0.600799	5.350551	-1.385899

H	-2.462649	4.368577	-1.809692
C	0.546506	5.372557	-0.590190
H	1.589170	4.441878	1.050398
H	-0.723404	6.078090	-2.183999
H	1.319801	6.114676	-0.769142
C	-3.807536	3.032487	0.538593
H	-3.583797	3.742124	1.350566
H	-4.248791	3.612135	-0.292367
O	-4.705551	2.042311	0.975469
C	-5.951054	2.579973	1.365337
H	-6.446702	3.085816	0.522688
H	-6.574247	1.747604	1.699796
H	-5.836307	3.299358	2.190225

$TS_{II'}$

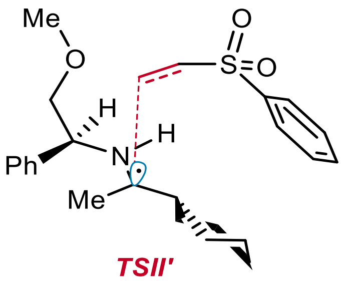

Geometry optimisation:
Sum of electronic and thermal Free Energies = -1650.799123 Hartrees
Thermal correction to Gibbs Free Energy = 0.490078 Hartrees

Single Point Energy:
Energy = -1651.565478 Hartrees

C	1.006874	3.047519	-0.679632
C	1.621222	3.721070	-2.978782
H	1.486023	4.704280	-2.542340
H	2.615403	3.287745	-2.934922
C	0.686882	3.248187	-3.838881
H	-0.291177	3.699323	-3.967743
S	0.951267	1.813417	-4.790157
O	0.317196	2.021556	-6.101595
O	2.372031	1.441733	-4.752493
C	0.047997	0.493416	-3.978065
C	-1.268071	0.708347	-3.565272
C	0.678887	-0.730624	-3.781579
C	-1.948893	-0.317838	-2.916761
H	-1.755441	1.664914	-3.736028
C	-0.016330	-1.755941	-3.141415
H	1.704865	-0.868461	-4.108945
C	-1.322562	-1.548104	-2.703120
H	-2.969054	-0.158205	-2.579882
H	0.470160	-2.712823	-2.975356
H	-1.856092	-2.345322	-2.193108

C	1.914694	3.965192	0.083050
H	1.700056	5.020250	-0.105804
H	1.836672	3.808837	1.170457
H	2.954245	3.785944	-0.205527
C	1.318285	1.560522	-0.698802
C	2.805785	1.218472	-0.858640
C	0.763210	0.871182	0.570025
H	0.782151	1.125053	-1.556909
C	3.040908	-0.294734	-0.888634
H	3.369479	1.639594	-0.013749
H	3.209130	1.669996	-1.771649
C	0.999335	-0.640585	0.546066
H	1.250528	1.307595	1.455566
H	-0.307896	1.082871	0.661290
C	2.480844	-0.975288	0.362031
H	4.114131	-0.498005	-0.987739
H	2.555273	-0.718647	-1.778127
H	0.615112	-1.092680	1.469150
H	0.425674	-1.076366	-0.285268
H	2.622466	-2.061474	0.299325
H	3.041779	-0.631032	1.244123
N	-0.349359	3.305790	-0.707998
H	-0.873305	2.702019	-1.326440
C	-1.058303	4.518119	-0.318398
H	-2.119282	4.247939	-0.399723
C	-0.813435	4.863029	1.149059
C	-1.082332	3.880797	2.109207
C	-0.335903	6.102339	1.579835
C	-0.869968	4.123501	3.462577
H	-1.449159	2.909928	1.783976
C	-0.120459	6.350803	2.937043
H	-0.103783	6.887570	0.865488
C	-0.383526	5.363315	3.882829
H	-1.083798	3.345707	4.191116
H	0.260795	7.319123	3.250783
H	-0.212542	5.555601	4.938628
C	-0.879830	5.699790	-1.274354
H	-1.464285	6.557859	-0.905468
H	0.173639	6.018465	-1.339875
O	-1.351888	5.311311	-2.542748
C	-1.181782	6.320526	-3.515961
H	-0.115550	6.548096	-3.667857
H	-1.603259	5.944257	-4.451126
H	-1.705615	7.244435	-3.229857

$TS_{III'}$

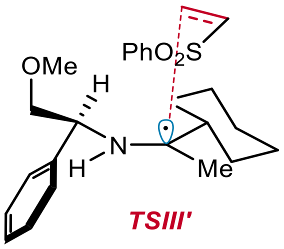

Geometry optimisation:
Sum of electronic and thermal Free Energies = -1650.797867 Hartrees
Thermal correction to Gibbs Free Energy = 0.491748 Hartrees

Single Point Energy:
Energy = -1651.565298 Hartrees

C	-1.007226	-0.087986	-0.345963
C	-2.472514	-1.534288	0.764715
H	-1.745305	-1.536618	1.570590
H	-3.249662	-0.772688	0.783679
C	-2.687589	-2.717211	0.111293
H	-1.997137	-3.555523	0.126862
S	-4.061311	-3.001428	-0.919899
O	-4.230797	-4.459732	-1.024589
O	-5.201796	-2.188102	-0.478853
C	-3.614680	-2.416630	-2.553602
C	-2.899309	-3.2504	-3.411247
C	-3.916796	-1.101183	-2.901225
C	-2.470309	-2.747754	-4.638575
H	-2.681827	-4.274244	-3.121728
C	-3.485447	-0.609770	-4.131821
H	-4.463277	-0.476274	-2.201050
C	-2.757752	-1.429625	-4.995961
H	-1.909916	-3.386531	-5.315079
H	-3.716058	0.414253	-4.413418
H	-2.416817	-1.041311	-5.951684
C	-0.475744	-1.057756	-1.370662
H	0.276973	-0.575448	-2.009480
H	-0.010499	-1.920418	-0.885431
H	-1.271283	-1.429339	-2.022682
C	-0.014802	0.248958	0.763099
C	1.191924	1.059393	0.230010
C	-0.531468	0.923222	2.047560
H	0.381499	-0.731972	1.070071
C	2.300708	1.161894	1.282473
H	0.849196	2.065565	-0.046669
H	1.594421	0.597368	-0.678360
C	0.570929	0.968937	3.110319
H	-0.824251	1.958089	1.837516
H	-1.415328	0.408604	2.441289
C	1.786559	1.746926	2.599812

H	3.124967	1.771422	0.891800
H	2.711108	0.158003	1.468346
H	0.181393	1.429316	4.026330
H	0.8723	-0.056413	3.372348
H	2.584344	1.748418	3.352430
H	1.497395	2.796414	2.439083
N	-1.804155	0.884924	-0.917440
H	-2.280194	0.571850	-1.753232
C	-2.473649	1.977585	-0.229047
H	-2.611362	1.712742	0.824341
C	-1.711444	3.292325	-0.314591
C	-1.807820	4.220831	0.725388
C	-0.943965	3.609542	-1.436822
C	-1.142532	5.443559	0.650097
H	-2.399372	3.980957	1.607844
C	-0.279933	4.832846	-1.518040
H	-0.848846	2.881485	-2.238902
C	-0.375747	5.752857	-0.473814
H	-1.218383	6.152645	1.470281
H	0.317213	5.065538	-2.396034
H	0.146298	6.704081	-0.533866
C	-3.878660	2.166350	-0.796059
H	-4.326021	3.067839	-0.350703
H	-3.832508	2.321557	-1.890180
O	-4.637186	1.024783	-0.482344
C	-5.996202	1.138322	-0.851060
H	-6.102876	1.322602	-1.931709
H	-6.469809	0.187077	-0.600692
H	-6.487852	1.956592	-0.304860

$TS_{IV'}$

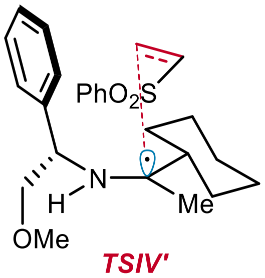

Geometry optimisation:
Sum of electronic and thermal Free Energies = -1650.788660 Hartrees
Thermal correction to Gibbs Free Energy = 0.490601 Hartrees

Single Point Energy:
Energy = -1651.555062 Hartrees

C	2.0271	3.089745	-0.181320
C	2.610398	2.016375	1.795446
H	3.511742	2.622540	1.829498
H	1.738209	2.407384	2.309533

C	2.762421	0.665089	1.620975
H	3.661712	0.203210	1.224042
S	1.475229	-0.473181	1.860004
O	2.082728	-1.772957	2.185123
O	0.472395	0.087159	2.778058
C	0.649681	-0.678204	0.278032
C	1.323810	-1.322959	-0.759710
C	-0.629422	-0.156994	0.094503
C	0.711925	-1.425955	-2.006181
H	2.314803	-1.737712	-0.596136
C	-1.235016	-0.266820	-1.157143
H	-1.141888	0.323090	0.922612
C	-0.562864	-0.892751	-2.206916
H	1.230056	-1.922398	-2.821501
H	-2.232047	0.137098	-1.308809
H	-1.035570	-0.972335	-3.181671
C	2.840455	2.186697	-1.078663
H	2.713055	2.478066	-2.130381
H	3.904896	2.237730	-0.830989
H	2.522663	1.143037	-0.982832
C	2.670464	4.443220	0.078957
C	2.866090	5.270296	-1.214674
C	2.072717	5.317798	1.187165
H	3.690485	4.190137	0.407810
C	3.715250	6.517872	-0.947844
H	1.891714	5.565599	-1.621513
H	3.351117	4.658339	-1.983075
C	2.956374	6.538049	1.458318
H	1.082888	5.678377	0.894327
H	1.930165	4.736620	2.105149
C	3.144248	7.370434	0.187495
H	3.797706	7.109933	-1.867693
H	4.736105	6.204289	-0.682784
H	2.508623	7.148149	2.252020
H	3.938326	6.206455	1.828013
H	3.800068	8.227354	0.384103
H	2.170268	7.780071	-0.120743
N	0.676469	2.871955	-0.357338
H	0.492990	2.008811	-0.846492
C	-0.549282	3.651788	-0.184429
H	-1.328698	2.934974	-0.475535
C	-0.879325	4.048992	1.253159
C	-0.745749	3.093176	2.263182
C	-1.3656	5.314317	1.596144
C	-1.056562	3.396366	3.586157
H	-0.382057	2.098103	2.026419
C	-1.680990	5.622512	2.920241
H	-1.487241	6.088125	0.843328
C	-1.522358	4.667551	3.922360
H	-0.932599	2.634886	4.351810
H	-2.047417	6.616122	3.165132

H	-1.761570	4.909507	4.954470
C	-0.682677	4.800907	-1.187308
H	-1.716318	5.180919	-1.155300
H	-0.014635	5.640037	-0.937067
O	-0.390369	4.307487	-2.470938
C	-0.499581	5.303270	-3.464783
H	0.205426	6.128624	-3.280111
H	-0.258598	4.835031	-4.421692
H	-1.519059	5.714991	-3.510502

Int$_{III}$

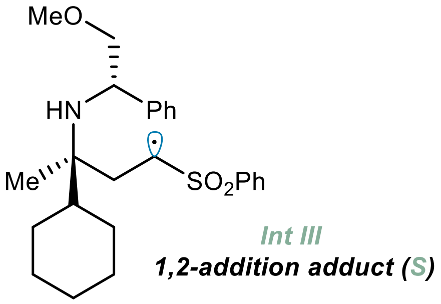

Geometry optimisation:
Sum of electronic and thermal Free Energies = -1650.827978 Hartrees
Thermal correction to Gibbs Free Energy = 0.494214 Hartrees

Single Point Energy:
Energy = -1651.586857 Hartrees

C	-1.291255	0.446074	-0.858063
C	-0.724389	1.085158	-2.153080
H	-0.461508	2.135675	-1.956112
H	0.218652	0.597687	-2.414355
C	-1.661174	1.007481	-3.301962
H	-2.737611	1.132030	-3.196133
S	-1.106996	0.480484	-4.870151
O	-2.242131	0.532944	-5.796229
O	0.125479	1.202117	-5.209656
C	-0.675336	-1.226970	-4.590528
C	-1.682110	-2.191812	-4.634608
C	0.634343	-1.549359	-4.235306
C	-1.364859	-3.508718	-4.314668
H	-2.694710	-1.913864	-4.912615
C	0.938196	-2.870819	-3.915625
H	1.400737	-0.780274	-4.219923
C	-0.059401	-3.845183	-3.951813
H	-2.136433	-4.272018	-4.347621
H	1.954673	-3.137322	-3.641269
H	0.181220	-4.874352	-3.700352
C	-1.612415	-1.030081	-1.150583
H	-2.372634	-1.102180	-1.934500
H	-0.724518	-1.575523	-1.486607

H	-2.004806	-1.533217	-0.260537
C	-0.220782	0.566260	0.281047
C	-0.717866	0.015569	1.626850
C	1.144955	-0.070981	-0.041235
H	-0.034350	1.638490	0.422847
C	0.283962	0.286028	2.752521
H	-0.871644	-1.070682	1.548221
H	-1.685573	0.457641	1.886755
C	2.160130	0.192319	1.077441
H	1.031203	-1.157314	-0.170696
H	1.555567	0.318809	-0.979363
C	1.655206	-0.311159	2.430555
H	-0.098613	-0.120784	3.697221
H	0.382159	1.372881	2.889817
H	3.118392	-0.279763	0.827552
H	2.346575	1.274868	1.142759
H	2.375710	-0.067412	3.221140
H	1.573536	-1.408277	2.399816
N	-2.590852	1.063036	-0.543618
H	-3.077271	0.503251	0.141911
C	-2.876217	2.485840	-0.315461
H	-3.842192	2.468154	0.204344
C	-1.924690	3.256873	0.599913
C	-2.081820	3.139439	1.984864
C	-0.891115	4.067668	0.118328
C	-1.211465	3.775590	2.866470
H	-2.893709	2.530515	2.378803
C	-0.016331	4.709229	0.995271
H	-0.746029	4.201371	-0.950836
C	-0.167530	4.558358	2.372736
H	-1.348655	3.659985	3.938473
H	0.785047	5.327406	0.598975
H	0.516268	5.054417	3.056318
C	-3.160897	3.270465	-1.601938
H	-3.421184	4.305316	-1.324788
H	-2.285220	3.328729	-2.267093
O	-4.234250	2.669167	-2.288859
C	-4.544592	3.347313	-3.487378
H	-3.693149	3.329689	-4.186153
H	-5.391871	2.831536	-3.944901
H	-4.819183	4.395384	-3.294991

Int$_{III'}$

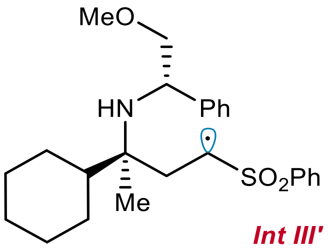

Geometry optimisation:
Sum of electronic and thermal Free Energies = -1650.823712 Hartrees
Thermal correction to Gibbs Free Energy = 0.492549 Hartrees

Single Point Energy:
Energy = -1651.590764 Hartrees

C	-0.801396	0.359820	-0.483401
C	-0.290288	1.007324	-1.792179
H	-0.344778	2.101161	-1.677715
H	0.772254	0.790519	-1.937082
C	-1.077632	0.589884	-2.976137
H	-2.156721	0.447928	-2.926606
S	-0.383687	0.386222	-4.558741
O	-0.971467	1.384280	-5.463396
O	1.076802	0.322639	-4.438852
C	-0.982684	-1.218409	-5.052169
C	-2.259469	-1.323159	-5.605042
C	-0.187655	-2.340544	-4.823245
C	-2.748074	-2.585101	-5.932357
H	-2.853234	-0.431142	-5.783236
C	-0.687716	-3.596730	-5.157816
H	0.803646	-2.229443	-4.394019
C	-1.964284	-3.718197	-5.707228
H	-3.738298	-2.683618	-6.367114
H	-0.079948	-4.480339	-4.986534
H	-2.351034	-4.700759	-5.962623
C	0.123169	0.822285	0.661569
H	0.284276	1.903406	0.639279
H	-0.309110	0.571058	1.635163
H	1.103199	0.337757	0.584820
C	-0.771385	-1.200261	-0.618887
C	0.549018	-1.779902	-1.156462
C	-1.137272	-1.933540	0.685457
H	-1.552173	-1.440651	-1.358017
C	0.446449	-3.293615	-1.373241
H	1.366075	-1.573815	-0.449564
H	0.829250	-1.307878	-2.104202
C	-1.252297	-3.446367	0.473189
H	-0.361458	-1.741632	1.440657
H	-2.081018	-1.564719	1.106449
C	0.044615	-4.026461	-0.092943

H	1.400445	-3.678887	-1.754949
H	-0.306744	-3.490404	-2.150652
H	-1.508972	-3.935976	1.420574
H	-2.077002	-3.651485	-0.225410
H	-0.069126	-5.100253	-0.286343
H	0.844967	-3.919765	0.654660
N	-2.225631	0.701118	-0.303580
H	-2.588594	0.180624	0.481943
C	-2.798177	2.054410	-0.264465
H	-3.770582	1.902378	0.221698
C	-2.054550	3.077263	0.593010
C	-2.137028	2.959496	1.985208
C	-1.266974	4.101667	0.060012
C	-1.430202	3.815814	2.823943
H	-2.752671	2.171318	2.415768
C	-0.555674	4.965172	0.895488
H	-1.186788	4.237232	-1.015816
C	-0.628183	4.820822	2.279261
H	-1.505224	3.700672	3.902193
H	0.057470	5.750121	0.460321
H	-0.070312	5.489509	2.929334
C	-3.158305	2.614758	-1.645573
H	-3.653815	3.589544	-1.504433
H	-2.275387	2.792994	-2.277714
O	-4.029230	1.719783	-2.298717
C	-4.351548	2.146735	-3.605606
H	-3.451720	2.198027	-4.239245
H	-5.044276	1.412643	-4.024391
H	-4.835681	3.134821	-3.597056

X-ray Crystallography Data

The absolute configurations of the α-tertiary amine derivatives made using (R)-2-methoxy-1-phenylethan-1-amine CAT reagent were determined to be (R). The absolute stereochemistry of all other products in the scope have been assigned in analogy to the two compounds shown below.

(R)-5-methyl-5-(tetrahydro-2*H*-pyran-4-yl)pyrrolidin-2-one, 6

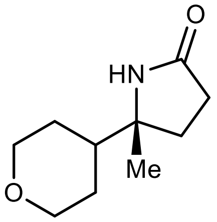

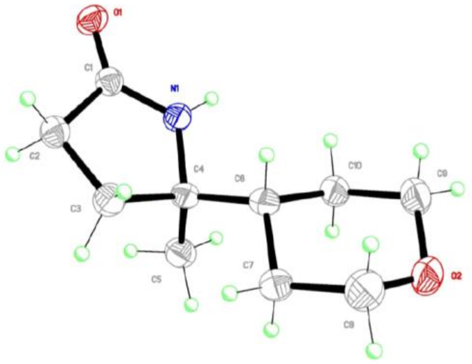

Crystal growth	Slow evaporation from ethyl acetate	
Deposition number	CCDC 2172068	
Identification code	MG_B2_0047	
Chemical formula	$C_{10}H_{17}NO_2$	
Formula weight	183.24	
Temperature	180(2) K	
Crystal system	monoclinic	
Space group	C_2	
Unit cell dimensions	a = 10.5391(6) Å	α = 90 °
	b = 7.4765(4) Å	β = 95.898(2) °
	c = 12.6630(7) Å	γ = 90 °
Volume	992.51(10) $Å^3$	
Z	4	
Density (calculated)	1.226 Mg/m^3	
F(000)	400	
Absorption coefficient	0.682 mm^{-1}	
Crystal size	0.35 x 0.30 x 0.30 mm^3	
ϴ range for data collection	14.06 to 133.08 °	
Index ranges	-12≤h≤12, -8≤k≤8, -15≤l≤15	
Reflections collected	1689	
Completeness to max 2ϴ	97.9%	
Absorption correction	Multi-scan	
Max. and min. transmission	0.6137 and 0.7528	
Refinement method	Full-matrix least-squares on F^2	
Data / restraints / parameter	1689 / 1 / 124	
Final R values [1 > 2s(I)]	R1 = 0.0339, wR2 = 0.0862	
R indices (all data)	R1 = 0.0340, wR2 = 0.0862	
Goodness-of-fit on F2	1.070	
Largest diff. peak and hole	0.168 and -0.117 e $Å^{-3}$	

***tert*-Butyl (R)-4-(2,3-dihydro-1*H*-inden-2-yl)-4-(4-nitrobenzamido)pentanoate, benzoylated 3j**

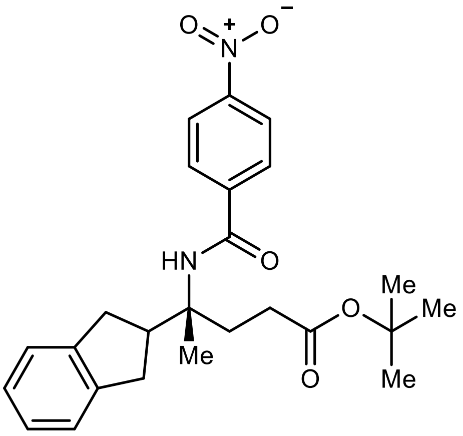

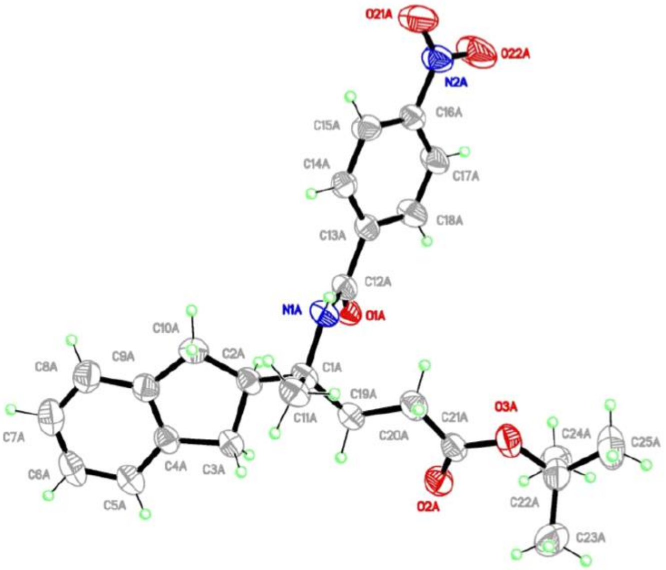

Crystal growth	slow evaporation from ethyl acetate	
Deposition number	CCDC 2172070	
Identification code	MG_B2_0048	
Chemical formula	$C_{25}H_{30}N_2O_5$	
Formula weight	438.51	
Temperature	180(2) K	
Crystal system	monoclinic	
Space group	C_2	
Unit cell dimensions	a = 18.8100(11) Å	α = 90 °
	b = 6.7893(4) Å	β = 93.049(3) °
	c = 37.227(2) Å	γ = 90 °
Volume	4747.4(5) Å^3	
Z	8	
Density (calculated)	1.227 Mg/m^3	
F(000)	1872	
Absorption coefficient	0.696 mm^{-1}	
Crystal size	0.30 x 0.10 x 0.02 mm^3	
Θ range for data collection	4.75 to 133.64 °	
Index ranges	-22≤h≤22, -8≤k≤7, -44≤l≤44	
Reflections collected	8296	
Completeness to max 2Θ	99.2%	
Absorption correction	Multi-scan	
Max. and min. transmission	0.6208 and 0.7528	
Refinement method	Full-matrix least-squares on F^2	
Data / restraints / parameter	8296 / 1 / 593	
Final R values [1 > 2s(I)]	R1 = 0.0402, wR2 = 0.1006	
R indices (all data)	R1 = 0.0456, wR2 = 0.1051	
Goodness-of-fit on F2	1.047	
Largest diff. peak and hole	0.206 and -0.131 e Å^{-3}	

References

1. Flodén, N. J.; Trowbridge, A. D.; Willcox, D.; Walton, S. M.; Kim, Y.; Gaunt, M. J.; Streamlined Synthesis of C(sp3)-Rich N-Heterospirocycles Enabled by Visible-Light-Mediated Photocatalysis, *J. Am. Chem. Soc.*, **2019**, *141*, 8426–8430.
2. Mosa, F.; Thirsk, C.; Vaultier, M.; Maw, G.; Whiting, A.; Lettan, R. B.; Wipf, P.; High-yielding, Large-scale Synthesis of N-Protected-β-aminonitriles: *tert*-Butyl (1r)-2-Cyano-1-phenylethylcarbamate, *Org. Synth.*, **2008**, *85*, 219.
3. Woźniak, Ł.; Rajkiewicz, A. A.; Monsigny, L.; Kajetanowicz, A.; Grela, K.; Preparation of Functionalized α,β-Unsaturated Sulfonamides via Olefin Cross-Metathesis, *Org. Lett.*, **2020**, *22*, 4970–4973.
4. Gaussian 09, Revision D.01, Frisch, M. J.; Trucks, G. W.; Schlegel, H. B.; Scuseria, G. E.; Robb, M. A.; Cheeseman, J. R.; Scalmani, G.; Barone, V.; Mennucci, B.; Petersson, G. A.; Nakatsuji, H.; Caricato, M.; Li, X.; Hratchian, H. P.; Izmaylov, A. F.; Bloino, J.; Zheng, G.; Sonnenberg, J. L.; Hada, M.; Ehara, M.; Toyota, K.; Fukuda, R.; Hasegawa, J.; Ishida, M.; Nakajima, T.; Honda, Y.; Kitao, O.; Nakai, H.; Vreven, T.; Montgomery, J. A.; Peralta, J. E.; Ogliaro, F.; Bearpark, M.; Heyd, J. J.; Brothers, E.; Kudin, K. N.; Staroverov, V. N.; Kobayashi, R.; Normand, J.; Raghavachari, K.; Rendell, A.; Burant, J. C.; Iyengar, S. S.; Tomasi, J.; Cossi, M.; Rega, N.; Millam, J. M.; Klene, M.; Knox, J. E.; Cross, J. B.; Bakken, V.; Adamo, C.; Jaramillo, J.; Gomperts, R.; Stratmann, R. E.; Yazyev, O.; Austin, A. J.; Cammi, R.; Pomelli, C.; Ochterski, J. W.; Martin, R. L.; Morokuma, K.; Zakrzewski, V. G.; Voth, G. A.; Salvador, P.; Dannenberg, J. J.; Dapprich, S.; Daniels, A. D.; Farkas, Ö.; Foresman, J. B.; Ortiz, J. V.; Cioslowski, J.; Fox, D. J.; *Gaussian, Inc*., Wallingford CT, **2009**.
5. Da Chai, J.; Head-Gordon, M.; Long-range corrected hybrid density functionals with damped atom-atom dispersion corrections, *Phys. Chem. Chem. Phys.*, **2008**, *10*, 6615–6620.
6. Petersson, G. A.; Bennett, A.; Tensfeldt, T. G.; Al-Laham, M. A.; Shirley, W. A.; Mantzaris, J.; A complete basis set model chemistry. I. The total energies of closed-shell atoms and hydrides of the first-row elements, *J. Chem. Phys.*, **1988**, *89*, 2193–2218.
7. Petersson, G. A.; Al-Laham, M. A.; A complete basis set model chemistry. II. Open-shell systems and the total energies of the first-row atoms, *J. Chem. Phys.*, **1991**, *94*, 6081–6090.
8. Miertuš, S.; Scrocco, E.; Tomasi, J.; Electrostatic interaction of a solute with a continuum. A direct utilizaion of AB initio molecular potentials for the prevision of solvent effects, *Chem. Phys.*, **1981**, *55*, 117–129.
9. Miertus̃, S.; Tomasi, J.; Approximate evaluations of the electrostatic free energy and internal energy changes in solution processes, *Chem. Phys.*, **1982**, *65*, 239–245.
10. Krishnan, R.; Binkley, J. S.; Seeger, R.; Pople, J. A.; Self-consistent molecular orbital methods. XX. A basis set for correlated wave functions, *J. Chem. Phys.*, **1980**, *72*, 650–654.
11. Zhang, G.; Konstantinov, I. A.; Arturo, S. G.; Yu, D.; Broadbelt, L. J.; Assessment of a cost-effective approach to the calculation of kinetic and thermodynamic properties of methyl methacrylate homopolymerization: A comprehensive theoretical study, *J. Chem. Theory Comput.*, **2014**, *10*, 5668–5676.
12. Shatskiy, A.; Axelsson, A.; Stepanova, E. V.; Liu, J. Q.; Temerdashev, A. Z.; Kore, B. P.; Blomkvist, B.; Gardner, J. M.; Dinér, P.; Kärkäs, M. D.; Stereoselective synthesis of unnatural α-amino acid derivatives through photoredox catalysis, *Chem. Sci.*, **2021**, *12*, 5430–5437.
13. Marenich, A. V.; Cramer, C. J.; Truhlar, D. G.; Universal solvation model based on solute electron density and on a continuum model of the solvent defined by the bulk dielectric constant and atomic surface tensions, *J. Phys. Chem. B*, **2009**, *113*, 6378–6396.

Chiral HPLC and SFC Data

***tert*-butyl (*R*)-4-cyclobutyl-4-(4-nitrobenzamido)pentanoate, benzoylated 3b**

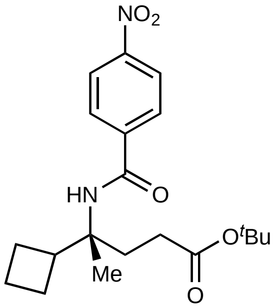

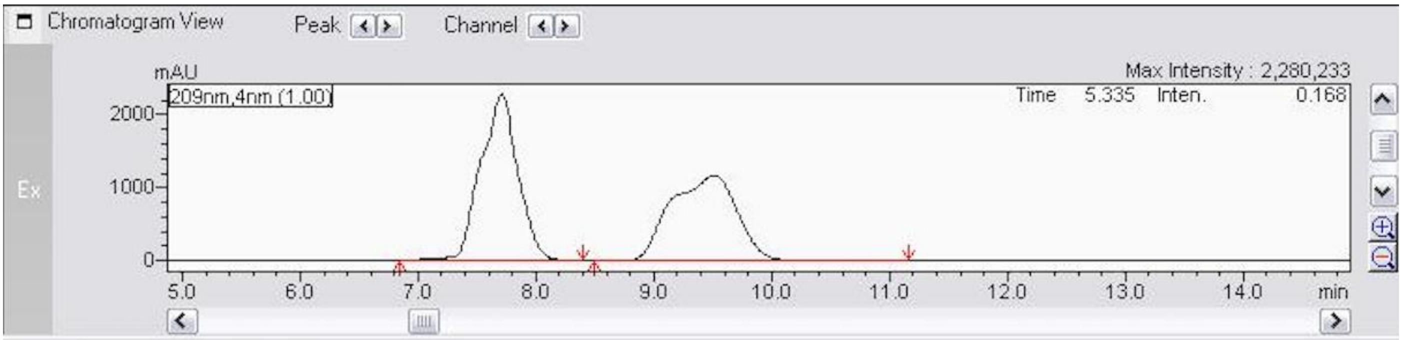

Results View - Peak Table

Peak Table | Compound | Group | Calibration Curve

Peak#	Ret. Time	Area	Height	Area%
1	7.713	50605321	2279450	52.520
2	9.509	45748382	1174028	47.480
Total		96353703	3453479	100.000

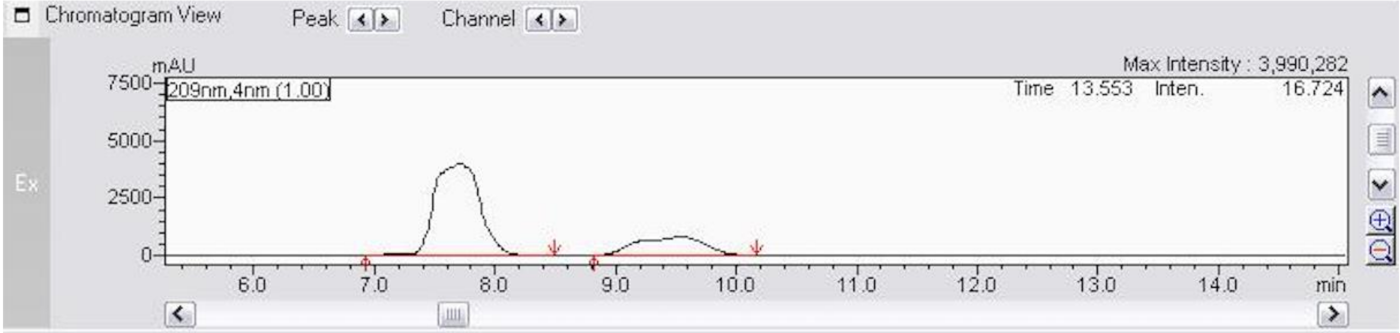

Results View - Peak Table

Peak Table | Compound | Group | Calibration Curve

Peak#	Ret. Time	Area	Height	Area%
1	7.696	104185365	3975204	76.723
2	9.543	31608135	808798	23.277
Total		135793500	4784002	100.000

***tert*-butyl (*R*)-4-cyclohexyl-4-(4-nitrobenzamido)pentanoate, benzoylated 3c**

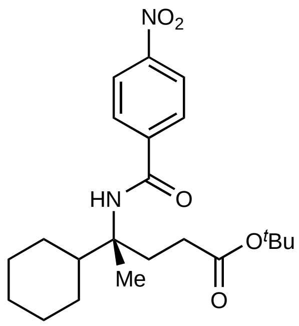

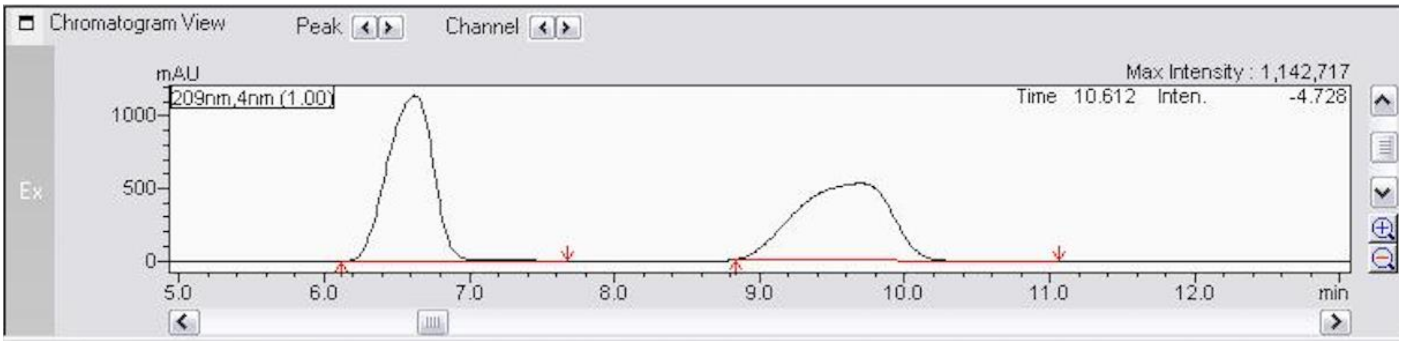

Results View - Peak Table

Peak Table | Compound | Group | Calibration Curve

Peak#	Ret. Time	Area	Height	Area%
1	6.624	25133548	1146075	51.058
2	9.696	24091591	531813	48.942
Total		49225139	1677888	100.000

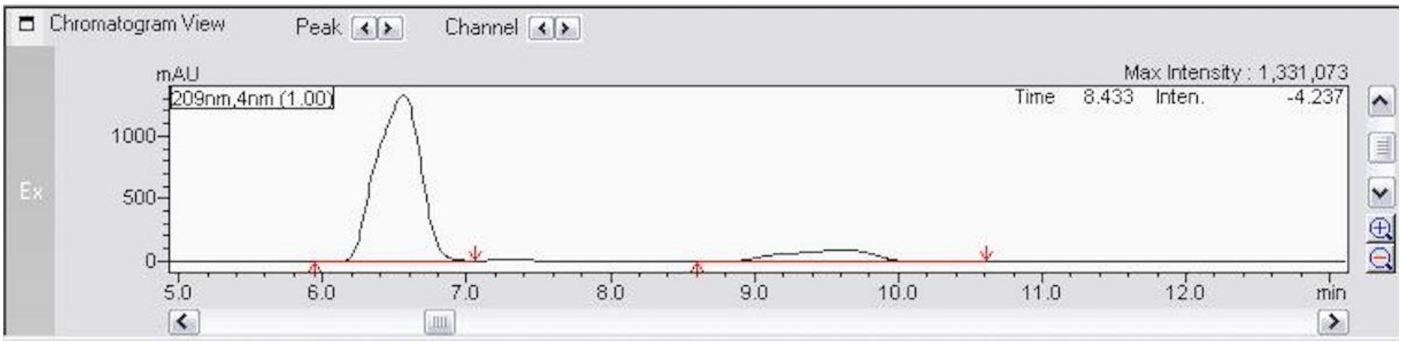

Results View - Peak Table

Peak Table | Compound | Group | Calibration Curve

Peak#	Ret. Time	Area	Height	Area%
1	0.067	2574	1123	0.008
2	6.563	28046106	1330032	87.313
3	9.628	4072759	92490	12.679
Total		32121439	1423644	100.000

***tert*-butyl (*R*)-4,5-dimethyl-4-(4-nitrobenzamido)hexanoate, benzoylated 3d**

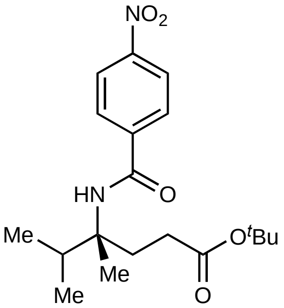

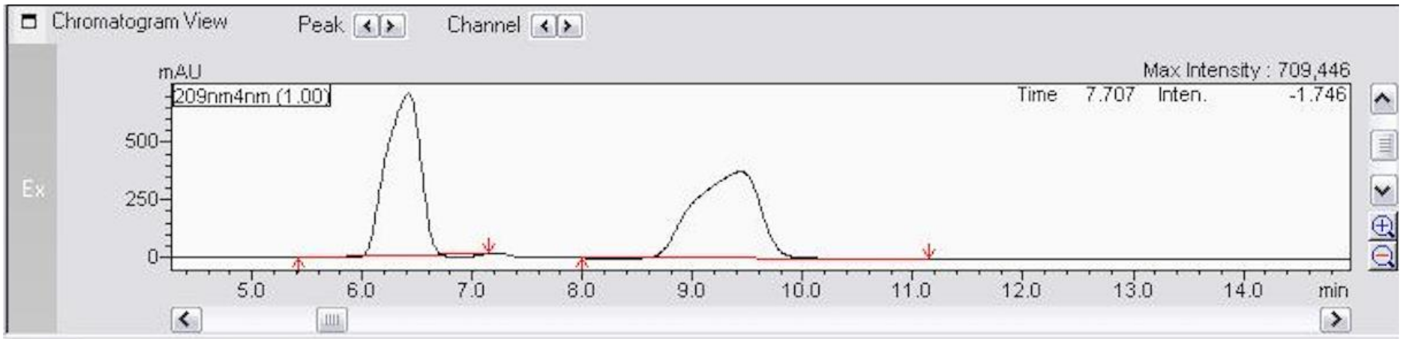

Results View - Peak Table

Peak Table | Compound | Group | Calibration Curve

Peak#	Ret. Time	Area	Height	Area%
1	6.422	14714449	701033	48.445
2	9.438	15659210	377241	51.555
Total		30373659	1078274	100.000

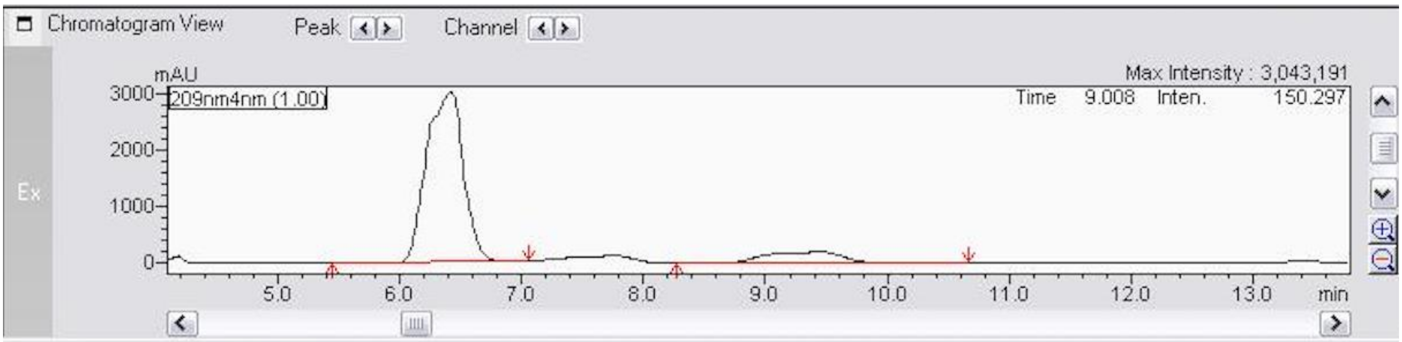

Results View - Peak Table

Peak Table | Compound | Group | Calibration Curve

Peak#	Ret. Time	Area	Height	Area%
1	6.418	62238159	3006628	88.305
2	9.439	8242717	199742	11.695
Total		70480875	3206370	100.000

***tert*-butyl (*R*)-4-cycloheptyl-4-(4-nitrobenzamido)pentanoate, benzoylated 3e**

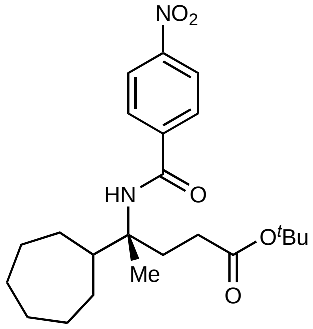

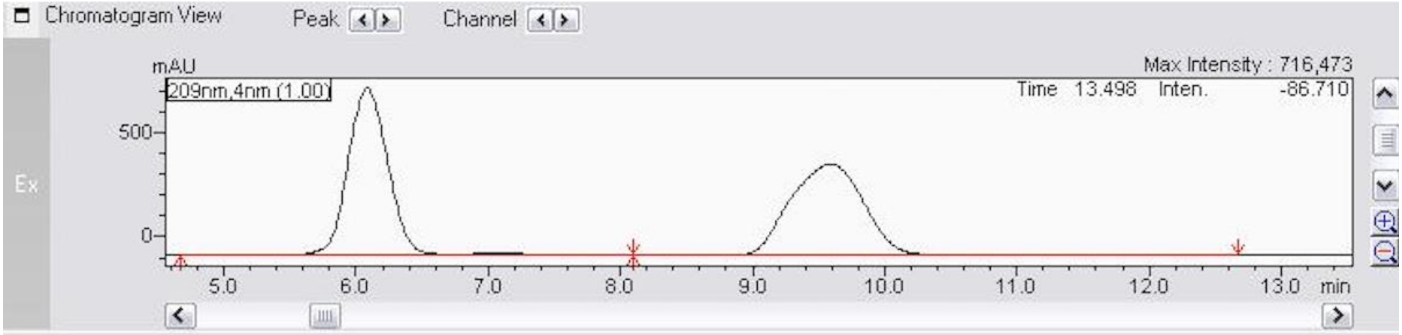

Results View - Peak Table

Peak Table | Compound | Group | Calibration Curve

Peak#	Ret. Time	Area	Height	Area%
1	6.086	16858014	802602	50.322
2	9.588	16642083	438991	49.678
Total		33500097	1241593	100.000

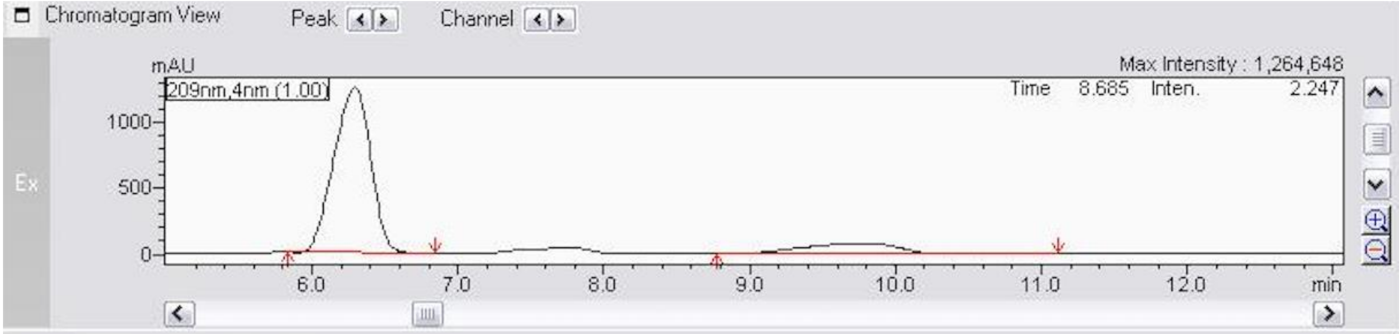

Results View - Peak Table

Peak Table | Compound | Group | Calibration Curve

Peak#	Ret. Time	Area	Height	Area%
1	6.292	21926965	1251802	85.483
2	9.805	3723722	79233	14.517
Total		25650687	1331035	100.000

tert-butyl (_R_)-4-(4,4-dimethylcyclohexyl)-4-(4-nitrobenzamido)pentanoate, benzoylated 3f

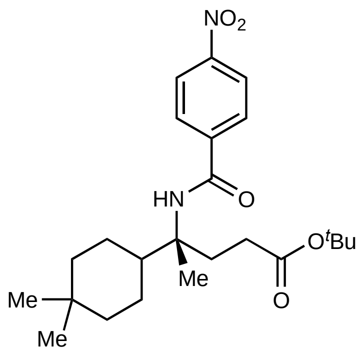

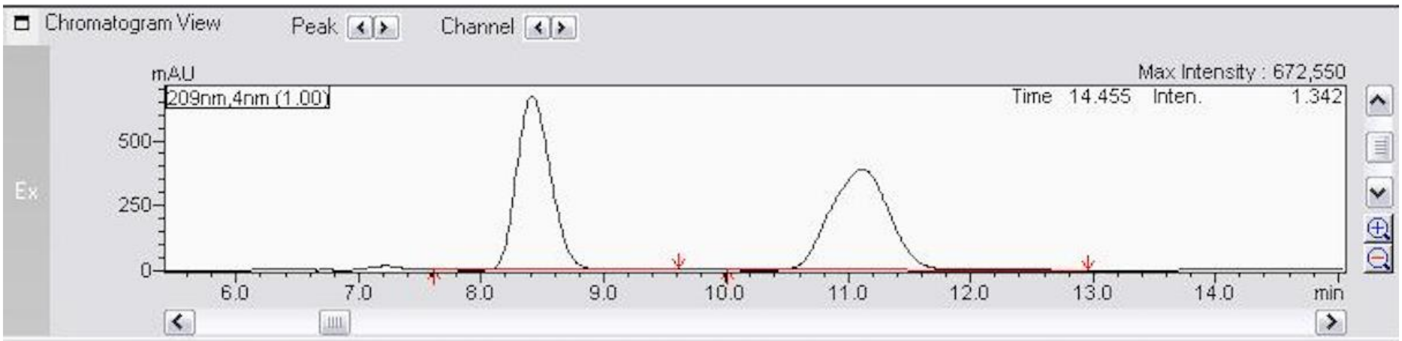

Results View - Peak Table

Peak Table | Compound | Group | Calibration Curve

Peak#	Ret. Time	Area	Height	Area%
1	8.417	12872098	670911	49.873
2	11.117	12937538	389188	50.127
Total		25809636	1060099	100.000

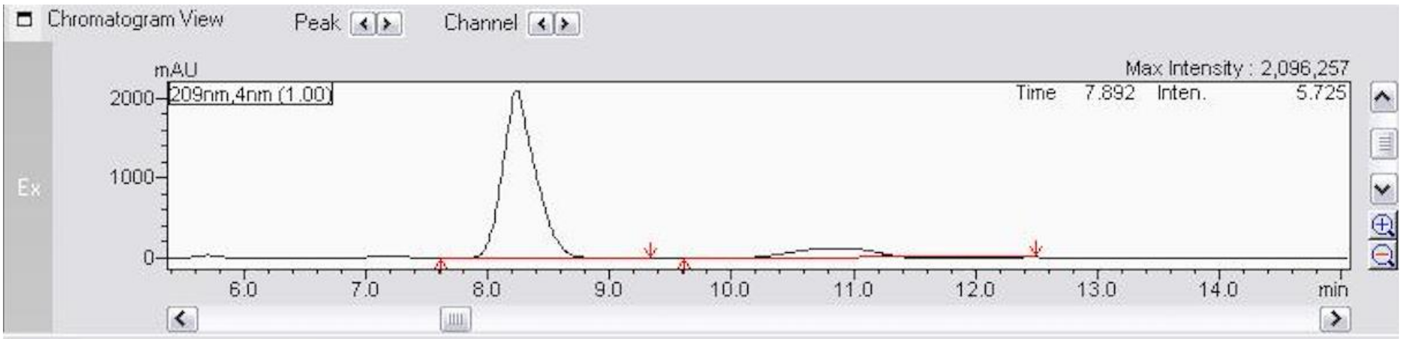

Results View - Peak Table

Peak Table | Compound | Group | Calibration Curve

Peak#	Ret. Time	Area	Height	Area%
1	8.238	38575341	2095695	88.384
2	10.851	5069600	120932	11.616
Total		43644940	2216627	100.000

tert-butyl (*R*)-4-(4-nitrobenzamido)-4-(tetrahydro-2H-thiopyran-4-yl)pentanoate, benzoylated 3g

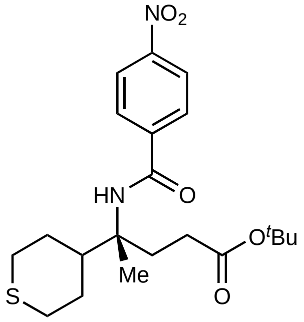

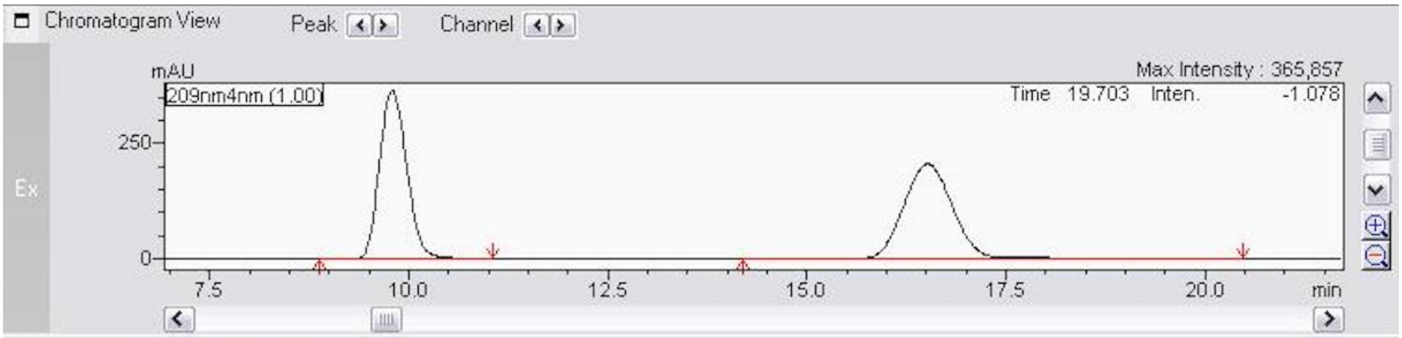

Peak#	Ret. Time	Area	Height	Area%
1	9.792	8678349	365844	49.536
2	16.517	8840838	205686	50.464
Total		17519187	571531	100.000

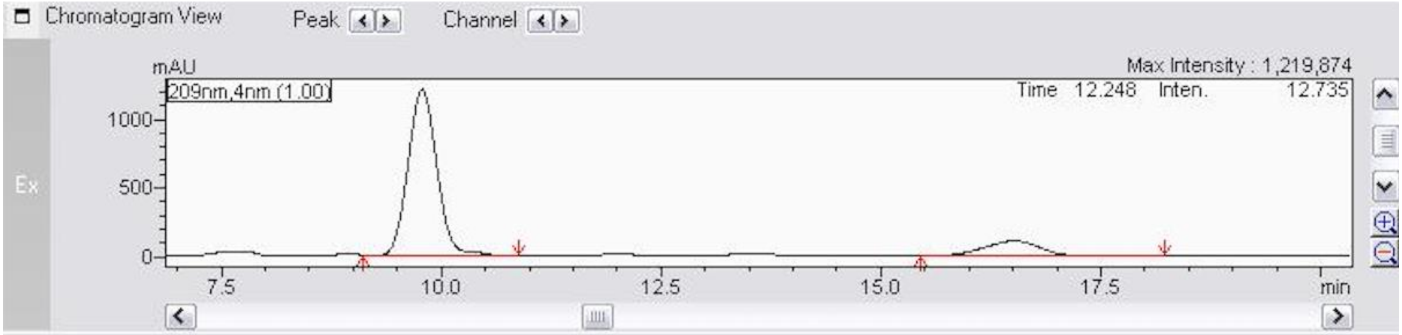

Peak#	Ret. Time	Area	Height	Area%
1	9.788	27580988	1211802	84.091
2	16.516	5136217	111286	15.660
3	34.481	81675	1737	0.249
Total		32798880	1324825	100.000

tert-butyl (R)-4-(5-(tert-butoxy)-2-(4-nitrobenzamido)-5-oxopentan-2-yl)piperidine-1-carboxylate, benzoylated 3h

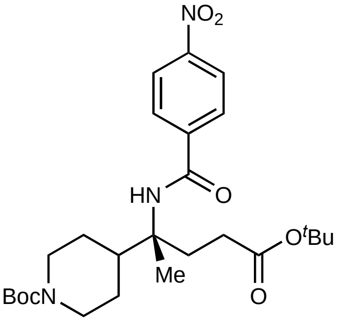

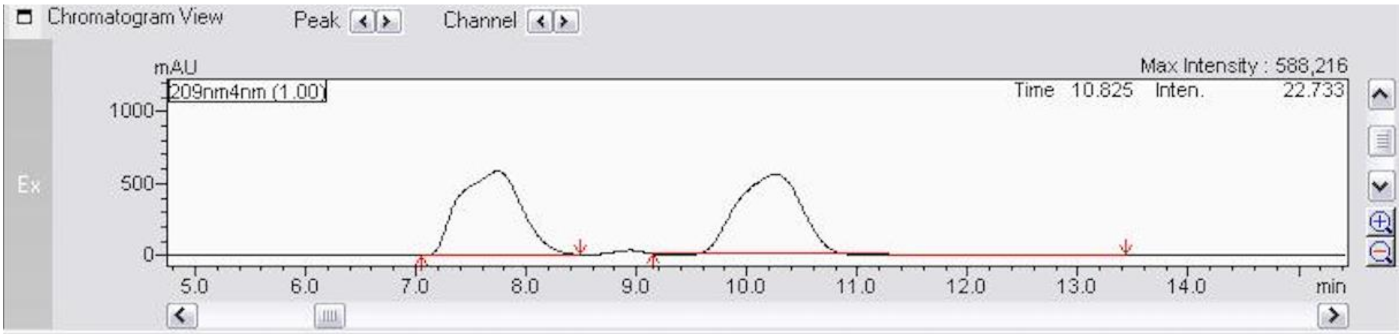

Results View - Peak Table

Peak Table | Compound | Group | Calibration Curve

Peak#	Ret. Time	Area	Height	Area%
1	7.744	22889357	584372	50.989
2	10.257	22001483	558001	49.011
Total		44890840	1142373	100.000

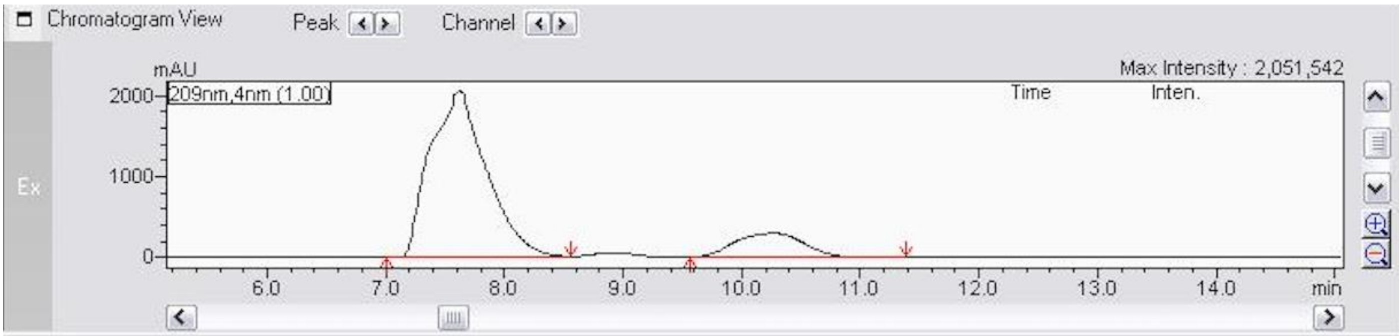

Results View - Peak Table

Peak Table | Compound | Group | Calibration Curve

Peak#	Ret. Time	Area	Height	Area%
1	7.622	67540292	2043557	85.226
2	10.272	11708415	294588	14.774
Total		79248708	2338145	100.000

***tert*-butyl (*R*)-4-(4-nitrobenzamido)-4-(1,4-dioxaspiro[4.5]decan-8-yl)pentanoate, benzoylated 3i**

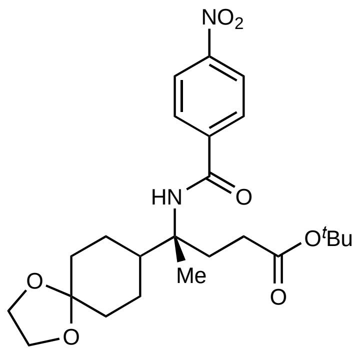

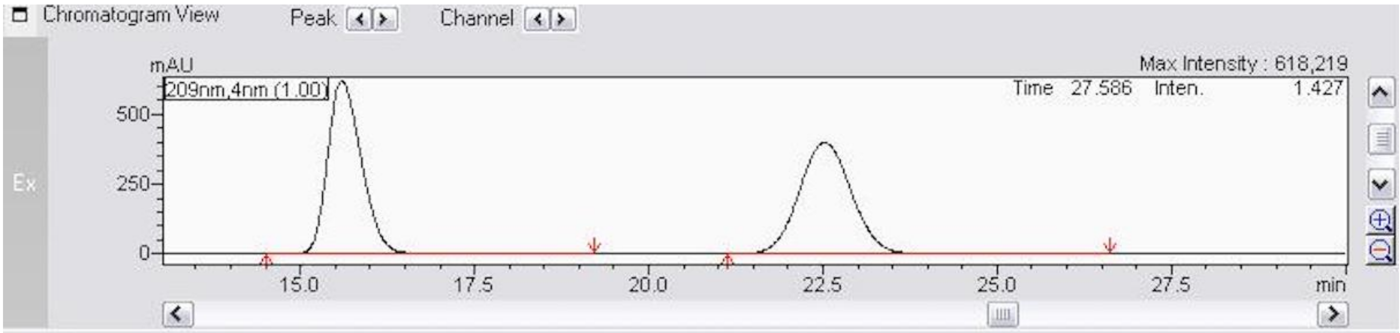

Results View - Peak Table

Peak Table | Compound | Group | Calibration Curve

Peak#	Ret. Time	Area	Height	Area%
1	15.598	20498063	615136	49.679
2	22.523	20762716	396273	50.321
Total		41260779	1011410	100.000

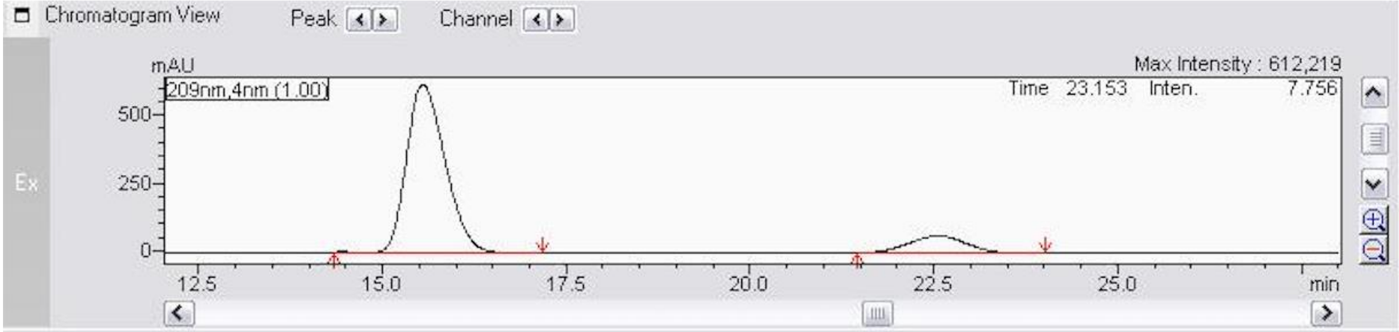

Results View - Peak Table

Peak Table | Compound | Group | Calibration Curve

Peak#	Ret. Time	Area	Height	Area%
1	15.552	22414098	617182	86.377
2	22.551	3535040	63755	13.623
Total		25949138	680937	100.000

***tert*-butyl (*R*)-4-(2,3-dihydro-1*H*-inden-2-yl)-4-(4-nitrobenzamido)pentanoate, benzoylated 3j**

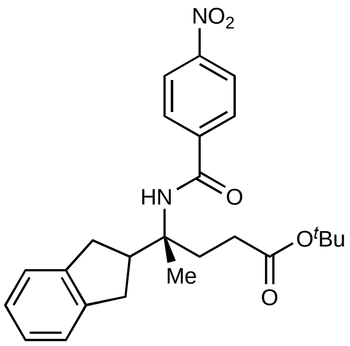

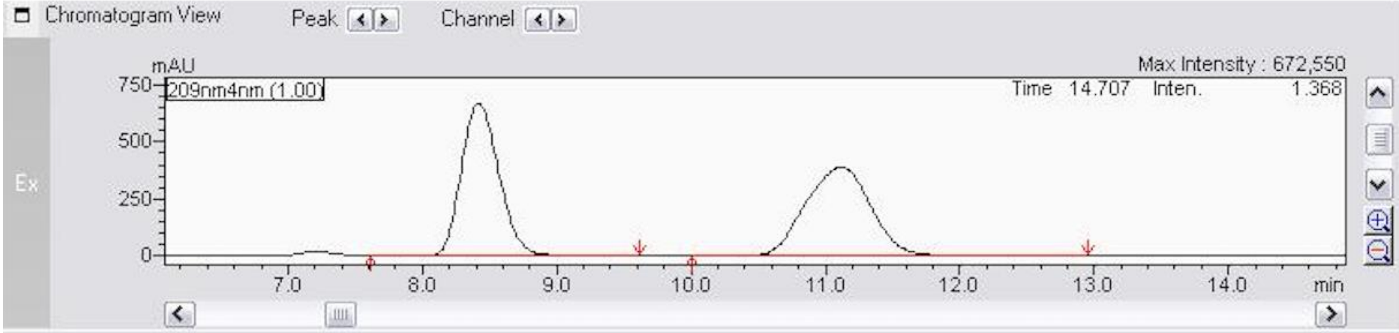

Results View - Peak Table

Peak Table | Compound | Group | Calibration Curve

Peak#	Ret. Time	Area	Height	Area%
1	8.417	12872098	670911	49.873
2	11.117	12937538	389188	50.127
Total		25809636	1060099	100.000

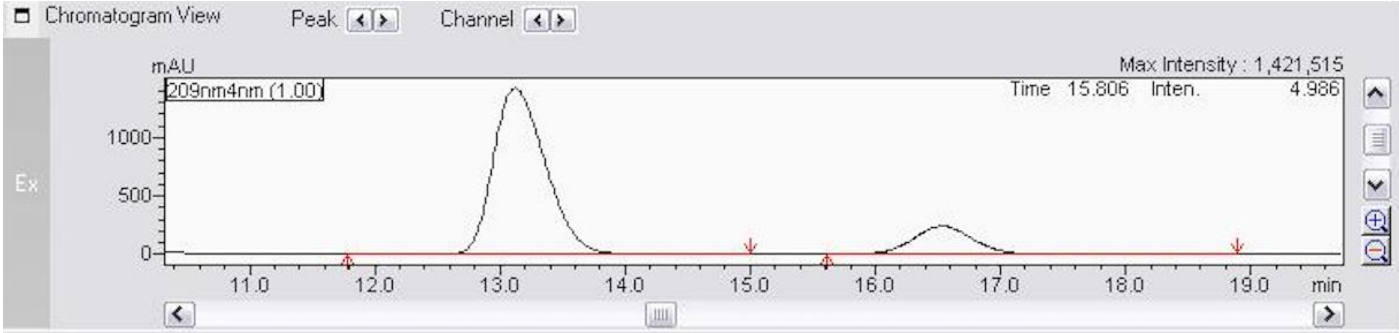

Results View - Peak Table

Peak Table | Compound | Group | Calibration Curve

Peak#	Ret. Time	Area	Height	Area%
1	13.121	41597930	1418999	83.861
2	16.536	8005550	240960	16.139
Total		49603480	1659958	100.000

4-nitro-*N*-(4-(phenylsulfonyl)-2-(tetrahydro-*2H*-pyran-4-yl)butan-2-yl)benzamide, benzoylated 3k

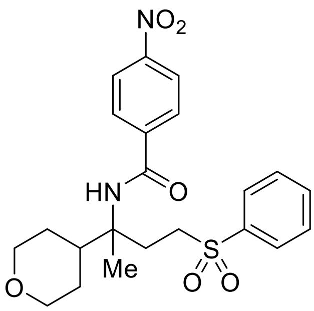

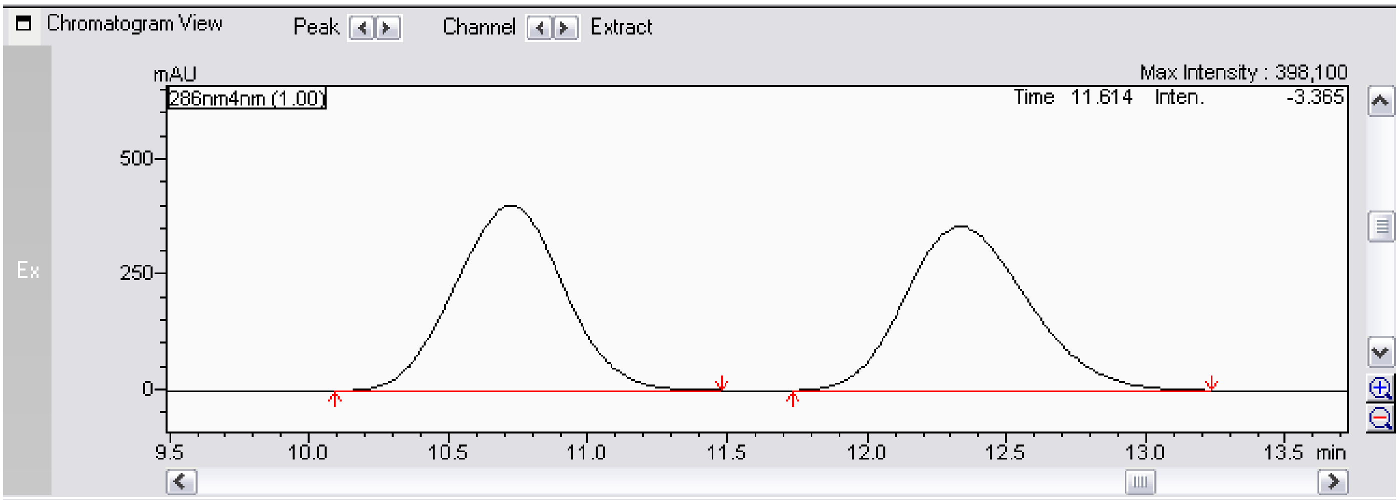

Results View - Peak Table

Peak Table | Compound | Group | Calibration Curve

Peak#	Ret. Time	Area	Height	Area%
1	10.724	10919001	401311	49.981
2	12.335	10927110	355606	50.019
Total		21846111	756917	100.000

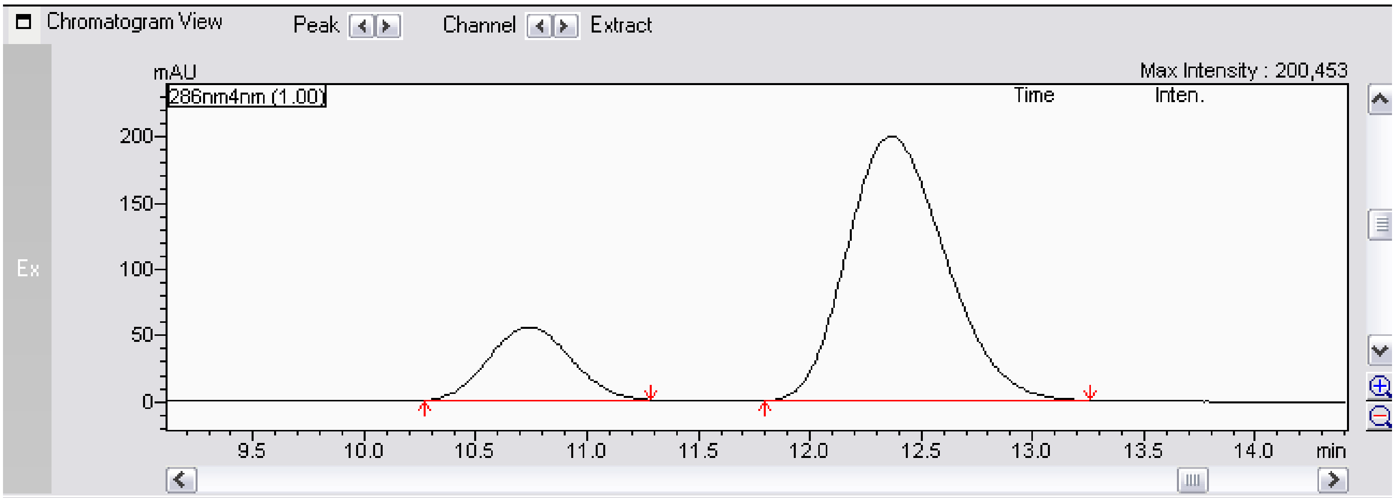

Results View - Peak Table

Peak Table | Compound | Group | Calibration Curve

Peak#	Ret. Time	Area	Height	Area%
1	10.738	1418397	55221	18.837
2	12.365	6111317	199435	81.163
Total		7529714	254656	100.000

Diethyl (3-(4-nitrobenzamido)-3-(tetrahydro-*2H*-pyran-4-yl)butyl)phosphonate, benzoylated 3l

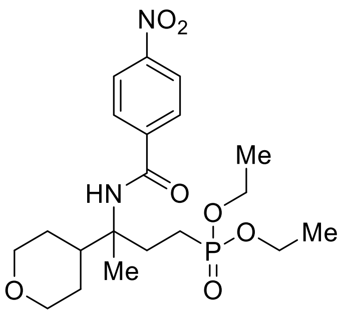

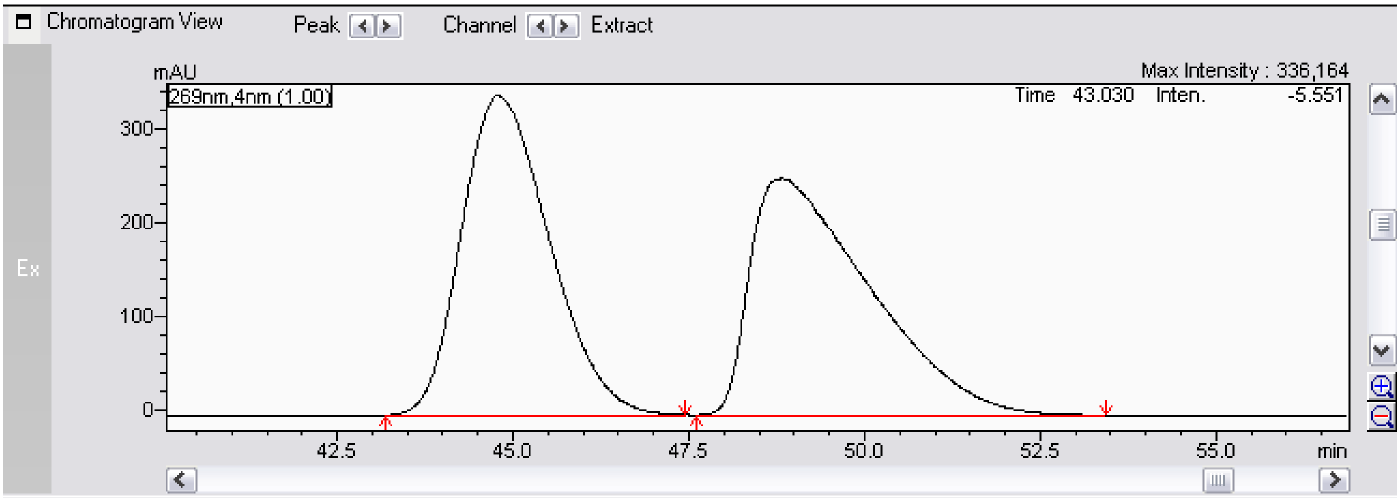

Results View - Peak Table

Peak Table | Compound | Group | Calibration Curve

Peak#	Ret. Time	Area	Height	Area%
1	44.776	29720544	341716	49.835
2	48.824	29916779	252599	50.165
Total		59637323	594315	100.000

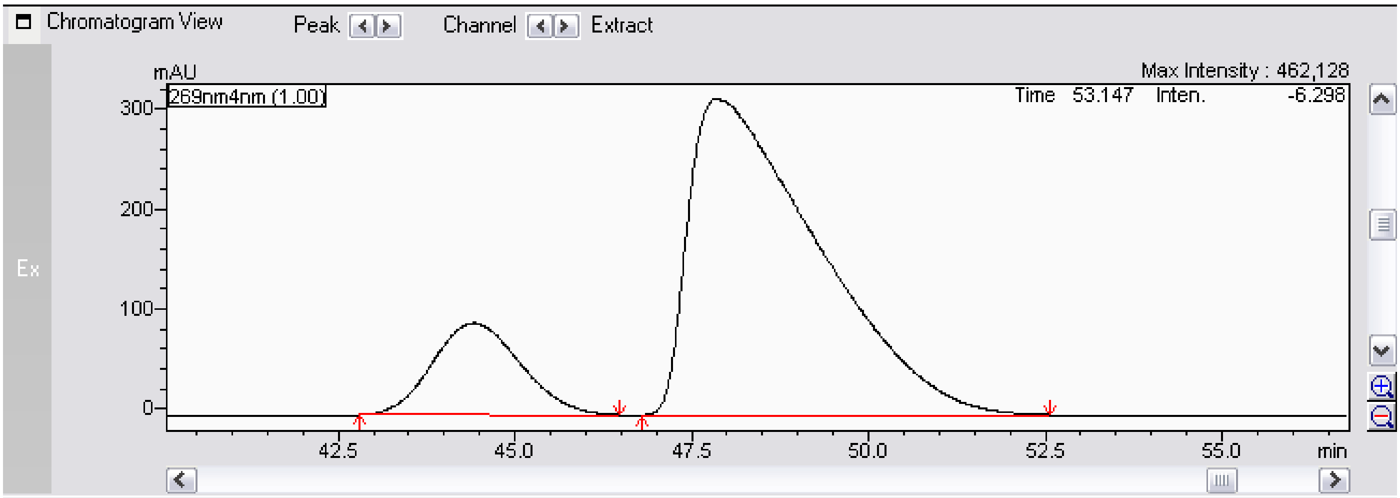

Results View - Peak Table

Peak Table | Compound | Group | Calibration Curve

Peak#	Ret. Time	Area	Height	Area%
1	44.432	7944029	91875	16.529
2	47.832	40118325	316917	83.471
Total		48062355	408792	100.000

_N_-(4-cyano-2-(tetrahydro-_2H_-pyran-4-yl)butan-2-yl)-4-nitrobenzamide, benzoylated 3m

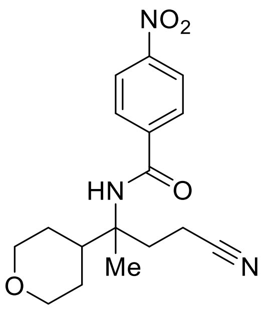

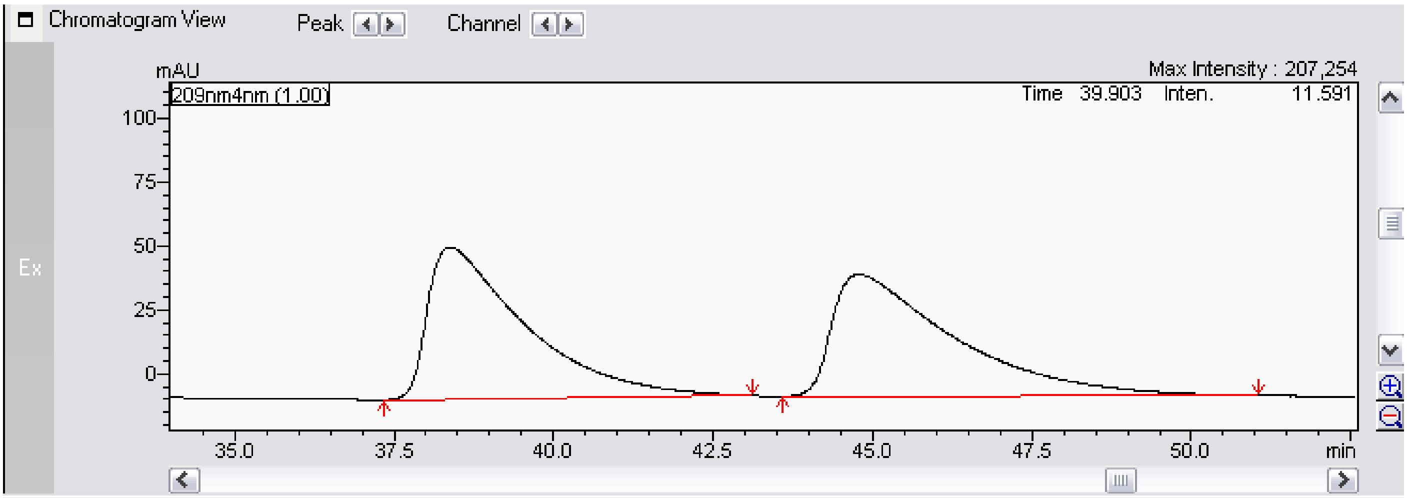

Results View - Peak Table

Peak Table | Compound | Group | Calibration Curve

Peak#	Ret. Time	Area	Height	Area%
1	38.379	6290371	59368	50.077
2	44.762	6270977	47616	49.923
Total		12561348	106984	100.000

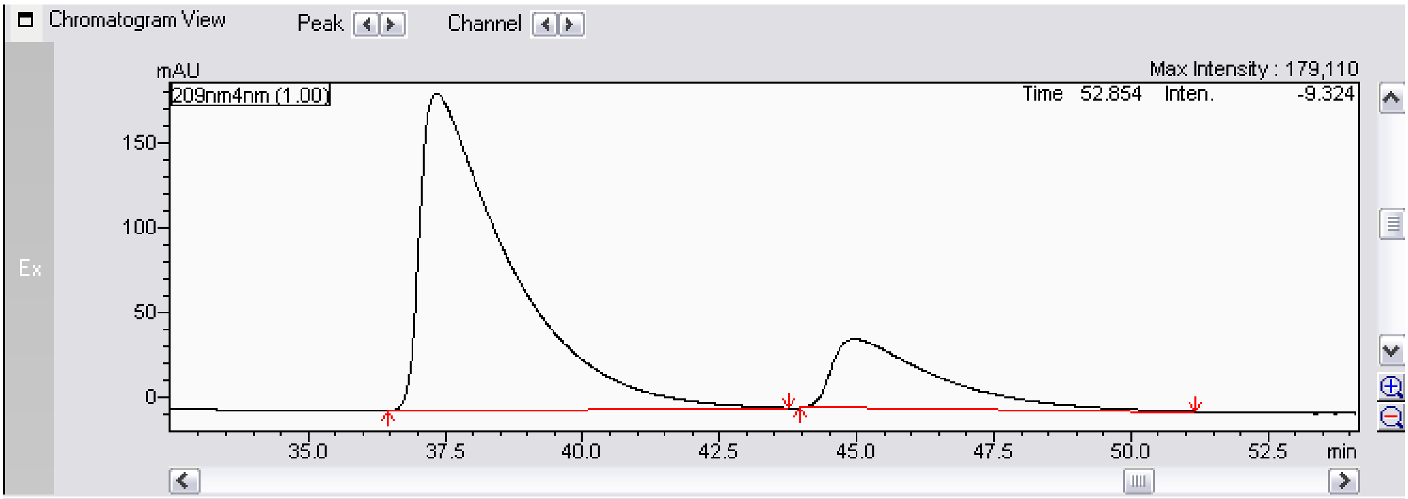

Results View - Peak Table

Peak Table | Compound | Group | Calibration Curve

Peak#	Ret. Time	Area	Height	Area%
1	37.347	20917930	186813	79.665
2	44.985	5339284	40680	20.335
Total		26257215	227493	100.000

***tert*-butyl 4-(4-nitrobenzamido)-4-(tetrahydro-*2H*-pyran-4-yl)pentanoate, benzoylated 3n**

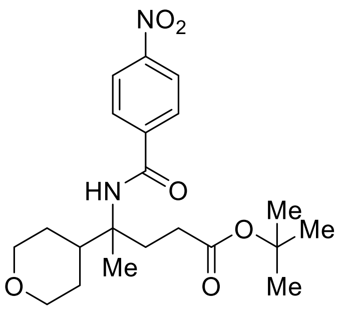

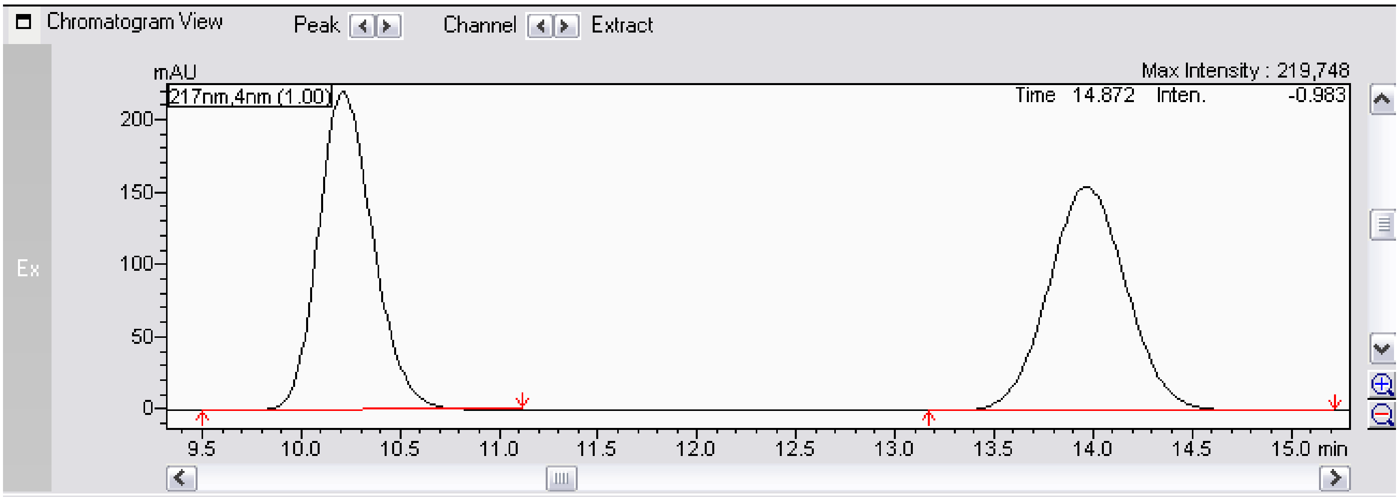

Results View - Peak Table

Peak Table | Compound | Group | Calibration Curve

Peak#	Ret. Time	Area	Height	Area%
1	10.210	4240418	220460	49.835
2	13.969	4268535	155051	50.165
Total		8508953	375511	100.000

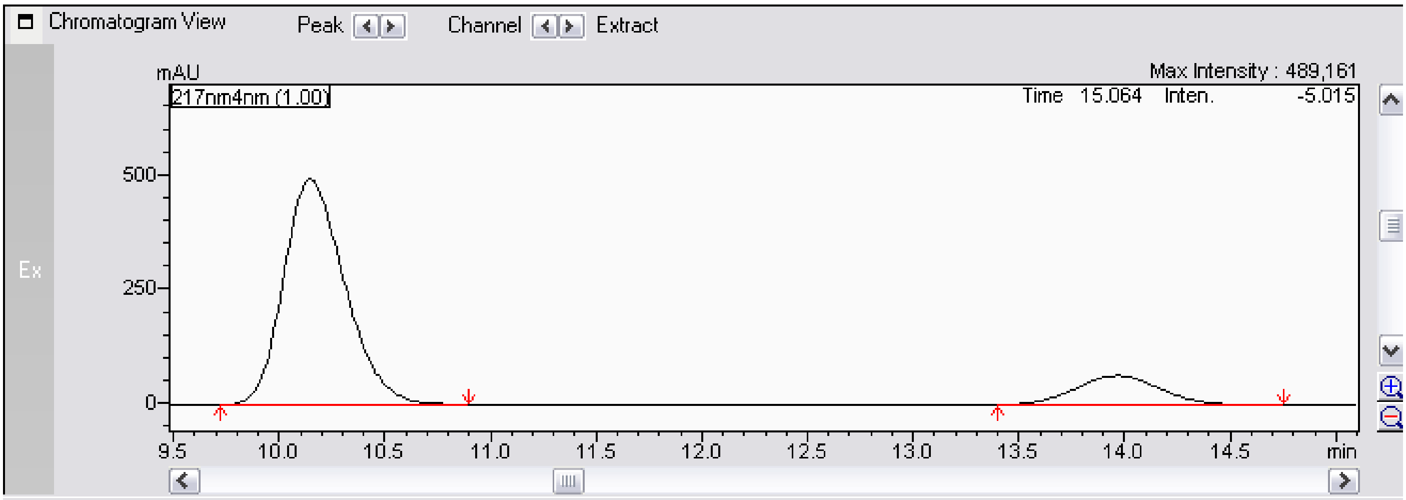

Results View - Peak Table

Peak Table | Compound | Group | Calibration Curve

Peak#	Ret. Time	Area	Height	Area%
1	10.148	9916168	492687	84.618
2	13.966	1802550	64452	15.382
Total		11718718	557139	100.000

N-(5,5,6,6,7,7,8,8,9,9,10,10,11,11,12,12,12-heptadecafluoro-2-(tetrahydro-2H-pyran-4-yl)dodecan-2-yl)-4-nitrobenzamide, benzoylated 3o

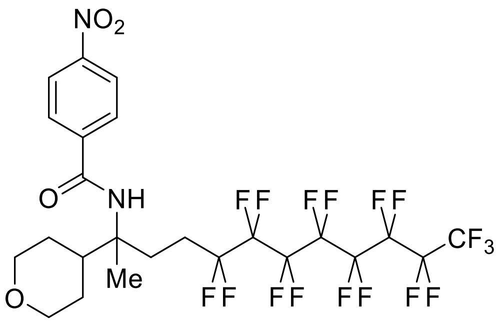

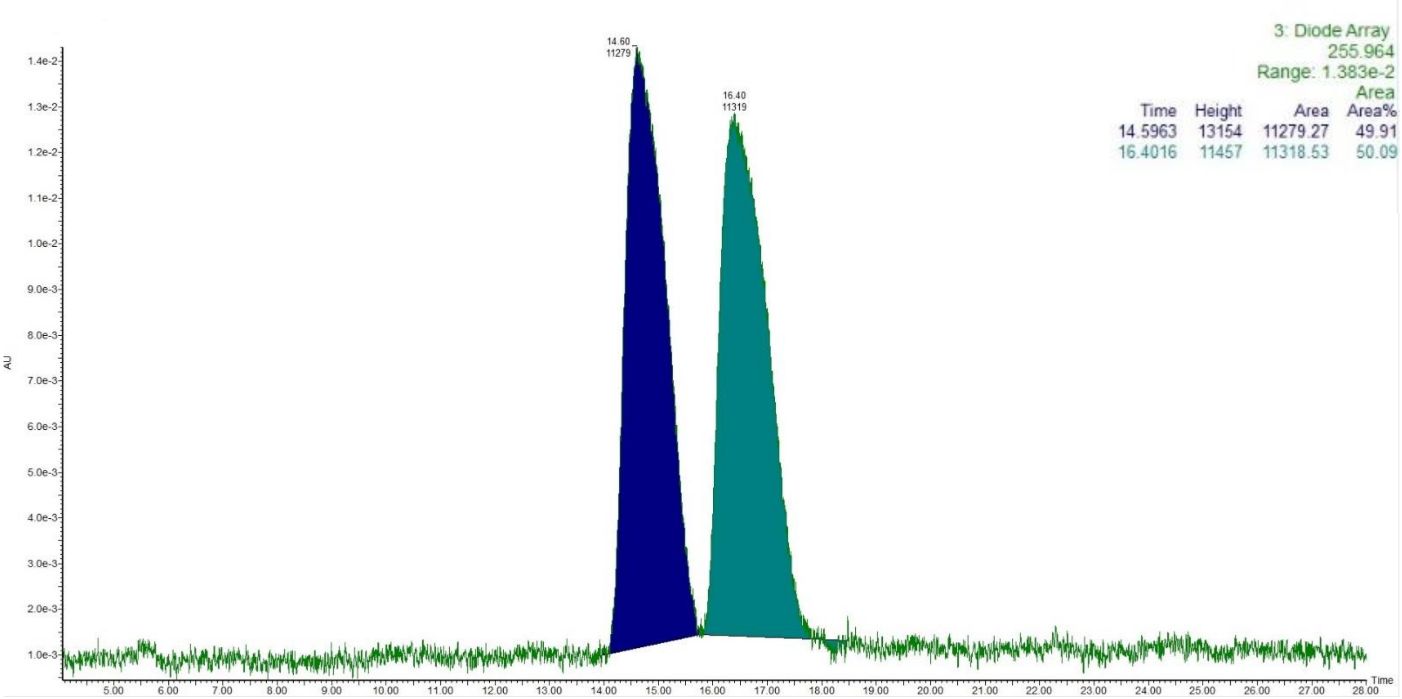

Time	Height	Area	Area%
14.5963	13154	11279.27	49.91
16.4016	11457	11318.53	50.09

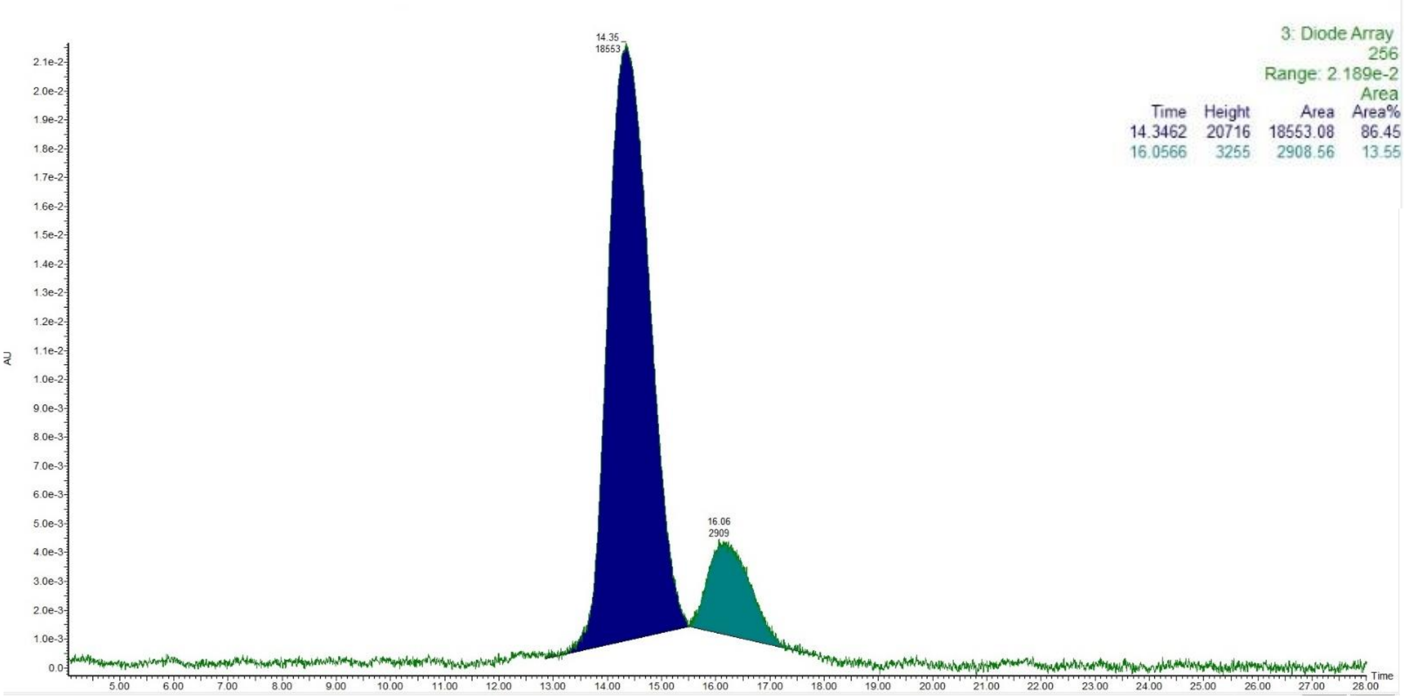

Time	Height	Area	Area%
14.3462	20716	18553.08	86.45
16.0566	3255	2908.56	13.55

N-(4-(N-methyl-N-(3-phenyl-3-(4-(trifluoromethyl)phenoxy)propyl)sulfamoyl)-2-(tetrahydro-2H-pyran-4-yl)butan-2-yl)-4-nitrobenzamide, benzoylated 3p

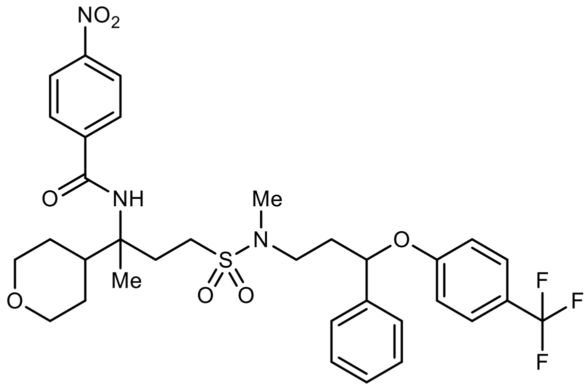

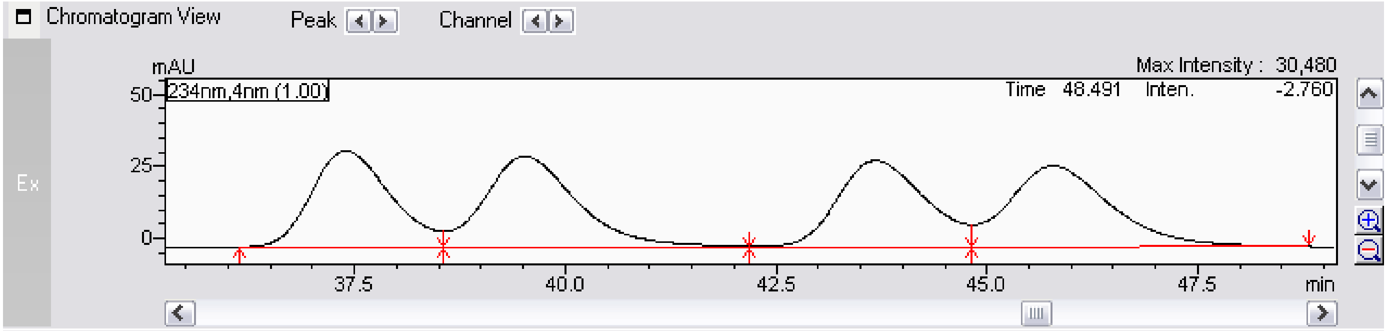

Results View - Peak Table

Peak Table | Compound | Group | Calibration Curve

Peak#	Ret. Time	Area	Height	Area%
1	37.397	2184653	33541	23.942
2	39.519	2390439	31587	26.198
3	43.679	2172284	29960	23.807
4	45.783	2377225	28159	26.053
Total		9124601	123246	100.000

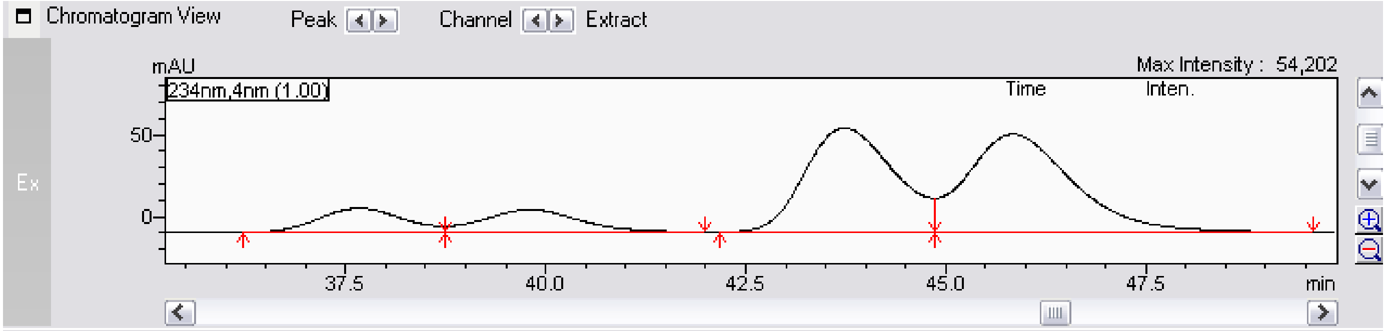

Results View - Peak Table

Peak Table | Compound | Group | Calibration Curve

Peak#	Ret. Time	Area	Height	Area%
1	37.665	1085472	14707	8.554
2	39.794	1138418	13713	8.971
3	43.730	4931589	63704	38.864
4	45.837	5533815	59820	43.610
Total		12689293	151944	100.000

N-(4-(morpholinosulfonyl)-2-(tetrahydro-2H-pyran-4-yl)butan-2-yl)-4-nitrobenzamide, benzoylated 3q

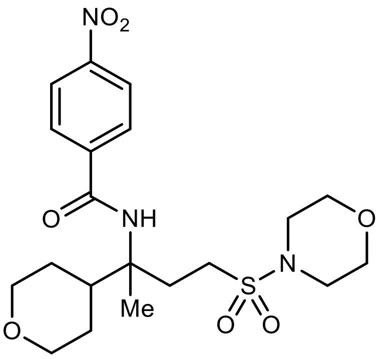

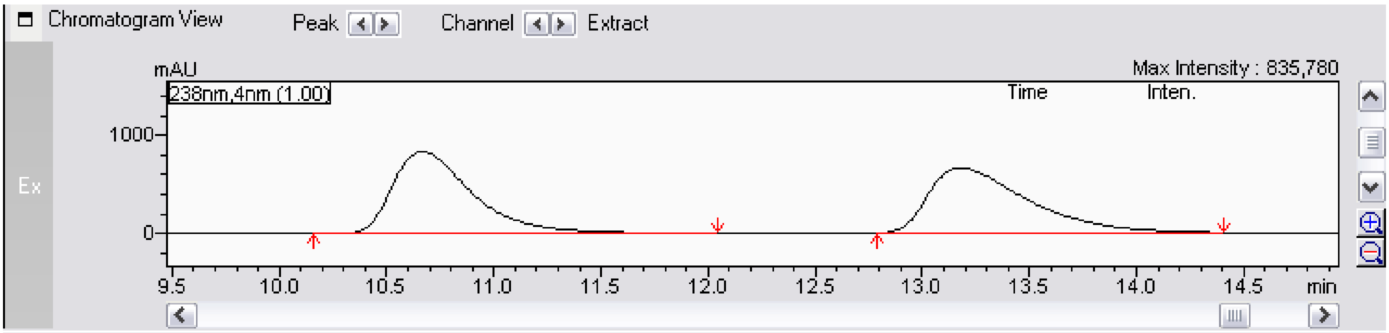

Results View - Peak Table

Peak Table | Compound | Group | Calibration Curve

Peak#	Ret. Time	Area	Height	Area%
1	10.667	21663911	834473	50.451
2	13.179	21276678	660970	49.549
Total		42940589	1495442	100.000

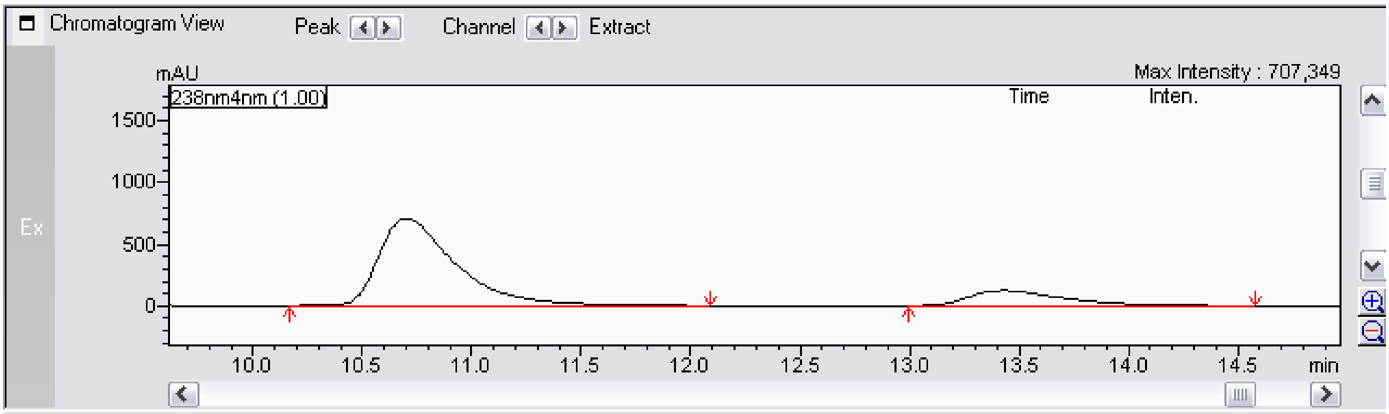

Results View - Peak Table

Peak Table | Compound | Group | Calibration Curve

Peak#	Ret. Time	Area	Height	Area%
1	10.701	17087819	703567	82.094
2	13.429	3727059	123316	17.906
Total		20814877	826884	100.000

N-(4-((4-(6-fluorobenzo[d]isoxazol-3-yl)piperidin-1-yl)sulfonyl)-2-(tetrahydro-2H-pyran-4-yl)butan-2-yl)-4-nitrobenzamide, benzoylated 3r

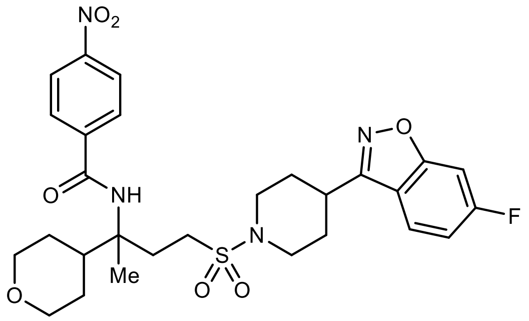

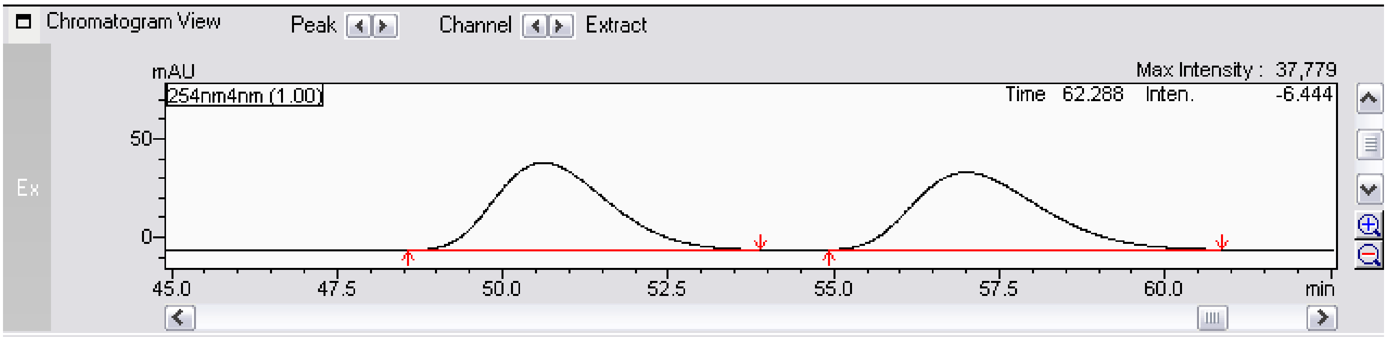

Results View - Peak Table

Peak Table | Compound | Group | Calibration Curve

Peak#	Ret. Time	Area	Height	Area%
1	50.599	5305423	44281	50.060
2	56.981	5292757	39234	49.940
Total		10598181	83515	100.000

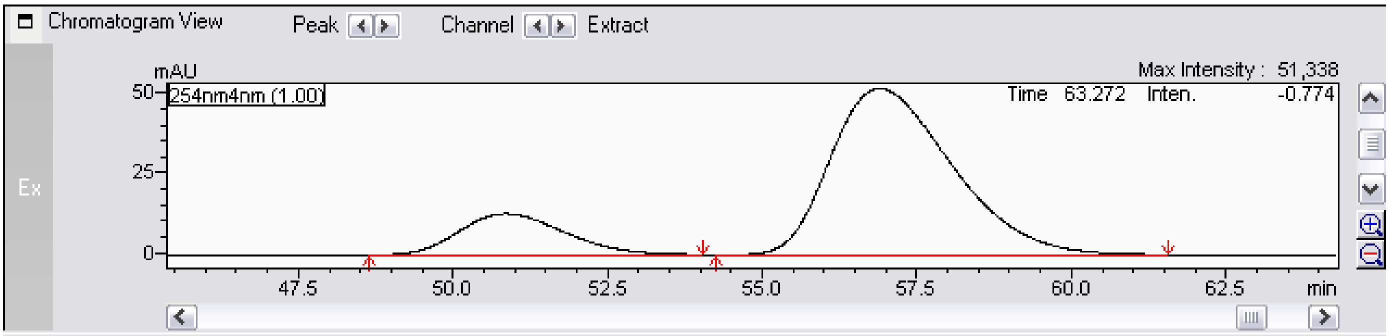

Results View - Peak Table

Peak Table | Compound | Group | Calibration Curve

Peak#	Ret. Time	Area	Height	Area%
1	50.866	1534985	12906	17.772
2	56.910	7102294	51956	82.228
Total		8637280	64861	100.000

tert-butyl 2-methyl-4-(4-nitrobenzamido)-4-(tetrahydro-_2H_-pyran-4-yl)pentanoate, benzoylated 3s

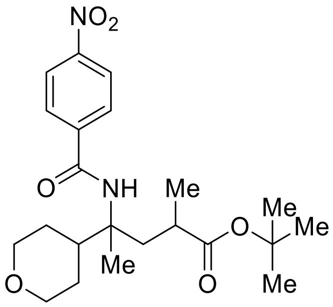

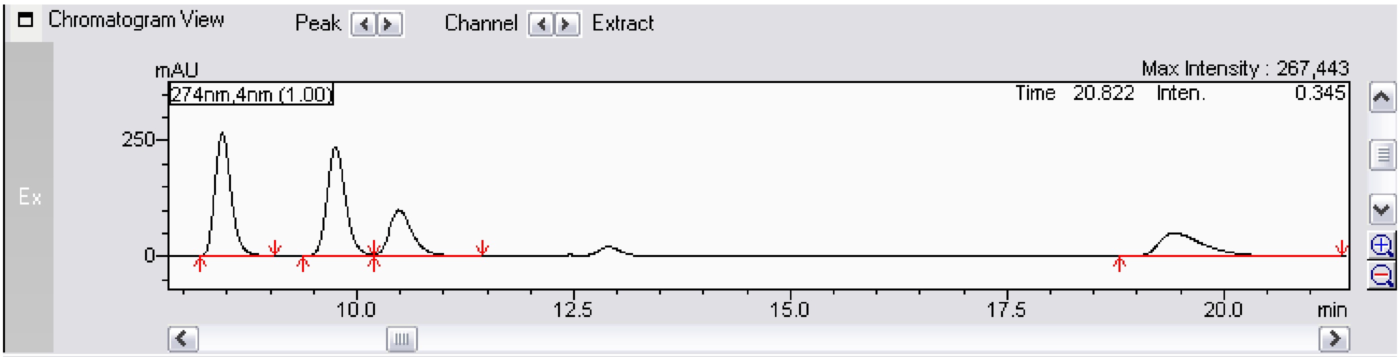

Results View - Peak Table

Peak Table | Compound | Group | Calibration Curve

Peak#	Ret. Time	Area	Height	Area%
1	8.450	3478003	266730	32.404
2	9.755	3476381	235793	32.389
3	10.490	1865430	99397	17.380
4	19.418	1913390	51348	17.827
Total		10733204	653267	100.000

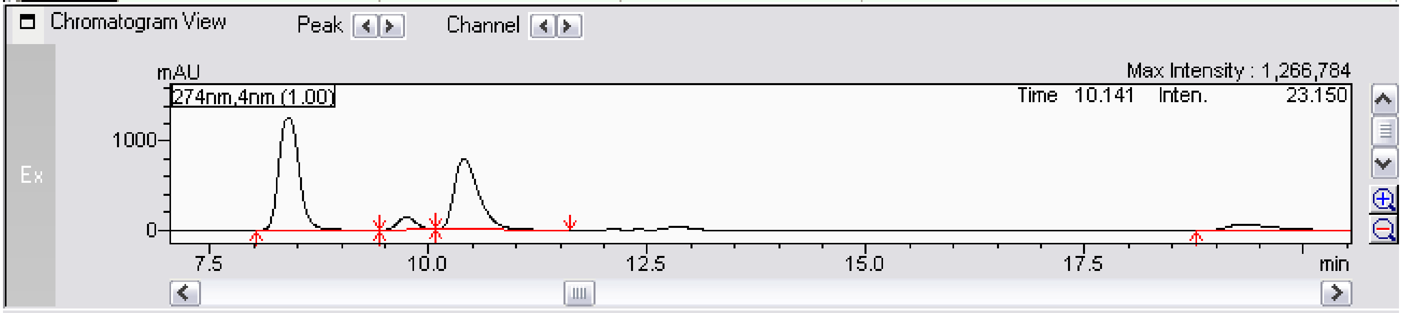

Results View - Peak Table

Peak Table | Compound | Group | Calibration Curve

Peak#	Ret. Time	Area	Height	Area%
1	8.396	20216207	1265906	50.263
2	9.743	2052636	143898	5.103
3	10.396	15592608	796356	38.768
4	19.312	2359056	63052	5.865
Total		40220507	2269212	100.000

(S)-2-(benzyloxy)-1-(3,5-bis(trifluoromethyl)phenyl)ethan-1-amine, 4e

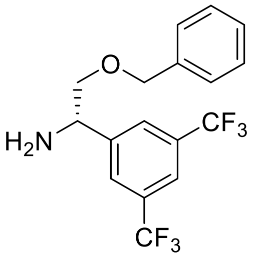

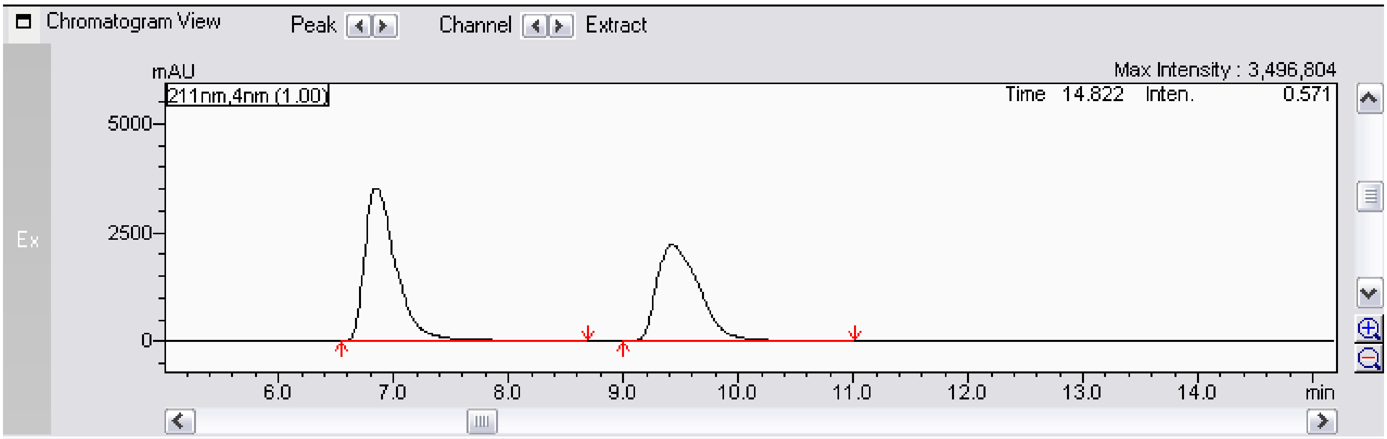

Results View - Peak Table

Peak Table | Compound | Group | Calibration Curve

Peak#	Ret. Time	Area	Height	Area%
1	6.848	66730609	3474376	53.992
2	9.424	56861839	2225458	46.008
Total		123592447	5699834	100.000

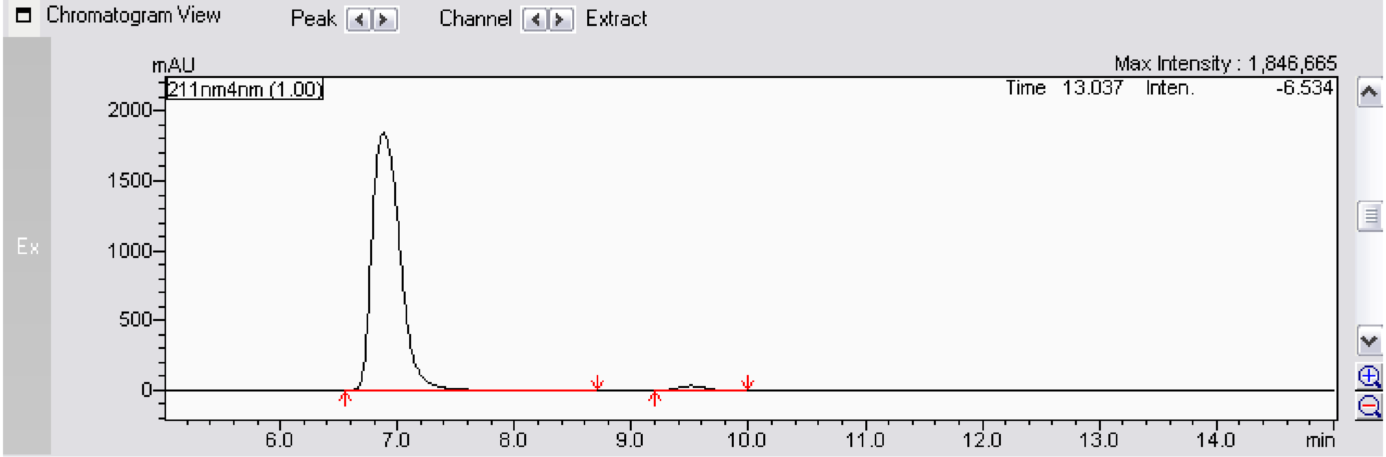

Results View - Peak Table

Peak Table | Compound | Group | Calibration Curve

Peak#	Ret. Time	Area	Height	Area%
1	6.882	30878407	1845962	98.003
2	9.509	629119	36264	1.997
Total		31507527	1882226	100.000

5-methyl-5-(tetrahydro-2H-pyran-4-yl)pyrrolidin-2-one, 6

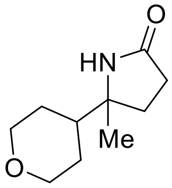

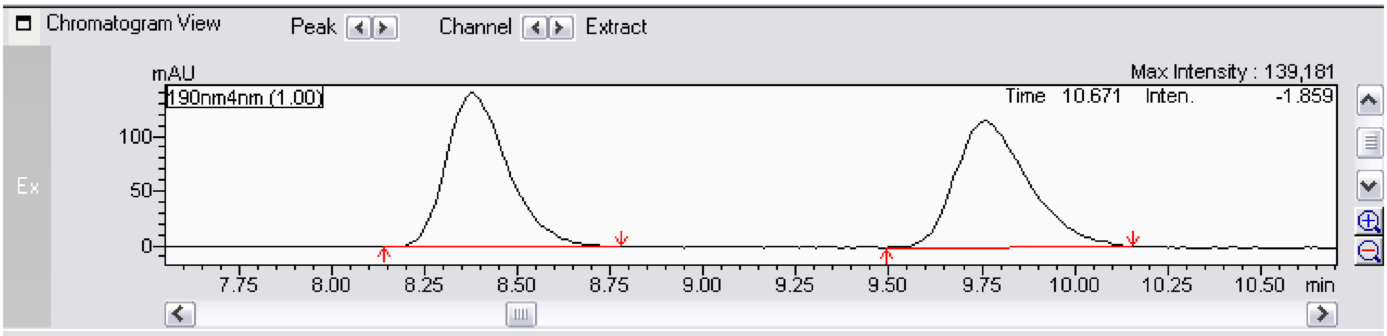

Results View - Peak Table

Peak Table | Compound | Group | Calibration Curve

Peak#	Ret. Time	Area	Height	Area%
1	8.377	1608621	139889	50.603
2	9.759	1570294	115330	49.397
Total		3178915	255219	100.000

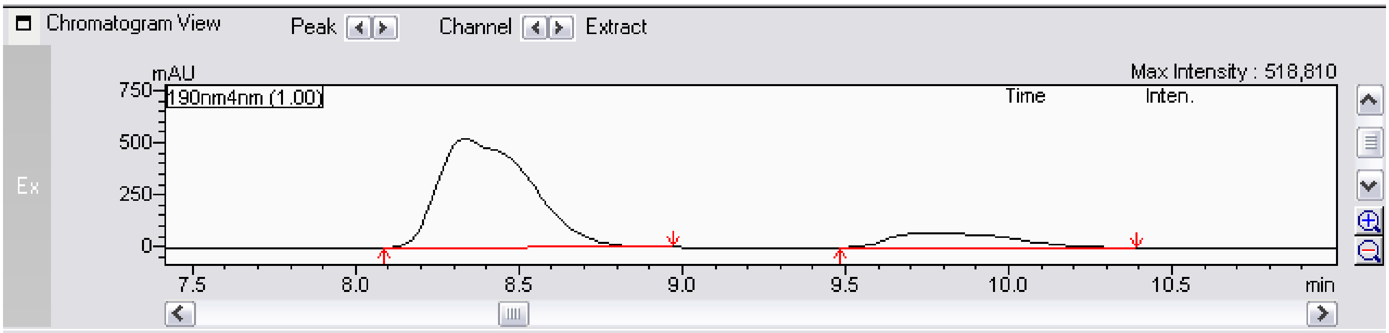

Results View - Peak Table

Peak Table | Compound | Group | Calibration Curve

Peak#	Ret. Time	Area	Height	Area%
1	8.332	10178978	521281	85.320
2	9.753	1751330	70260	14.680
Total		11930308	591541	100.000

2-methyl-2-(tetrahydro-2H-pyran-4-yl)pyrrolidine, 7

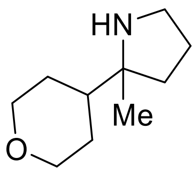

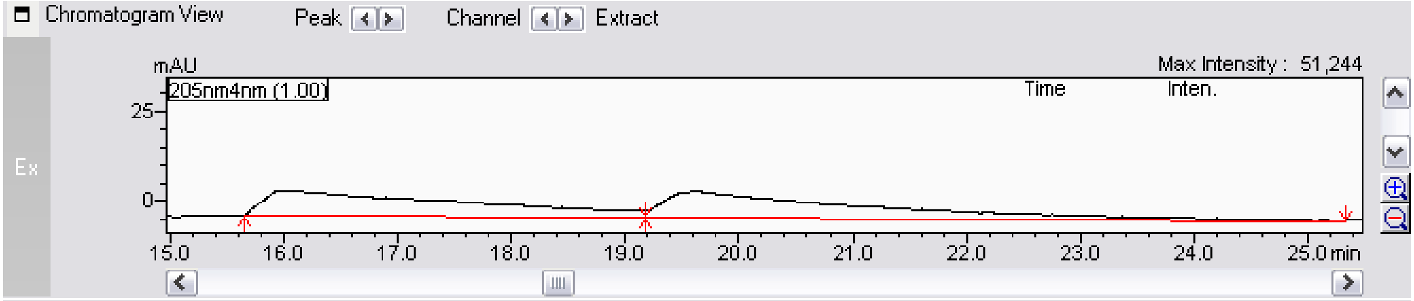

Results View - Peak Table

Peak Table | Compound | Group | Calibration Curve

Peak#	Ret. Time	Area	Height	Area%
1	15.961	903607	7057	49.642
2	19.637	916639	7352	50.358
Total		1820246	14409	100.000

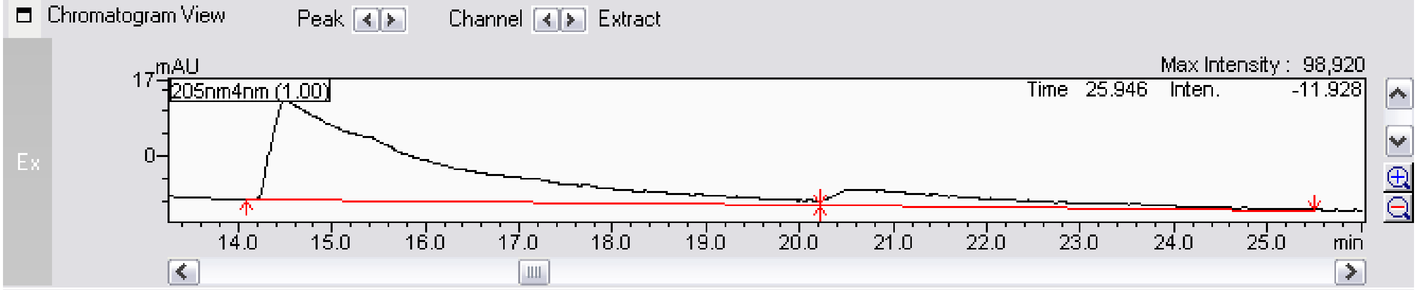

Results View - Peak Table

Peak Table | Compound | Group | Calibration Curve

Peak#	Ret. Time	Area	Height	Area%
1	14.493	2541415	22195	84.710
2	20.653	458738	3743	15.290
Total		3000153	25938	100.000

***tert*-butyl 3-(4-aminotetrahydro-2*H*-pyran-4-yl)-2-methylpropanoate, 8**

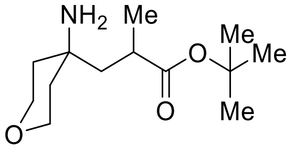

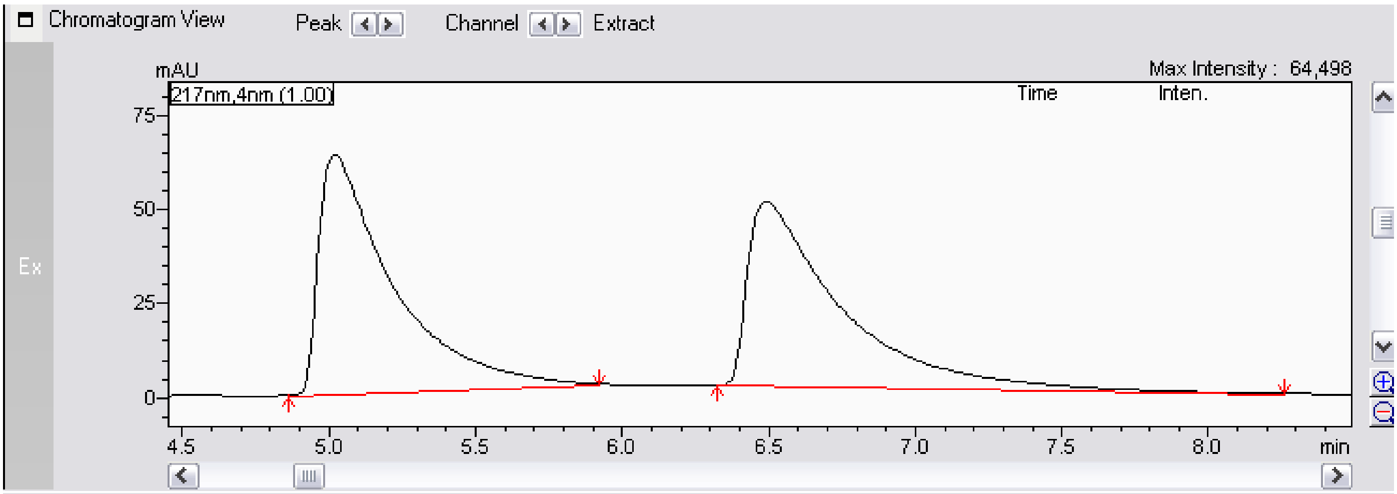

Results View - Peak Table

Peak Table | Compound | Group | Calibration Curve

Peak#	Ret. Time	Area	Height	Area%
1	5.020	1126704	63700	50.476
2	6.490	1105452	49255	49.524
Total		2232155	112954	100.000

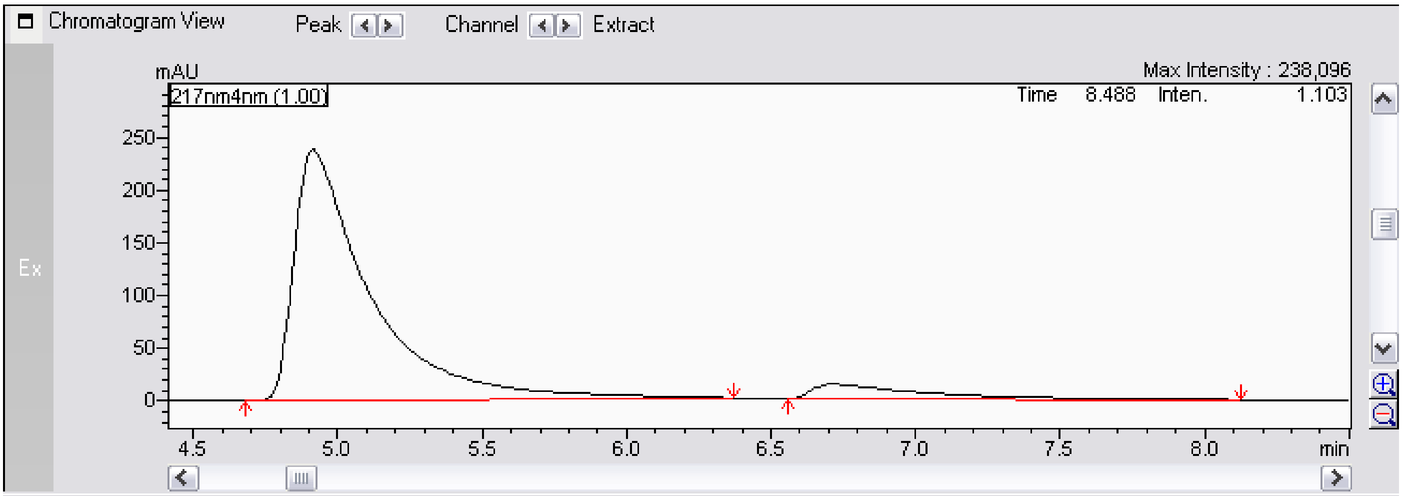

Results View - Peak Table

Peak Table | Compound | Group | Calibration Curve

Peak#	Ret. Time	Area	Height	Area%
1	4.915	4315299	237171	92.853
2	6.717	332170	13173	7.147
Total		4647469	250344	100.000

NMR Spectra

4-(vinylsulfonyl)morpholine, 2g ^{1}H NMR (500 MHz, $CDCl_3$)

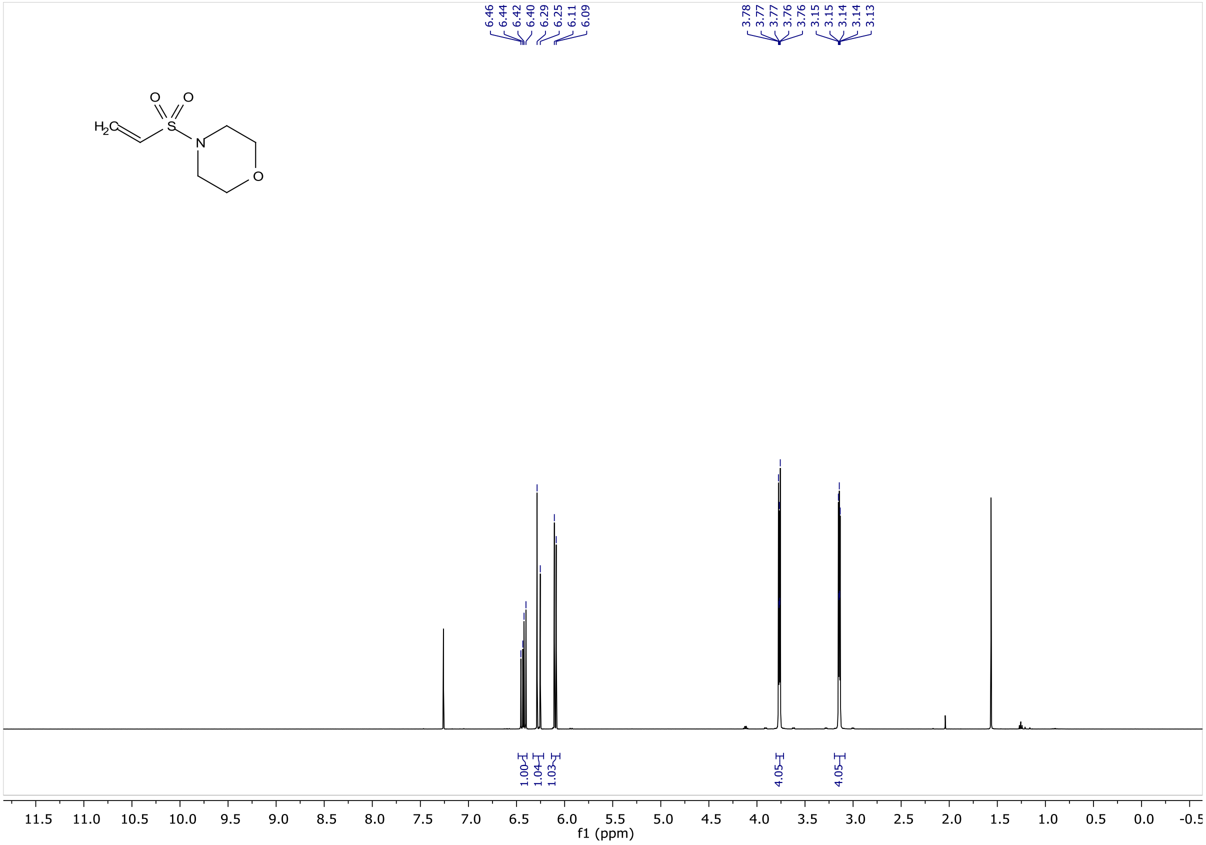

^{13}C NMR (126 MHz, $CDCl_3$)

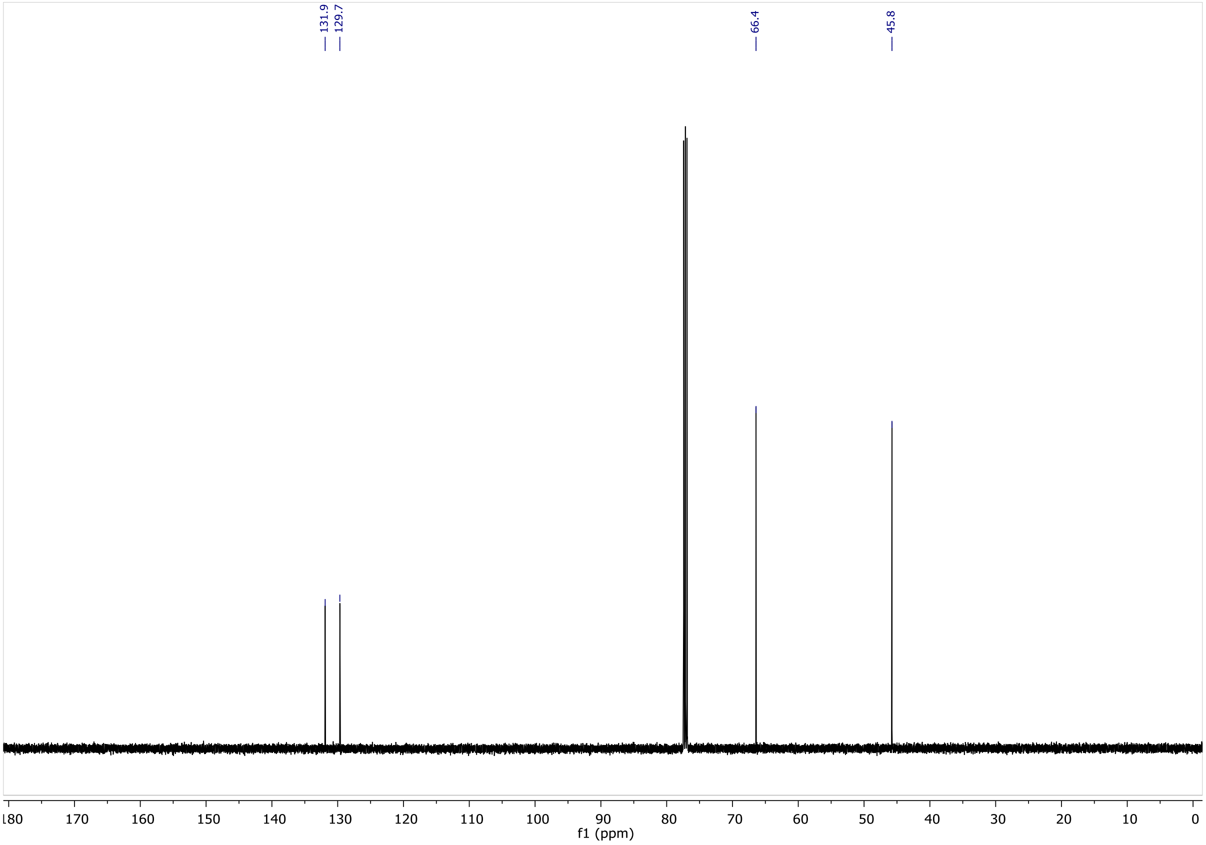

N-methyl-N-(3-phenyl-3-(4-(trifluoromethyl)phenoxy)propyl)ethenesulfonamide, 2f ^{1}H NMR (500 MHz, $CDCl_3$)

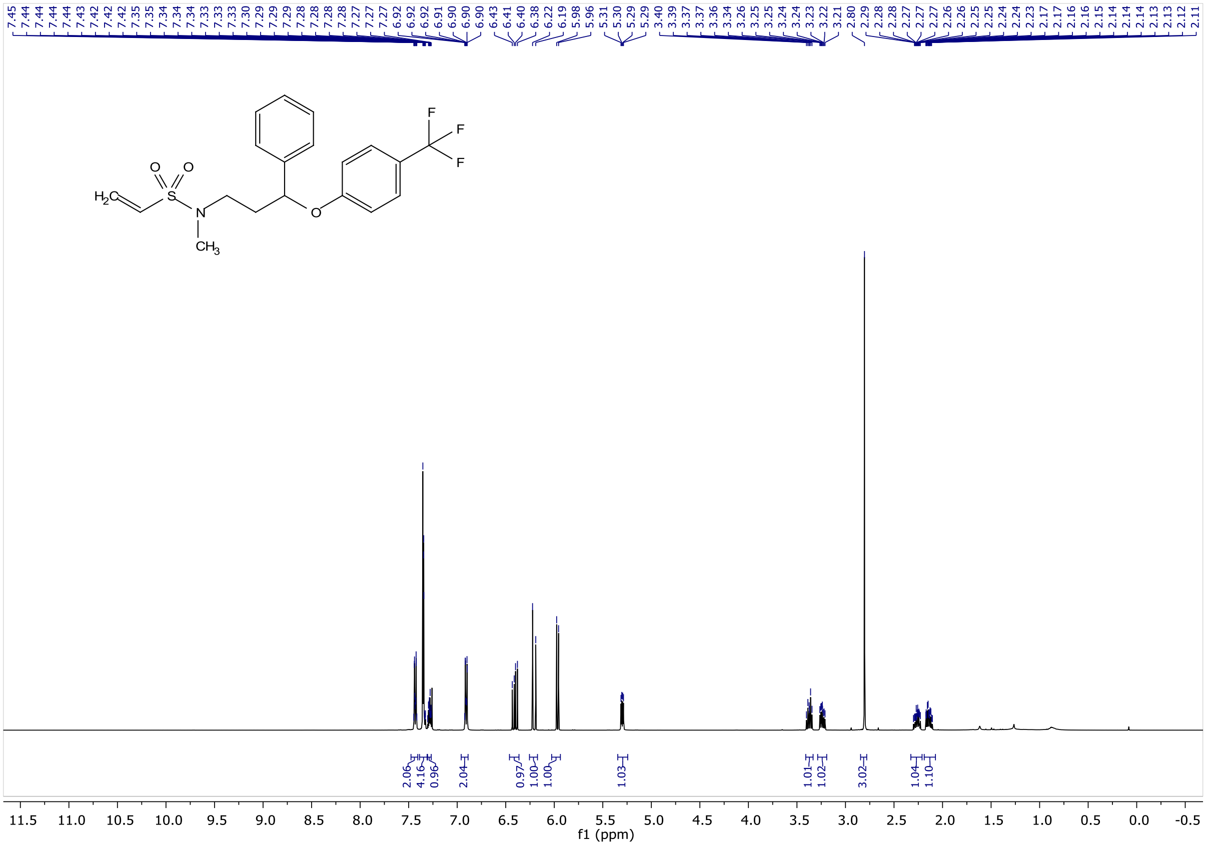

^{13}C NMR (126 MHz, $CDCl_3$)

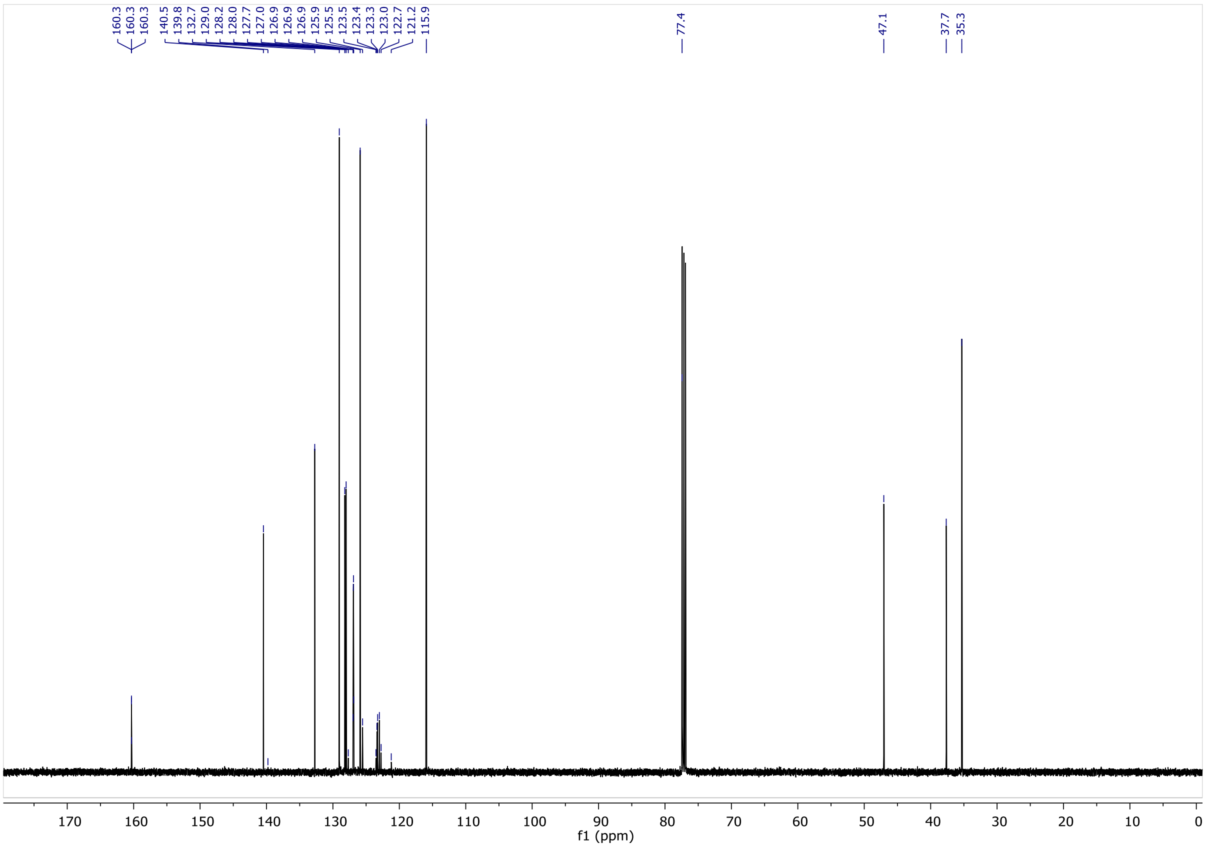

^{19}F NMR (376 MHz, $CDCl_3$)

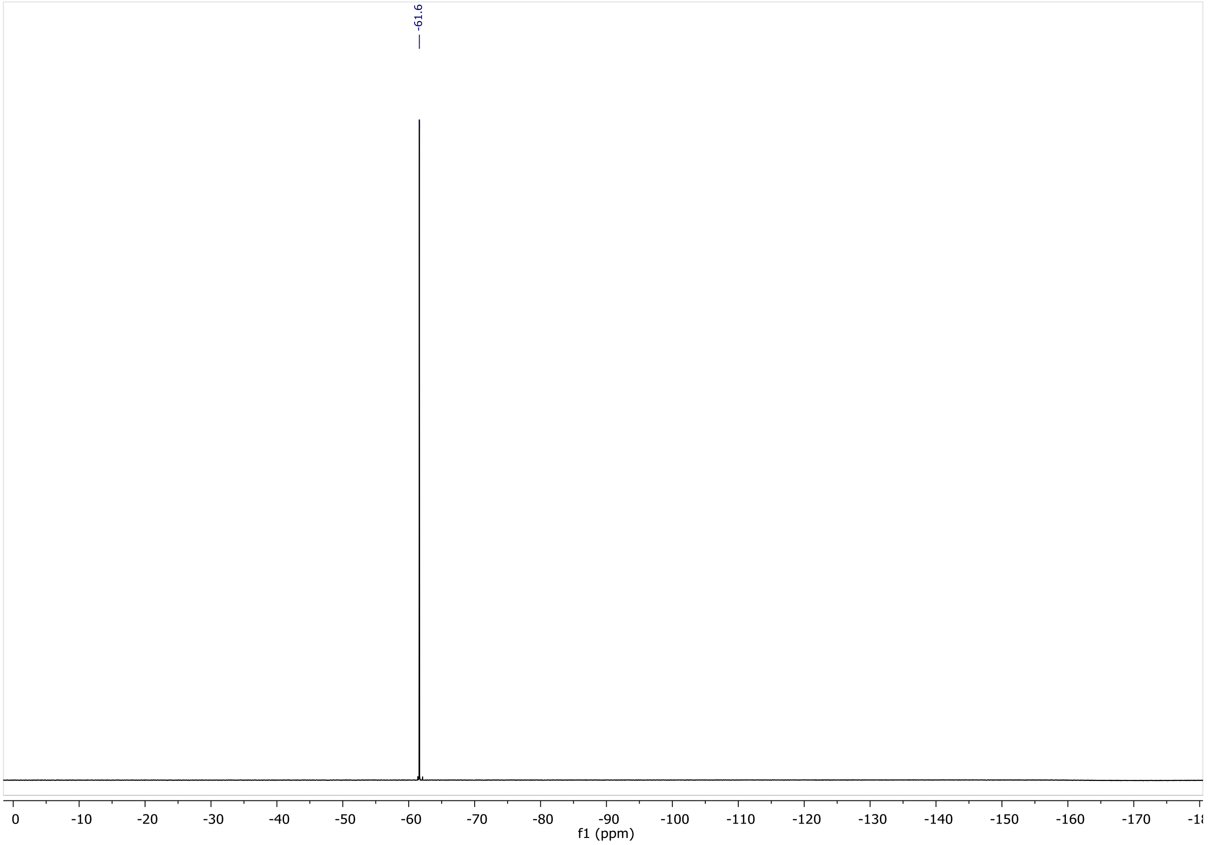

6-fluoro-3-(1-(vinylsulfonyl)piperidin-4-yl)benzo[d]isoxazole, 2h ^{1}H NMR (400 MHz, $CDCl_3$)

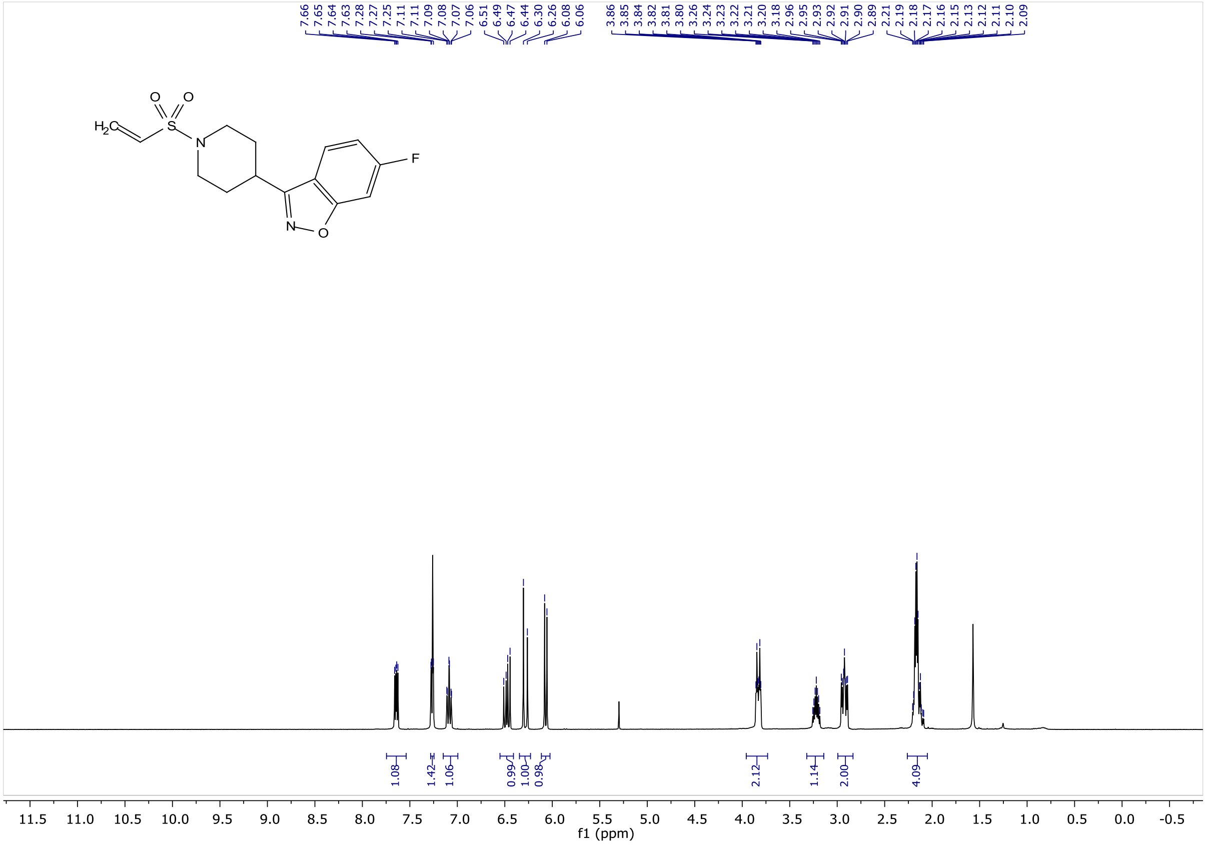

^{13}C NMR (101 MHz, $CDCl_3$)

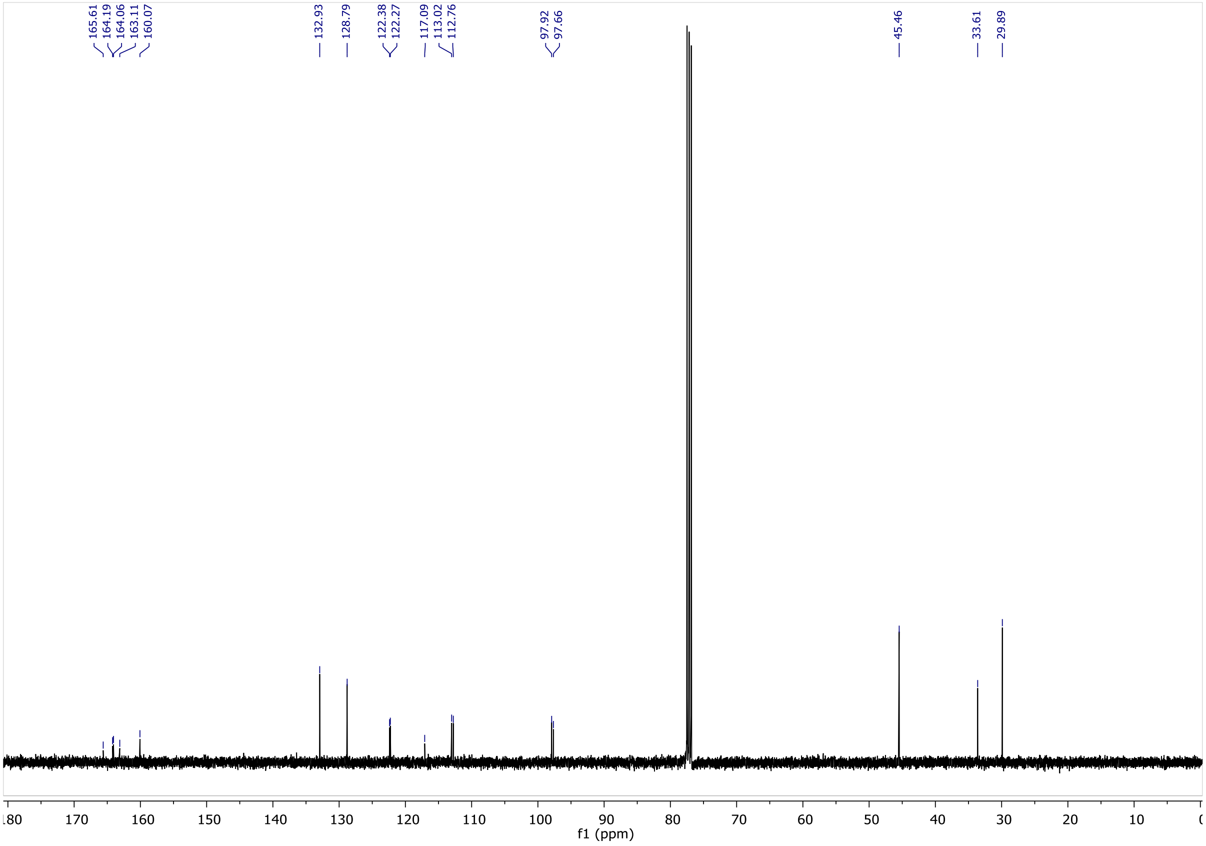

^{19}F NMR (376 MHz, $CDCl_3$)

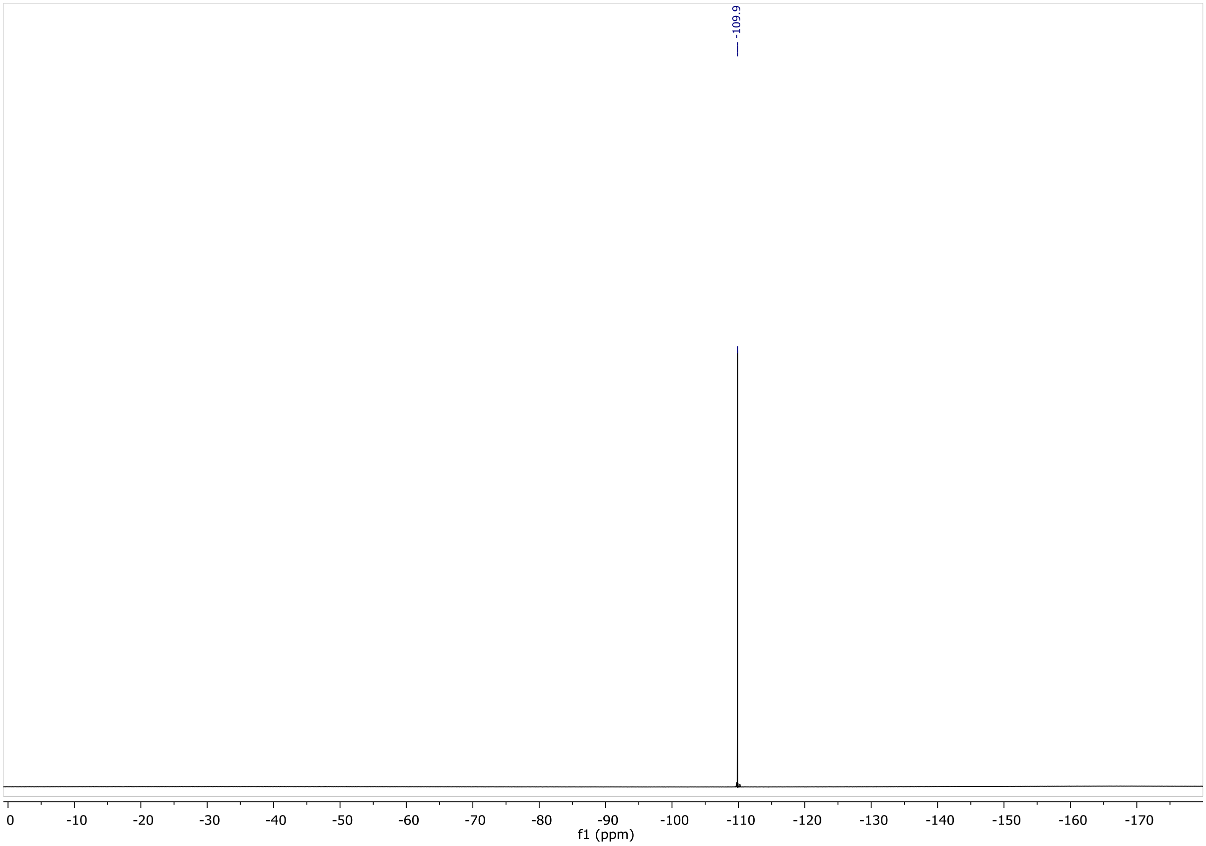

***tert*-butyl (*R*)-4-amino-4-cyclobutylpentanoate, 3b** ^{1}H NMR (400 MHz, $CDCl_3$)

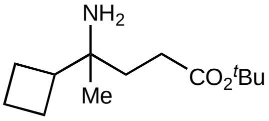

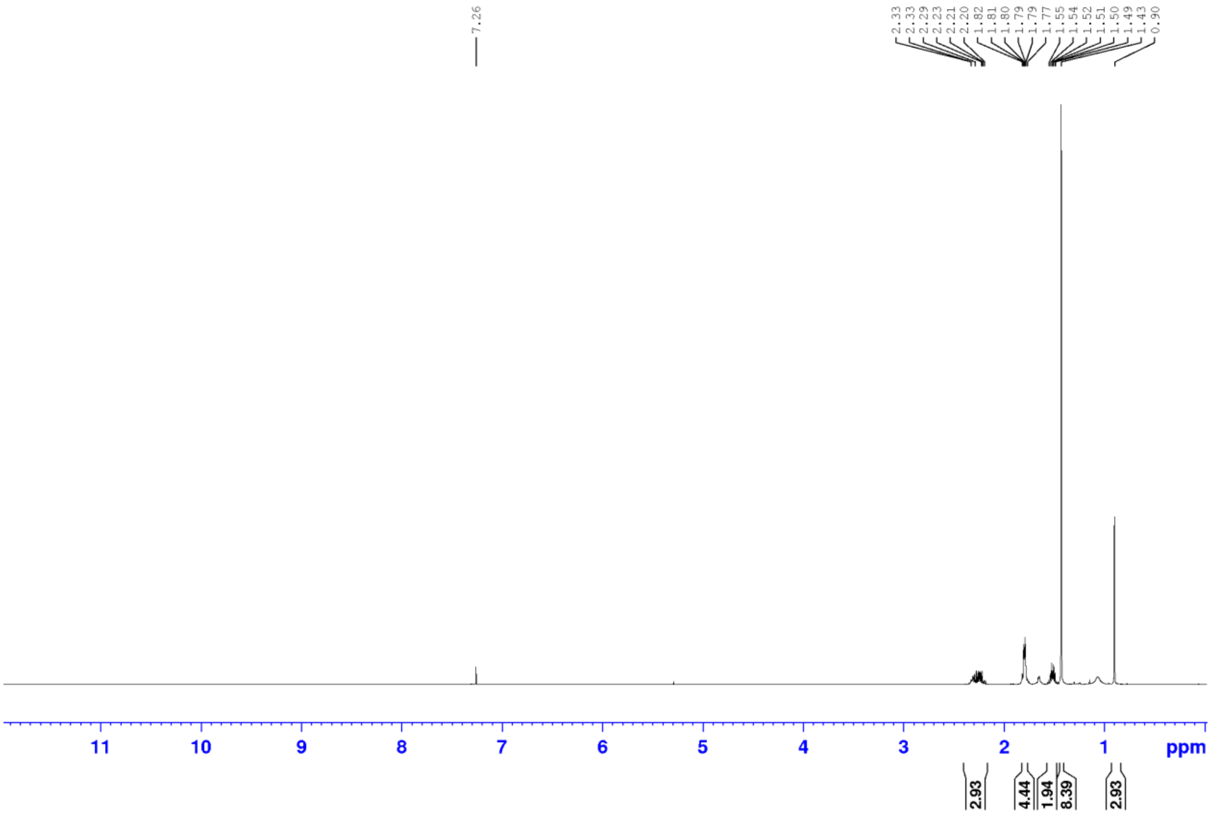

^{13}C NMR (101 MHz, $CDCl_3$)

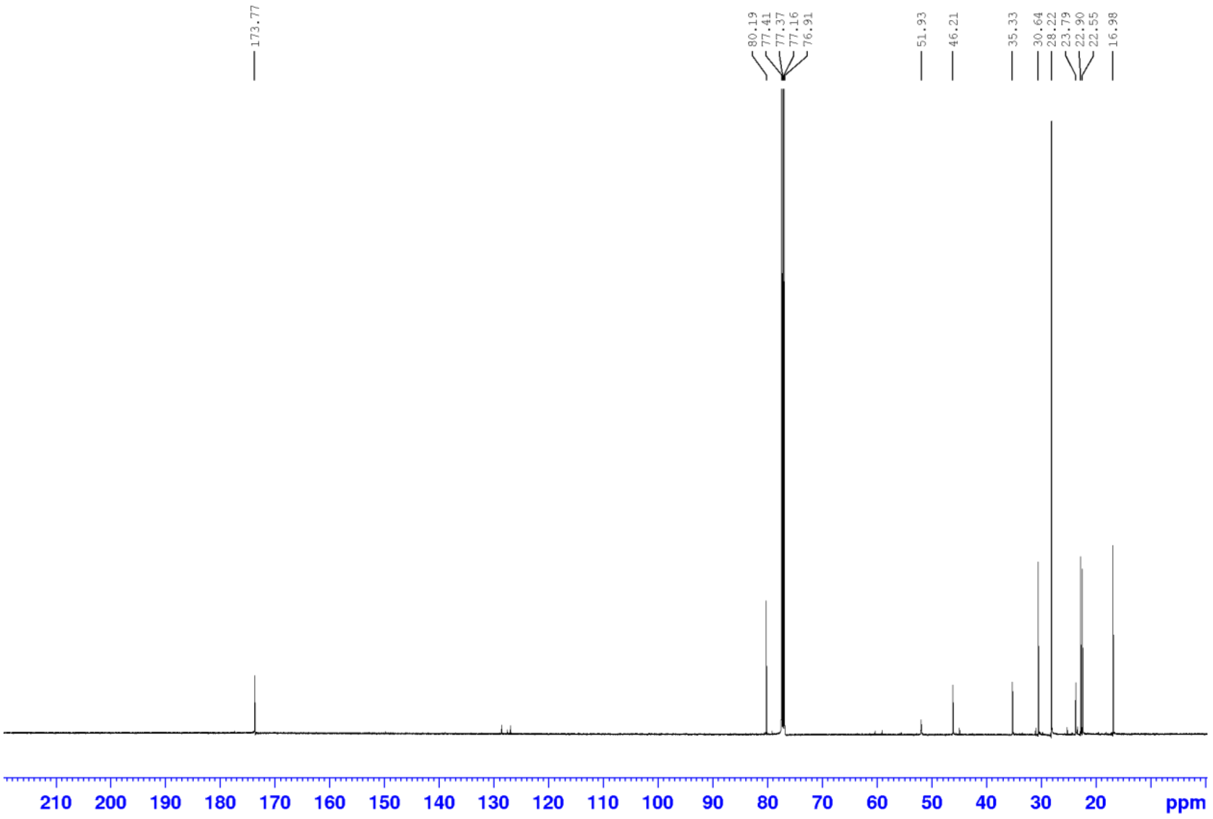

***tert*-butyl (*R*)-4-amino-4-cyclohexylpentanoate, 3c** ^{1}H NMR (400 MHz, $CDCl_3$)

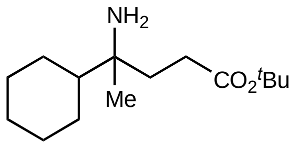

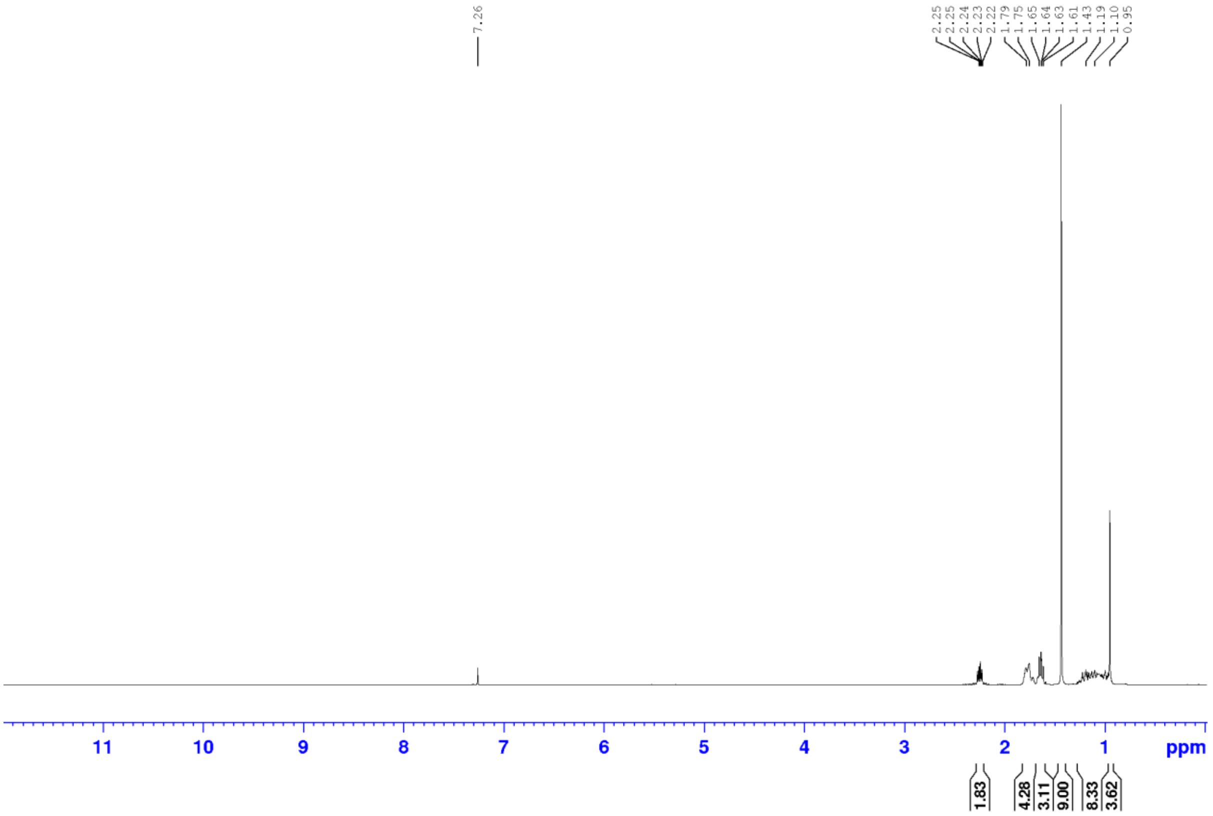

^{13}C NMR (101 MHz, $CDCl_3$)

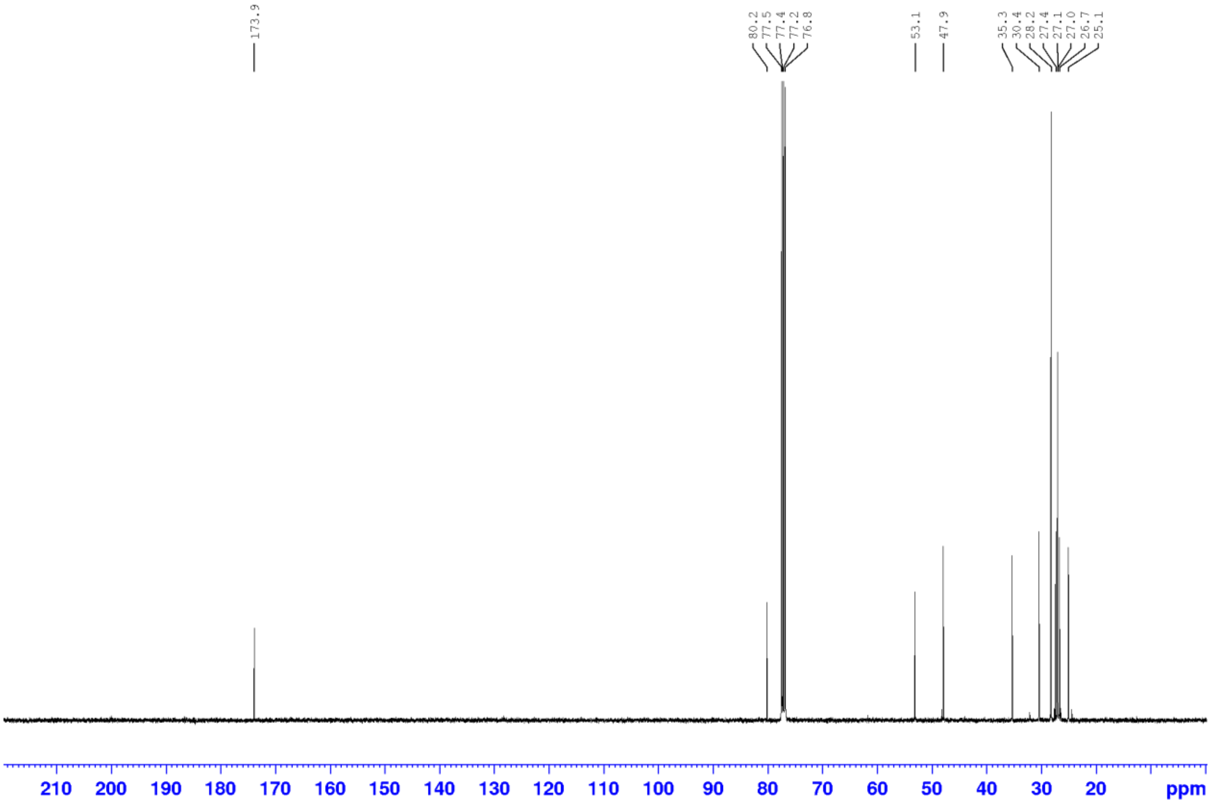

***tert*-butyl (*R*)-4-amino-4,5-dimethylhexanoate, 3d** ^{1}H NMR

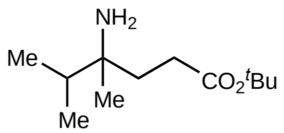

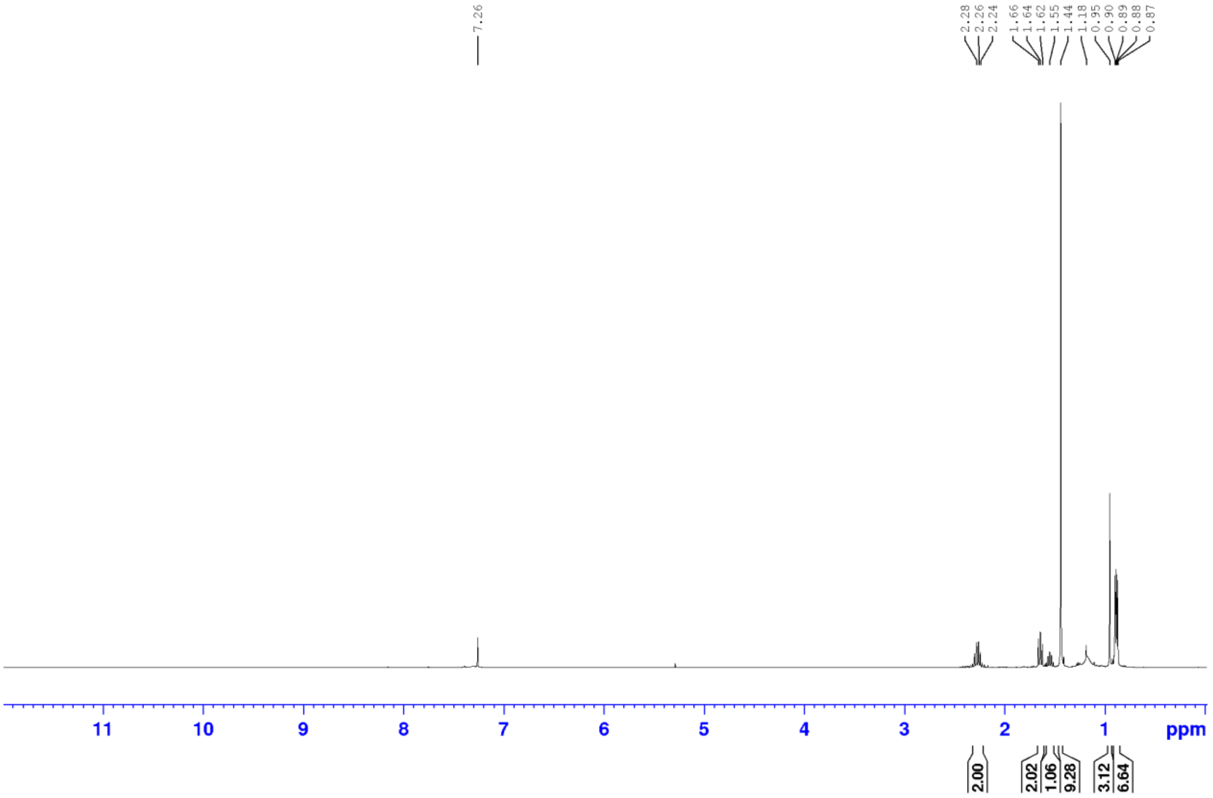

^{13}C NMR (101 MHz, CDCl3)

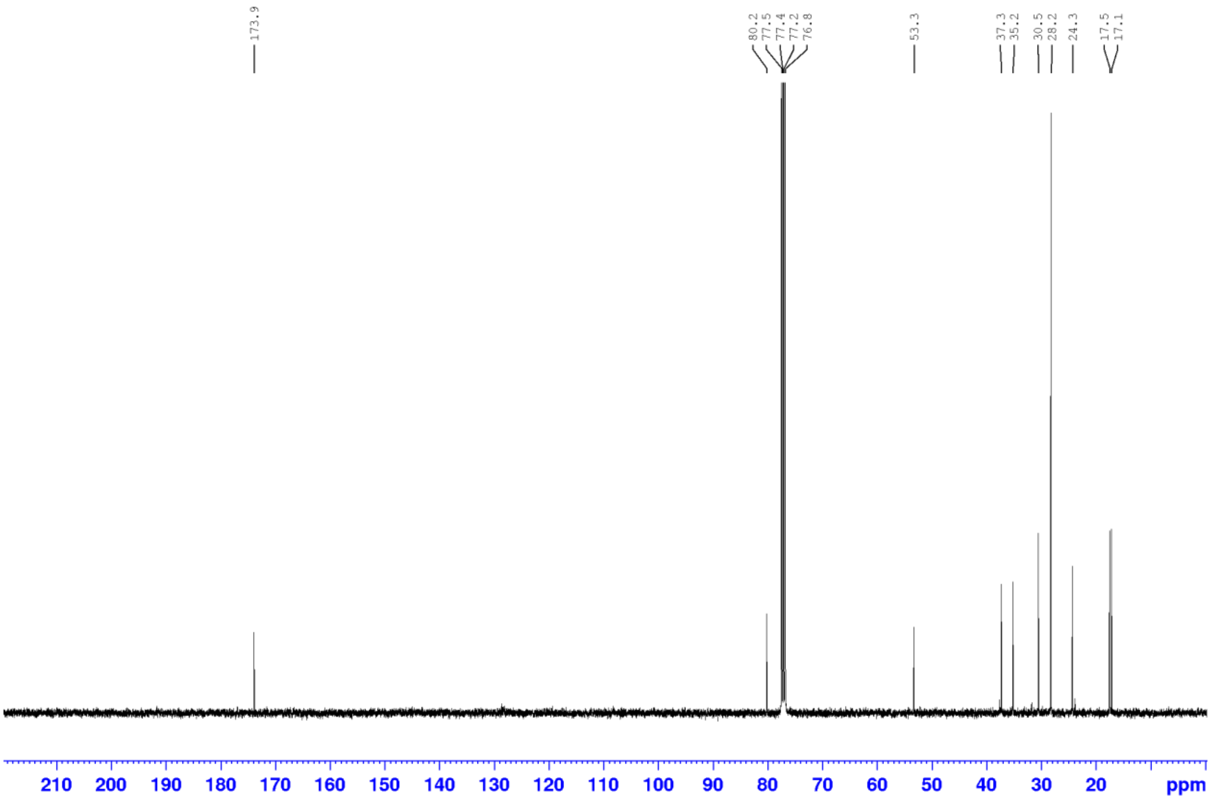

***tert*-butyl (*R*)-4-amino-4-cycloheptylpentanoate, 3e** ^{1}H NMR (400 MHz, $CDCl_3$)

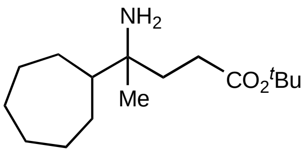

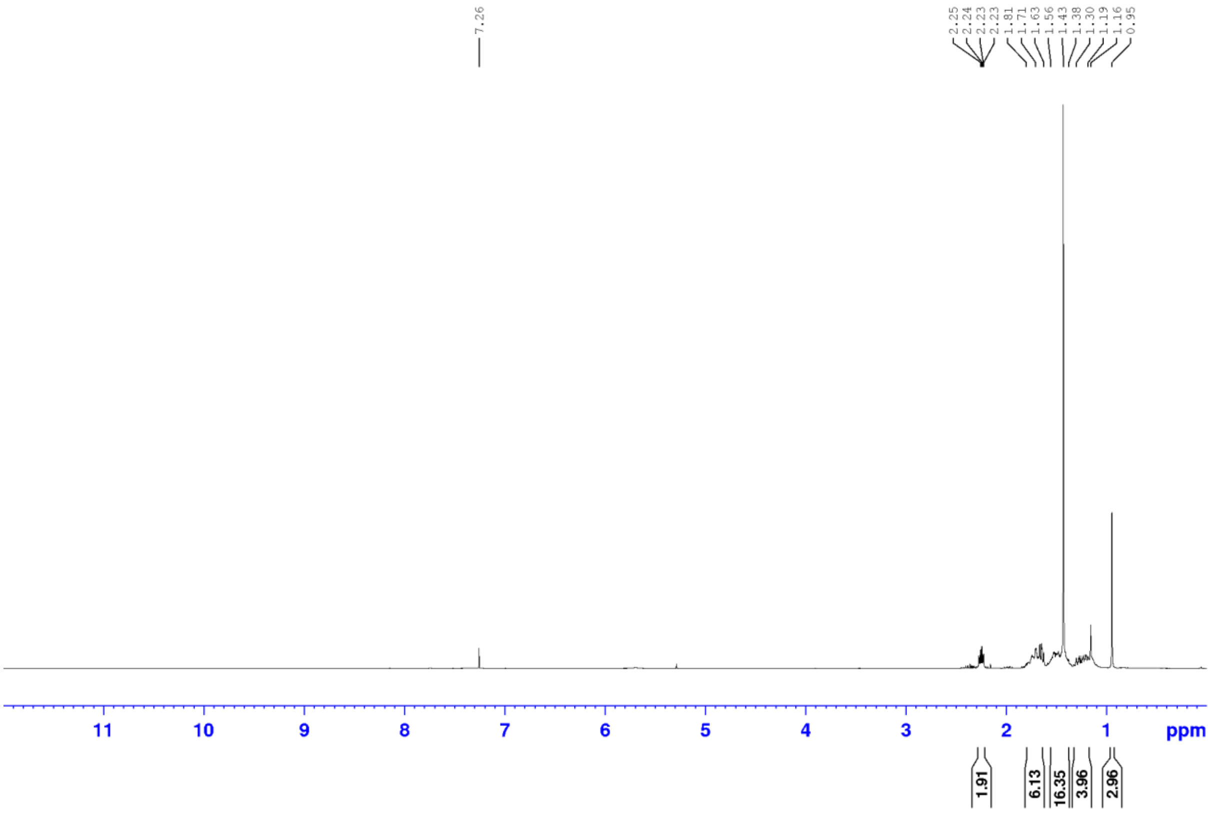

^{13}C NMR (101 MHz, $CDCl_3$)

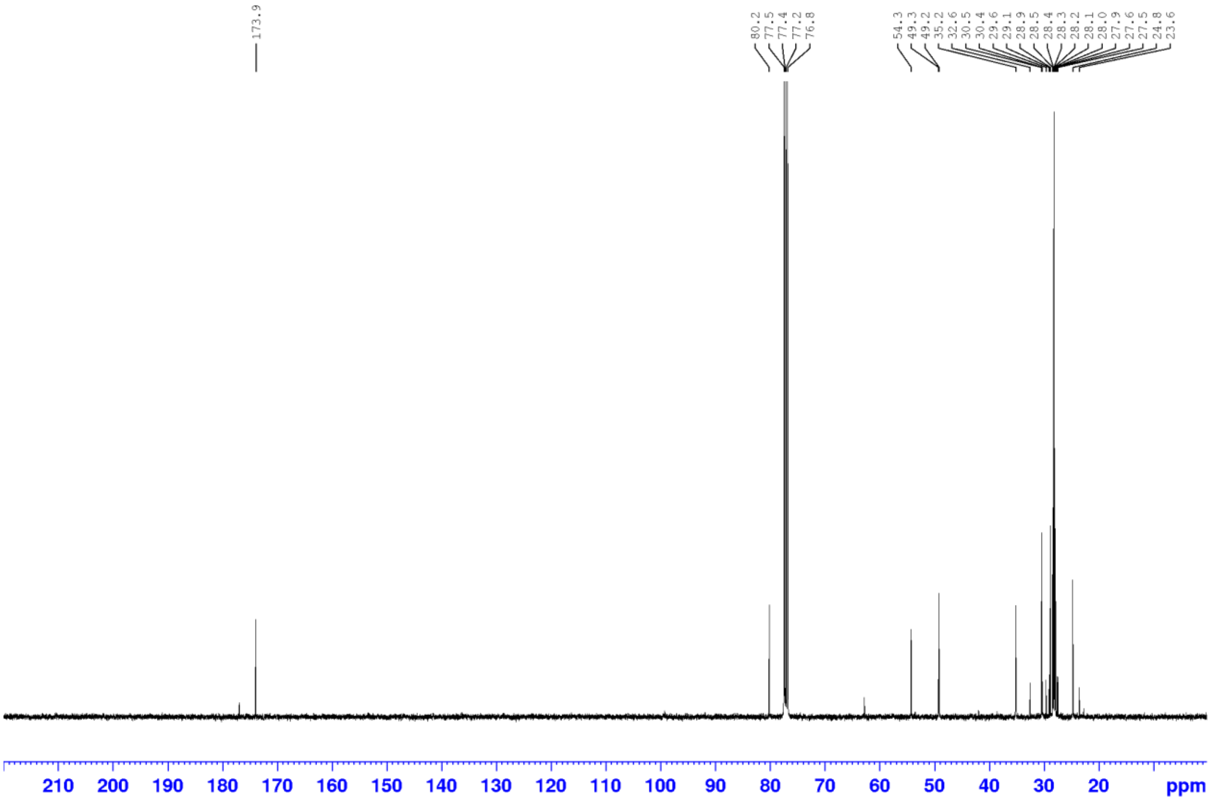

***tert*-butyl (*R*)-4-amino-4-(4,4-dimethylcyclohexyl)pentanoate, 3f** ^{1}H NMR (400 MHz, $CDCl_3$)

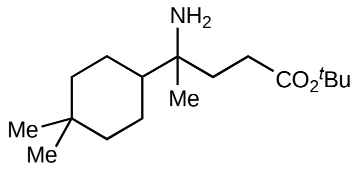

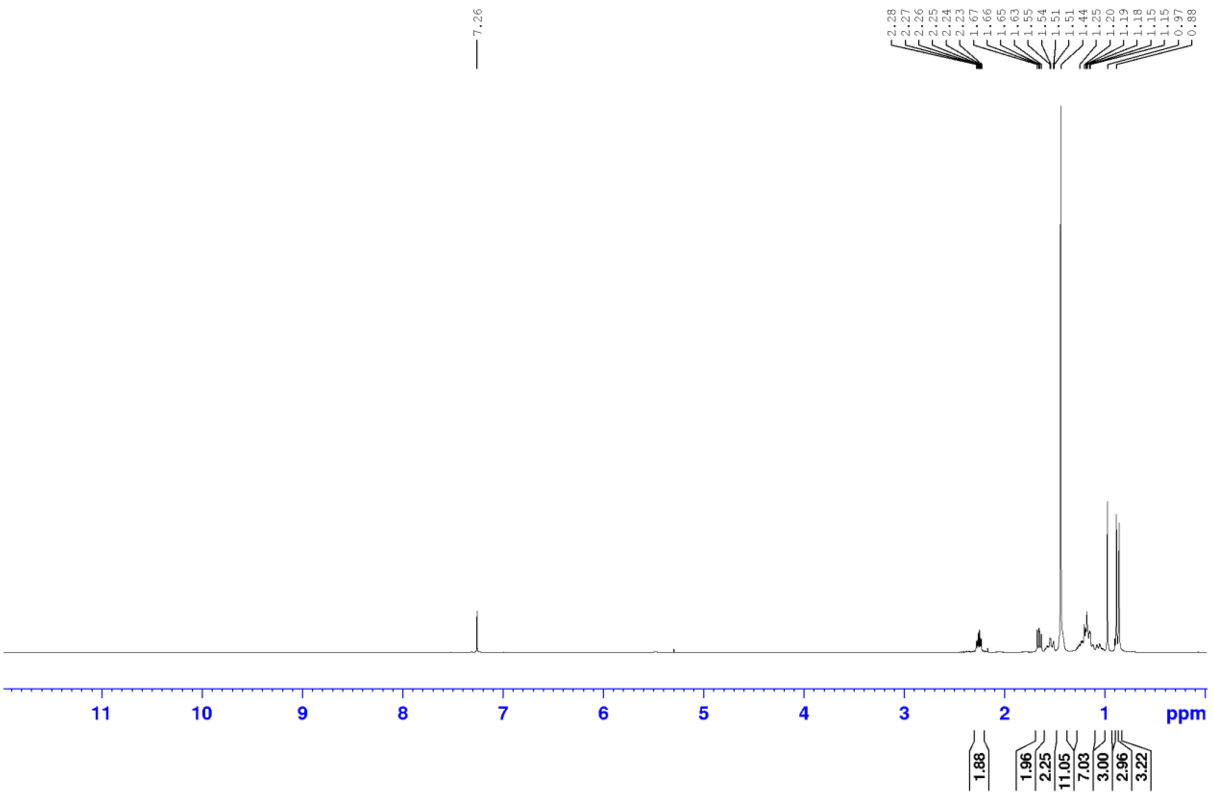

^{13}C NMR (101 MHz, $CDCl_3$)

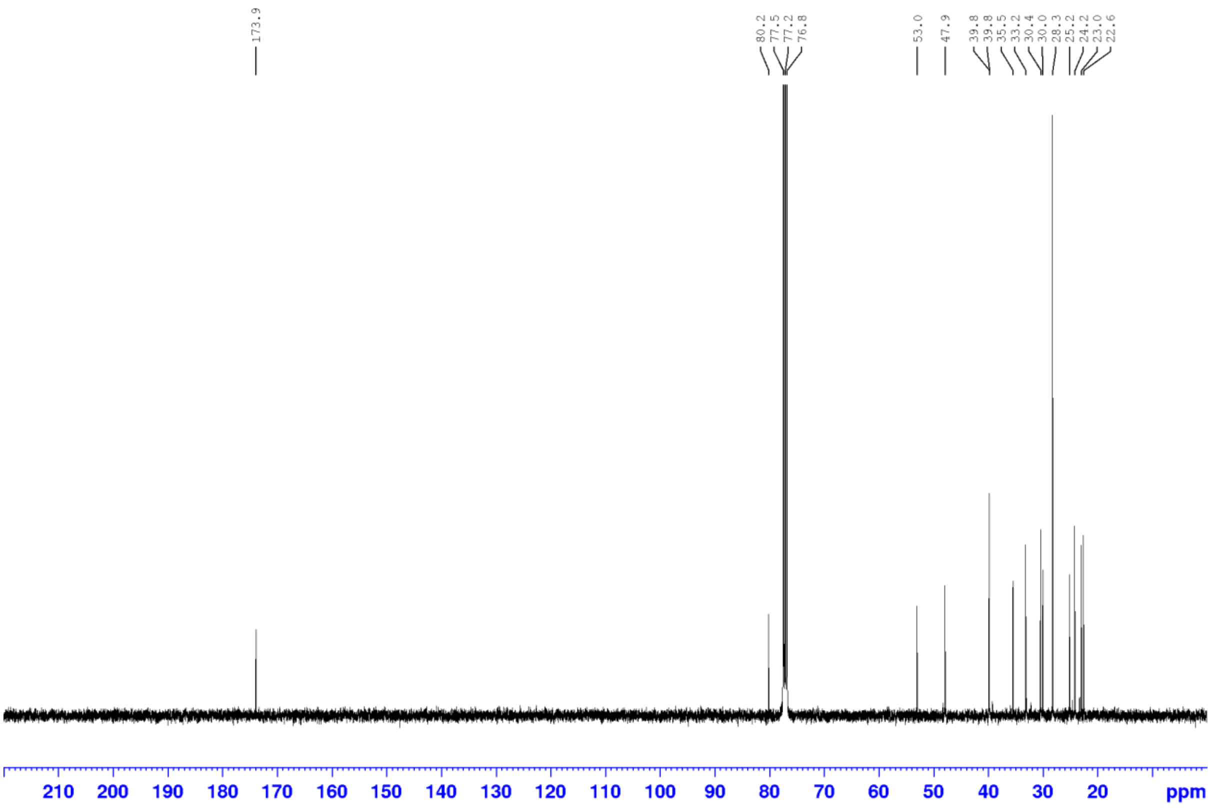

***tert*-butyl (*R*)-4-amino-4-(tetrahydro-2*H*-thiopyran-4-yl)pentanoate, 3g** ^{1}H NMR (400 MHz, $CDCl_3$)

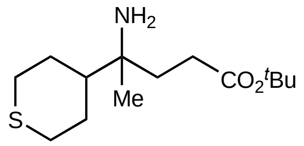

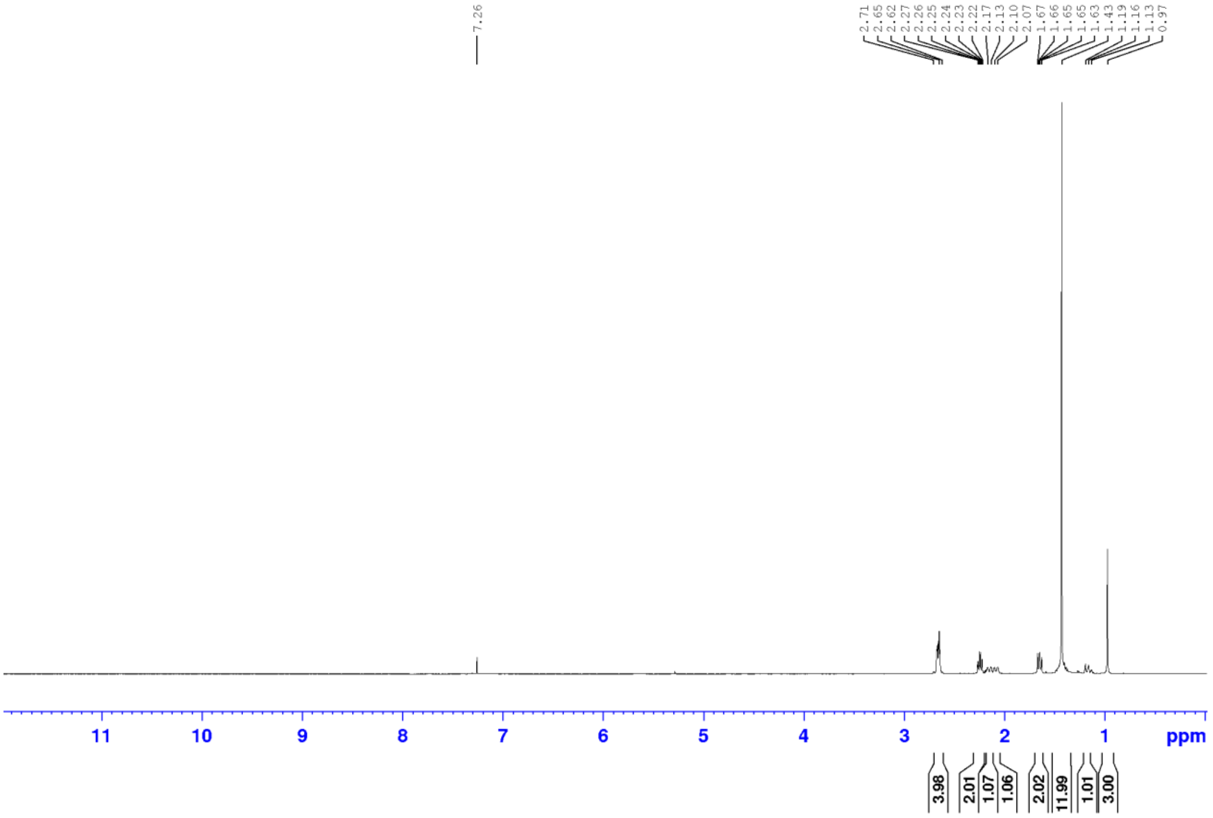

^{13}C NMR (101 MHz, $CDCl_3$)

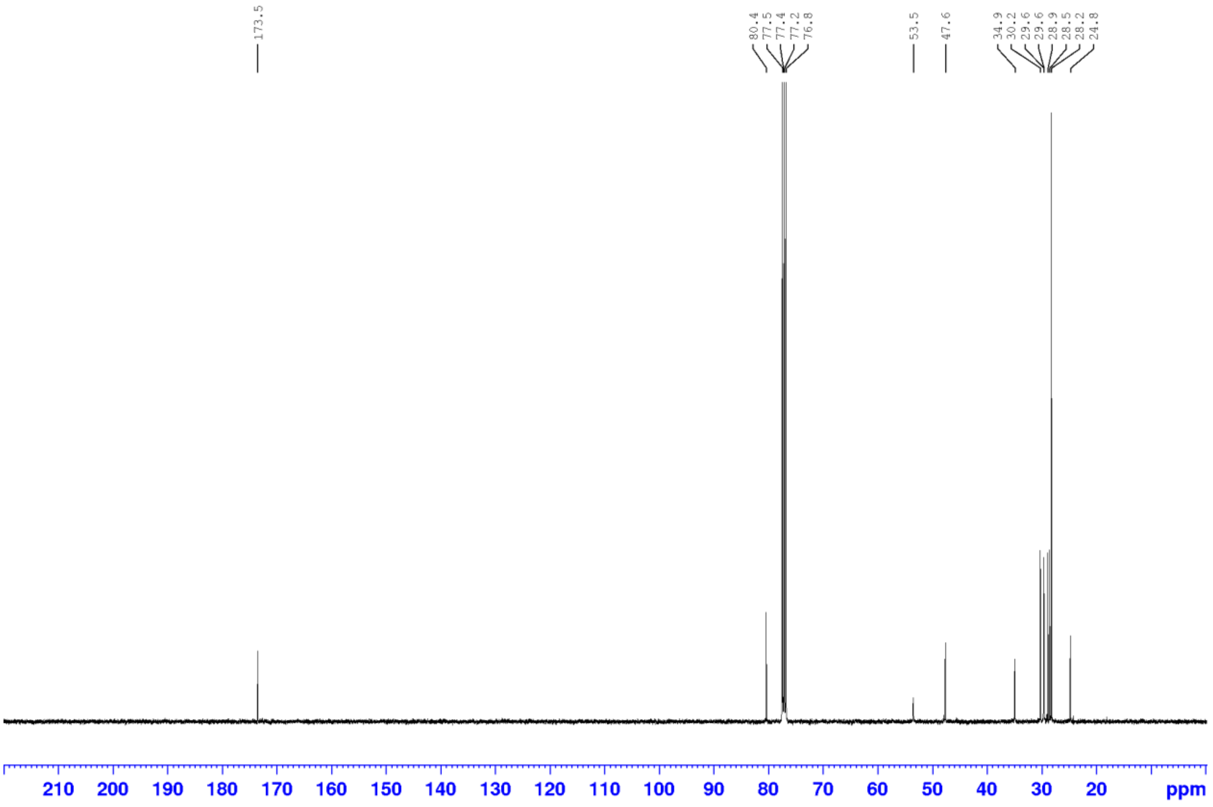

***tert*-butyl (*R*)-4-(2-amino-5-(*tert*-butoxy)-5-oxopentan-2-yl)piperidine-1-carboxylate, 3h** ^{1}H NMR (400 MHz, $CDCl_3$)

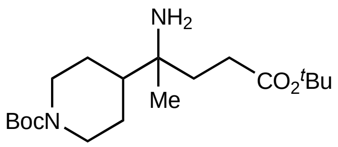

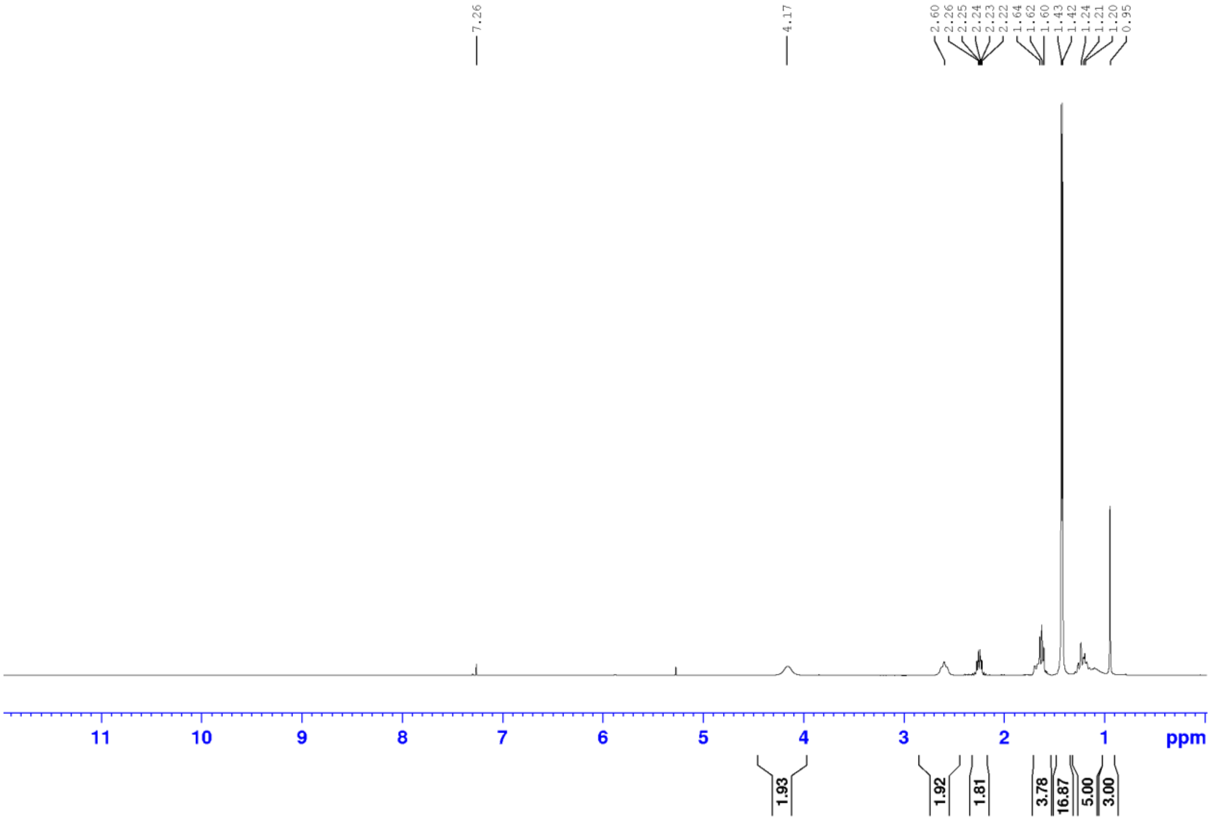

^{13}C NMR (101 MHz, $CDCl_3$)

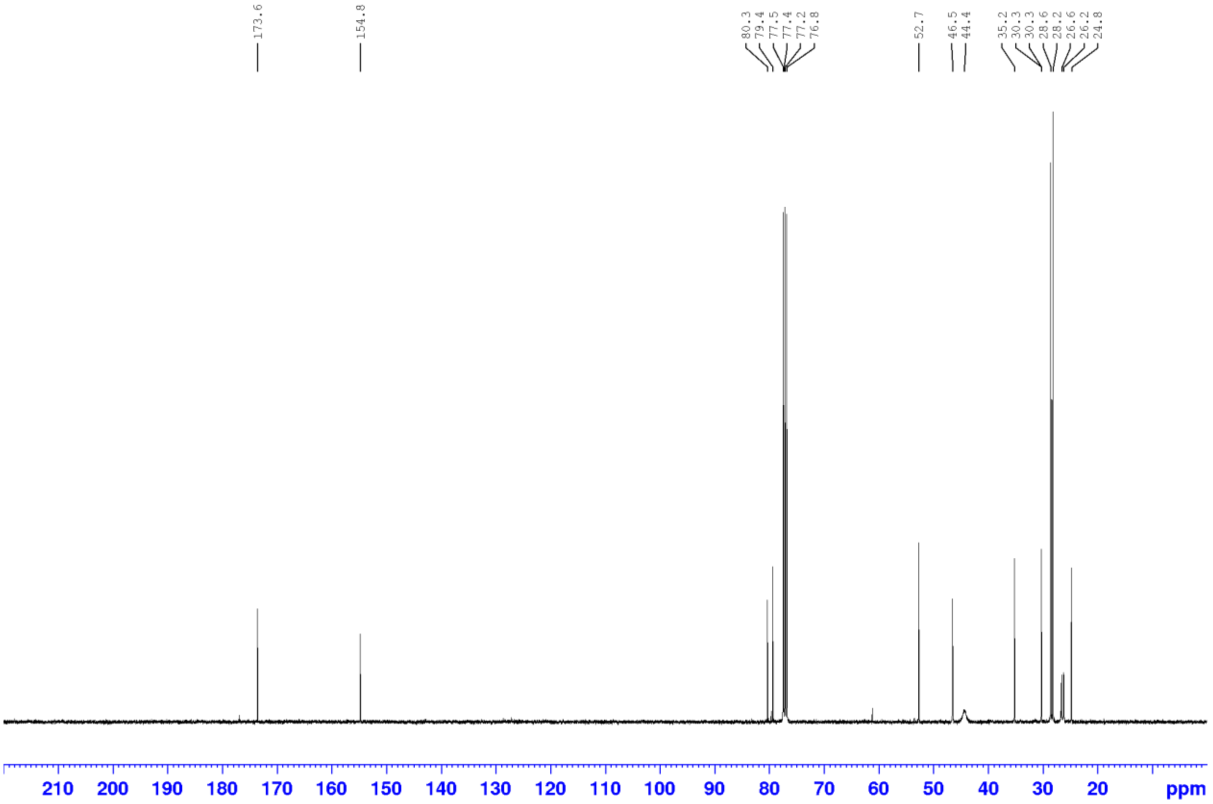

***tert*-butyl (*R*)-4-amino-4-(1,4-dioxaspiro[4.5]decan-8-yl)pentanoate, 3i** ^{1}H NMR (400 MHz, $CDCl_3$)

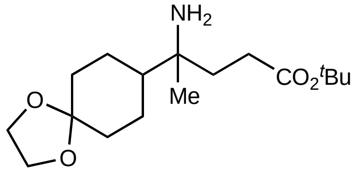

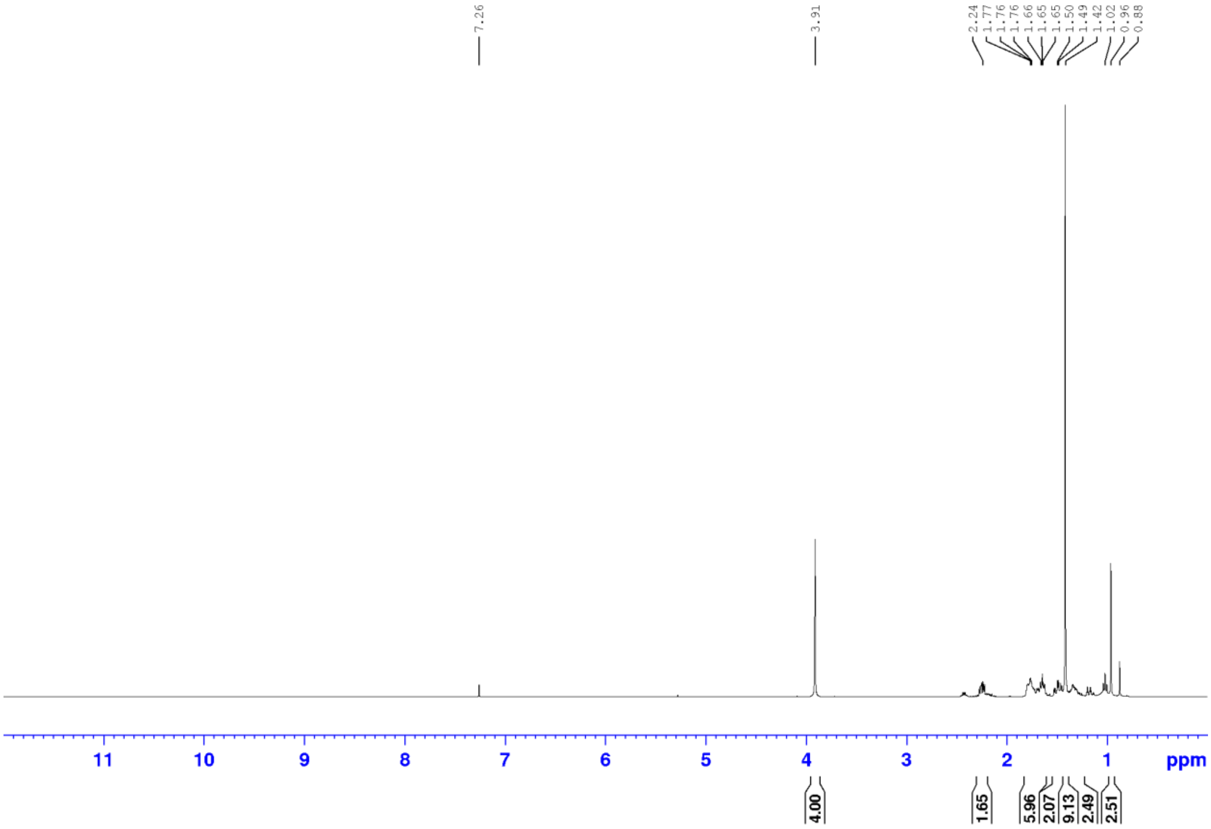

^{13}C NMR (101 MHz, $CDCl_3$)

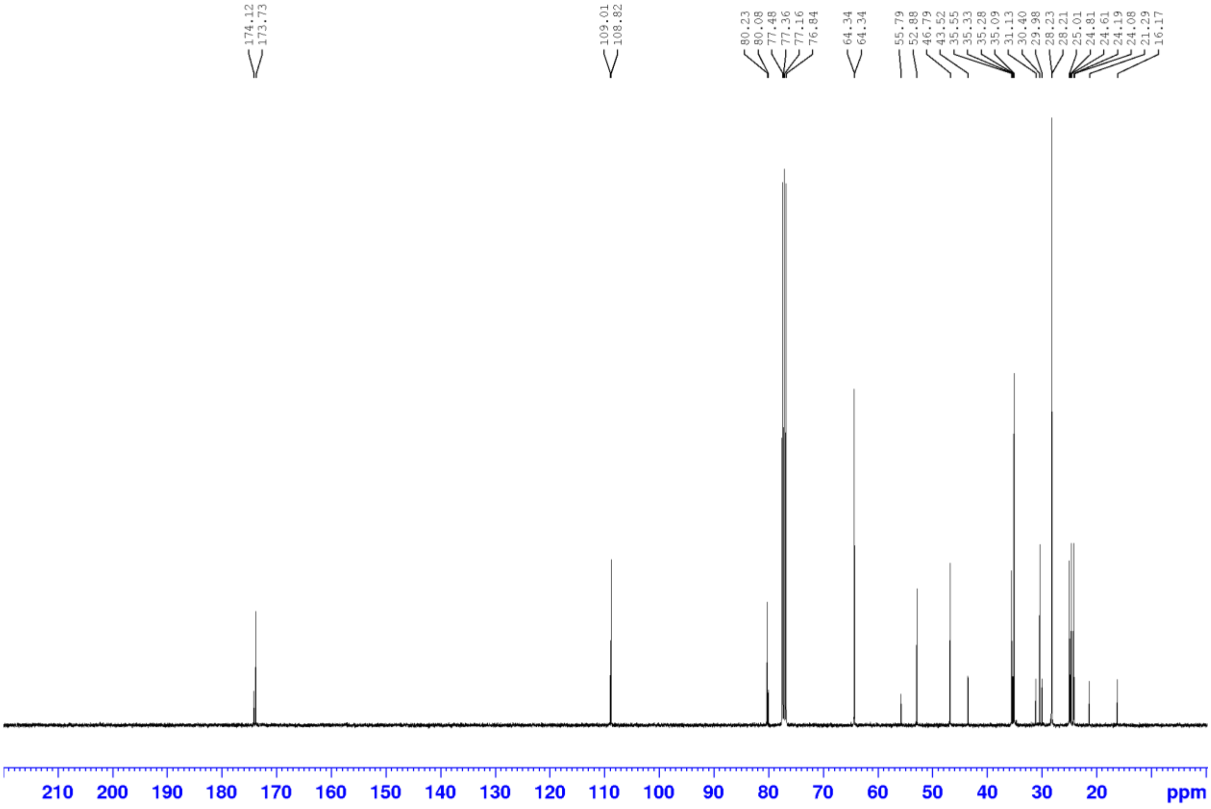

***tert*-butyl (*R*)-4-amino-4-(2,3-dihydro-1*H*-inden-2-yl)pentanoate, 3j** ^{1}H NMR (400 MHz, $CDCl_3$)

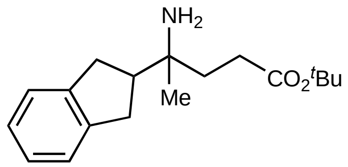

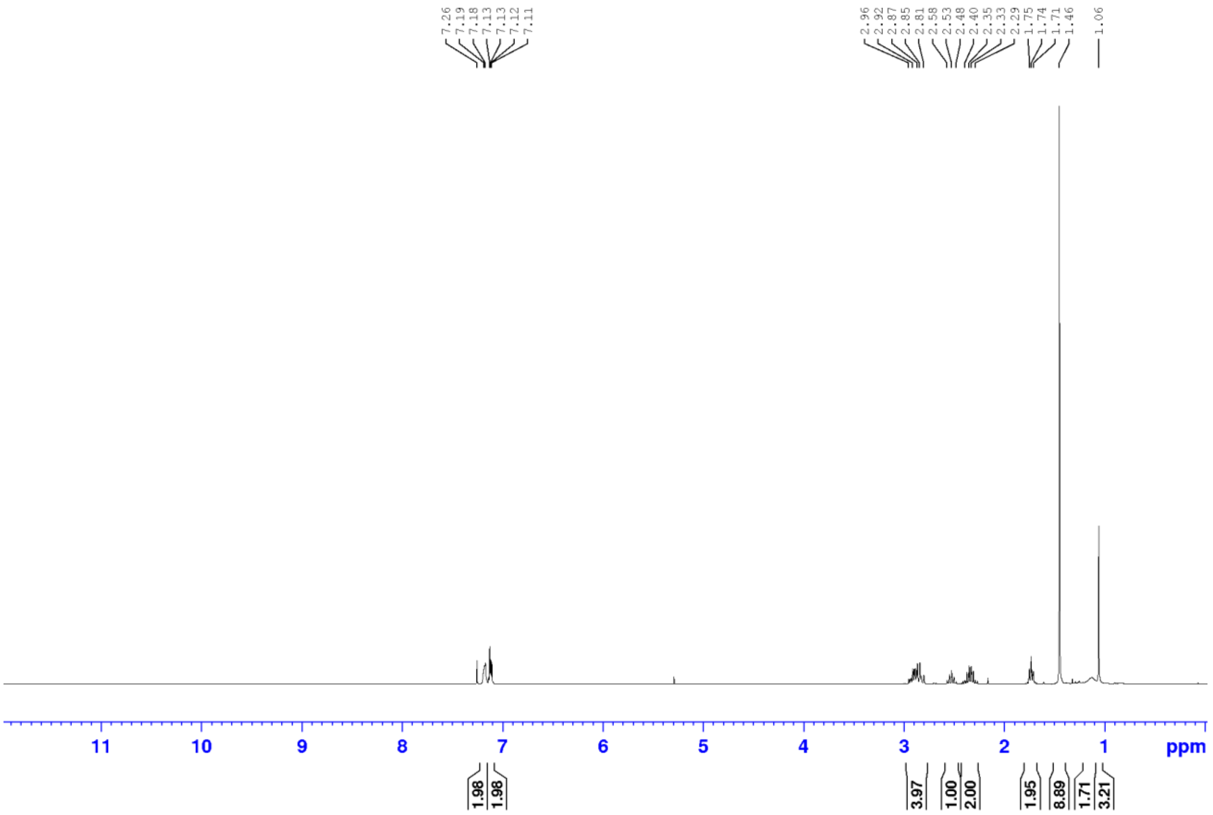

^{13}C NMR (101 MHz, $CDCl_3$)

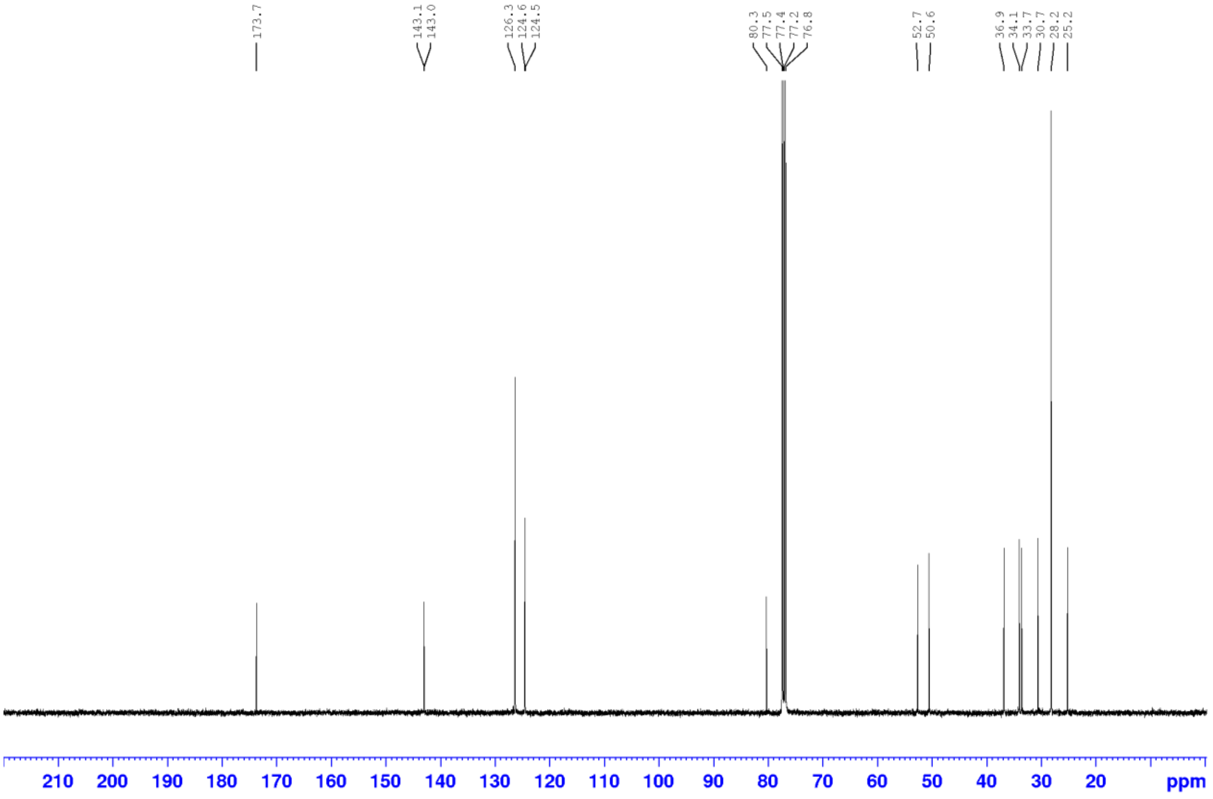

4-(Phenylsulfonyl)-2-(tetrahydro-2H-pyran-4-yl)butan-2-amine, 3k ^{1}H NMR (500 MHz, CDCl$_3$)

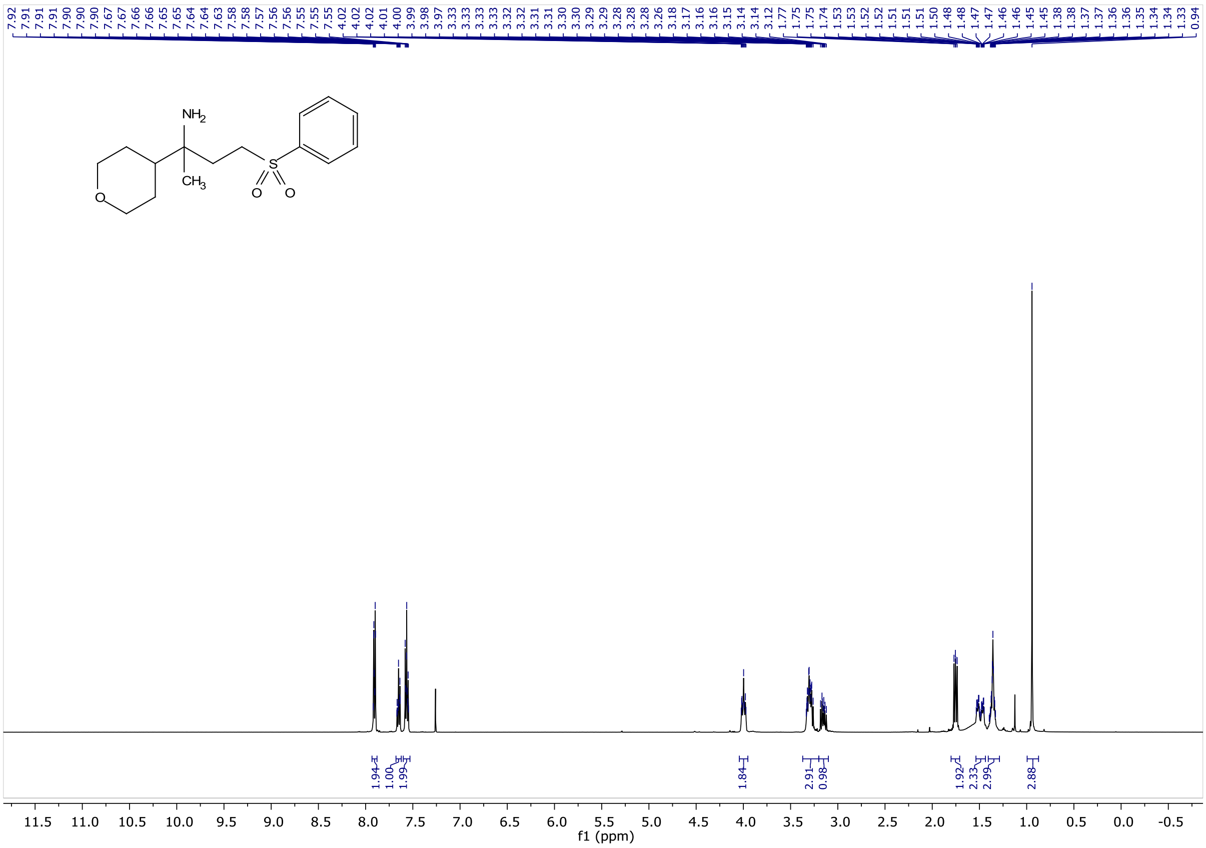

^{13}C NMR (126 MHz, CDCl$_3$)

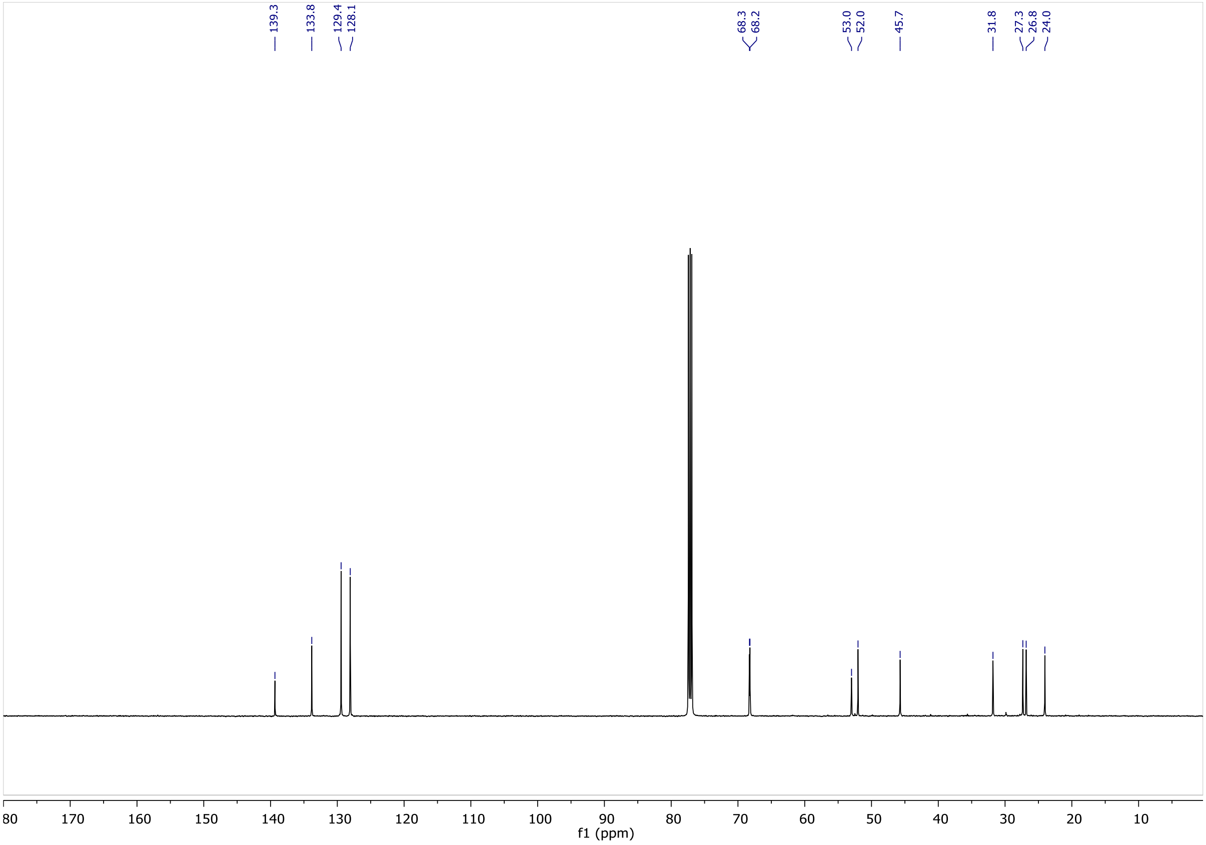

Diethyl (3-amino-3-(tetrahydro-*2H*-pyran-4-yl)butyl)phosphonate, 3l ^{1}H NMR (700 MHz, $CDCl_3$)

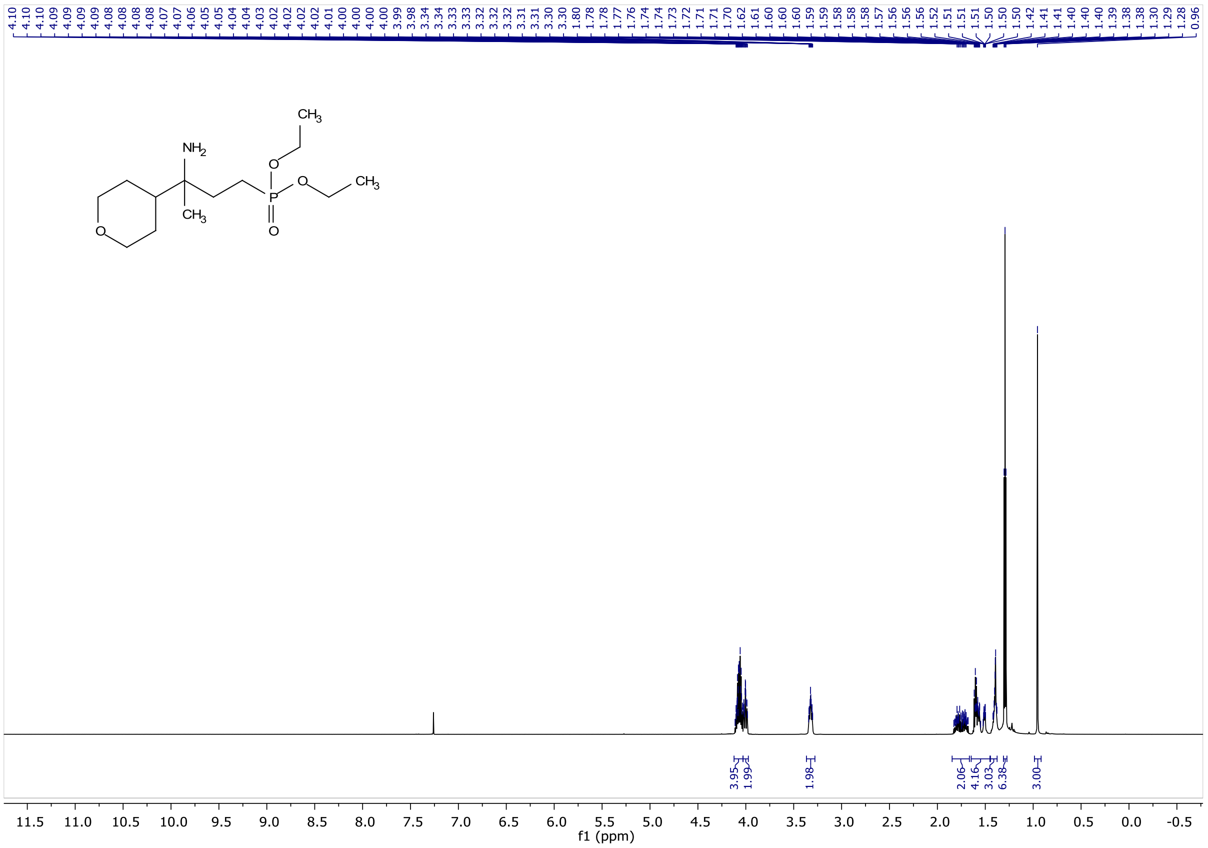

^{13}C NMR (126 MHz, $CDCl_3$)

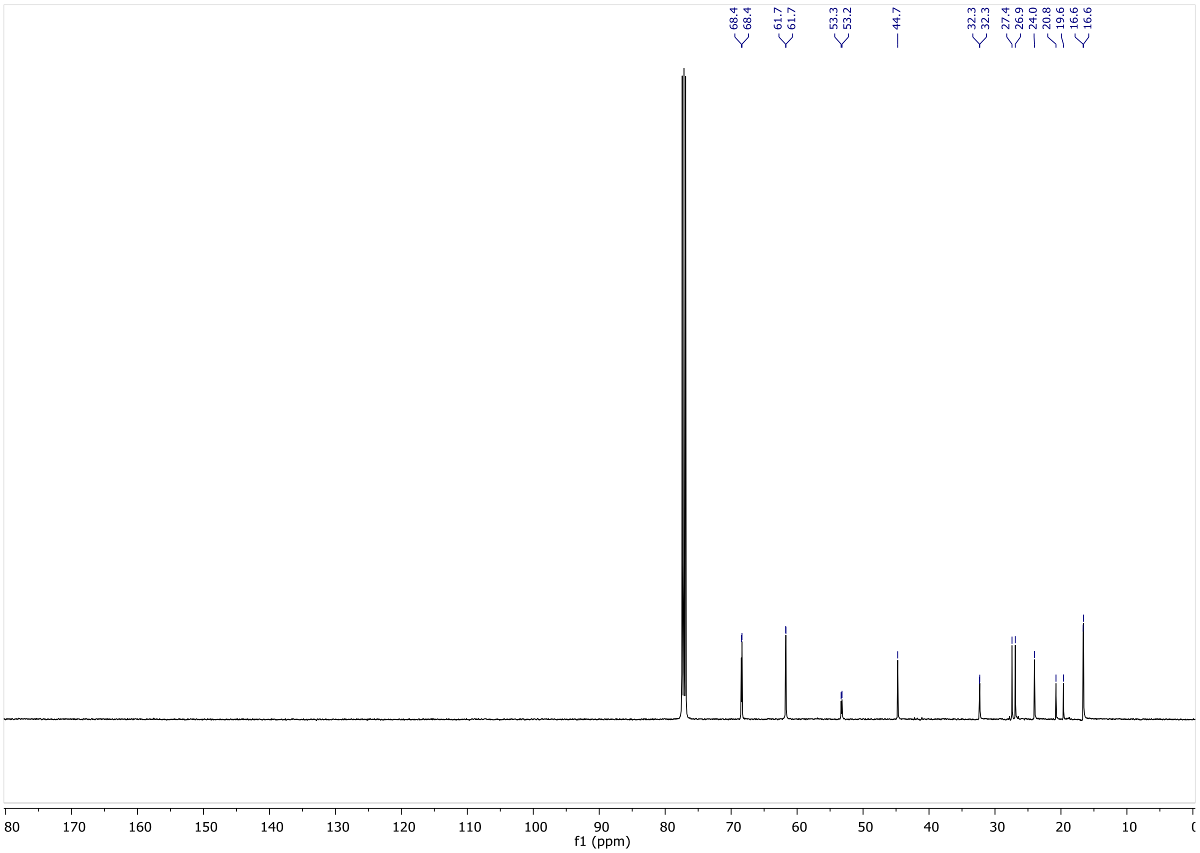

4-Amino-4-(tetrahydro-2H-pyran-4-yl)pentanenitrile, 3m ^{1}H NMR (700 MHz, $CDCl_3$)

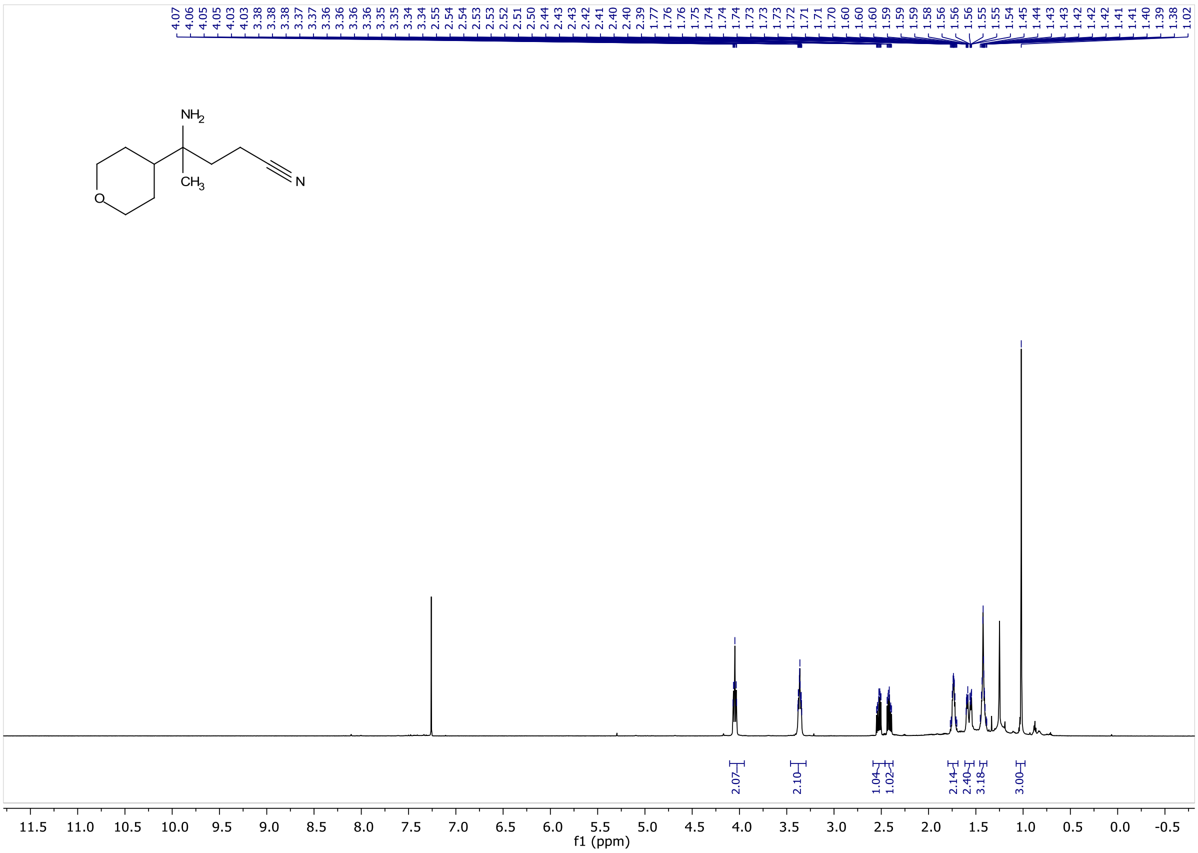

^{13}C NMR (101 MHz, $CDCl_3$)

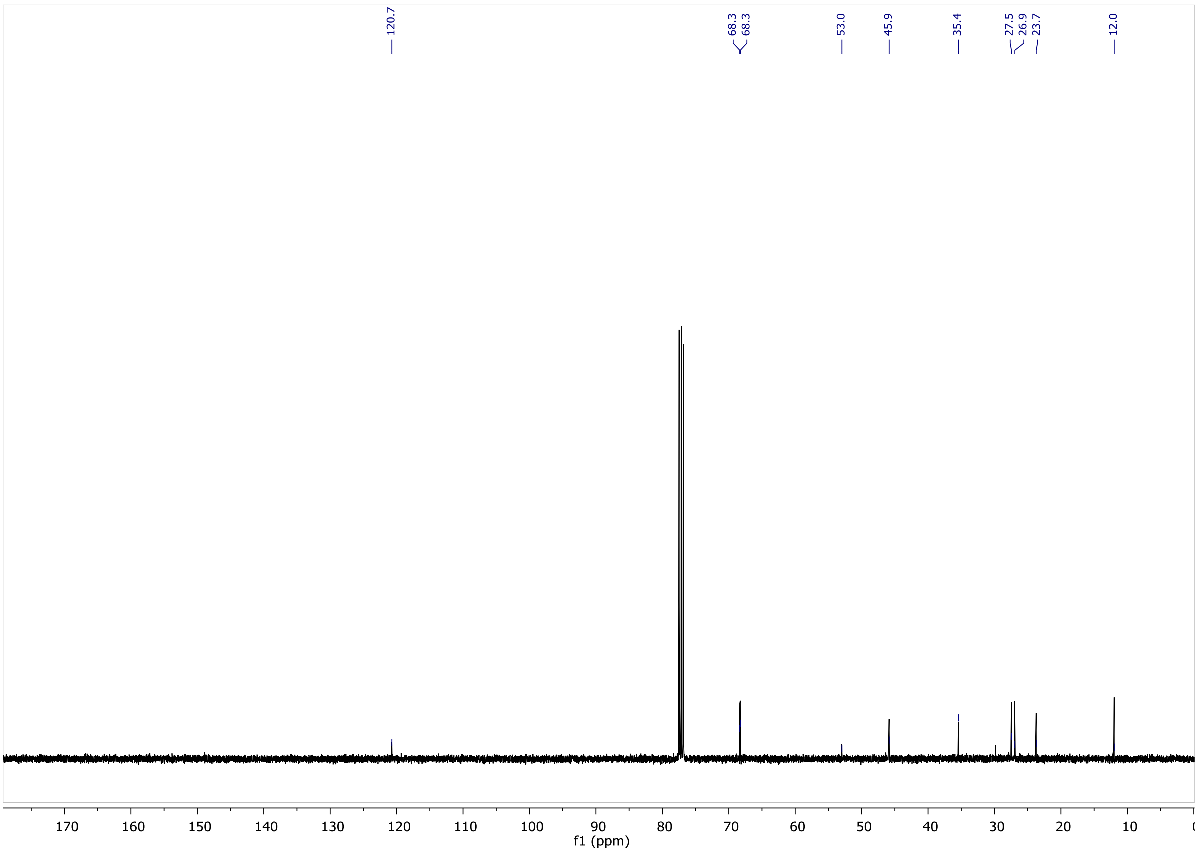

***tert*-Butyl 4-amino-4-(tetrahydro-2H-pyran-4-yl)pentanoate, 3n** ^{1}H NMR (500 MHz, $CDCl_3$)

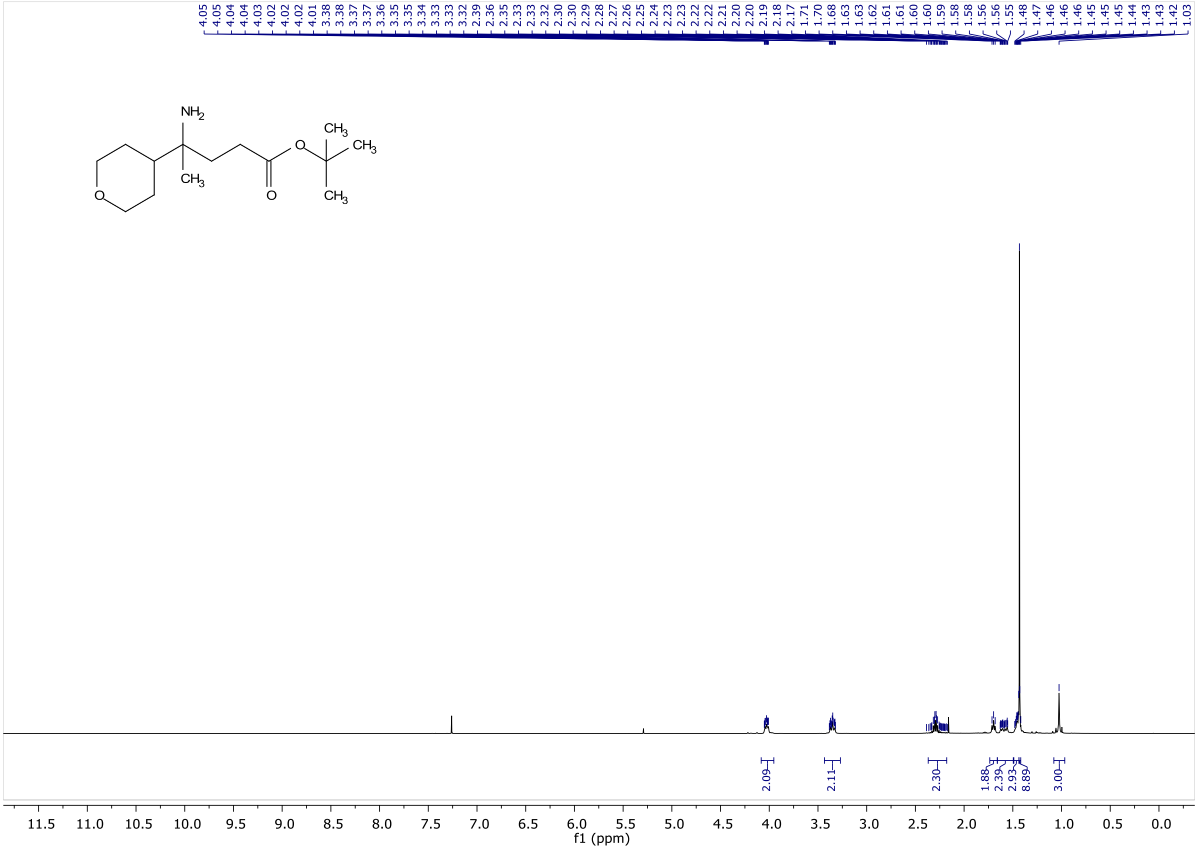

^{13}C NMR (126 MHz, $CDCl_3$)

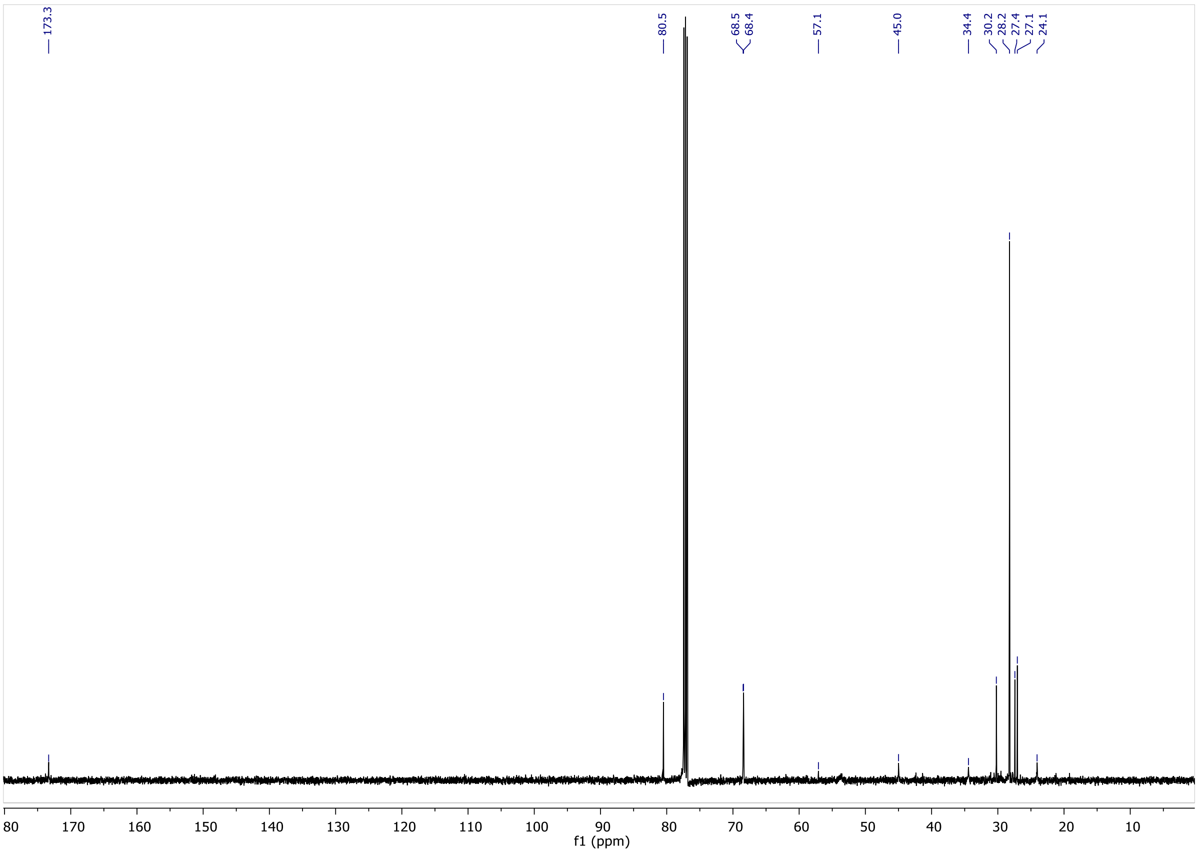

5,5,6,6,7,7,8,8,9,9,10,10,11,11,12,12,12-heptadecafluoro-2-(tetrahydro-*2H*-pyran-4-yl)dodecan-2-amine, 3o ^{1}H NMR (400 MHz, $CDCl_3$)

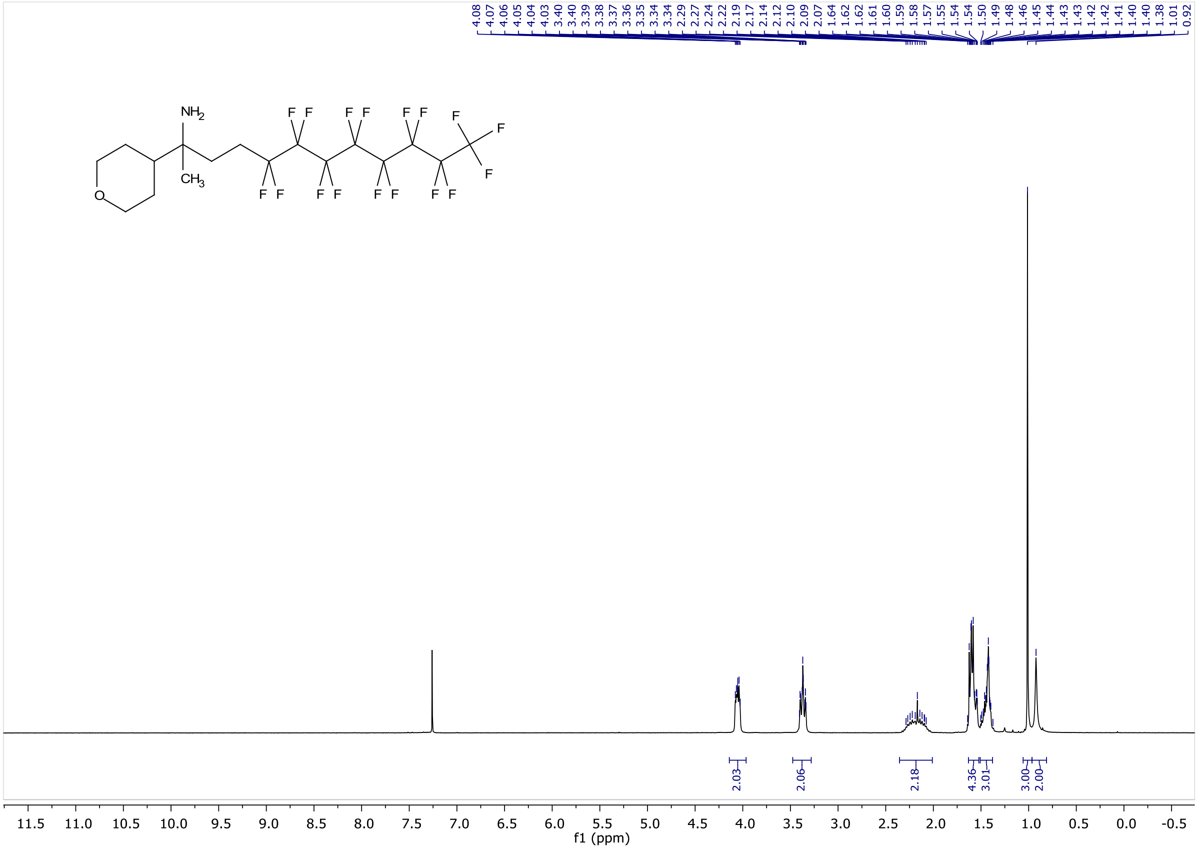

^{13}C NMR (101 MHz, $CDCl_3$)

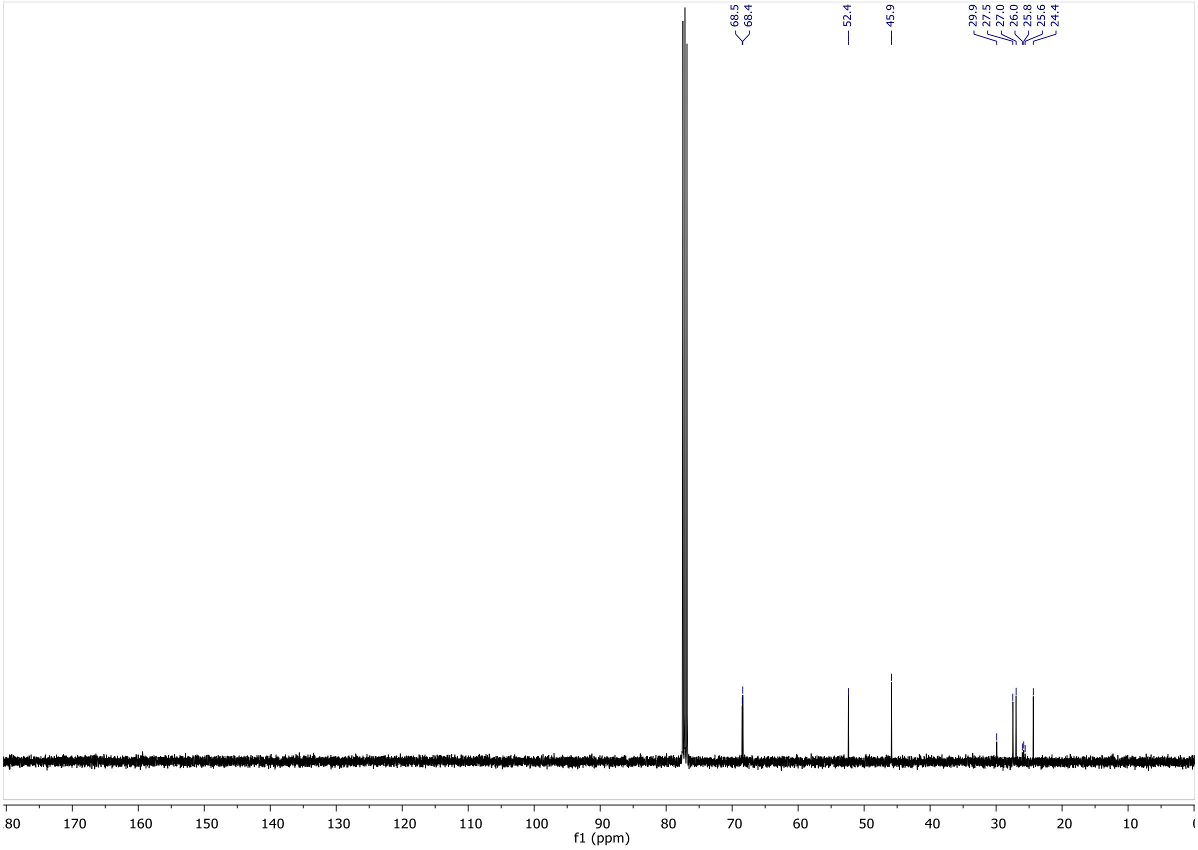

^{19}F NMR (376 MHz, $CDCl_3$)

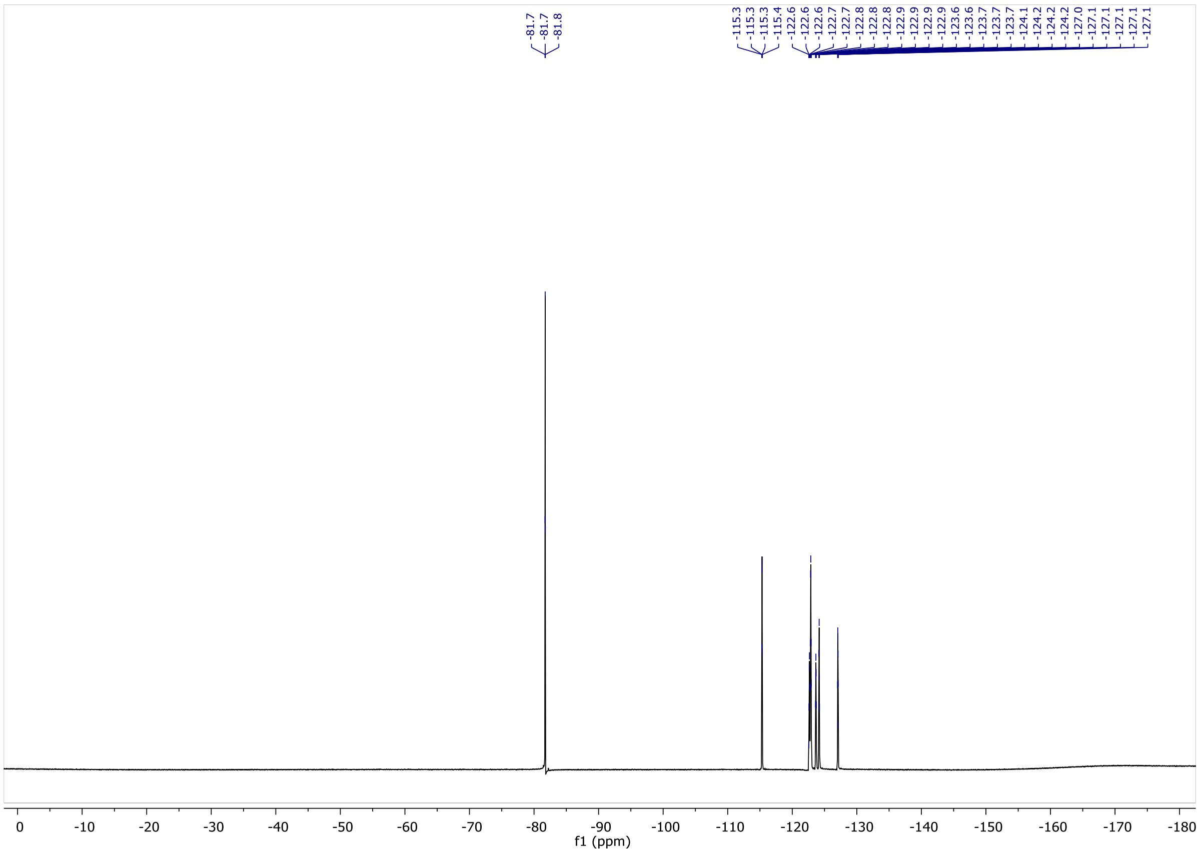

3-amino-N-methyl-N-(3-phenyl-3-(4-(trifluoromethyl)phenoxy)propyl)-3-(tetrahydro-2H-pyran-4-yl)butane-1-sulfonamide, 3p ^{1}H NMR (400 MHz, $CDCl_3$)

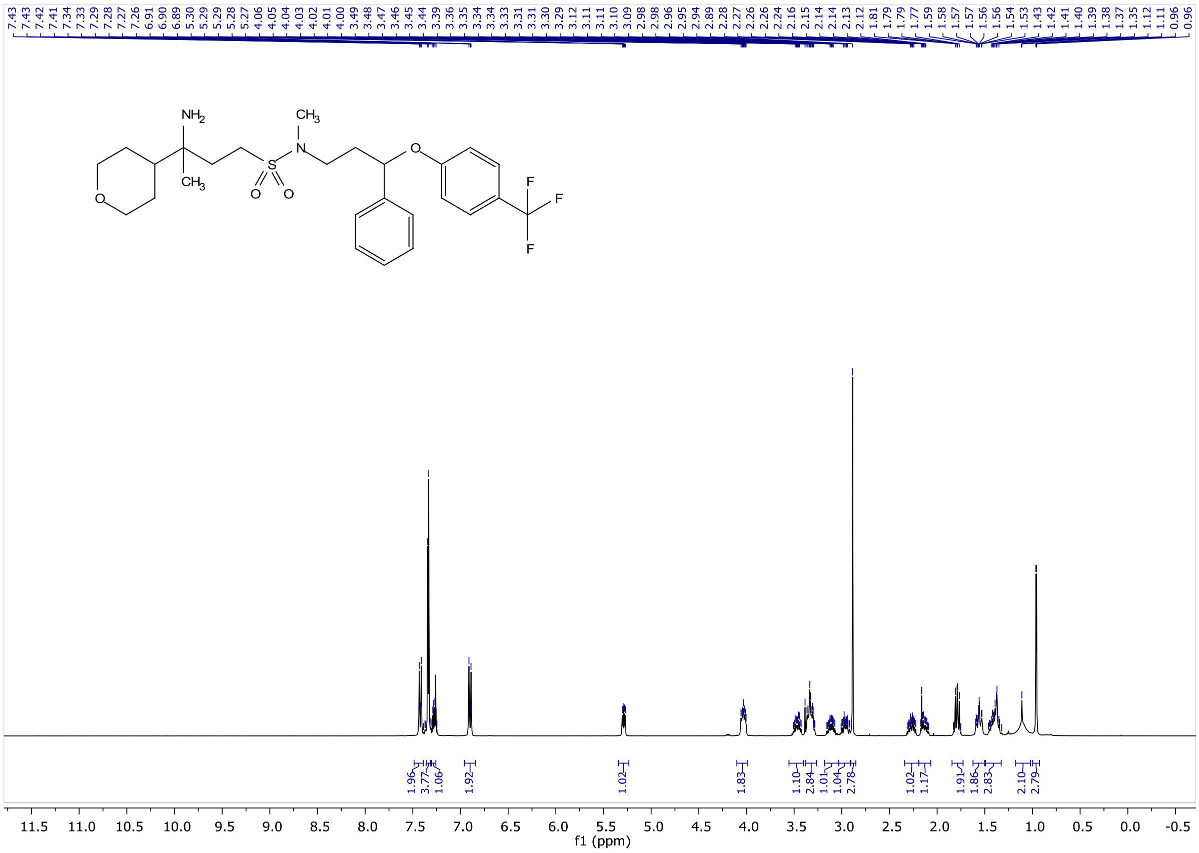

^{13}C NMR (101 MHz, $CDCl_3$)

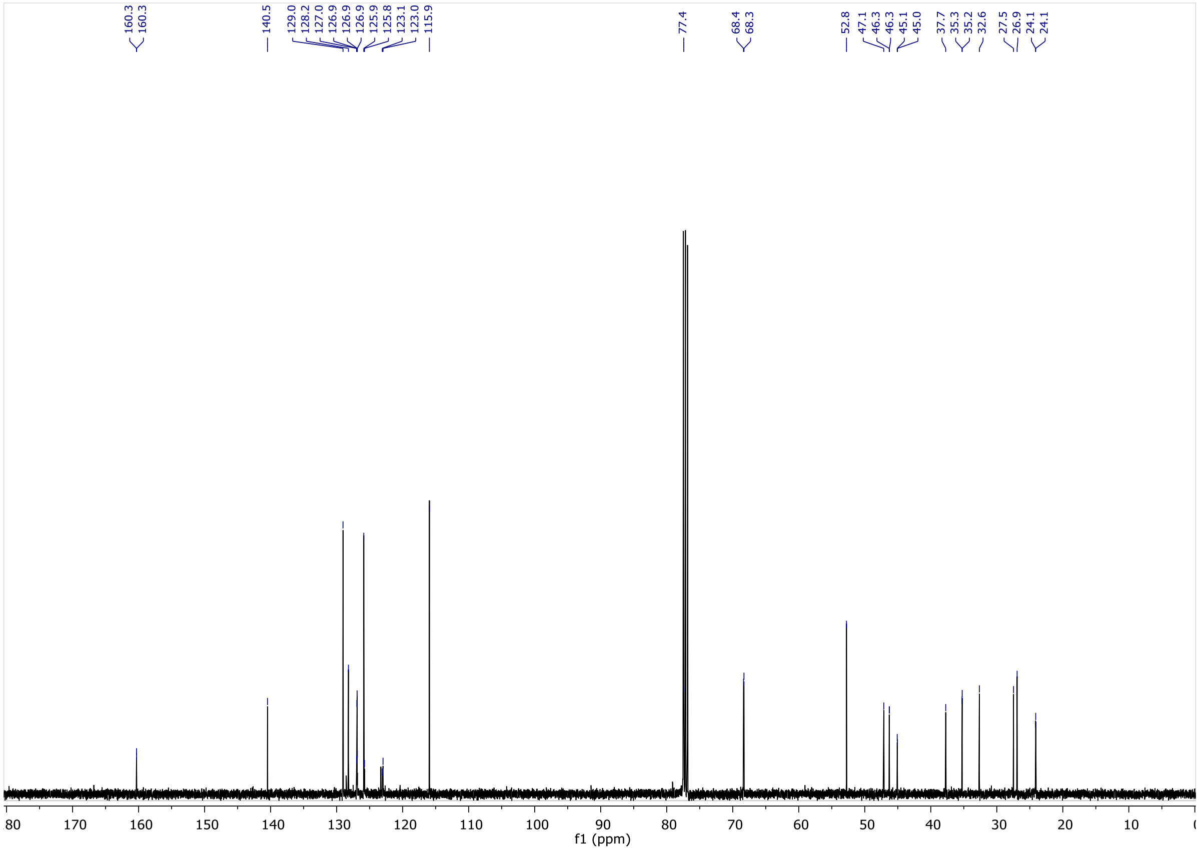

^{19}F NMR (376 MHz, $CDCl_3$)

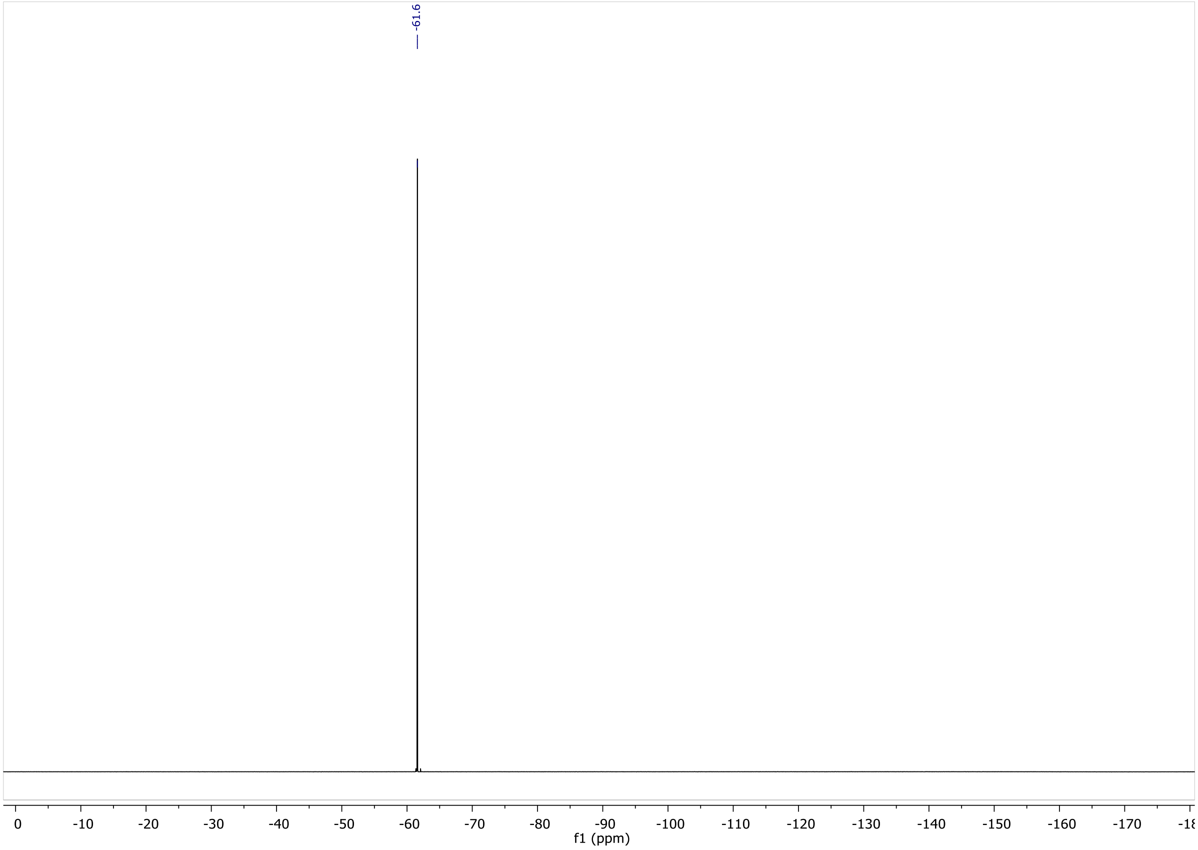

4-(morpholinosulfonyl)-2-(tetrahydro-2H-pyran-4-yl)butan-2-amine, 3q ^{1}H NMR (500 MHz, $CDCl_3$)

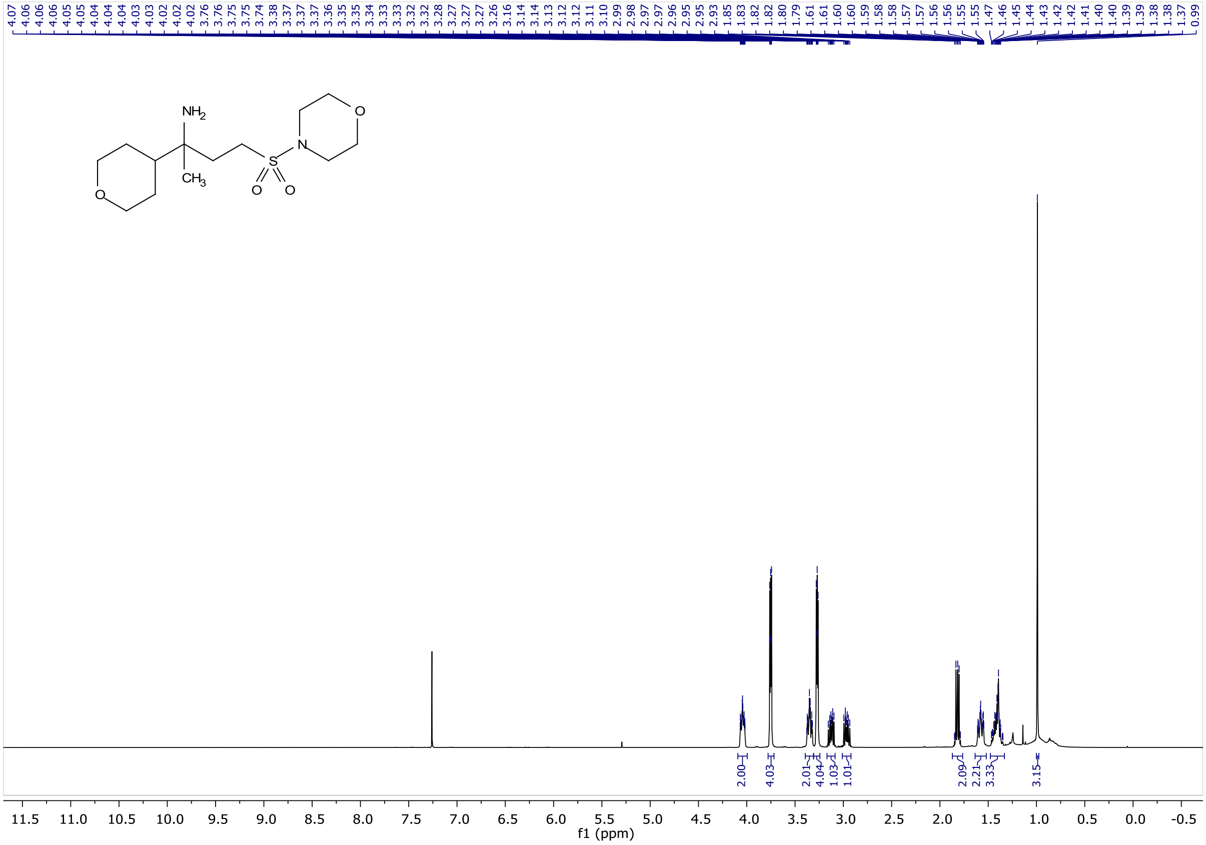

^{13}C NMR (126 MHz, $CDCl_3$)

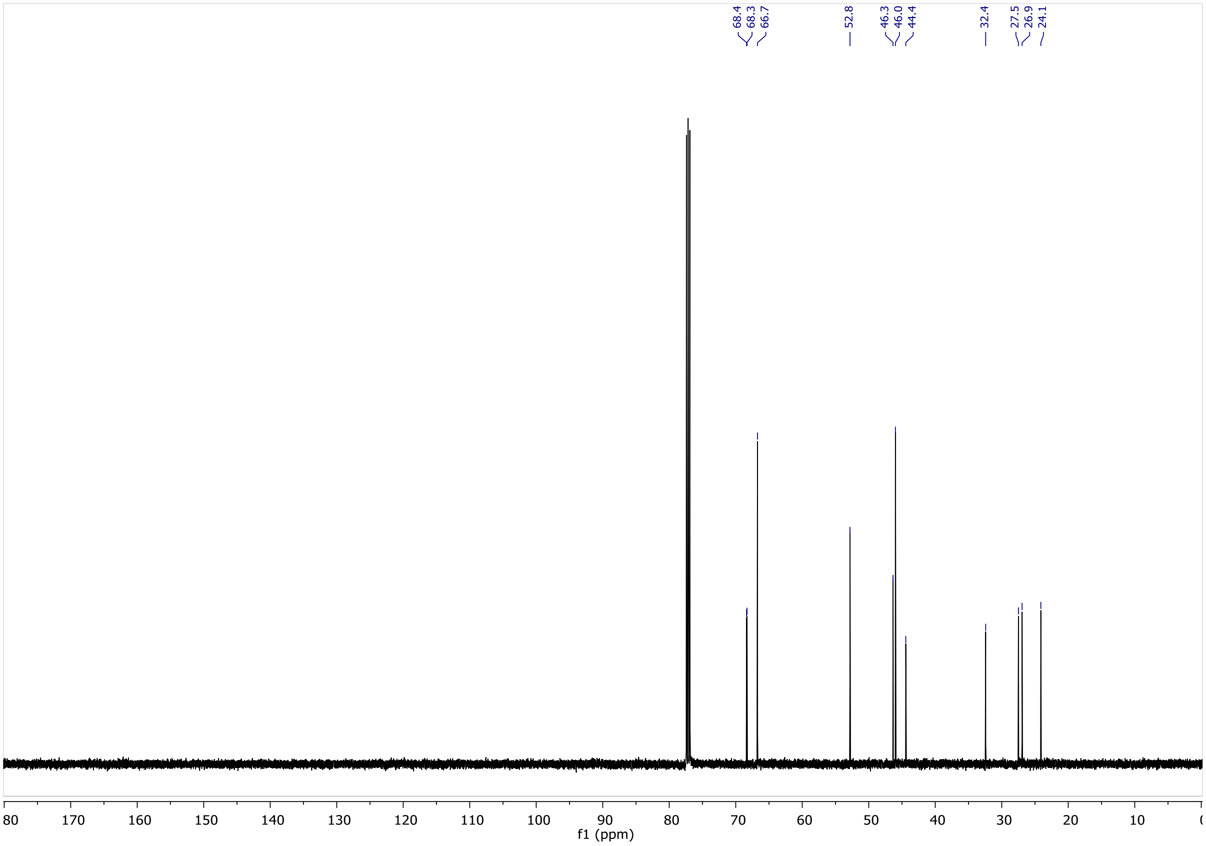

4-((4-(6-fluorobenzo[d]isoxazol-3-yl)piperidin-1-yl)sulfonyl)-2-(tetrahydro-2H-pyran-4-yl)butan-2-amine, 3r ^{1}H NMR (400 MHz, $CDCl_3$)

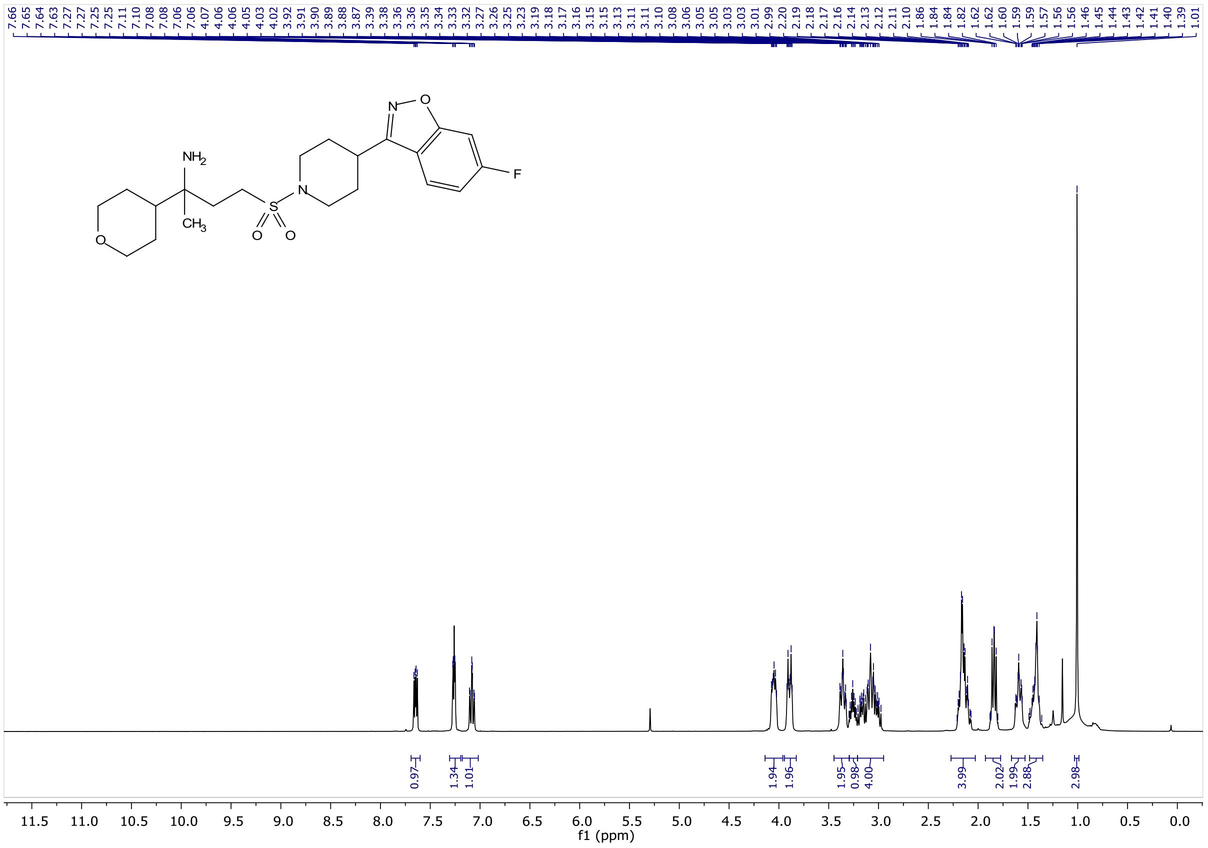

^{13}C NMR (101 MHz, $CDCl_3$)

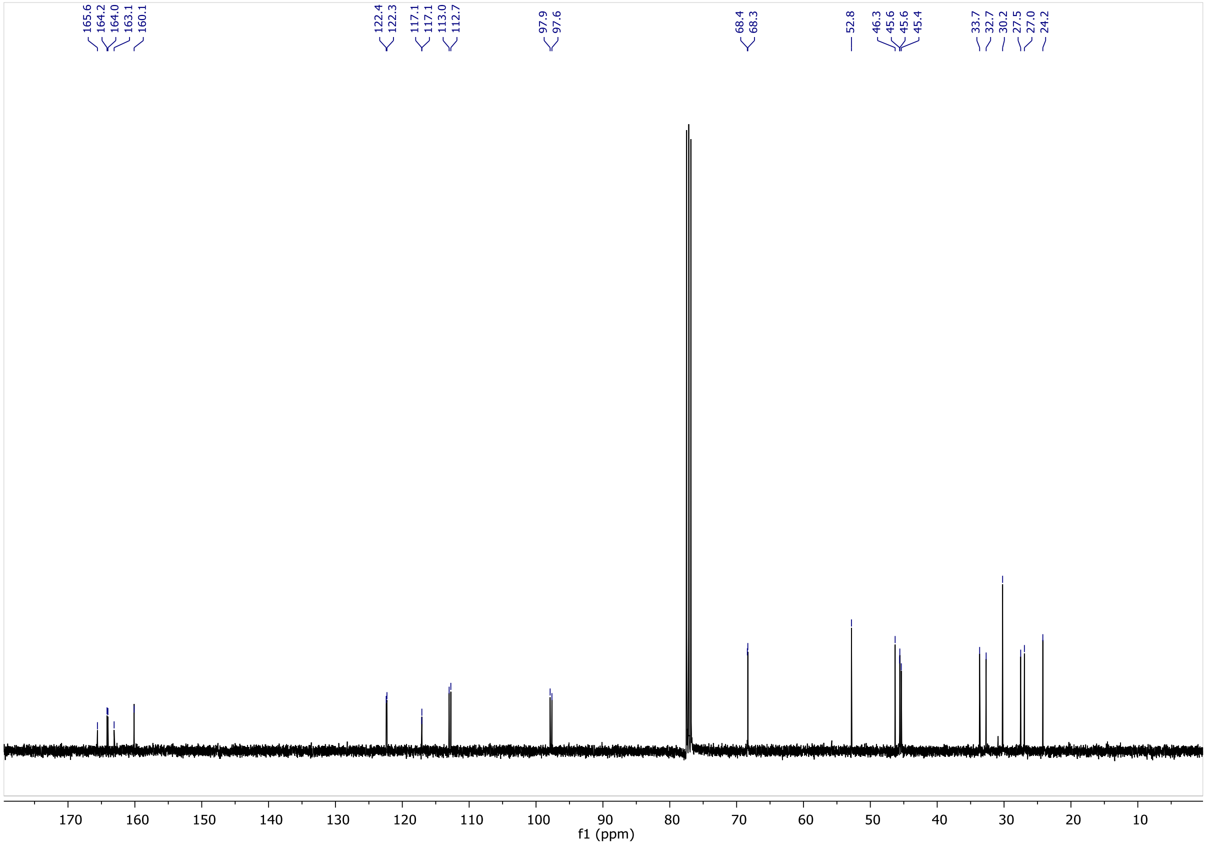

^{19}F NMR (376 MHz, $CDCl_3$)

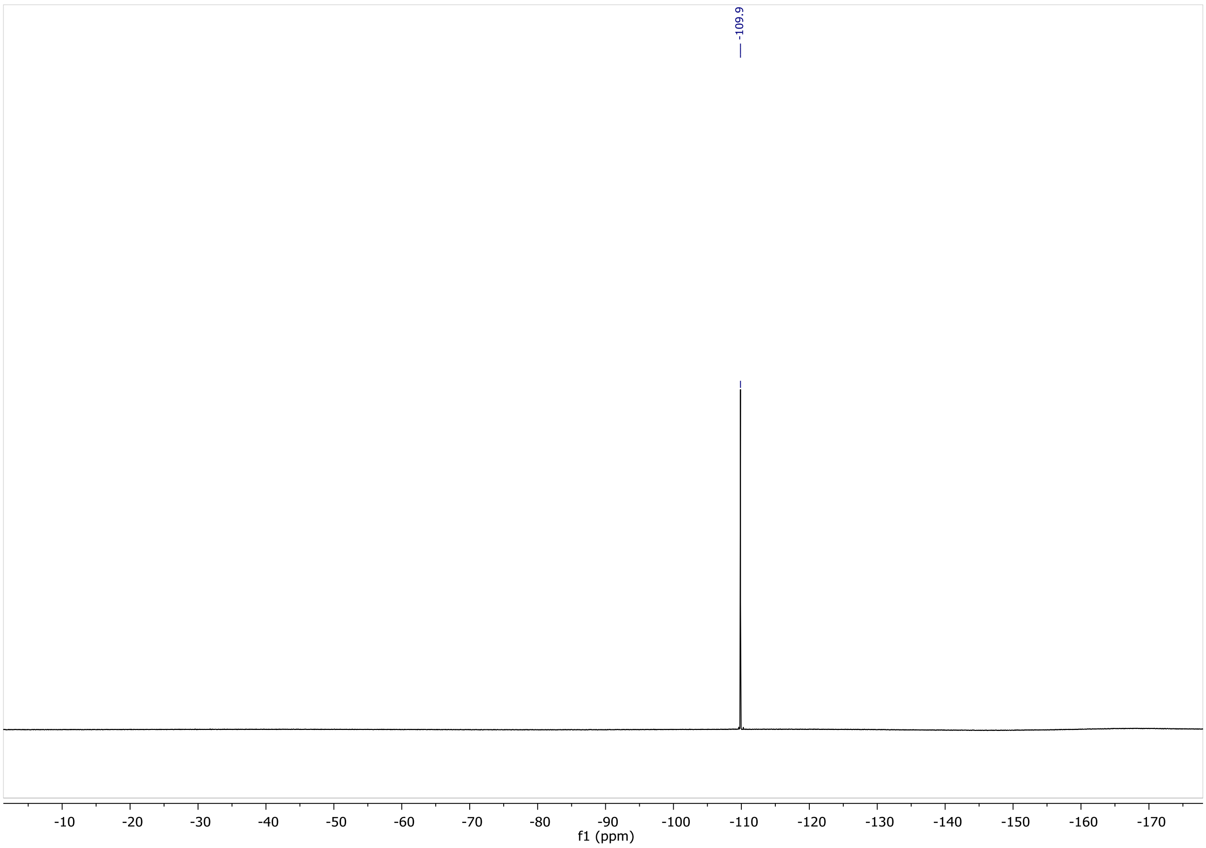

***tert*-Butyl 4-amino-2-methyl-4-(tetrahydro-2H-pyran-4-yl)pentanoate, 3s** ^{1}H NMR (500 MHz, $CDCl_3$)

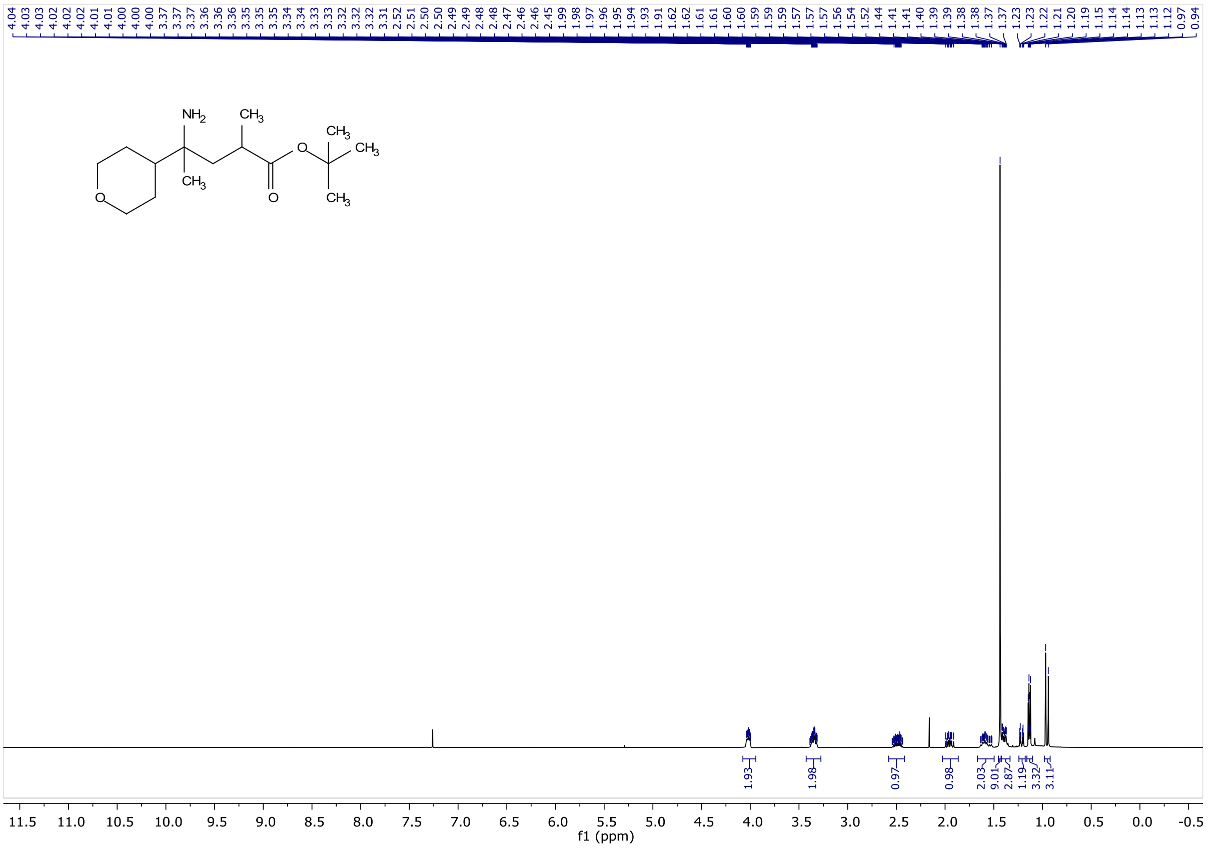

^{13}C NMR (126 MHz, $CDCl_3$)

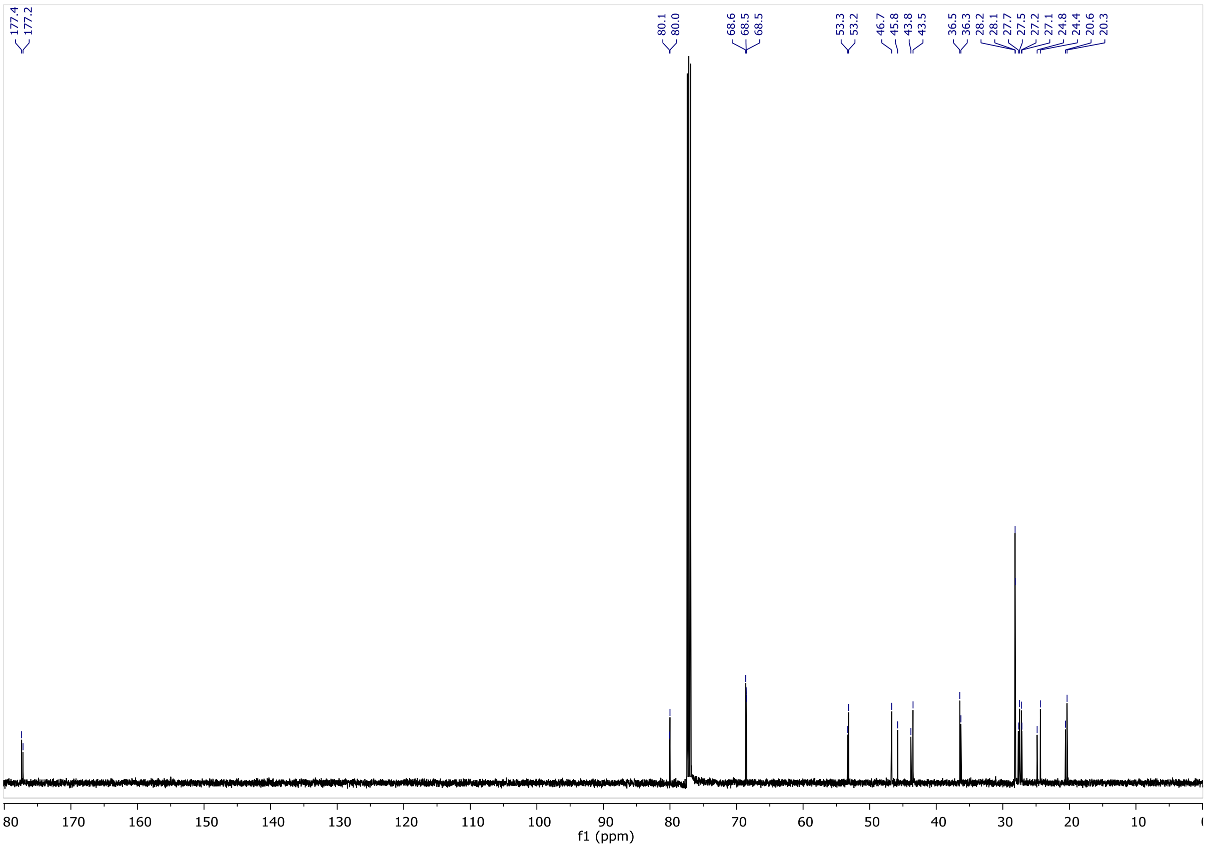

(R)-2-methoxy-1-phenylethan-1-amine, 4a ^{1}H NMR (400 MHz, $CDCl_3$)

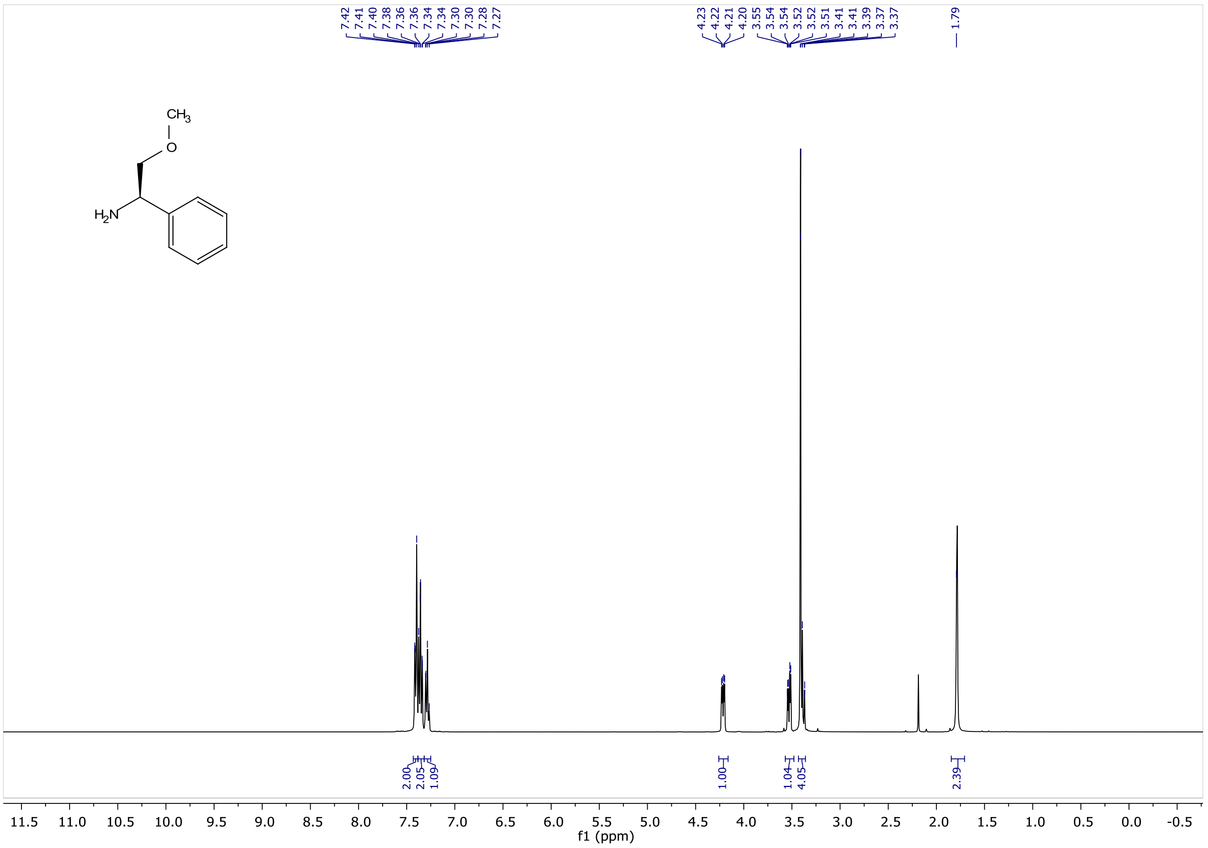

^{13}C NMR (101 MHz, $CDCl_3$)

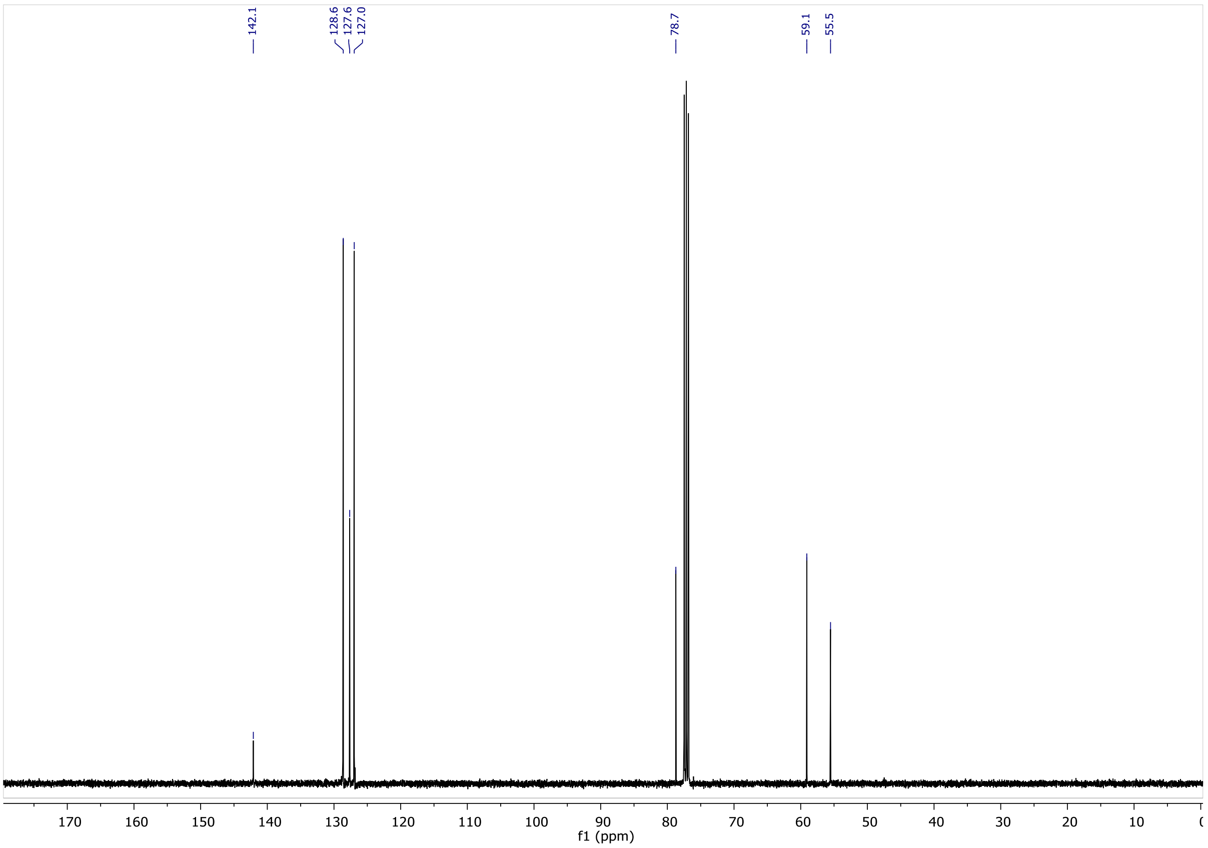

***tert*-Butyl (R)-(2-hydroxy-1-(4-hydroxyphenyl)ethyl)carbamate** ^{1}H NMR (400 MHz, MeOD)

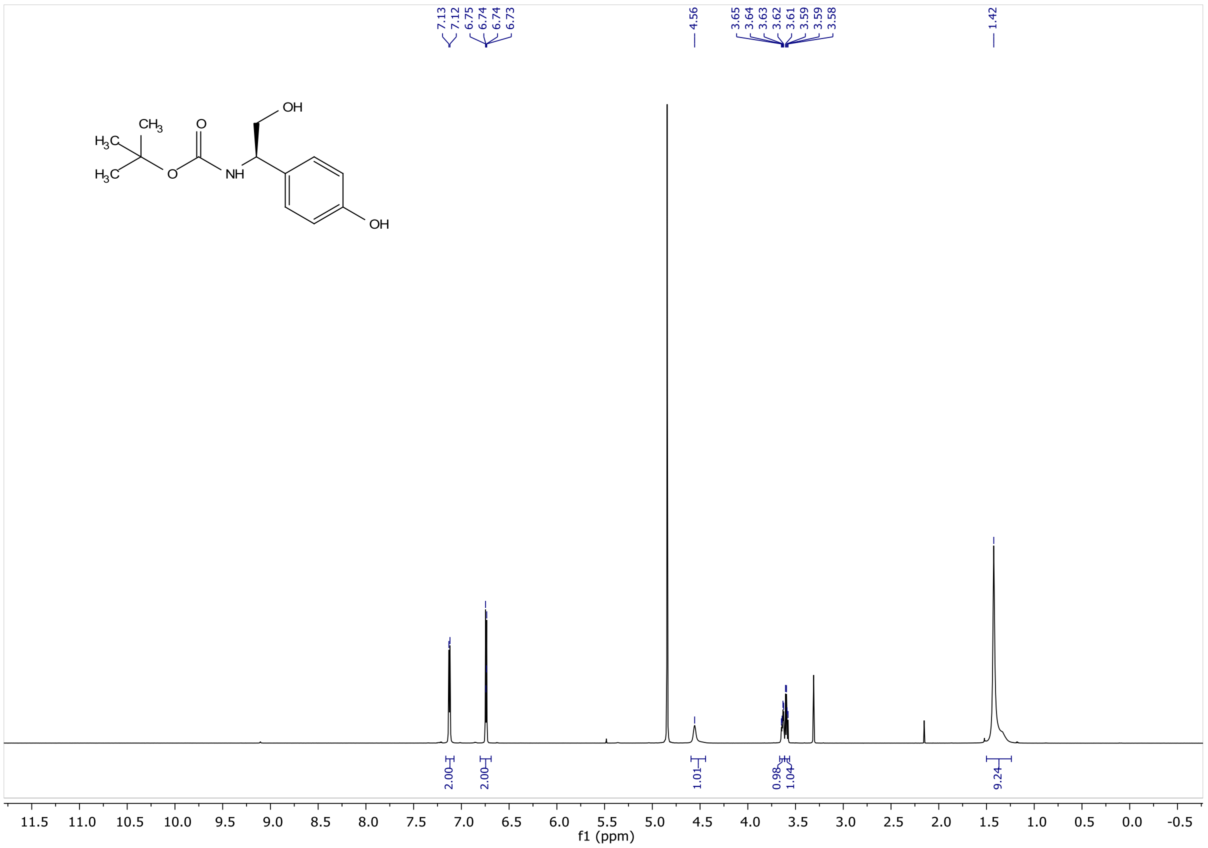

^{13}C NMR (176 MHz, DMSO)

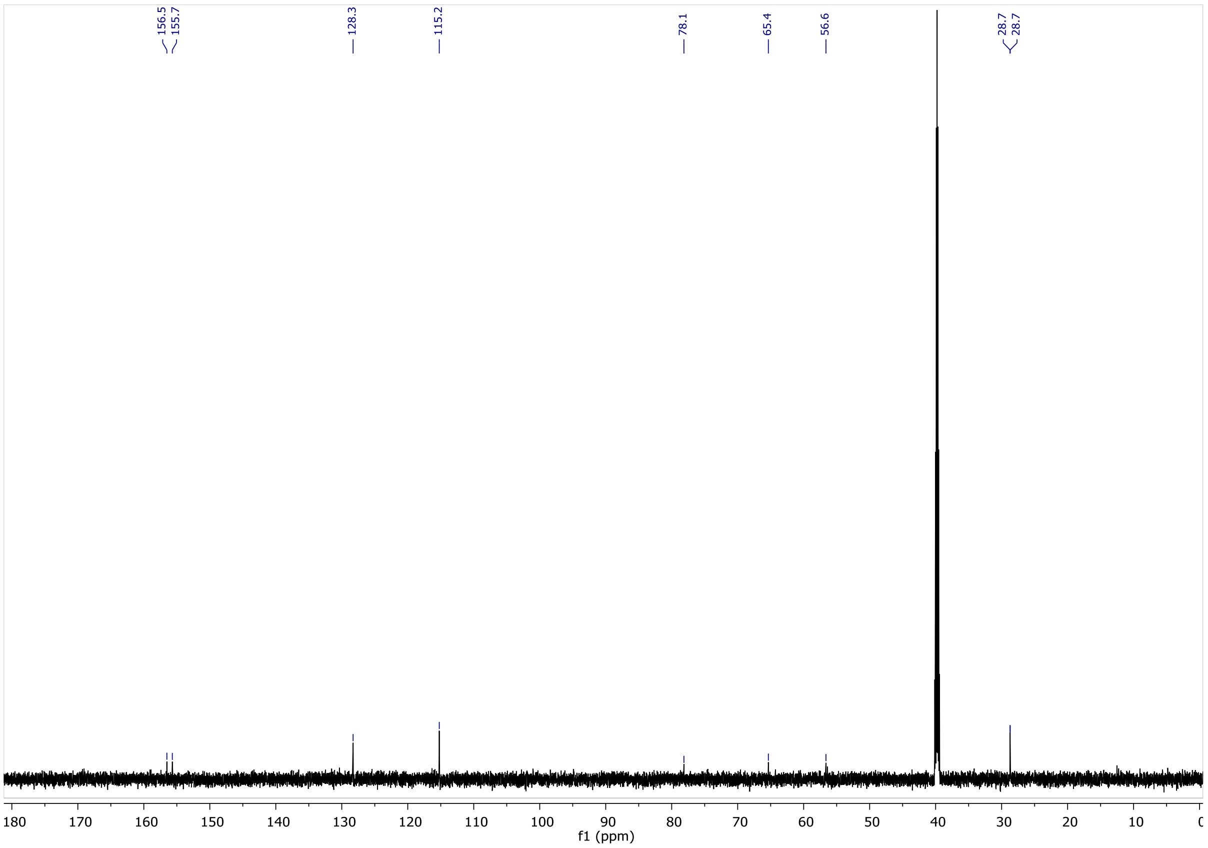

***tert*-Butyl (R)-(2-methoxy-1-(4-methoxyphenyl)ethyl)carbamate** ^{1}H NMR (700 MHz, $CDCl_3$)

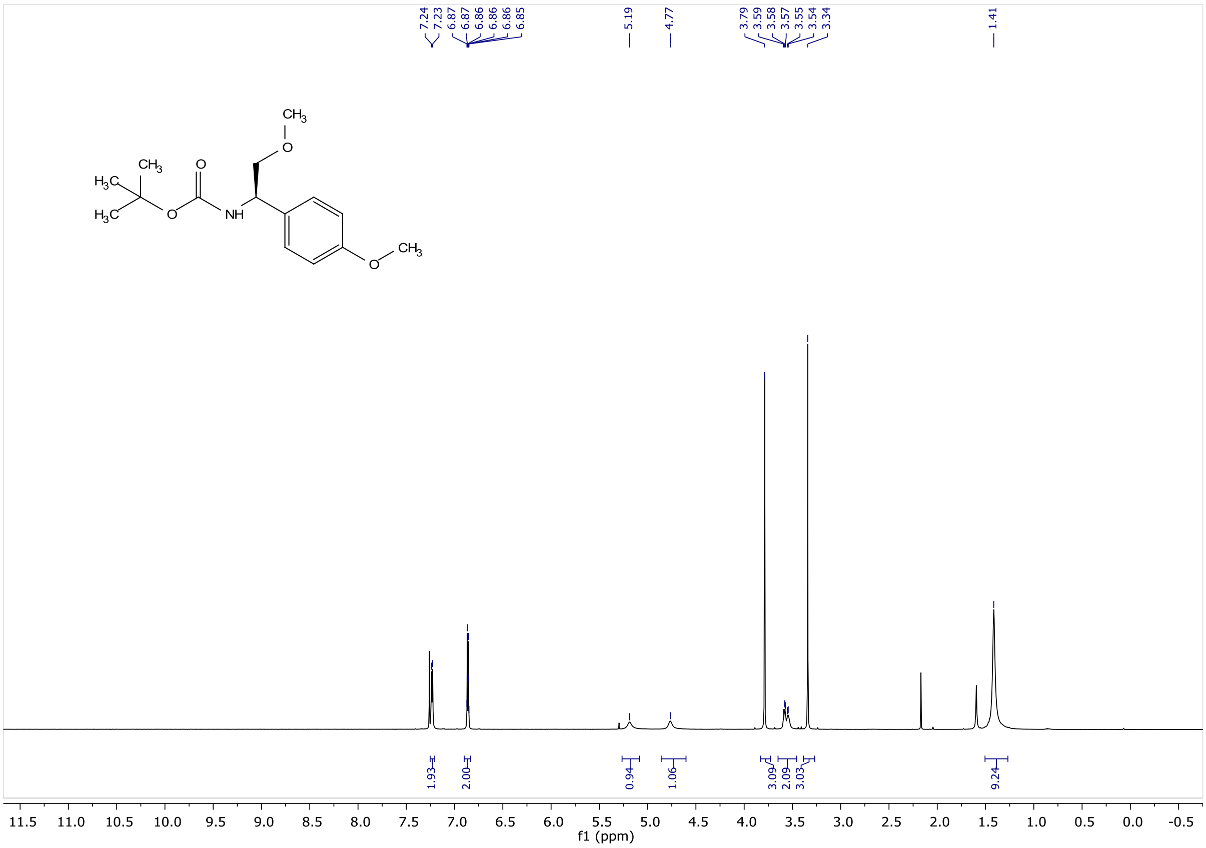

^{13}C NMR (101 MHz, $CDCl_3$)

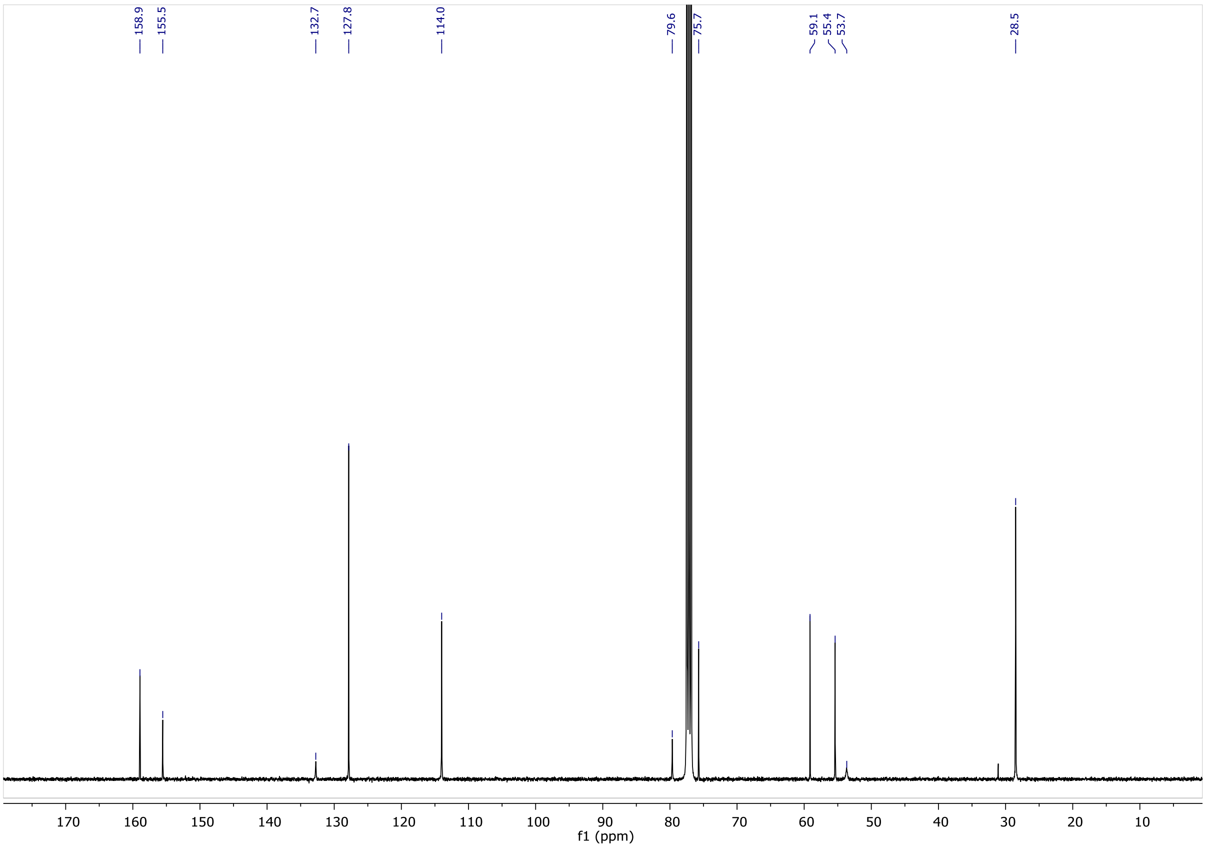

(R)-2-((4-methoxybenzyl)oxy)-1-phenylethan-1-amine, 4c ^{1}H NMR (400 MHz, $CDCl_3$)

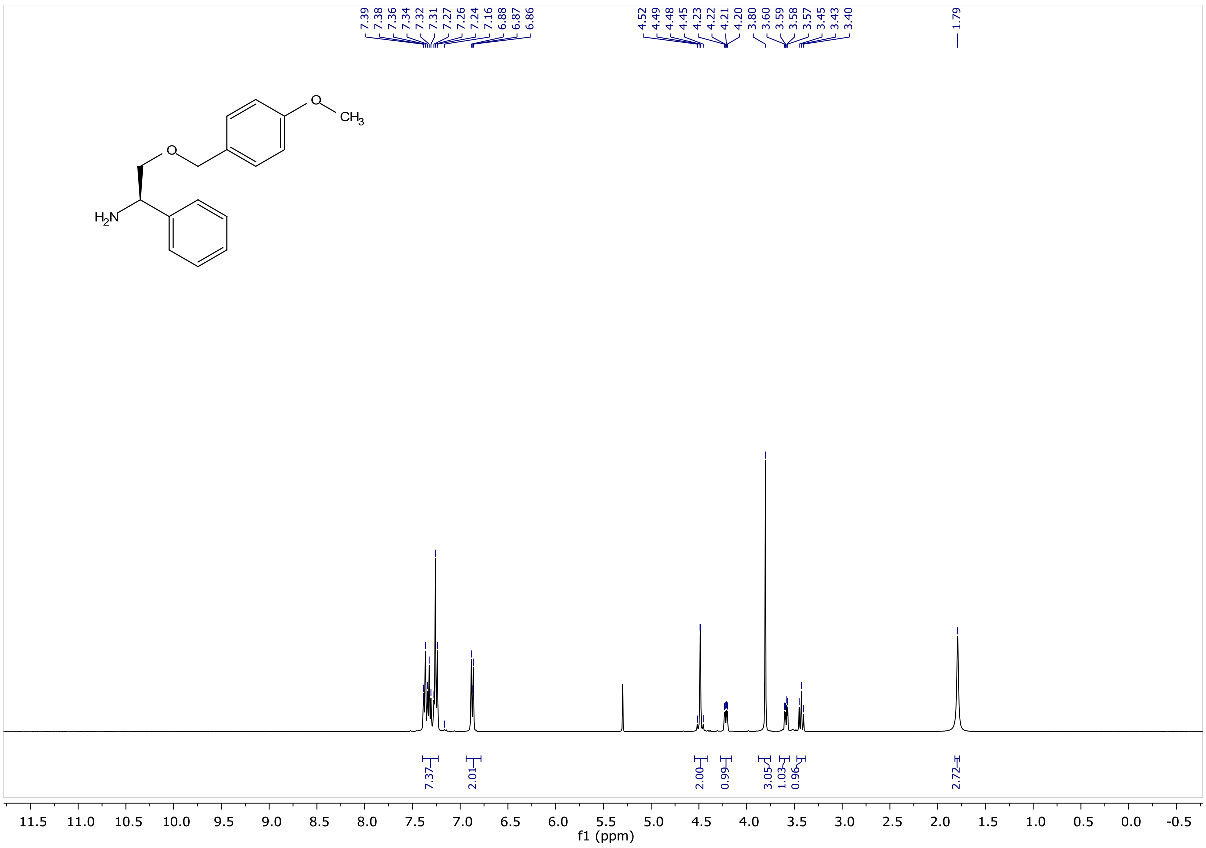

^{13}C NMR (101 MHz, $CDCl_3$)

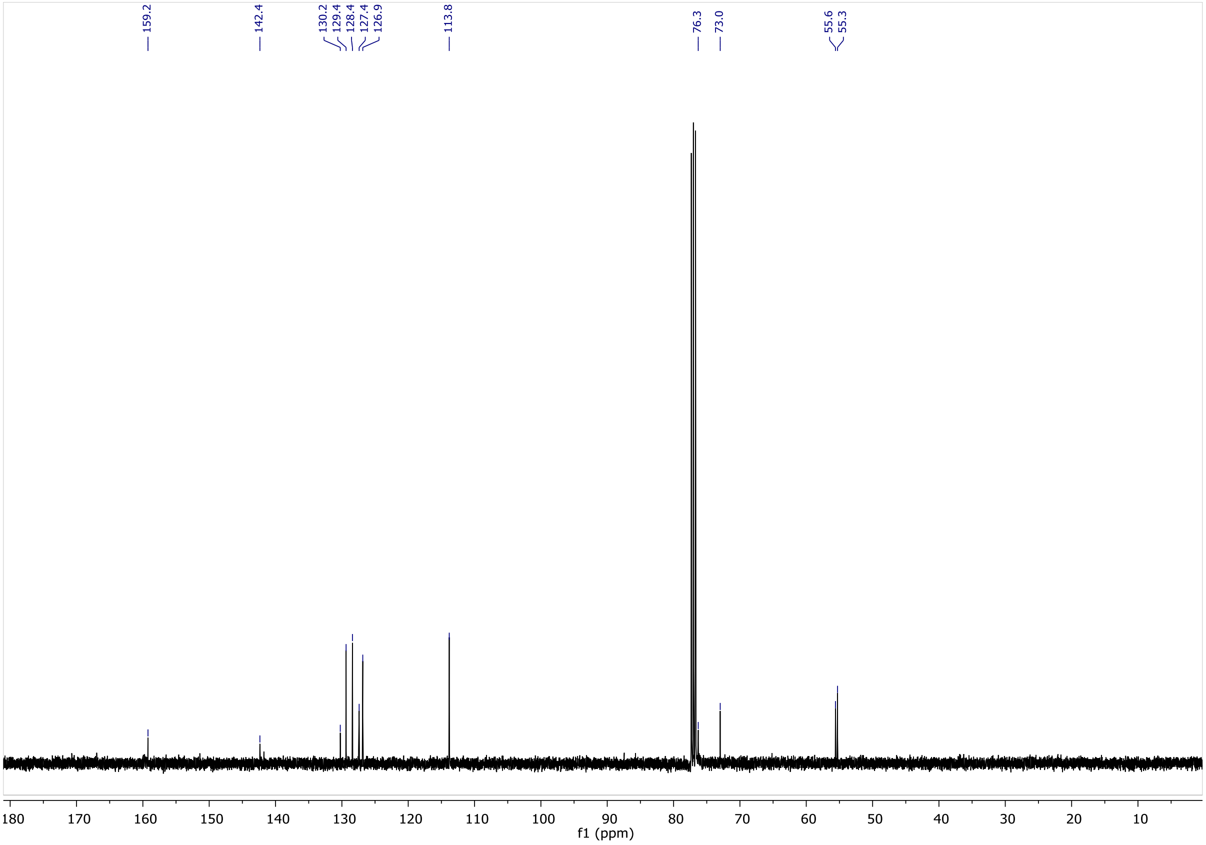

(R)-2-methoxy-1-(4-methoxyphenyl)ethan-1-amine, 4d ^{1}H NMR (400 MHz, CDCl$_3$)

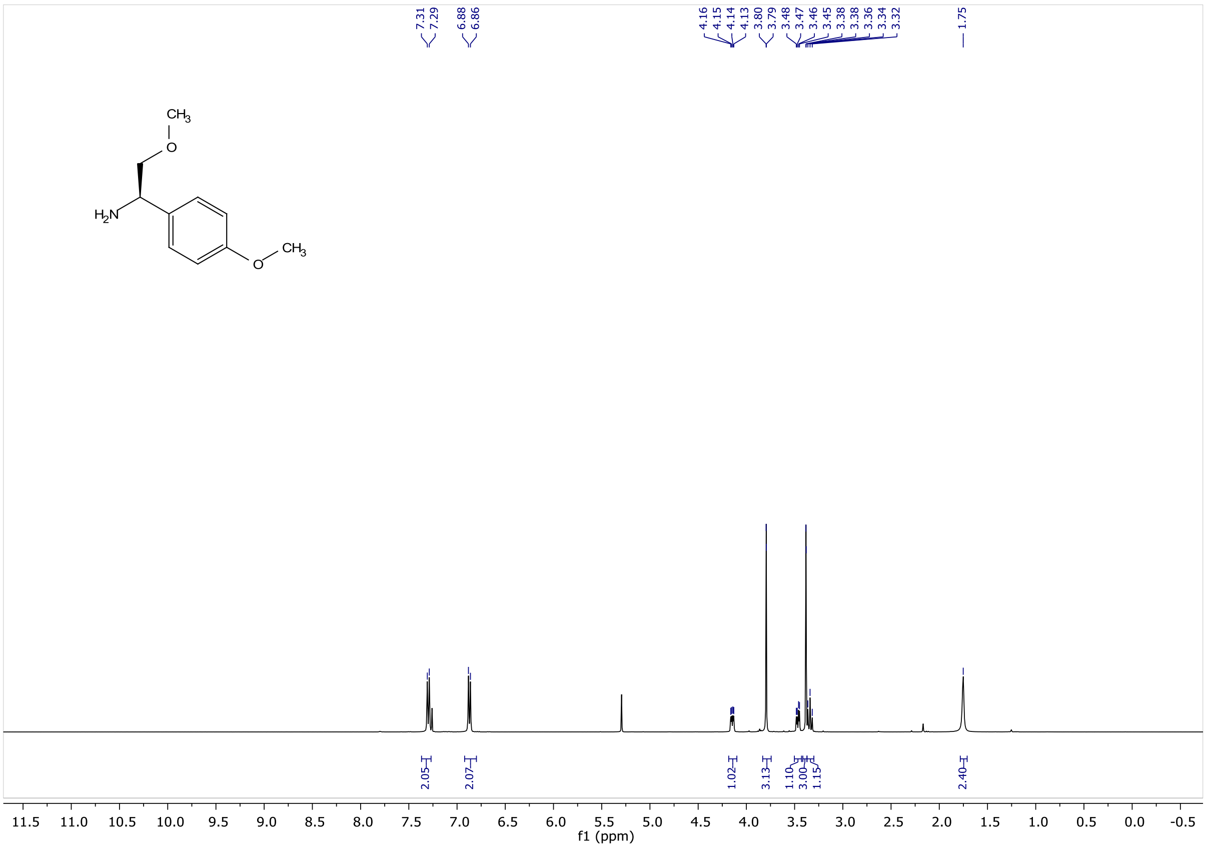

^{13}C NMR (101 MHz, CDCl$_3$)

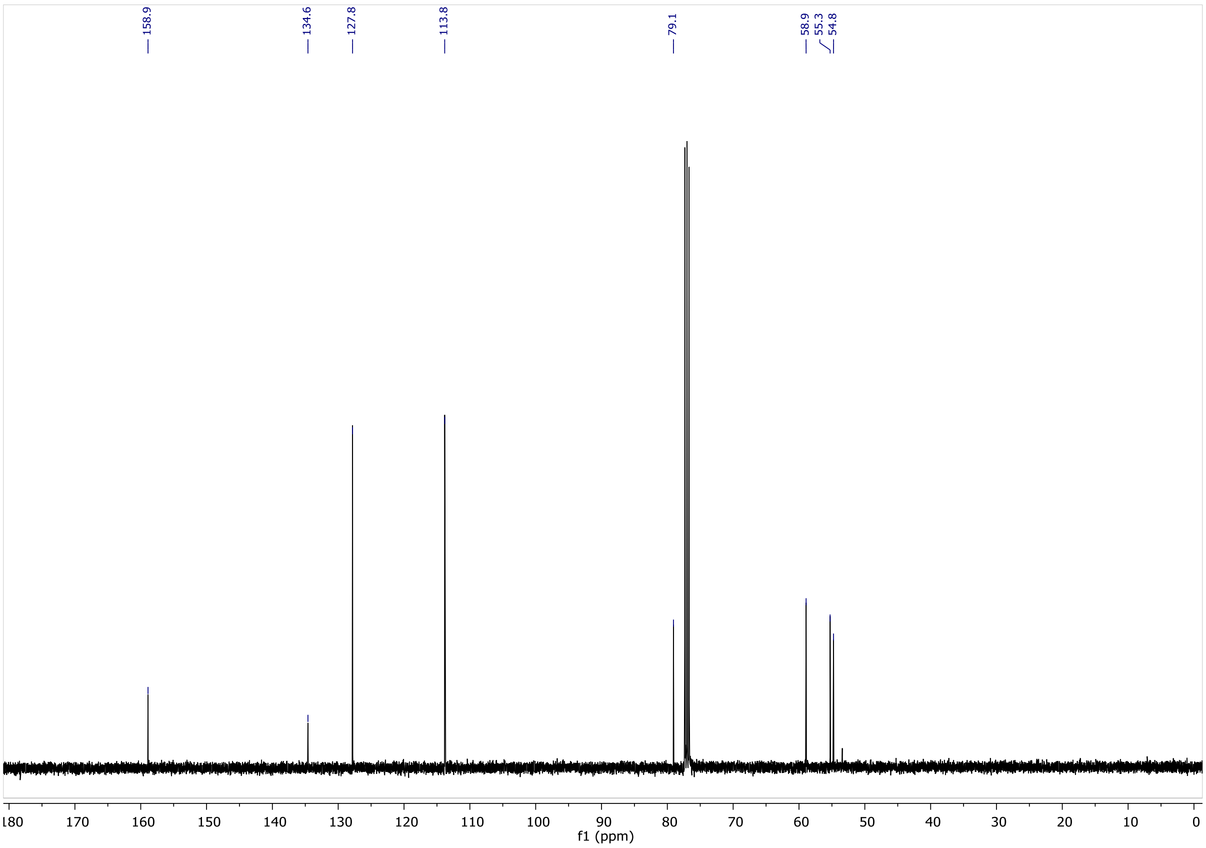

(S)-2-(benzyloxy)-1-(3,5-bis(trifluoromethyl)phenyl)ethan-1-amine, 4e ^{1}H NMR (500 MHz, $CDCl_3$)

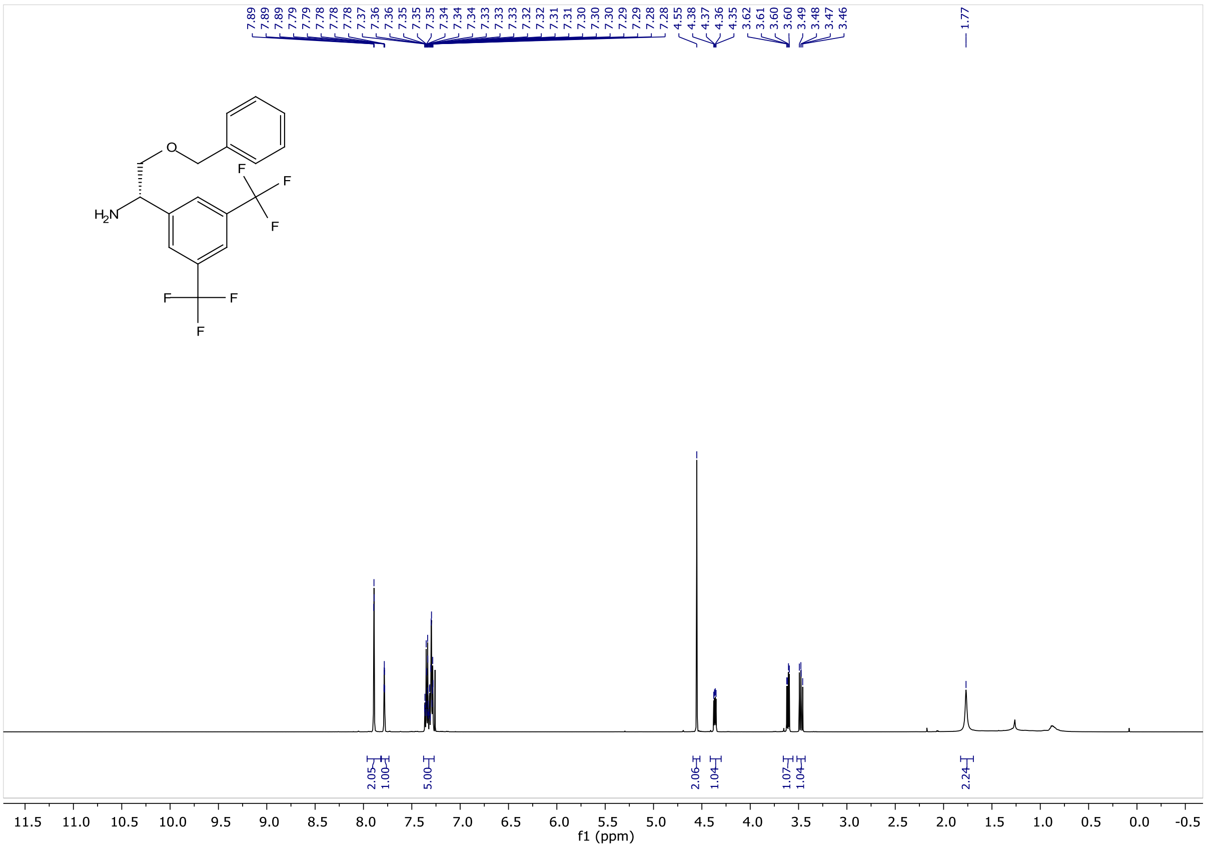

^{13}C NMR (126 MHz, $CDCl_3$)

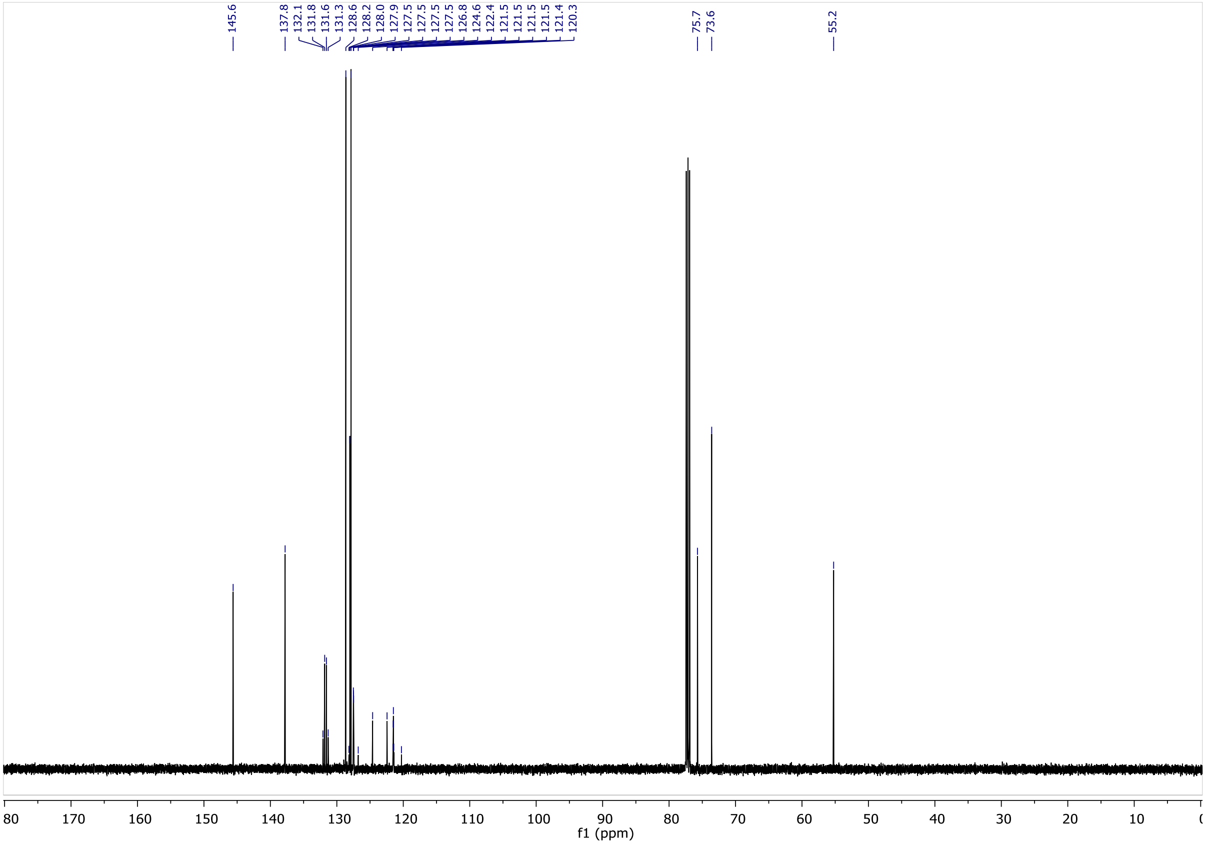

^{19}F (376 MHz, $CDCl_3$)

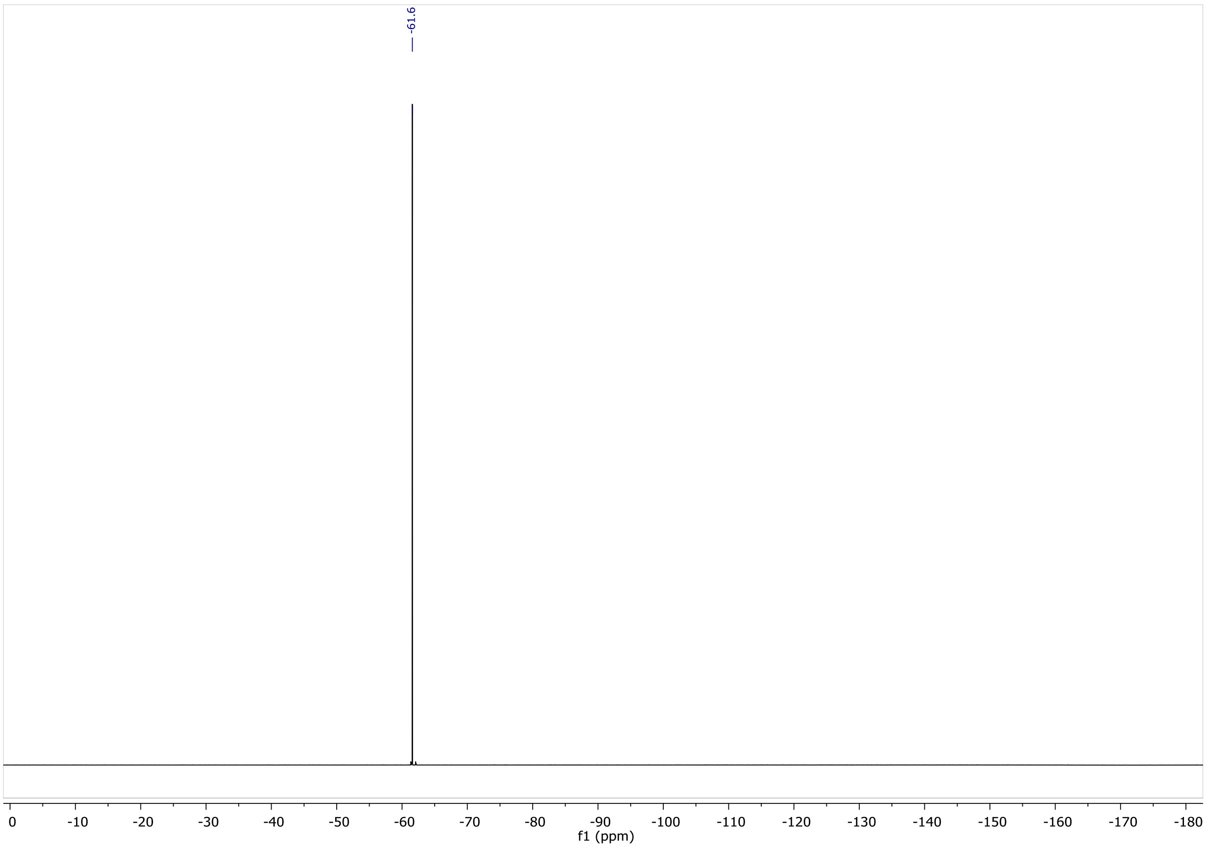

5-methyl-5-(tetrahydro-2H-pyran-4-yl)pyrrolidin-2-one, 6 ^{1}H NMR (500 MHz, $CDCl_3$)

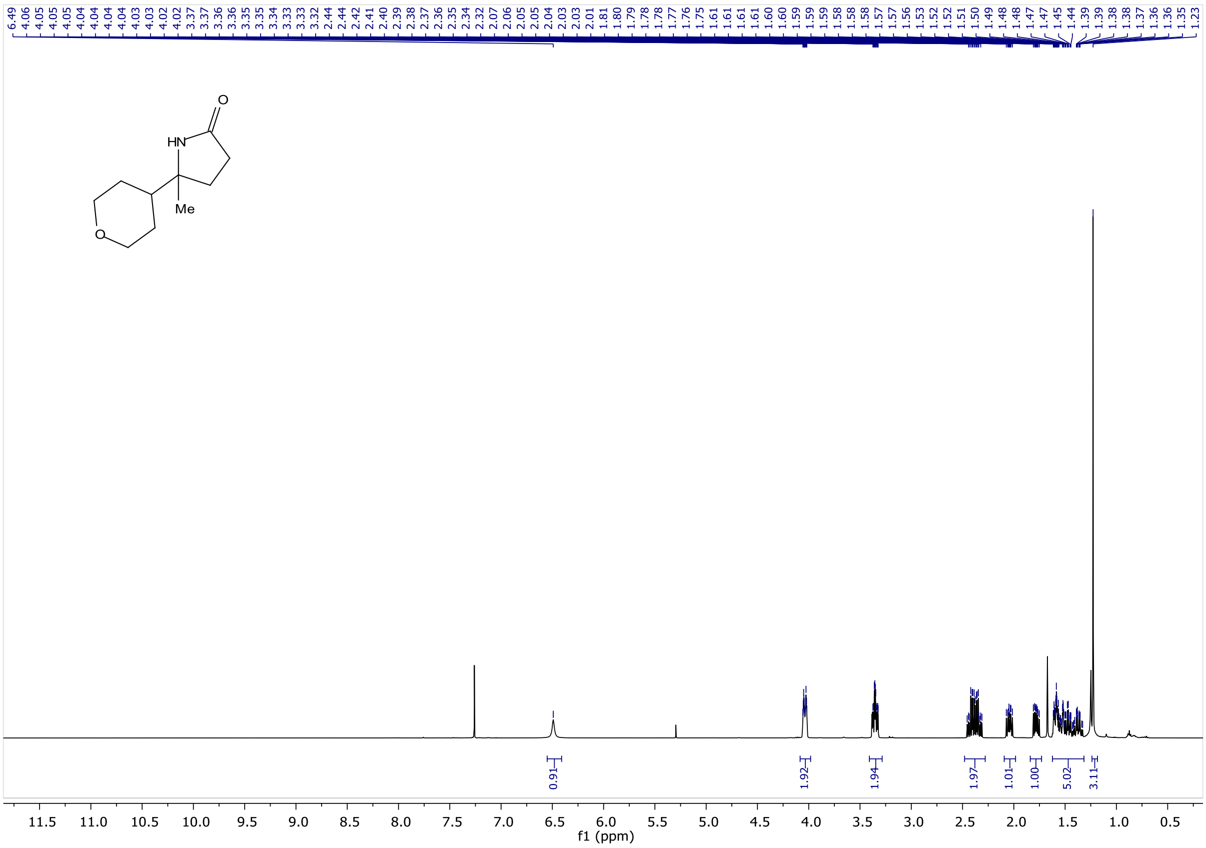

^{13}C NMR (126 MHz, $CDCl_3$)

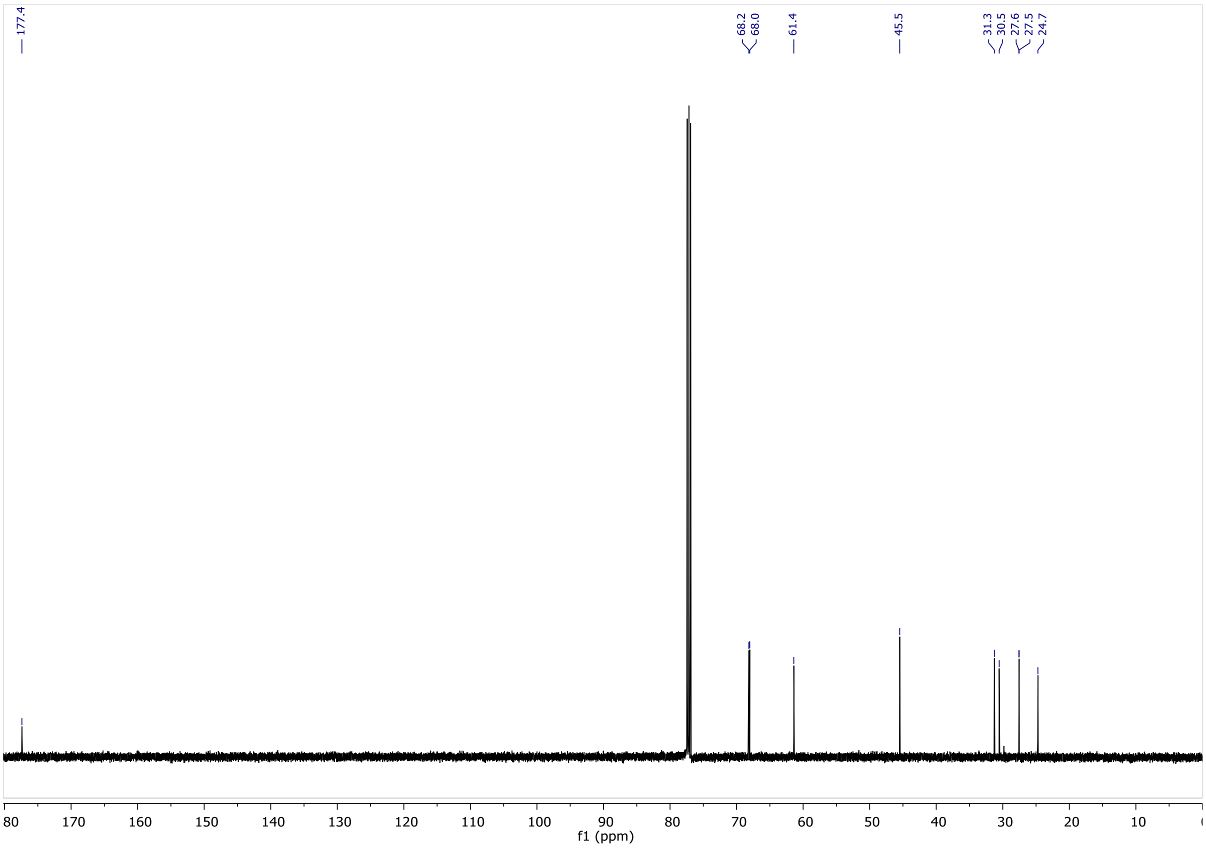

2-methyl-2-(tetrahydro-2H-pyran-4-yl)pyrrolidine, 7 ^{1}H NMR (500 MHz, $CDCl_3$)

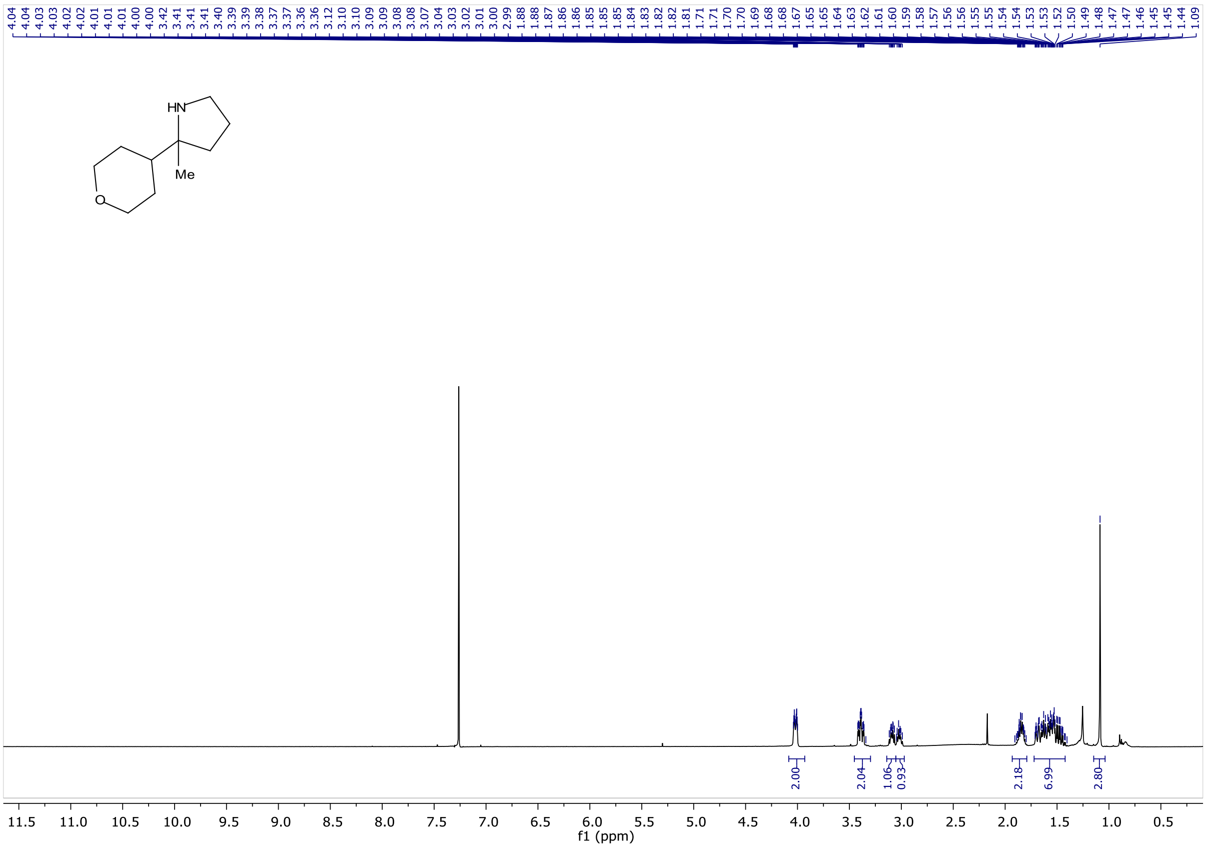

^{13}C NMR (126 MHz, $CDCl_3$)

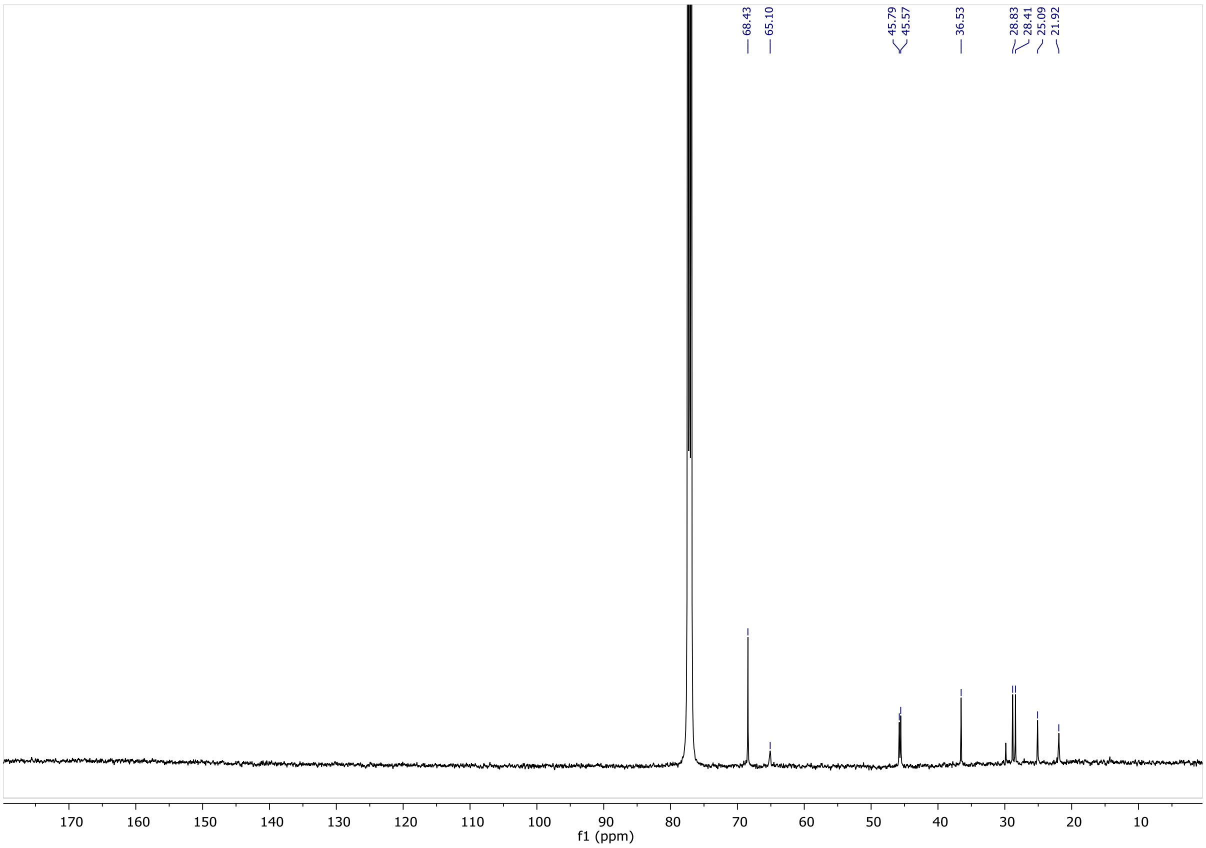

***tert*-butyl 3-(4-aminotetrahydro-2*H*-pyran-4-yl)-2-methylpropanoate, 8** ^{1}H NMR (400 MHz, $CDCl_3$)

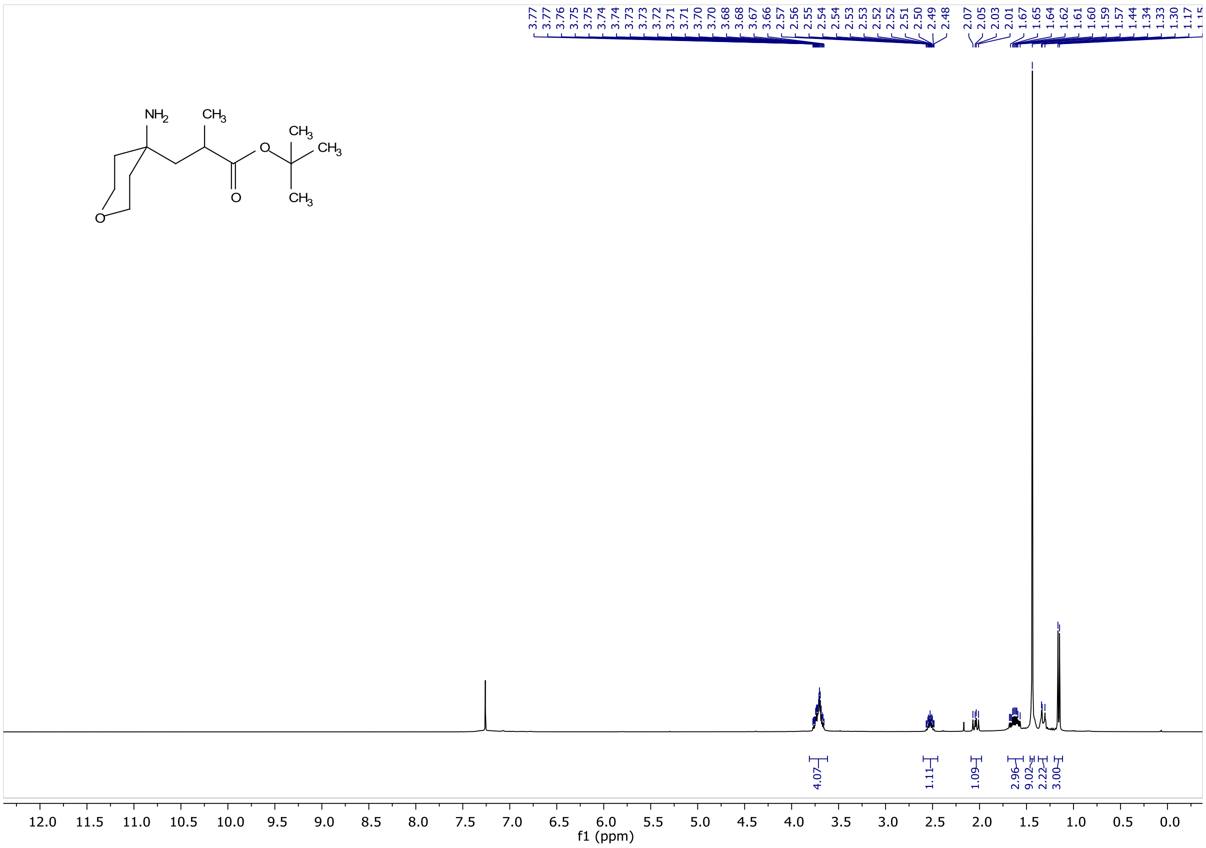

^{13}C NMR (101 MHz, $CDCl_3$)

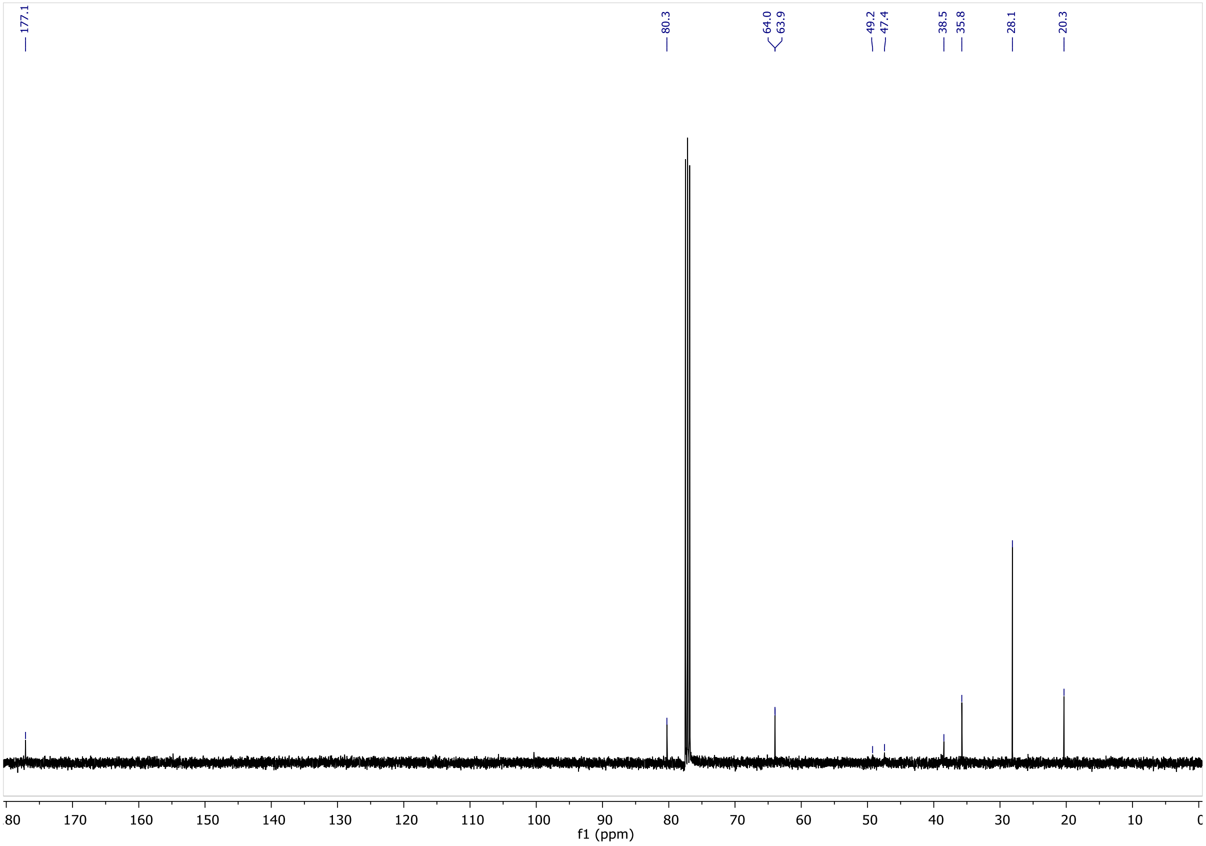

Supplement: Supplementary file 1 — ol2c04308_si_001.pdf [file ol2c04308_si_001.pdf]
